# Dietary Patterns and Non-Communicable Disease Biomarkers: A Network Meta-Analysis and Nutritional Geometry Approach

**DOI:** 10.3390/nu15010076

**Published:** 2022-12-23

**Authors:** Shuang Liang, Jovana Mijatovic, Ang Li, Nicholas Koemel, Reeja Nasir, Clémence Toniutti, Kim Bell-Anderson, Michael Skilton, Fiona O’Leary

**Affiliations:** 1 Faculty of Medicine and Health, The University of Sydney, Sydney, NSW 2006, Australia; 2 Centre for Health Policy, Melbourne School of Population and Global Health, The University of Melbourne, Melbourne, VIC 3010, Australia; 3 The Charles Perkins Centre, The University of Sydney, Sydney, NSW 2006, Australia; 4 Faculty of Science, The University of Sydney, Sydney, NSW 2006, Australia; 5 Sydney Institute for Women, Children and Their Families, Sydney Local Health District, Sydney, NSW 2050, Australia

**Keywords:** systematic review, diet, biological markers, intervention trials, chronic disease, GRADE

## Abstract

Quantitative rankings of multiple dietary patterns for their effects on non-communicable disease (NCD) biomarkers is lacking and would inform primary prevention strategies. Accordingly, a network meta-analysis (NMA) was conducted to compare and rank the effects of different dietary patterns on NCD biomarkers, and associations of dietary patterns’ underlying macronutrient composition with NCD biomarkers were determined by a nutritional geometry approach. Randomised controlled trials (RCTs) were eligible for inclusion if they enrolled healthy participants, employed food-based dietary pattern interventions without energy restriction, and reported NCD biomarker outcomes. NCD biomarkers were included as an outcome if ≥10 trials were available. A systematic search of five electronic databases identified 4008 records. Sixty-eight articles from 59 RCTs reporting lipids, glycemic, and inflammatory biomarkers were included for quantitative syntheses. Risk-of-bias was predominantly categorized as low or having some concerns, and confidence-of-evidence low. Relative to western habitual diet, the Mediterranean, Dietary Approaches to Stop Hypertension (DASH), dietary guidelines-based, plant-based, and low-fat diets reduced low-density lipoprotein cholesterol (mean difference range: −0.29 to −0.17 mmol/L), total cholesterol (−0.36 to −0.24 mmol/L), and apolipoprotein B (−0.11 to −0.07 g/L) (all *p* < 0.05); the Paleo, plant-based and dietary guidelines-based diets reduced homeostasis model assessment of insulin resistance (−0.95 to −0.35, all *p* < 0.05). No dietary pattern ranked consistently highest. The Paleo diet received the highest all-outcomes-combined average Surface Under the Cumulative Ranking Curve value (67%), followed by DASH (62%) and Mediterranean diets (57%), whereas western habitual diet was lowest (36%). Our findings were independent of macronutrient composition, highlighting the significance of dietary pattern-level analysis.

## 1. Introduction

### 1.1. Dietary Patterns and Non-Communicable Diseases

Non-communicable diseases (NCDs), including obesity, cardiovascular diseases, diabetes, and cancers, are a major and ongoing burden to global health systems, accounting for 71% of all deaths [1]. Diet is an important modifiable risk factor for NCDs. The Global Burden of Disease study showed that nearly eight million deaths and 188 million disability adjusted life years were attributable to dietary risk factors worldwide in 2019 alone [2]. An approach that focuses on individual nutrients, and, to a lesser extent, food groups, fails to account for the synergistic and antagonistic effects of nutrient, phytochemical and antinutrient combinations within meals. An alternative approach is describing dietary intake at the dietary pattern-level. Dietary patterns take the quality, quantity, and frequency of dietary intake into consideration, inherently accounting for nutrient-nutrient interactions, intercorrelations and food matrix characteristics. As such, dietary patterns provide a more holistic indication of the net effects of dietary intake, in addition to being more relevant, and more easily translatable, to real-world dietary intake [3].

### 1.2. Biomarkers of Non-Communicable Diseases

Biomarkers of NCDs can either be risk factors or reflect intermediate and/or disease phenotypes and play an important role in understanding diet-disease relationships [4,5]. NCD biomarkers, in particular laboratory-measured markers, are objective and widely accepted as surrogate outcomes in research, including clinical trials, and in clinical practice as part of NCD prevention strategies [6]. Blood lipid levels are established risk factors for NCDs [7,8,9], some of which have shown causal relationships with various cardiovascular diseases [10,11,12]. Other commonly measured NCD biomarkers include those that reflect glycemic control including insulin resistance [13], which itself is associated with risk of diabetes and cardiovascular disease [14,15,16,17,18]. In addition, biomarkers that indicate chronic low-grade inflammation have been shown to play a pivotal mechanistic role in the development and progression of atherosclerosis [19,20,21], and are associated with risk of incident cardiovascular disease events [22,23,24]. More recently, chronic low-grade inflammation has been associated with increased risks for a wider range of NCDs across diverse ethnic groups [25].

### 1.3. A Role for Network Meta-Analysis

Several meta-analyses have investigated the effects of a single dietary pattern on NCD biomarkers [26,27,28]. This approach limits the comparison of multiple dietary patterns within a consistent framework. Network meta-analysis (NMA) is a powerful extension to traditional meta-analysis which uses both direct and indirect evidence within a connected network of trials to compare and rank multiple treatments [29]. Several previous NMAs have described the comparative effects of dietary patterns in NCD management [28,30,31,32,33,34,35]. Those NMAs were predominantly conducted in populations at high risk or with pre-existing disease, such as type 2 diabetes [28,32,33,34], hypertension [35], and/or obesity [28,30,31]. Comparison of dietary patterns for primary prevention remains limited despite dietary patterns increasingly being the primary focus of dietary guidelines [36].

Accordingly, we conducted a systematic review with an NMA of randomised controlled trials to compare and rank the effects of various dietary patterns on common NCD biomarkers. We hypothesized that the Mediterranean diet would be among the most highly ranked overall based.

## 2. Materials and Methods

This systematic review is conducted as per the Preferred Reporting Items for Systematic Reviews and Meta-Analyses (PRISMA) extension statement for network meta-analysis [37]. We registered our systematic review in PROSPERO (Registration no. CRD42019129839); this registration specifically covered a broad review on dietary patterns and biochemical markers. Results of the association of dietary patterns and nutritional biomarkers has been published [38]. This current NMA is of an exploratory analysis derived from papers retrieved as part of the registered protocol, specifically relating to NCD biomarkers. The PRISMA NMA checklist is included as Supplementary Table S1.

### 2.1. Literature Search and Study Selection

**Figure 1:**
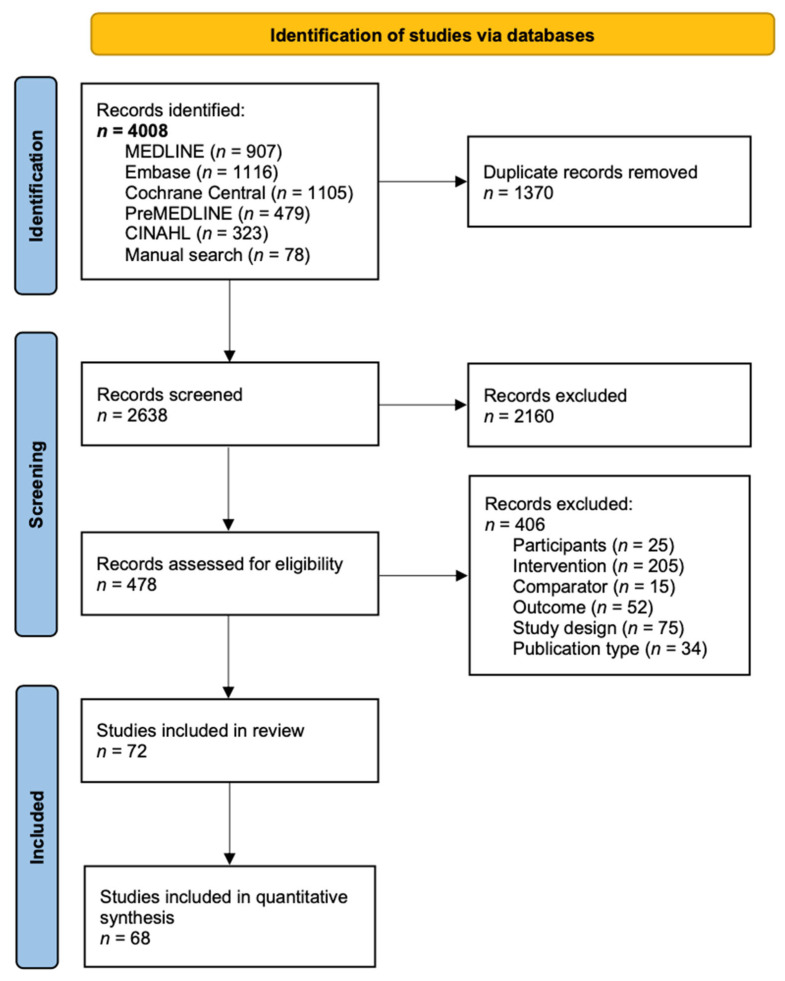
PRISMA flow diagram of study selection.

The PRISMA flow diagram of study selection is presented in Figure 1. A systematic search was conducted in MEDLINE, Embase, Cochrane Central, PreMEDLINE, and CINAHL until 16 November 2020. The full electronic search strategy for MEDLINE is presented in Supplementary Document S1. A manual search of any relevant publications in the literature from additional sources and citations in included publications was performed to supplement the electronic database search until 1 August 2021.

Publications were included if they met all of the following inclusion criteria: (1) randomised controlled trial (RCT) study design; (2) healthy adult participants that were not selected specifically based on disease diagnosis, including studies in which some of the participants have a chronic disease; (3) food-based interventions, which were not achieved by nutritional supplementation, and have a focus on dietary patterns that are pre-defined, or based on a priori pattern indexes, or derived using a posteriori methods, e.g., principal component analysis, cluster analysis, and reduced rank regression; (4) having an appropriate comparator, i.e., a different dietary pattern, and (5) biochemical markers that are laboratory-measured and are relevant to NCD risk stratification or prevention. Given the exploratory nature of this review, no biomarkers were pre-specified. Publications were excluded if they met any of the following exclusion criteria: (1) all participants had a current diagnosis of metabolic syndrome, diabetes, cardiovascular diseases, cancers, or other diseases; (2) study participants were pregnant or breastfeeding; (3) the dietary intervention focused on one component of the diet, such as a single nutrient, individual food or food group; (4) the intervention involved nutritional supplementation or energy restriction, and the control group(s) differed in less than two components of the intervention diet; (5) article not written in English language, and (6) research only published in the form of a scientific abstract. No limits on publication year, study country, or intervention duration were applied.

Eligible publications along with their reported NCD biomarkers were recorded as candidates for full data extraction. NCD biomarkers with data available from at least 10 different trials were included as an outcome to ensure adequate statistical power and to reduce the likelihood of publication bias [30,39], given the exploratory nature of this NMA. Only one set of data was included from the same trial, and the chosen data was based on the largest sample size, and/or the longest intended intervention duration.

Title and abstract screening were undertaken by one reviewer (Shuang Liang), with a random sample of 12% of all abstracts double coded by a second reviewer (Fiona O’Leary), and 100% agreement was reached. Full text of the articles was screened in duplicate by three reviewers independently (Shuang Liang, Reeja Nasir, and Clémence Toniutti) using reference management software (EndNote, version 9, Clarivate Analytics, Philadelphia, PA, USA). Any discrepancies were resolved by consultation with reviewers (Fiona O’Leary, Michael Skilton, Kim Bell-Anderson, and Shuang Liang).

### 2.2. Data Syntheses and Statistical Analyses

Data were extracted from each of the identified articles by two reviewers (Shuang Liang and Jovana Mijatovic) independently, using a data collection form developed and pilot-tested according to the Cochrane handbook [40]. Data extracted included first author, publication year, study origin (country), study design (RCT: parallel or crossover), population size, participant characteristics (age, sex, and health status), study timeframe, description of dietary patterns, dietary compliance assessment, end-point values with corresponding standard deviations for disease biomarkers and biological compartment. Where the end-point values with the corresponding standard deviations (SD) were not readily available, the baseline and change from baseline values were used to impute the end-point values, the change scores were used if not possible to impute [40]. Where median and confidence intervals/interquartile range were reported, the mean and SD were estimated [41,42,43]. If impossible to estimate, the SD was imputed from a similar article based on the intervention dietary patterns, intervention duration, sample size, and population characteristics [40]. The biomarker concentrations were converted to standard units. We contacted authors for missing data, 6 of 11 contacted authors provided additional information.

Cochrane risk-of-bias tool version 2 [44] was followed by two reviewers independently (Shuang Liang and Jovana Mijatovic) to assess the risk of bias of each publication. Any discrepancies were resolved through discussion. A total of five domains were assessed: (1) bias arising from the randomisation process; (2) bias due to deviations from intended interventions; (3) bias due to missing outcome data; (4) bias in measurement of the outcome and (5) bias in selection of the reported result. Risk of bias judgements were made as “Low risk of bias”, “Some concerns” and “High risk of bias” at domain levels and overall risk of bias judgement was given per the criteria set in the Cochrane risk-of-bias tool version 2 (Cochrane Collaboration, London, UK).

The Confidence in Network Meta-Analysis (CINeMA) framework [45] was followed to assess the confidence of evidence. CINeMA is the extension based on the Grading of Recommendations Assessment, Development and Evaluation (GRADE) system, with the consideration of six domains: within-study bias, reporting bias, indirectness, imprecision, heterogeneity, and incoherence [46].

We illustrated the available direct comparisons between different dietary patterns using a network diagram for each outcome. The size of the nodes is proportional to the sample size of each dietary pattern intervention, and the thickness of the lines is proportional to the number of studies available. The assumption of transitivity was assessed by comparing the distribution of the potential effect modifiers including participants’ age, percentage of female participants, and intervention duration across the available direct comparisons [47,48]. Given the generally healthy populations in scope, the distribution of BMI was not assessed.

Quantitative syntheses were conducted including studies with arithmetic means (*n* = 68), i.e., studies reporting geometric means or medians that could not be imputed were excluded from the analyses (*n* = 4). For each outcome of interest, a random effects NMA was conducted to estimate all possible pairwise relative effects and to obtain a relative ranking of the different dietary patterns. The summary mean differences with corresponding 95% CI were calculated. The effect sizes were deemed as statistically significant at 0.05 level. The relative ranking of the different dietary patterns for each outcome was assessed using the distribution of the ranking probabilities and the surface under the cumulative ranking curves (SUCRA) [49]. The SUCRA is a value with a range from 0 to 100% that is associated with each treatment in a connected network. A higher SUCRA indicates a greater chance of the treatment being the best for achieving a favourable outcome, e.g., a lower LDL cholesterol level, or a higher high-density lipoprotein (HDL) cholesterol level. For each of the dietary patterns, a category-level combined SUCRA was calculated by averaging biomarker-level SUCRAs in a category, and an all-outcomes-combined SUCRA was calculated by averaging category-level combined SUCRAs. We used the loop-specific approach for the detection of loops of evidence that might present important inconsistency [50]. A common heterogeneity (between-study variance) for comparisons within each loop is assumed. We performed side-splitting approach to detect any potential inconsistency present between direct and indirect evidence [51]. We applied the global methods to jointly investigate the presence of inconsistency from all possible sources in the entire network simultaneously using the design-by-treatment interaction model [52,53]. The presence of small-study effects for each outcome was evaluated by drawing a comparison-adjusted funnel plots that adjusts for different dietary pattern comparisons being included [54]. Sensitivity analyses were conducted to reflect the following themes: (1) Risk of bias, by removing studies considered being at high risk; (2) Intervention duration, by removing studies with the intervention longer than 52 weeks, as dietary compliance tends to decline as the intervention duration increases [55]; (3) Participants’ age, by removing studies which had a population with a mean age of 70 years and above, as most NCDs attributed “premature” deaths occur in the population under the age of 70 years [1]; and (4) high sensitivity C-reactive protein (hsCRP) levels, by removing studies reporting a plasma concentration greater than 10 mg/L, as a higher hsCRP level suggests acute infection or inflammation, rather than low-grade inflammation that is relevant for NCD risk [56]. The network meta-analyses were performed and presented using the *network* package [57] and *network graphs* package [58] in Stata version 16.0 (StataCorp, College Station, TX, USA).

A nutritional geometry approach [59] was applied to determine whether the association of macronutrient composition with NCD biomarkers in these trials is consistent with the effects of dietary patterns as determined by the NMA. For each outcome of interest, five mixture models [60] were fitted to test the linear and non-linear associations between macronutrients, as a percentage of dietary energy, and the NCD biomarker; Model 1 represents the null model where there is no association with macronutrient composition, Model 2 reflects linear associations, Model 3 reflects quadratic associations, and Models 4 and 5 reflect cubic associations. Models were compared and the preferred model determined by the lowest Akaike Information Criterion value. The effects predicted by the Akaike Information Criterion-favoured model was visualised as response surfaces in right-angle mixture triangles. Each space on these right-angle mixture triangles represents 100% of dietary energy, being the sum of the *x*-axis (fat), the *y*-axis (carbohydrate), and an inferred *z*-axis (protein). The values of predicted effects are presented on the response surface as contour lines, with the distribution of the surface limited to the intakes observed in the included dietary studies. Mixture models were built using the “MixModel” function in the R program *mixexp* package version 1.2 (R Core Team, Vienna, Austria).

## 3. Results

### 3.1. Data Availability and Study Characteristics

Of the 4008 records retrieved from literature search, 478 full texts were screened, and 72 articles met the inclusion criteria. A total of 68 articles were derived from 59 RCTs were included in NMAs and geometric framework for quantitative syntheses, including 56 for HDL cholesterol, 55 for triglycerides, 54 for LDL cholesterol, 53 for total cholesterol, 39 for glucose, 32 for insulin, 28 for hsCRP, 17 for apoB, 16 for apoA-1, 13 for Homeostatic Model Assessment for Insulin Resistance (HOMA-IR), and 8 for IL-6. All trials conducted some form of dietary assessment to monitor the adherence to the dietary pattern intervention, and/or have partially or fully provided the food, and/or have supervised the consumption; however, one trial did not report these results. The most commonly used assessment methods included weighted or unweighted food records, 24-h dietary recalls, diet history, food frequency questionnaires, and food checklists. Five trials have incorporated nutritional biomarkers for dietary assessment. The main characteristics of included studies are shown in Supplementary Table S2. The characteristics and biomarker levels of the studies excluded from statistical analyses are detailed in Supplementary Table S3. The dietary patterns examined included the Mediterranean diet, DASH diet, Paleo diet, plant-based diet, diet patterns based on dietary guidelines, low Glycemic Index (GI)/Glycemic Load (GL) diet, high GI/GL diet, low-fat diet, low carbohydrate high-fat diet, traditional Mexican diet, and western habitual diet. Although we did not restrict the method used for deriving dietary patterns, all articles included in this NMA reported dietary patterns that were defined a priori, i.e., pre-described, or based on pattern indexes. Intervention duration ranged from 10 days to 5 years. The mean age of participants ranged from 23.1 to 70.9 years.

Detailed risk of bias assessment is shown in Supplementary Table S4. Twenty-two articles were assessed as low risk of bias. Thirty-nine articles were judged as having some concerns and seven as high risk of bias, with inadequate information on randomisation process raising the risk of bias for most articles affected. Furthermore, while it is almost impossible to achieve blinding in dietary studies, the Cochrane risk-of-bias tool notes that lack of blinding does not necessarily lead to a higher risk-of-bias unless there is evidence of deviations arising due to the trial content. We considered the likelihood of such deviations on a case-by-case basis and found them to be generally low.

### 3.2. Network Meta-Analysis (Dietary Patterns and NCD Biomarkers)

The network diagrams for each of the NCD biomarkers are shown in Supplementary Figures S1–S3 (LDL cholesterol, Figure 2). The league tables summarising the effect size estimates for comparisons between all dietary patterns in the network are shown in Supplementary Tables S5–S14 (LDL cholesterol, Table 1), with results for each individual biomarker described below. The SUCRA values (Table 2) provide a relative ranking of the dietary patterns for each individual biomarker and for combined outcomes. There was no indication of violated transitivity according to the distribution of potential effect modifiers, although for some comparisons there were insufficient studies for appropriate examination (Supplementary Figures S4–S14). Rankograms for all NCD biomarker outcomes show distinct ranking distributions between best and worst dietary patterns (Supplementary Figures S15–S25), indicating relatively high precision of the rankings.

#### 3.2.1. Individual Lipids and Apolipoproteins

The Mediterranean diet, DASH diet, dietary guidelines-based diets, plant-based diets, and low-fat diets effectively reduced total cholesterol, LDL cholesterol, and apoB when compared to the western habitual diet (effect sizes range from −0.36 to −0.24 mmol/L for total cholesterol, −0.29 to −0.17 mmol/L for LDL cholesterol, and −0.11 to −0.07 g/L for apoB, all *p* < 0.05). When compared to the low carbohydrate high-fat diet, the Mediterranean diet, DASH diet, plant-based diet and low-fat diet were more effective for reducing total cholesterol (−0.48 to −0.42 mmol/L, all *p* < 0.05); the Mediterranean diet, plant-based diet and low-fat diet were more effective for reducing LDL cholesterol (−0.37 to −0.33 mmol/L, all *p* < 0.05); and the Mediterranean diet, DASH diet, dietary guidelines-based diet and low-fat diet were more effective for reducing apoB (−0.17 to −0.15 g/L, all *p* < 0.05). The confidence ratings were moderate (13%), low (53%) and very low (33%) for LDL cholesterol, and were moderate (2%), low (67%), and very low (31%) for total cholesterol.

The low carbohydrate high-fat diet significantly improved HDL cholesterol when compared to the DASH diet, plant-based diet, low-fat diet, dietary guidelines-based diets, and Mediterranean diet (effect size (95% CI): 0.19 (0.08, 0.30), 0.19 (0.09, 0.29), 0.17 (0.06, 0.28), 0.16 (0.05, 0.26), and 0.12 (0.02, 0.22) mmol/L, respectively); and the Mediterranean diet resulted in a marginal improvement in HDL cholesterol levels when compared to the plant-based diet, the DASH diet, and the low-fat diet (effect size (95% CI): 0.07 (0.02, 0.13), 0.07 (0.00, 0.14), and 0.05 (0.00, 0.10) mmol/L, respectively). A number of dietary patterns resulted in a reduction in HDL cholesterol when compared to the western habitual diet. These included the DASH diet, plant-based diet, low-fat diet, and dietary guidelines-based diets (effect size (95% CI): −0.10 (−0.15, −0.04), −0.10 (−0.14, −0.05), −0.08 (−0.12, −0.03), and −0.06 (−0.11, −0.02) mmol/L, respectively). The confidence ratings for HDL cholesterol were high (4%), moderate (27%), low (33%), and very low (36%). The low carbohydrate high-fat diet showed significant beneficial effects on apoA-1 and triglycerides when compared to the plant-based diet (effect size (95% CI): 0.22 (0.02,0.42) g/L and −0.22 (−0.44, −0.01) mmol, respectively).

The low carbohydrate high-fat diet was ranked the best at increasing HDL cholesterol and apoA-1, and for reducing triglycerides (SUCRA: 92.6%, 93.4%, and 91.3%, respectively); the plant-based diet, Mediterranean diet, and DASH diet were respectively the best at reducing total cholesterol, LDL cholesterol, and apoB (SUCRA: 76.0%, 79.2%, and 77.9%, respectively).

#### 3.2.2. Individual Glycemic Biomarkers

The Paleo diet, plant-based diet, and dietary-guidelines based diets were the most effective for lowering HOMA-IR when compared to the western habitual diet (effect size (95% CI): −0.95 (−1.68, −0.21), −0.90 (−1.21, −0.59), and −0.35 (−0.59, −0.10), respectively). Additionally, the plant-based diet was more effective in HOMA-IR reduction when compared to the low-fat diet, Mediterranean diet, and dietary guidelines-based diets (effect size (95% CI): −0.75 (−1.21, −0.29), −0.72 (−1.21, −0.23), −0.55 (−0.95, −0.16), respectively); and the Paleo diet was more effective relative to the low-fat diet (effect size (95% CI): −0.80 (−1.56, −0.03)). The confidence ratings for HOMA-IR were moderate (14%), low (61%) and very low (25%). The plant-based diet was ranked the best for reducing HOMA-IR (SUCRA 90.4%).

Only the low-fat diet showed a significant but relatively small reduction in fasting insulin concentrations when compared to the western habitual diet [effect size (95% CI): −3.09 (−6.18, −0.00) mU/L], and had the highest SUCRA value for insulin (78.5%). No dietary pattern significantly altered fasting glucose levels compared to other dietary patterns, although the low carbohydrate high-fat diet was ranked the best for glucose (SUCRA 80.8%).

#### 3.2.3. Individual Inflammatory Biomarkers

No dietary pattern significantly affected hsCRP or IL-6 levels. The confidence ratings for hsCRP were moderate (43%), low (43%), and very low (14%). The Paleo diet had the highest SUCRA values for both hsCRP and IL-6 (79.0% and 95.0%, respectively).

#### 3.2.4. Summary across Outcomes

The Mediterranean diet had the highest average SUCRA value (63.1%) in the lipids and apolipoproteins category; a low carbohydrate high-fat diet was the highest (66.4%) in the glycemic control category; and the Paleo diet was the highest (87.0%) in the inflammation category.

When combining all three categories, the Paleo diet had the highest average SUCRA value (67.2%), followed by the DASH (62.4%) and the Mediterranean diet (57.4%). The western habitual diet performed the worst (35.6%). The overall SUCRA values were not available for the low GI/GL, high GI/GL, and traditional Mexican diets as there was no data for at least one biomarker category.

#### 3.2.5. Inconsistency, Sensitivity Analyses, Publication Bias, and Confidence of Evidence

Based on the loop-specific approach, no inference was made for inconsistency for LDL cholesterol, HDL cholesterol, triglycerides, apoB, apoA-1, glucose, and HOMA-IR. Borderline inconsistency was inferred for total cholesterol in the loops between the Mediterranean diet, dietary guideline-based diets and low-fat diet and between dietary guideline-based diets, plant-based diets and low-fat diet; and inconsistency for insulin, especially in the loop of the Mediterranean diet, low-fat diet and western habitual diet (Supplementary Tables S15–S24). IL-6 was not eligible for this method as no multiple-arm (≥ 3) study was included. The side-splitting approach showed no inconsistency for apoA-1, glucose, HOMA-IR, hsCRP, and IL-6. Inconsistency was observed for lipids (LDL cholesterol, HDL cholesterol, total cholesterol, triglycerides, and apoB), and insulin, for some of the comparisons between the Mediterranean diet, dietary guidelines-based diets, low-fat diet, plant-based diet, and western habitual diet (Supplementary Tables S25–S35). The design-by-treatment method showed no inconsistency for hsCRP (*p* = 0.96), IL-6 (*p* = 0.61), glucose (*p* = 0.43), total cholesterol (*p* = 0.59), LDL cholesterol (*p* = 0.55), apoA-1 (*p* = 0.30), and HOMA-IR (*p* = 0.96); and suggested inconsistency for triglycerides (*p* < 0.001), HDL cholesterol (*p* = 0.004); insulin (*p* = 0.002) and apoB (*p* = 0.04).

Sensitivity analyses resulted in similar results for the majority of applicable biomarker outcomes, except for exclusion of studies with a high risk of bias resulted in additional significant findings for insulin, with the Paleo, DASH, and dietary guideline-based diets showing significant beneficial effects compared to the Mediterranean diet or low-fat diet; and additional estimated effects achieved statistical significance for hsCRP and apoA-1 (Supplementary Tables S36–S62).

In comparison-adjusted funnel plots, no asymmetry was detected for any of the outcomes (Supplementary Figures S26–S36), consistent with no evidence of publication bias. Overall confidence in the evidence for dietary pattern comparisons were high (1%), moderate (18%), low (51%), and very low (29%) (Supplementary Tables S63–S67).

### 3.3. Nutritional Geometry Approach (Macronutrients and NCD Biomarkers)

The relationship between macronutrient composition and NCD biomarkers is shown in Supplementary Figures S37–S39. Using trial-level data, macronutrient composition was associated with total cholesterol, HDL cholesterol, and apoA-1 (*p* < 0.001 for all; Supplementary Table S68), but not for other NCD biomarkers included in this review. The lowest total cholesterol was associated with a diet comprised of approximately 15% energy from protein, 80% from carbohydrate, and 5% from fat, while the highest was seen in the diets with 25% energy from protein, 5% from carbohydrate, and 70% from fat. The highest HDL cholesterol levels were associated with diets comprised of 30% energy from protein, ≤40% from carbohydrate, and ≥35% from fat, while the lowest was found in the area with 15% energy from protein, ≥50% from carbohydrate, and ≤45% from fat. The highest apoA-1 was associated with diets comprised of 25% energy from protein, 5% from carbohydrate, and 70% from fat, whereas the lowest was seen when the composition was 10% energy from protein, 70% from carbohydrate, and 20% from fat.

## 4. Discussion

We compared up to 11 dietary patterns for their effects on 11 NCD biomarkers. The Mediterranean diet was ranked best for improving lipid profiles, a low carbohydrate high-fat diet was best for glycemic control, and the Paleo diet was ranked highest for the inflammatory biomarkers category. Across all outcomes, the Paleo diet had the highest all-outcomes-combined average SUCRA value (67.2%), followed by the DASH diet (62.4%) and Mediterranean diet (57.4%). The western habitual diet received the lowest overall average SUCRA (35.6%). The confidence in the evidence was rated low for the majority of dietary patterns and their influences on NCD biomarkers indicating more research is needed.

Our results indicate potential benefits of the Paleo diet for improving biomarker risk profile, and, as such, potentially for NCD prevention. In concordance with our findings, emerging evidence has demonstrated health benefits of the Paleo diet [61,62]. In a pair-wise meta-analysis, the Paleo diet was associated with a significant decrease in cardiovascular disease risk factors, including blood lipids and CRP, even when compared to a heterogeneous control group that included various well-documented healthy dietary patterns, such as the Mediterranean diet and diets based on dietary guidelines [62]. Similar to previous NMAs in populations with type 2 diabetes, the Paleo diet was ranked among the top dietary patterns for several blood lipids, such as triglycerides and HDL cholesterol [32], and glycemic outcomes including fasting blood glucose and HbA1c [28,34]. Another NMA focusing on populations with type 2 diabetes included nine outcomes covering glycemic control, cardiovascular risk, and weight loss targets [33]. However, that NMA included only 10 trials and compared 5 diets (low-fat diet, Mediterranean diet, low carbohydrate diet, high carbohydrate diet, and regular diet), and found that the Mediterranean diet was linked with the best outcomes. The top overall ranking of the Paleo diet in our NMA was a result of high SUCRA values for all categories, especially inflammatory biomarkers. The effects of the Paleo diet on individual inflammatory biomarkers did not reach statistical significance in the main analysis, which is similar to a previous NMA focused on weight loss that included CRP [30]. Although our sensitivity analysis excluding studies with high risk of bias, found a significant reduction for hsCRP but not for IL-6. In principle, the Paleo diet attempts to emulate the dietary intake of human ancestors from the Palaeolithic age, focusing on greater intake of fruit, vegetables, lean meat, and fish, while eliminating processed foods, grains, beans, legumes, and dairy. Although some components of the Paleo diet are in line with modern dietary guidelines, others are not and may be detrimental to some long-term health outcomes and environmental sustainability goals [63]. High meat intake is consistent with an increased risk in cardiovascular events, eliminating dairy may have implications for bone health [62], and limiting intakes of grains, beans and legumes can result in dietary carbohydrate restriction [64,65]. In addition, although dietary energy content was outside the scope of this current review, and RCTs that explicitly involved energy restriction were excluded in our study selection, we acknowledge that inadvertent weight reduction may have occurred due to energy intake differences between dietary patterns, with implications for NCD biomarker levels. However, two previous NMAs with a focus on macronutrient composition and weight loss in overweight or obese adults concluded that individual named diets are similarly effective, irrespective of macronutrient content [30,31]. The net effect of the Paleo diet on health and NCD outcomes remains largely inconclusive. Large scale RCTs are warranted to confirm whether the potential benefits of the Paleo diet on NCD biomarkers identified in our research translates to prevention of clinical NCD outcomes.

The DASH diet and Mediterranean diet were the next highest SUCRA ranked dietary patterns in our NMA. The DASH diet is specifically designed to lower blood pressure and is linked to lower cardiovascular disease incidence [66]. Detailing effects on blood pressure was outside the scope of our review; however, a previous NMA ranked the DASH diet best for blood pressure lowering, followed by the Paleo diet [35]. Our NMA supports the DASH diet for overall NCD prevention, ranking highly in all categories; although, it ranked second-worst for HDL cholesterol and apoA-1, potentially due to overall fat restriction. This finding for HDL cholesterol is comparable with a previous NMA focusing on weight loss [30]. Only two other NMAs have included HDL cholesterol as an outcome. Although the DASH diet was not included in either of those, one reported a low-fat diet to be associated with lowest HDL cholesterol levels [33], while the other found that a vegetarian diet was worst [32], which is also generally low in fat. There is a strong body of evidence from observational cohorts and large clinical trials indicating the Mediterranean diet improves clinical cardiovascular outcomes [67]. Two previous NMAs focusing on people with type 2 diabetes concluded that the Mediterranean diet was most beneficial for reducing cardiovascular disease risk factors [32,33]. We found similar benefits of the Mediterranean diet in healthy populations and across a broader range of NCD biomarkers, particularly for improving lipid levels.

Although our results support the Paleo diet, DASH diet, and Mediterranean diet for improving overall NCD biomarker profile, our findings may be of relevance for tailoring dietary recommendations to meet the needs of specific groups. For example, people with dyslipidaemia may particularly benefit from a Mediterranean diet for maximal benefits to lipid levels.

Comparison of the macronutrient composition of dietary patterns demonstrated dietary patterns cannot be described purely by their macronutrient composition. The plant-based diet and low-fat diet included in our analyses had similar macronutrient composition, as did the Paleo diet and low carbohydrate high-fat diet. Despite their similarities in macronutrient composition, these dietary patterns had distinctly different outcomes. As such, although macronutrient profile may partially explain diet-disease relationships, other aspects of a dietary pattern, including source of macronutrients (e.g., plant vs. animal), levels of food processing, food structure, micronutrients, phytochemicals, antioxidants, such as phenolic compounds [68], fatty acid profile, fibre content, etc., likely have contributed to the observed effects of dietary patterns and act as a combined matrix to affect health and NCD outcomes. Although accounting for these dietary aspects is beyond the scope of our work, it would be an area of importance for future research.

Limitations of this systematic review include the broad nature of the search terms. The goal of this review was to identify a broad selection of NCD biomarkers, and this was reflected in our search terms which did not specify individual NCD biomarkers, although we supplemented the search with a thorough manual search for relevant publications. We assume a reasonable level of adherence to dietary interventions for all studies in our analysis as this information is rarely reported. Although almost all included RCTs have employed dietary compliance assessment and/or food provision, and the sensitivity analysis excluding trials with extended intervention duration, which itself is often associated with lesser adherence over time, found similar results. There are a number of distinct variations of the Mediterranean diet. We adopted a pragmatic approach and grouped these as a single broad dietary pattern. This approach captures the totality of evidence under the Mediterranean diet umbrella and is consistent with how the Mediterranean diet is applied in practice. Similarly, the roles of different fatty acids within dietary patterns were not analysed. This NMA would have been statistically underpowered for comparing different variations of Mediterranean diet and specific roles of individual fatty acids; however, future trials should look to compare the different types of Mediterranean diet and variations of diets that were defined by their macronutrients (i.e., low-fat diet and low CHO high-fat diet) [69]. NMA are limited by the inherent statistical assumptions and the quality of the evidence used. Confidence in the results was assessed using CINeMA however residual bias e.g., from small sample sizes may still exist. CINeMA ratings should be taken into account when interpreting the results. We used standard models for NMA, which may be associated with increased type I error rate due to multiple testing [70]. We have not adjusted for multiple comparisons given the exploratory nature of this analysis, although this would be appropriate in future targeted work. SUCRA rankings are an important outcome in this study, although this method does not directly reflect the risk of bias within individual studies. Nonetheless, sensitivity analysis excluding studies at high risk of bias were predominantly consistent with the main results. SUCRA rankings also do not account for effect sizes in different studies. We focused on the combined SUCRA-based ranking to provide a concise indication of overall ranking, noting the number of distinct individual pair-wise comparisons given the possible combinations of dietary patterns and NCD biomarkers covered in this review. Combined SUCRAs were derived by averaging SUCRAs for each biomarker in a given category, and then taking the average for each category. However, this approach equally weights the three categories, which may not be adequately robust for NCD risk prediction. There is currently no evidence to support the use of specific weights of individual categories or biomarkers for combined effects calculation.

This systematic review has multiple strengths. The application of NMA allows the simultaneous comparisons of all possible pairs of dietary patterns, utilising both direct and indirect evidence. Furthermore, we used a geometric framework approach as a novel means to study the macronutrient composition of dietary patterns as a possible mechanism driving NCD biomarkers. Additional strengths include the high number of included randomised trials, and the inclusion of 11 biomarker outcomes across various categories and the use of CINeMA confidence of evidence ratings.

## 5. Conclusions

Our NMA showed that the Mediterranean diet was likely the most effective dietary pattern in lipid profile improvement, a low carbohydrate high-fat diet may be beneficial for glycemic control, and the Paleo diet appeared best for reducing inflammation. No dietary pattern performed consistently best across all major NCD biomarkers or categories, although overall the Paleo diet, DASH diet, and Mediterranean diet had the highest ratings. The majority of the confidence of evidence ratings were low, indicating more research is needed including high-quality randomised trials with clinical outcomes to determine whether the Paleo diet indeed has a role in NCD prevention. Furthermore, our findings were predominantly independent of macronutrient composition, highlighting the potential importance and significance of evidence and advice at the dietary pattern-level.

## Figures and Tables

**Figure 2:**
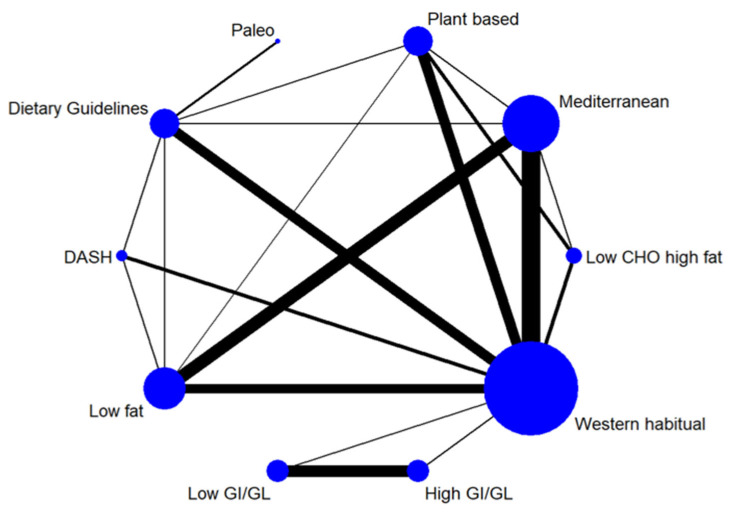
Network diagram for LDL cholesterol illustrating the available direct comparisons between different dietary patterns. The size of the nodes is proportional to the sample size of each dietary pattern intervention, and the thickness of the lines is proportional to the number of studies available. The number of studies for each dietary pattern were: Mediterranean diet (*n* = 20); Dietary Approaches to Stop Hypertension (*n* = 5); Paleo diet (*n* = 2); Dietary guidelines-based diets (*n* = 12); low GI/GL diet (*n* = 9); plant-based diets (*n* = 10); low-fat diet (*n* = 14); low carbohydrate high-fat diet (*n* = 4); high GI/GL diet (*n* = 9); and western habitual diet (*n* = 37).

**Table 1:** League table for LDL cholesterol showing comparative effect sizes between dietary patterns ^1^.

Mediterranean									
−0.03 (−0.27, 0.21) ^4^	DASH								
−0.25 (−0.71, 0.22) ^4^	−0.21 (−0.70, 0.28) ^4^	Paleo							
−0.11 (−0.30, 0.07) ^3^	−0.08 (−0.32, 0.16) ^3^	0.13 (−0.30, 0.56) ^4^	DG-based						
−0.23 (−0.85, 0.39) ^5^	−0.20 (−0.84, 0.45) ^5^	0.02 (−0.74, 0.78) ^4^	−0.12 (−0.74, 0.51) ^5^	Low GI/GL					
−0.01 (−0.20, 0.19) ^3^	0.02 (−0.24, 0.28) ^5^	0.24 (−0.24, 0.71) ^4^	0.10 (−0.10, 0.31) ^2^	0.22 (−0.41, 0.85) ^5^	Plant-based				
−0.03 (−0.18, 0.11) ^3^	0.00 (−0.24, 0.24) ^3^	0.21 (−0.26, 0.69) ^4^	0.08 (−0.12, 0.28) ^4^	0.20 (−0.43, 0.82) ^5^	−0.02 (−0.22, 0.18) ^4^	Low-fat			
−0.37 (−0.68, −0.05) ^3^	−0.33 (−0.70, 0.03) ^4^	−0.12 (−0.66, 0.42) ^4^	−0.25 (−0.59, 0.08) ^4^	−0.14 (−0.82, 0.54) ^5^	−0.36 (−0.66, −0.05) ^2^	−0.33 (−0.66, −0.01) ^4^	Low CHO high-fat		
−0.27 (−0.88, 0.34) ^5^	−0.24 (−0.87, 0.39) ^5^	−0.03 (−0.78, 0.73) ^4^	−0.16 (−0.78, 0.46) ^5^	−0.04 (−0.25, 0.16) ^5^	−0.26 (−0.88, 0.36) ^5^	−0.24 (−0.86, 0.38) ^5^	0.09 (−0.58, 0.76) ^5^	High GI/GL	
−0.29 (−0.41, −0.16) ^3^	−0.25 (−0.47, −0.04) ^3^	−0.04 (−0.50, 0.42) ^4^	−0.17 (−0.33, −0.02) ^3^	−0.06 (−0.67, 0.55) ^5^	−0.28 (−0.44, −0.12) ^3^	−0.26 (−0.41, −0.11) ^3^	0.08 (−0.22, 0.38) ^3^	−0.01 (−0.61, 0.58) ^5^	Western habitual

^1^ The values correspond to the mean difference (95% CI) in LDL cholesterol (mmol/L) between the column dietary pattern and the row dietary pattern; column minus row. ^2^ CINeMA (Confidence in Network Meta-Analysis) ratings were high. ^3^ CINeMA ratings were moderate. ^4^ CINeMA ratings were low. ^5^ CINeMA ratings were very low. Abbreviations: CHO, carbohydrate; DASH, Dietary Approaches to Stop Hypertension; DG, dietary guidelines; GI/GL, glycemic index/glycemic load.

**Table 2:** SUCRA-values for each biomarker, category and for all outcomes combined ^1^.

	Inflammation			Glycemic Control				Lipids and Apolipoproteins							All-Outcomes-Combined ^3^
	hsCRP	IL-6	Combined ^2^	Glucose	Insulin	HOMA-IR	Combined ^2^	TC	LDL-c	HDL-c	TG	ApoB	ApoA1	Combined ^2^	
Mediterranean	56.4	59.7	58.1	50.0	63.1	40.4	51.2	65.3	79.2	57.9	72.0	62.8	41.3	63.1	57.4
DASH	71.3		71.3	61.0	58.2		59.6	75.2	70.3	18.1	63.0	77.9	33.9	56.4	62.4
Paleo	79.0	95.0	87.0	35.3	70.7	89.7	65.2	32.9	35.5	78.6	51.1			49.5	67.2
Dietary guidelines-based	73.4	31.0	52.2	16.7	59.3	58.7	44.9	54.2	52.3	37.4	44.7	64.3	37.9	48.5	48.5
Low GI/GL				48.2	31.1	31.3	36.9	51.1	42.2	43.2	29.8			41.6	
Plant-based	44.9		44.9	57.6	27.6	90.4	58.5	76.0	75.6	16.9	27.5	49.6	21.1	44.5	49.3
Low-fat	14.5	20.4	17.5	48.2	78.5	35.6	54.1	74.3	70.7	28.8	47.6	77.0	45.1	57.3	42.9
Low CHO high-fat	31.2		31.2	80.8	51.9		66.4	14.8	16.8	92.6	91.3	4.3	93.4	52.2	49.9
Traditional Mexican				58.8			58.8								
High GI/GL				66.3	25.8	35.3	42.5	34.2	34.3	55.2	18.4			35.5	
Western habitual	29.3	43.9	36.6	27.2	33.8	18.6	26.5	22.1	23.1	71.5	54.6	14.1	77.2	43.8	35.6

^1^ SUCRA is a value ranges from 0 to 100% that associated with each treatment in a connected network. A higher SUCRA indicates a greater chance of the associated treatment being the best for achieving a favourable outcome. ^2^ Category-level combined SUCRA is the average of biomarkers in the category. ^3^ All-outcomes-combined SUCRA is the average of category-level combined SUCRAs. Abbreviations: CHO, carbohydrate; DASH, Dietary Approaches to Stop Hypertension; GI/GL glycemic index/glycemic load; HDL-c, high-density lipoprotein cholesterol; HOMA-IR, homeostatic model assessment for insulin resistance; hsCRP, high sensitivity C-reactive protein; IL-6, interleukin-6; LDL-c, low-density lipoprotein cholesterol; SUCRA, surface under the cumulative ranking curve; TC, total cholesterol; TG, triglycerides.

## Data Availability

Not applicable.

## Supplementary Materials

The following supporting information can be downloaded at: https://www.mdpi.com/article/10.3390/nu15010076/s1, Table S1: PRISMA NMA checklist; Document S1: Search strategy; Figure S1: Network diagrams for lipids illustrating the available direct comparisons between dietary patterns; Figure S2: Network diagrams for glycemic control biomarkers illustrating the available direct comparisons between dietary patterns; Figure S3: Network diagrams for inflammatory biomarkers illustrating the available direct comparisons between dietary patterns; Table S2: Characteristics of studies included for quantitative syntheses; Table S3: Study-outcomes excluded from quantitative synthesis; Table S4: Risk of bias for individual studies; Table S5: League table for HDL-c showing comparative effect sizes between dietary patterns; Table S6: League table for total cholesterol showing comparative effect sizes between dietary patterns; Table S7: League table for triglycerides showing comparative effect sizes between dietary patterns; Table S8: League table for ApoB showing comparative effect sizes between dietary patterns; Table S9: League table for ApoA1 showing comparative effect sizes between dietary patterns; Table S10: League table for glucose showing comparative effect sizes between dietary patterns; Table S11: League table for insulin showing comparative effect sizes between dietary patterns; Table S12: League table for HOMA-IR showing comparative effect sizes between dietary patterns; Table S13: League table for hsCRP showing comparative effect sizes between dietary patterns; Table S14: League table for interleukin-6 showing comparative effect sizes between dietary patterns; Figure S4: Box plot showing the distribution of participants’ age across the direct comparisons for LDL-c; Figure S5: Box plot showing the distribution of participants’ age across the direct comparisons for ApoB; Figure S6: Box plot showing the distribution of participants’ age across the direct comparisons for glucose; Figure S7: Box plot showing the distribution of participants’ age across the direct comparisons for hsCRP; Figure S8: Box plot showing the distribution of participants’ age across the direct comparisons for interleukin-6; Figure S9: Box plot showing the distribution of percentage of female participants across the direct comparisons for LDL-c; Figure S10: Box plot showing the distribution of percentage of female participants across the direct comparisons for glucose; Figure S11: Box plot showing the distribution of percentage of female participants across the direct comparisons for hsCRP; Figure S12: Box plot showing the distribution of intervention duration across the direct comparisons for LDL-c; Figure S13: Box plot showing the distribution of intervention duration across the direct comparisons for glucose; Figure S14: Box plot showing the distribution of intervention duration across the direct comparisons for hsCRP; Figure S15: Rankograms for LDL-c; Figure S16: Rankograms for HDL-c; Figure S17: Rankograms for total cholesterol; Figure S18: Rankograms for triglycerides; Figure S19: Rankograms for ApoB; Figure S20: Rankograms for ApoA1; Figure S21: Rankograms for glucose; Figure S22: Rankograms for insulin; Figure S23: Rankograms for HOMA-IR; Figure S24: Rankograms for hsCRP; Figure S25: Rankograms for interleukin-6; Table S15: Loop-specific approach assessing inconsistency for LDL-c; Table S16: Loop-specific approach assessing inconsistency for HDL-c; Table S17: Loop-specific approach assessing inconsistency for total cholesterol; Table S18: Loop-specific approach assessing inconsistency for triglycerides; Table S19: Loop-specific approach assessing inconsistency for ApoB; Table S20: Loop-specific approach assessing inconsistency for ApoA1; Table S21: Loop-specific approach assessing inconsistency for glucose; Table S22: Loop-specific approach assessing inconsistency for insulin; Table S23: Loop-specific approach assessing inconsistency for HOMA-IR; Table S24: Loop-specific approach assessing inconsistency for hsCRP; Table S25: Side-splitting approach assessing inconsistency for LDL-c; Table S26: Side-splitting approach assessing inconsistency for HDL-c; Table S27: Side-splitting approach assessing inconsistency for total cholesterol; Table S28: Side-splitting approach assessing inconsistency for triglycerides; Table S29: Side-splitting approach assessing inconsistency for ApoB; Table S30: Side-splitting approach assessing inconsistency for ApoA1; Table S31: Side-splitting approach assessing inconsistency for glucose; Table S32: Side-splitting approach assessing inconsistency for insulin; Table S33: Side-splitting approach assessing inconsistency for HOMA-IR; Table S34: Side-splitting approach assessing inconsistency for hsCRP; Table S35: Side-splitting approach assessing inconsistency for interleukin-6; Table S36: League table of sensitivity analysis excluding high risk of bias studies for LDL-c; Table S37: League table of sensitivity analysis excluding high risk of bias studies for HDL-c; Table S38: League table of sensitivity analysis excluding high risk of bias studies for total cholesterol; Table S39: League table of sensitivity analysis excluding high risk of bias studies for triglycerides; Table S40: League table of sensitivity analysis excluding high risk of bias studies for ApoB; Table S41: League table of sensitivity analysis excluding high risk of bias studies for ApoA1; Table S42: League table of sensitivity analysis excluding high risk of bias studies for glucose; Table S43: League table of sensitivity analysis excluding high risk of bias studies for insulin; Table S44: League table of sensitivity analysis excluding high risk of bias studies for hsCRP; Table S45: League table of sensitivity analysis excluding high risk of bias studies for interleukin-6; Table S46: League table of sensitivity analysis excluding studies with intervention duration longer than 52 weeks for LDL-c; Table S47: League table of sensitivity analysis excluding studies with intervention duration longer than 52 weeks for HDL-c; Table S48: League table of sensitivity analysis excluding studies with intervention duration longer than 52 weeks for total cholesterol; Table S49: League table of sensitivity analysis excluding studies with intervention duration longer than 52 weeks for triglycerides; Table S50: League table of sensitivity analysis excluding studies with intervention duration longer than 52 weeks for glucose; Table S51: League table of sensitivity analysis excluding studies with intervention duration longer than 52 weeks for insulin; Table S52: League table of sensitivity analysis excluding studies with intervention duration longer than 52 weeks for HOMA-IR; Table S53: League table of sensitivity analysis excluding studies with intervention duration longer than 52 weeks for hsCRP; Table S54: League table of sensitivity analysis excluding studies with intervention duration longer than 52 weeks for interleukin-6; Table S55: League table of sensitivity analysis excluding studies with participants’ mean age greater than 70 years for LDL-c; Table S56: League table of sensitivity analysis excluding studies with participants’ mean age greater than 70 years for HDL-c; Table S57: League table of sensitivity analysis excluding studies with participants’ mean age greater than 70 years for total cholesterol; Table S58: League table of sensitivity analysis excluding studies with participants’ mean age greater than 70 years for triglycerides; Table S59: League table of sensitivity analysis excluding studies with participants’ mean age greater than 70 years for glucose; Table S60: League table of sensitivity analysis excluding studies with participants’ mean age greater than 70 years for insulin; Table S61: League table of sensitivity analysis excluding studies with participants’ mean age greater than 70 years for hsCRP; Table S62: League table of sensitivity analysis excluding studies reported hsCRP levels greater than 10 mg/L; Figure S26: Comparison-adjusted funnel plot for LDL-c; Figure S27: Comparison-adjusted funnel plot for HDL-c; Figure S28: Comparison-adjusted funnel plot for total cholesterol; Figure S29: Comparison-adjusted funnel plot for triglycerides; Figure S30: Comparison-adjusted funnel plot for ApoB; Figure S31: Comparison-adjusted funnel plot for ApoA1; Figure S32: Comparison-adjusted funnel plot for glucose; Figure S33: Comparison-adjusted funnel plot for insulin; Figure S34: Comparison-adjusted funnel plot for HOMA-IR; Figure S35: Comparison-adjusted funnel plot for hsCRP; Figure S36: Comparison-adjusted funnel plot for interleukin-6; Table S63: The Confidence in Network Meta-Analysis (CINeMA) framework ratings for credibility for LDL-c; Table S64: The Confidence in Network Meta-Analysis (CINeMA) framework ratings for HDL-c; Table S65: The Confidence in Network Meta-Analysis (CINeMA) framework ratings for total cholesterol; Table S66: The Confidence in Network Meta-Analysis (CINeMA) framework ratings for HOMA-IR; Table S67: The Confidence in Network Meta-Analysis (CINeMA) framework ratings for hsCRP; Figure S37: Right-angle Mixture Triangles (RMTs) for total cholesterol; Figure S38: Right-angle Mixture Triangles (RMTs) for HDL-c; Figure S39: Right-angle Mixture Triangles (RMTs) for ApoA1; Table S68: Coefficients for Right-angle Mixture Triangles.
