# Supplementary material for: Dietary Patterns and Non-Communicable Disease Biomarkers: A Network Meta-Analysis and Nutritional Geometry Approach
Source: Nutrients. 2022 Dec 23;15(1):76. doi: 10.3390/nu15010076 (PMC9824098; doi:10.3390/nu15010076)
Supplement: Supplementary file 1 [file nutrients-15-00076-s001.zip › nutrients-2051126 - suppl final 23.12.2022.pdf]

nutrients

MDPI

Supplementary Materials

Dietary Patterns and Non-Communicable Disease Biomarkers: A Network Meta-Analysis and Nutritional Geometry Approach

Shuang Liang [1], Jovana Mijatovic [1], Ang Li [2], Nicholas Koemel [1], Reeja Nasir [1], Clémence Toniutti [1], Kim Bell-Anderson [3,4], Michael Skilton [1,5,*,†] and Fiona O'Leary [1,3,†]

[1] Faculty of Medicine and Health, The University of Sydney, Sydney, NSW 2006, Australia
[2] Centre for Health Policy, Melbourne School of Population and Global Health, The University of Melbourne, Melbourne, VIC 3010, Australia
[3] The Charles Perkins Centre, The University of Sydney, Sydney, NSW 2006, Australia
[4] Faculty of Science, The University of Sydney, Sydney, NSW 2006, Australia
[5] Sydney Institute for Women, Children and Their Families, Sydney Local Health District, Sydney, NSW 2050, Australia
* Correspondence: michael.skilton@sydney.edu.au; Tel.: +61-2-8627-1916
† These authors contributed equally to this work.

**Content**	**Page**
Supplementary Table S1: PRISMA NMA checklist	2-5
Supplementary Document S1: Search strategy	6-7
Supplementary Figures S1-S3: Network diagrams	8-12
Supplementary Table S2: Characteristics of studies included for quantitative syntheses	13-28
Supplementary Table S3: Study-outcomes excluded from quantitative synthesis	29-31
Supplementary Table S4: Risk of bias for individual studies	32-39
Supplementary Tables S5-S14: League tables	40-49
Supplementary Figures S4-S14: Box plots	50-60
Supplementary Figures S15-S25: Rankograms	61-71
Supplementary Tables S15-S24: Loop-specific approach	72-81
Supplementary Tables S25-S35: Side-splitting approach	82-92
Supplementary Tables S36-S62: Sensitivity analysis for Network Meta-Analysis	93-119
Supplementary Figures S26-S36: Comparison-adjusted funnel plots	120-130
Supplementary Tables S63-S67: The Confidence in Network Meta-Analysis (CINeMA) framework ratings for credibility	131-143
Supplementary Figures S37-S39: Right-angle Mixture Triangles	144-146
Supplementary Table S68: Coefficients for Right-angle Mixture Triangles	147
eReferences	148-151

Supplementary Table S1: PRISMA NMA Checklist of Items to Include When Reporting A Systematic Review Involving a Network Meta-analysis

Section/Topic	Item #	Checklist Item	Reported on Page #
**TITLE**			
Title	1	Identify the report as a systematic review *incorporating a network meta-analysis (or related form of meta-analysis).*	***1***
**ABSTRACT**			
Structured summary	2	Provide a structured summary including, as applicable:<br>**Background:** main objectives<br>**Methods:** data sources; study eligibility criteria, participants, and interventions; study appraisal; and *synthesis methods, such as network meta-analysis.*<br>**Results:** number of studies and participants identified; summary estimates with corresponding confidence/credible intervals; *treatment rankings may also be discussed. Authors may choose to summarize pairwise comparisons against a chosen treatment included in their analyses for brevity.*<br>**Discussion/Conclusions:** limitations; conclusions and implications of findings.<br>**Other:** primary source of funding; systematic review registration number with registry name.	1
**INTRODUCTION**			
Rationale	3	Describe the rationale for the review in the context of what is already known, *including mention of why a network meta-analysis has been conducted.*	***1-2***
Objectives	4	Provide an explicit statement of questions being addressed, with reference to participants, interventions, comparisons, outcomes, and study design (PICOS).	2
**METHODS**			
Protocol and registration	5	Indicate whether a review protocol exists and if and where it can be accessed (e.g., Web address); and, if available, provide registration information, including registration number.	PROSPERO CRD42019129839
Eligibility criteria	6	Specify study characteristics (e.g., PICOS, length of follow-up) and report characteristics (e.g., years considered, language, publication status) used as criteria for eligibility, giving rationale. *Clearly describe eligible treatments included in the treatment network, and note whether any*	***4***

		have been clustered or merged into the same node (with justification).	
Information sources	7	Describe all information sources (e.g., databases with dates of coverage, contact with study authors to identify additional studies) in the search and date last searched.	3
Search	8	Present full electronic search strategy for at least one database, including any limits used, such that it could be repeated.	Supplementary Document S1
Study selection	9	State the process for selecting studies (i.e., screening, eligibility, included in systematic review, and, if applicable, included in the meta-analysis).	4
Data collection process	10	Describe method of data extraction from reports (e.g., piloted forms, independently, in duplicate) and any processes for obtaining and confirming data from investigators.	4
Data items	11	List and define all variables for which data were sought (e.g., PICOS, funding sources) and any assumptions and simplifications made.	4
**Geometry of the network**	**S1**	Describe methods used to explore the geometry of the treatment network under study and potential biases related to it. This should include how the evidence base has been graphically summarized for presentation, and what characteristics were compiled and used to describe the evidence base to readers.	***6-7***
Risk of bias within individual studies	12	Describe methods used for assessing risk of bias of individual studies (including specification of whether this was done at the study or outcome level), and how this information is to be used in any data synthesis.	4-5
Summary measures	13	State the principal summary measures (e.g., risk ratio, difference in means). *Also describe the use of additional summary measures assessed, such as treatment rankings and surface under the cumulative ranking curve (SUCRA) values, as well as modified approaches used to present summary findings from meta-analyses.*	5
Planned methods of analysis	14	Describe the methods of handling data and combining results of studies for each network meta-analysis. This should include, but not be limited to:<br>• *Handling of multi-arm trials;*<br>• *Selection of variance structure;*<br>• *Selection of prior distributions in Bayesian analyses; and*<br>• *Assessment of model fit.*	4-6
**Assessment of Inconsistency**	**S2**	Describe the statistical methods used to evaluate the agreement of direct and indirect evidence in the treatment network(s) studied. Describe efforts taken to address its presence when found.	5

Risk of bias across studies	15	Specify any assessment of risk of bias that may affect the cumulative evidence (e.g., publication bias, selective reporting within studies).	**4-5**
Additional analyses	16	Describe methods of additional analyses if done, indicating which were pre-specified. This may include, but not be limited to, the following:<br>• Sensitivity or subgroup analyses;<br>• Meta-regression analyses;<br>• *Alternative formulations of the treatment network; and*<br>• *Use of alternative prior distributions for Bayesian analyses (if applicable).*	***5-6***
**RESULTS†**			
Study selection	17	Give numbers of studies screened, assessed for eligibility, and included in the review, with reasons for exclusions at each stage, ideally with a flow diagram.	6<br><br>Figure 1
**Presentation of network structure**	**S3**	Provide a network graph of the included studies to enable visualization of the geometry of the treatment network.	***Figure 2***<br><br>***Supplementary Figures S1-S3,***
**Summary of network geometry**	**S4**	Provide a brief overview of characteristics of the treatment network. This may include commentary on the abundance of trials and randomized patients for the different interventions and pairwise comparisons in the network, gaps of evidence in the treatment network, and potential biases reflected by the network structure.	***6-7***
Study characteristics	18	For each study, present characteristics for which data were extracted (e.g., study size, PICOS, follow-up period) and provide the citations.	Supplementary Table S2<br><br>Supplementary Table S3
Risk of bias within studies	19	Present data on risk of bias of each study and, if available, any outcome level assessment.	6<br><br>Supplementary Table S4
Results of individual studies	20	For all outcomes considered (benefits or harms), present, for each study: 1) simple summary data for each intervention group, and 2) effect estimates and confidence intervals. *Modified approaches may be needed to deal with information from larger networks.*	Supplementary Table S3
Synthesis of results	21	Present results of each meta-analysis done, including confidence/credible intervals. *In larger networks, authors may focus on comparisons versus a particular comparator (e.g. placebo or standard care), with full findings presented in an appendix. League tables and forest plots may be considered to summarize pairwise comparisons.* If additional summary measures were explored	***6-7, 10-11***<br><br>***Table 1, Table 2***<br><br>***Supplementary Tables S5-S14***

		(such as treatment rankings), these should also be presented.	
**Exploration for inconsistency**	**S5**	Describe results from investigations of inconsistency. This may include such information as measures of model fit to compare consistency and inconsistency models, *P* values from statistical tests, or summary of inconsistency estimates from different parts of the treatment network.	***11***<br><br>***Supplementary Tables S15-S35***
Risk of bias across studies	22	Present results of any assessment of risk of bias across studies for the evidence base being studied.	11<br>Supplementary Tables S52-S56
Results of additional analyses	23	Give results of additional analyses, if done (e.g., sensitivity or subgroup analyses, meta-regression analyses, *alternative network geometries studied, alternative choice of prior distributions for Bayesian analyses,* and so forth).	***11-12***<br><br>***Supplementary Tables S36-S62 Supplementary Figures S37-S39 Supplementary Table S68***
**DISCUSSION**			
Summary of evidence	24	Summarize the main findings, including the strength of evidence for each main outcome; consider their relevance to key groups (e.g., healthcare providers, users, and policy-makers).	13-14
Limitations	25	Discuss limitations at study and outcome level (e.g., risk of bias), and at review level (e.g., incomplete retrieval of identified research, reporting bias). *Comment on the validity of the assumptions, such as transitivity and consistency. Comment on any concerns regarding network geometry (e.g., avoidance of certain comparisons).*	14-15
Conclusions	26	Provide a general interpretation of the results in the context of other evidence, and implications for future research.	15
**FUNDING**			17
Funding	27	Describe sources of funding for the systematic review and other support (e.g., supply of data); role of funders for the systematic review. This should also include information regarding whether funding has been received from manufacturers of treatments in the network and/or whether some of the authors are content experts with professional conflicts of interest that could affect use of treatments in the network.	

PICOS = population, intervention, comparators, outcomes, study design.
* Text in italics indicates wording specific to reporting of network meta-analyses that has been added to guidance from the PRISMA statement.
† Authors may wish to plan for use of appendices to present all relevant information in full detail for items in this section.

Supplementary Document S1: Search strategy

MEDLINE search

1. Biomarkers/
2. Metabolome/
3. Metabolomics/
4. or/1-3
5. Diet, Carbohydrate Loading/
6. Diet, Atherogenic/
7. exp Diet, Carbohydrate-Restricted/
8. Diet, Fat-Restricted/
9. Diet, Gluten-Free/
10. Diet, High-Fat/
11. exp Diet, High-Protein/
12. Diet, Mediterranean/
13. Diet, Paleolithic/
14. Diet, Protein-Restricted/
15. Diet, Sodium-Restricted/
16. exp Diet, Vegetarian/
17. Diet, Western/
18. Dietary Approaches To Stop Hypertension/
19. Healthy Diet/
20. or/5-19
21. 4 and 20
22. ((Biomarker* or biochemical marker* or biological marker* or metabolomic* or metabolite* or metabolome*) adj5 (nutrition* or food* or diet* or DASH or eating)).tw.
23. 21 or 22
24. Randomized Controlled Trials as Topic/
25. randomized controlled trial/
26. Random Allocation/
27. Double Blind Method/
28. Single Blind Method/
29. clinical trial/
30. clinical trial, phase i.pt.
31. clinical trial, phase ii.pt.
32. clinical trial, phase iii.pt.
33. clinical trial, phase iv.pt.
34. controlled clinical trial.pt.
35. randomized controlled trial.pt.
36. multicenter study.pt.
37. clinical trial.pt.
38. exp Clinical Trials as topic/
39. (clinical adj trial$).tw.
40. ((singl$ or doubl$ or treb$ or tripl$) adj (blind$3 or mask$3)).tw.
41. PLACEBOS/
42. placebo$.tw.
43. randomly allocated.tw.

44. (allocated adj2 random$).tw.
45. or/24-44
46. case report.tw.
47. letter/
48. historical article/
49. or/46-48
50. 45 not 49
51. 23 and 50
52. animal/
53. human/
54. 52 not (52 and 53)
55. 51 not 54
56. limit 55 to english\
57. exp Adult/
58. Adult*.tw.
59. Aged.tw.
60. Elderly.tw.
61. or/57-60
62. 56 and 61

a

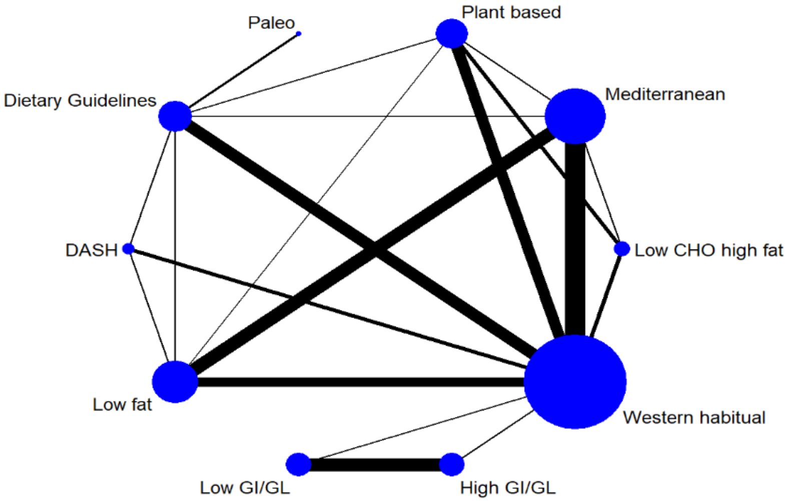
Paleo
Plant based
Dietary Guidelines
Mediterranean
DASH
Low CHO high fat
Low fat
Western habitual
Low GI/GL
High GI/GL

b

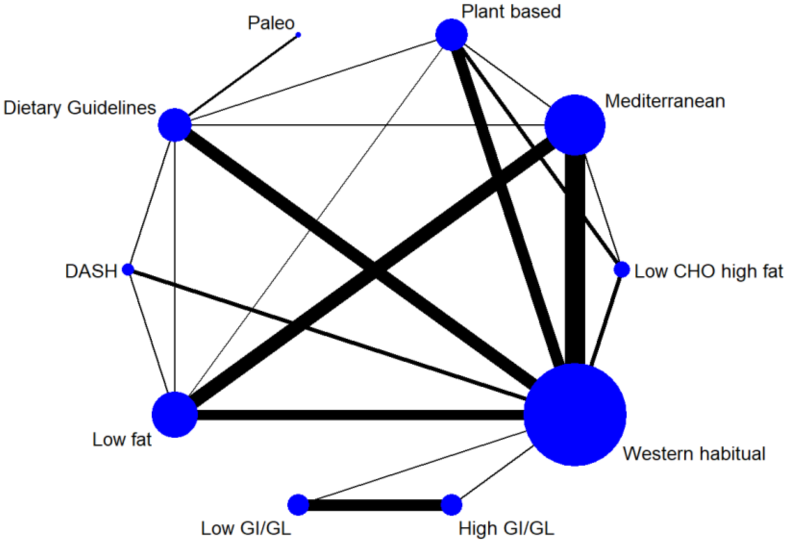
Paleo
Plant based
Dietary Guidelines
Mediterranean
DASH
Low CHO high fat
Low fat
Western habitual
Low GI/GL
High GI/GL

c

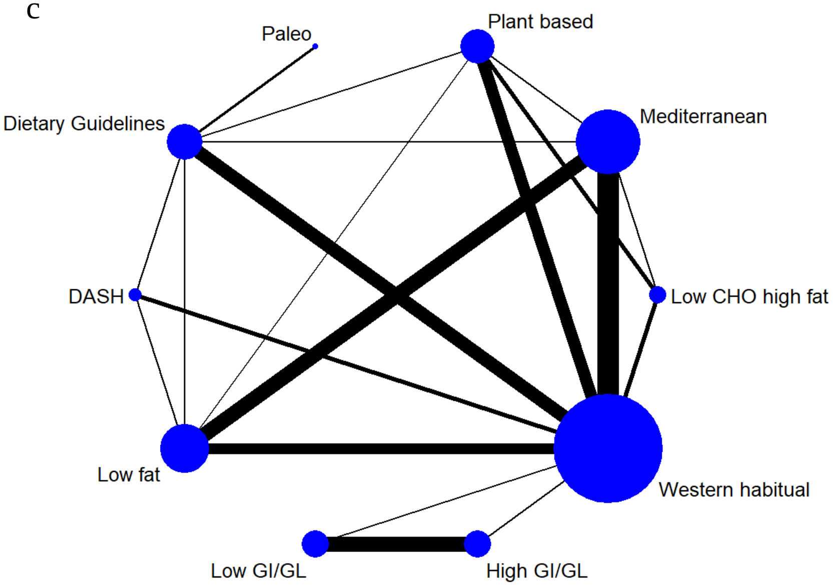
Paleo
Plant based
Dietary Guidelines
Mediterranean
DASH
Low CHO high fat
Low fat
Western habitual
Low GI/GL
High GI/GL

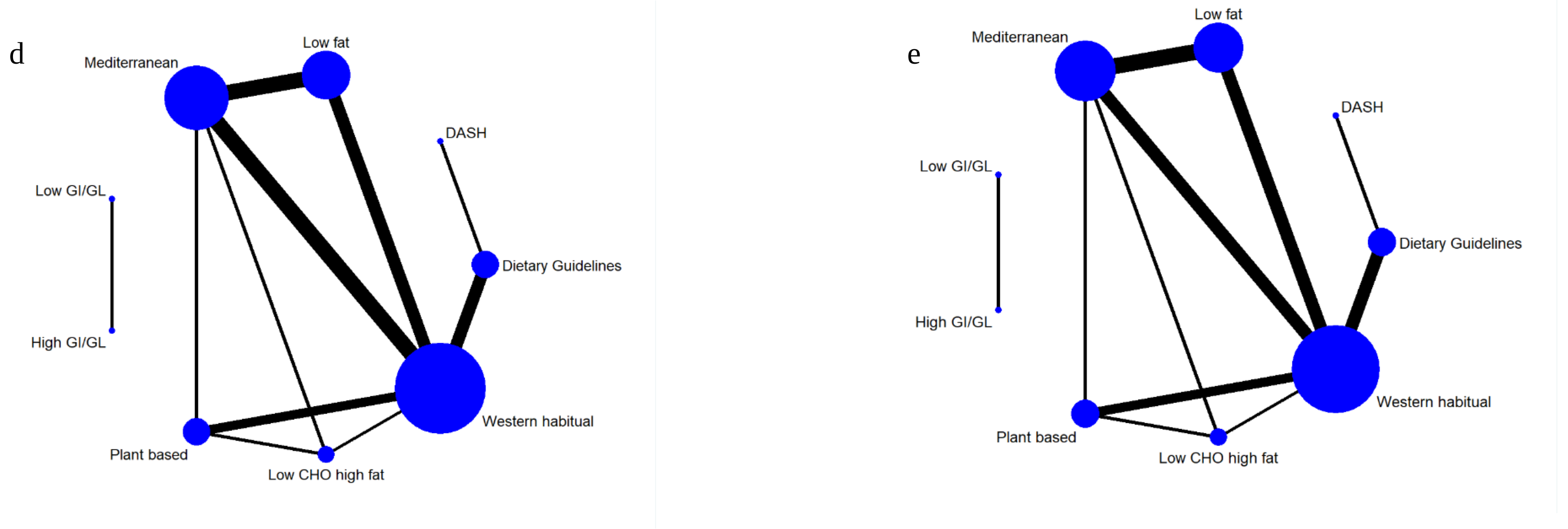

Supplementary Figure S1. Network diagrams for lipids illustrating the available direct comparisons between dietary patterns. The size of the nodes is proportional to the sample size of each dietary pattern intervention, and the thickness of the lines is proportional to the number of studies available. The number of studies for each dietary pattern were: a: High-Density Lipoprotein cholesterol: Mediterranean diet (n = 20); Dietary Approaches to Stop Hypertension (n = 5); Paleo diet (n = 2); Dietary guidelines-based diets (n = 13); low GI / GL diet (n = 10); plant-based diets (n = 10); low fat diet (n = 13); low carbohydrate high fat diet (n = 4); high GI / GL diet (n = 10); and western habitual diet (n = 35). b: Total cholesterol: Mediterranean diet (n = 20); Dietary Approaches to Stop Hypertension (n = 5); Paleo diet (n = 2); Dietary guidelines-based diets (n = 13); low GI / GL diet (n = 8); plant-based diets (n = 10); low fat diet (n = 13); low carbohydrate high fat diet (n = 4); high GI / GL diet (n = 8); and western habitual diet (n = 35). c: Triglycerides: Mediterranean diet (n = 20); Dietary Approaches to Stop Hypertension (n = 5); Paleo diet (n = 2); Dietary guidelines-based diets (n = 13); low GI / GL diet (n = 10); plant-based diets (n = 10); low fat diet (n = 13); low carbohydrate high fat diet (n = 4); high GI / GL diet (n = 10); and western habitual diet (n = 35). d: Apolipoprotein B: Mediterranean diet (n = 7); Dietary Approaches to Stop Hypertension (n = 1); Dietary guidelines-based diets (n = 5); plant-based diets (n = 4); low fat diet (n = 5); low carbohydrate high fat diet (n = 2); and western habitual diet (n = 13). e: Apolipoprotein A1: Mediterranean diet (n = 7); Dietary Approaches to Stop Hypertension (n = 1); Dietary guidelines-based diets (n = 5); plant-based diets (n = 4); low fat diet (n = 5); low carbohydrate high fat diet (n = 2); and western habitual diet (n = 12).

Abbreviations: CHO, carbohydrate; DASH, Dietary Approaches to Stop Hypertension; GI/GL glycemic index/glycemic load

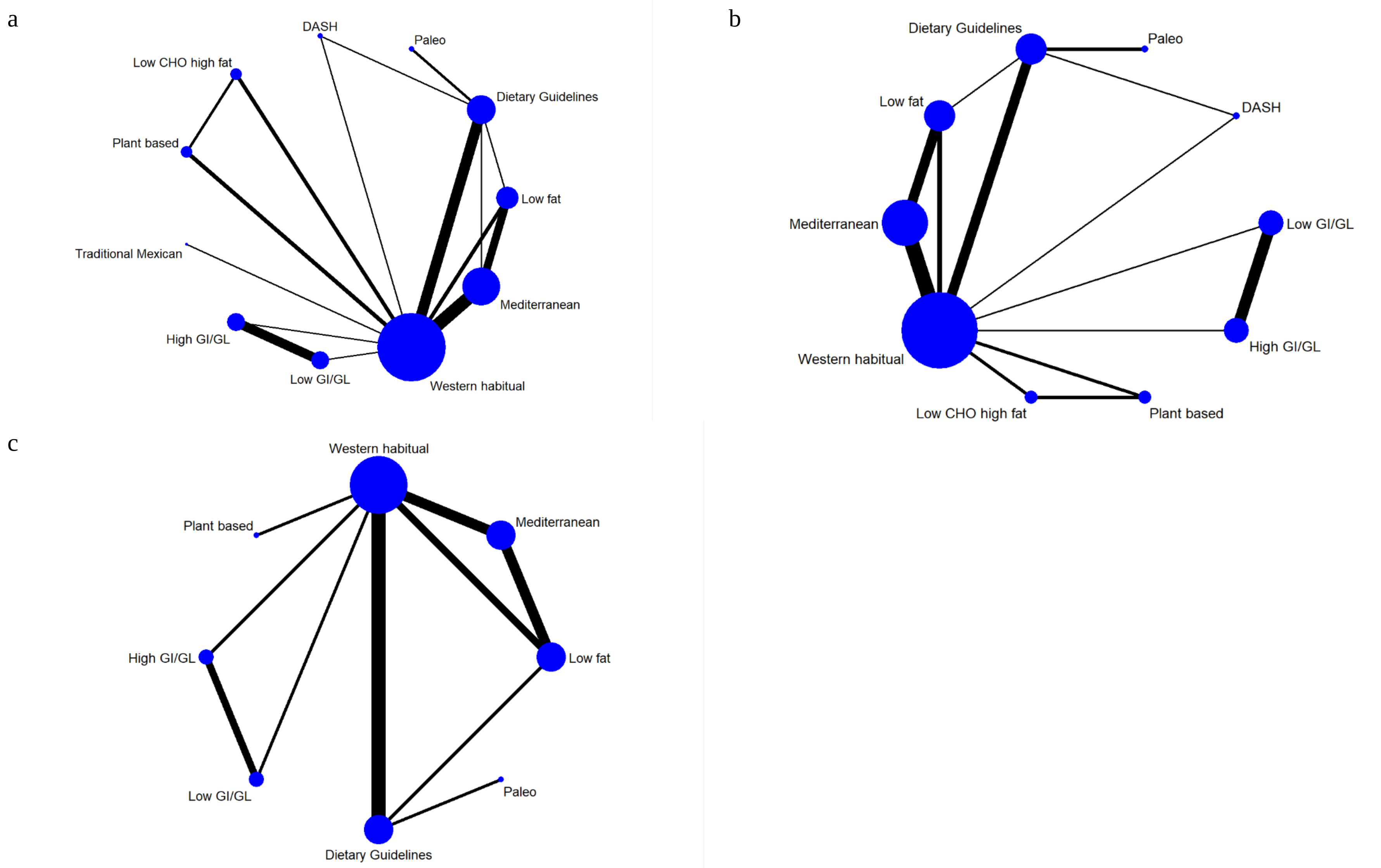
a
DASH
Paleo
Low CHO high fat
Dietary Guidelines
Plant based
Low fat
Traditional Mexican
Mediterranean
High GI/GL
Low GI/GL
Western habitual
b
Dietary Guidelines
Paleo
Low fat
DASH
Mediterranean
Low GI/GL
High GI/GL
Western habitual
Low CHO high fat
Plant based
c
Western habitual
Plant based
Mediterranean
High GI/GL
Low fat
Low GI/GL
Paleo
Dietary Guidelines

Supplementary Figure S2. Network diagrams for glycemic control biomarkers illustrating the available direct comparisons between dietary patterns. The size of the nodes is proportional to the sample size of each dietary pattern intervention, and the thickness of the lines is proportional to the number of studies available. The number of studies for each dietary pattern were: a: Glucose: Mediterranean diet (n = 15); Dietary Approaches to Stop Hypertension (n = 2); Paleo diet (n = 2); Dietary guidelines-based diets (n = 12); low GI / GL diet (n = 7); plant-based diets (n = 3); low fat diet (n = 7); low carbohydrate high fat diet (n = 3); traditional Mexican diet (n = 1); high GI / GL diet (n = 7); and western habitual diet (n = 25). b: Insulin: Mediterranean diet (n = 13); Dietary Approaches to Stop Hypertension (n = 2); Paleo diet (n = 2); Dietary guidelines-based diets (n = 9); low GI / GL diet (n = 7); plant-based diets (n = 2); low fat diet (n = 7); low carbohydrate high fat diet (n = 2); high GI / GL diet (n = 7); and western habitual diet (n = 19). c: Homeostatic model assessment for insulin resistance: Mediterranean diet (n = 5); Paleo diet (n = 1); Dietary guidelines-based diets (n = 5); low GI / GL diet (n = 2); plant-based diets (n = 1); low fat diet (n = 4); high GI / GL diet (n = 2); and western habitual diet (n = 9).

Abbreviations: CHO, carbohydrate; DASH, Dietary Approaches to Stop Hypertension; GI/GL glycemic index/glycemic load

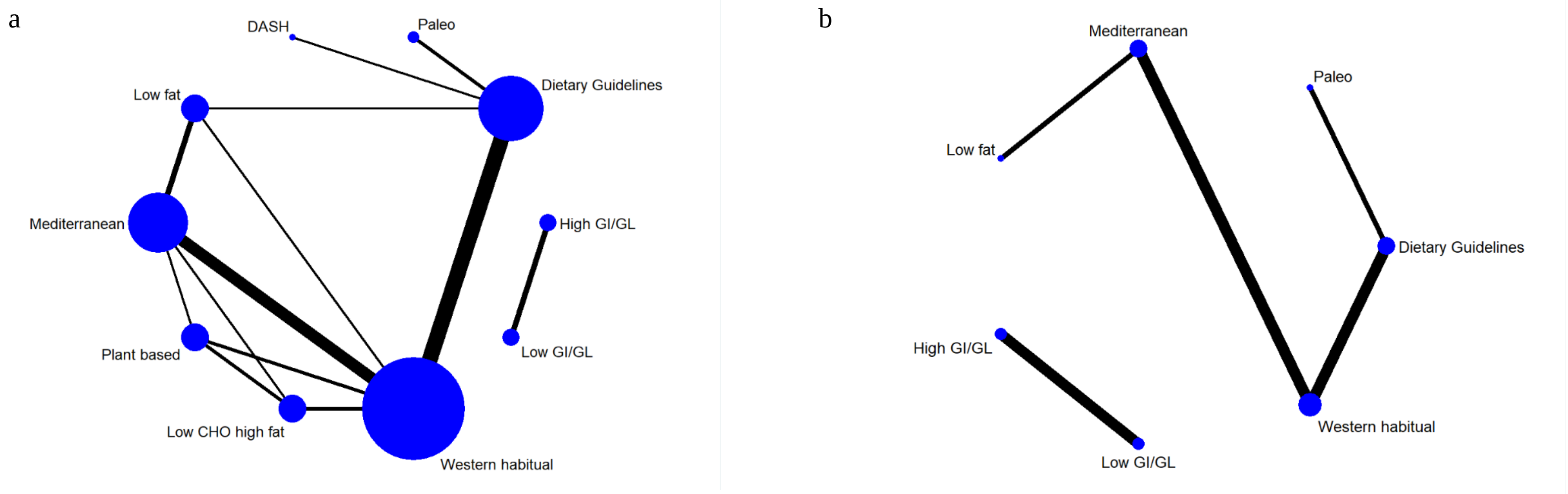

Supplementary Figure S3. Network diagrams for inflammatory biomarkers illustrating the available direct comparisons between dietary patterns. The size of the nodes is proportional to the sample size of each dietary pattern intervention, and the thickness of the lines is proportional to the number of studies available. The number of studies for each dietary pattern were: a: high sensitivity C-Reactive Protein: Mediterranean diet (n = 10); Dietary Approaches to Stop Hypertension (n = 1); Paleo diet (n = 2); Dietary guidelines-based diets (n = 12); plant-based diets (n = 3); low fat diet (n = 4); low carbohydrate high fat diet (n = 3); and western habitual diet (n = 18). b: Interleukin-6: Mediterranean diet (n = 3); Paleo diet (n = 1); Dietary guidelines-based diets (n = 3); low fat diet (n = 1); and western habitual diet (n = 4).

Abbreviations: CHO, carbohydrate; DASH, Dietary Approaches to Stop Hypertension; GI/GL glycemic index/glycemic load

Supplementary Table S2: Characteristics of studies included for quantitative syntheses

Reference	Age (year) mean SD	% Female	Health status	Compliance Assessment	Intervention	Control 1	Control 2	Biomarkers	RoB
Abedi 2010[1] Iran Parallel	51.5 5.2	100	healthy postmeno pausal	FFQ, 24h recall	Education (US DG)	Control	N/A	Glucose TC LDL-c HDL-c TG	Some concerns
Adamsson 2010[2] Sweden Parallel	53 7.9	63	Healthy	Food provided and diet history performed	Healthy Nordic	Western usual	N/A	CRP Glucose Insulin HOMA-IR TC LDL-c HDL-c TG ApoB ApoA1	Some concerns
Ambring 2006[3] Sweden Crossover	43 4.7	45	Healthy	uneaten items were to be returned and weighed at each visit. Furthermore, 3 unannounced telephone interviews (24-h recalls) were performed	MD	Ordinary Swedish	N/A	hsCRP IL-6 Glucose Insulin TC LDL-c HDL-c TG ApoB	Some concerns

Reference	Age (year) mean SD	% Female	Health status	Compliance Assessment	Intervention	Control 1	Control 2	Biomarkers	RoB
Blomquist 2017[4] Sweden Parallel	60.5 6.3	100	overweight/obese postmenopausal	4-day estimated self-reported food record	Paleo	Healthy Nordic (called prudent in paper)	N/A	IL-6	Some concerns
Bos 2010[5] the Netherlands Parallel	55.9 6.7	58	mild abdominal obesity	provided, high compliance	MD	*High MUFA diet otherwise similar to a western diet	Western habitual	Glucose Insulin HOMA-IR TC LDL-c HDL-c TG	Some concerns
Botero 2009[6] USA Crossover	29.4 4.4	0	overweight/obese	Meals were provided and monitered	Low GI	High GI	N/A	CRP TC LDL-c HDL-c TG	Low
Bouché 2002[7] France Crossover	46 3 (sem)	0	Healthy	7d food diary	Low GI	High GI	N/A	Glucose Insulin TC LDL-c HDL-c TG ApoB ApoA1	Some concerns
Bravo-Herrera 2004[8] Spain Crossover	23.4 5.6	48	Healthy	Provided and supervised	MD	Low fat (high CHO)	Western habitual	ApoB ApoA1	High

Reference	Age (year) mean SD	% Female	Health status	Compliance Assessment	Intervention	Control 1	Control 2	Biomarkers	RoB
Brynes 2003[9] UK Crossover	45 8	0	Overweight, at increased CHD risk, otherwise healthy	Some foods provided, returning containers/leftover foods; five 7d diet diaries 100% compliance	Low GI	High GI	High fat	Glucose Insulin HOMA-IR TC LDL-c HDL-c TG	Some concerns
Casas 2014[10] Spain Parallel	67.7 6	53	Increased risk of CVD, 77% T2D	14-item questionnaire MD 9-item LFD	MD	Low fat	N/A	hsCRP	High
Casas 2016[11] Spain Parallel	66.3 5.9	54	At increased CVD risk, 75% T2D	14-item questionnaire MD 9-item LFD	MD	Low fat	N/A	Glucose TC LDL-c HDL-c TG	High
Casas 2017[12] Spain Parallel	66.6 5.9	53	At increased risk of CVD, 64% T2D	14-item questionnaire MD 9-item LFD	MD	Low fat	N/A	IL-6	High
Chen 2010[13] USA DASH-Trial Parallel	44.7 10.7	49	prehypertensive or stage 1 hypertension not on med	Food was provided and supervised, food records used	DASH	High fruit and vegetables diet	Typical US	TC LDL-c HDL-c TG	Some concerns

Reference	Age (year) mean SD	% Female	Health status	Compliance Assessment	Intervention	Control 1	Control 2	Biomarkers	RoB
Clapp 2007[14] USA Crossover	35 8	100	Healthy	Meal was provided and monitored	Low GI	High GI	N/A	Glucose Insulin TC LDL-c HDL-c TG	Some concerns
Cooper 1982[15] USA Crossover	28 NA	33	Healthy	Standard recipes provided, 24h food records kept	Vegetarian	Typical US	N/A	Glucose TC LDL-c HDL-c TG ApoB ApoA1	Some concerns
Dansinger 2005[16] USA Parallel	49 11	51	overweig ht/obese	3-day diet records	Atkins (CHO restricted)	Ornish (fat restricted)	Zone (macros balanced)	CRP Glucose Insulin TC LDL-c HDL-c TG	Low
Davis 2017[17] Australia Parallel	70.9 4.8	56	nonsmoke rs, free of chronic disease	3-day weighed food records at 3 timepoints	MD	Habitual (AU)	N/A	hsCRP Glucose Insulin TC LDL-c HDL-c TG	Some concerns

Reference	Age (year) mean SD	% Female	Health status	Compliance Assessment	Intervention	Control 1	Control 2	Biomarkers	RoB
de Rougemont 2007[18] France Parallel	38.4 9.3	47	overweig ht	dietary records	Low GI	High GI	N/A	Glucose Insulin HOMA-IR TC LDL-c HDL-c TG	Some concerns
Djuric 2009[19] USA Parallel	44 NA	100	Healthy	7d food diary	MD	Control (usual care)	N/A	CRP Glucose Insulin TC LDL-c HDL-c TG	Some concerns
Due 2008[20] Denmark Parallel	28.2 4.8	58	overweig ht/obese at recruitem ent then >=8 % weight loss before randomisa tion	fat biopsy (oleic acid, linoleic acid, myristic acid	Healthy Eating Pyramid high MUFA	USDA food pyramid low fat	Control diet	hsCRP Glucose Insulin HOMA-IR TC LDL-c HDL-c TG	Low
Fernández-Real 2012[21] Spain Parallel	67.9 6.3	0	Increased CVD risk, or diagnosed with T2D	137-item FFQ 14-item questionnaire MD	MD	Low fat	N/A	Insulin	High

Reference	Age (year) mean SD	% Female	Health status	Compliance Assessment	Intervention	Control 1	Control 2	Biomarkers	RoB
Fuentes 2008[22] Spain Crossover	23.3 1.5	0	Healthy	analysing the fatty acids in LDL-cholesteryl esters at the end of each dietary period by the Ruiz-Gutierrez method	MD	Low fat (ALA enriched)	Western	TC LDL-c HDL-c TG	Some concerns
Gardner 2005[23] USA Parallel	48.5 9.1	50	Healthy	Food provided, Dietary daily log	low fat plant-based	Low fat	N/A	TC LDL-c HDL-c TG	Low
Gardner 2007[24] USA Parallel	41 6	100	Healthy	3d, 24h dietary recalls	Atkins (CHO restricted)	Ornish	Zone	Glucose Insulin TC LDL-c HDL-c TG	Low
Genoni 2016[25] Australia Parallel	47 13	100	Healthy	3d WFR daily checklist	Paleo	DG (AGHE)	N/A	CRP Glucose Insulin TC LDL-c HDL-c TG	Low
Hinderliter 2011[26] USA Parallel	52 10	67	overweig ht/obese, higher BP	No information	DASH	Western usual diet	N/A	Glucose Insulin TC LDL-c HDL-c TG	Some concerns

Reference	Age (year) mean SD	% Female	Health status	Compliance Assessment	Intervention	Control 1	Control 2	Biomarkers	RoB
Howard 2006[27] USA Parallel	62.3 6.9	100	healthy postmeno pausal	FFQ	Low fat high fruit, vegetables, whole grains	Usual diet	N/A	LDL-c HDL-c	Some concerns
Hunt 1998[28] USA Crossover	33.2 7.0	100	Healthy	Provided and supervised	Lacto-ovo vegetarian	Non vegetarian (western)	N/A	TC LDL-c HDL-c TG ApoB ApoA1	Some concerns
Hunt 1999[29] USA Crossover	33.2 7.0	100	Healthy	Provided and supervised	Lacto-ovo vegetarian	Non vegetarian (western)	N/A	CRP	Some concerns
Jenkins 1987[30] Canada Crossover	33 4	0	healthy	Provided, diet history recorded	Low GI	High GI	N/A	Glucose Insulin TC LDL-c HDL-c TG	Some concerns
Jensen 2008[31] Denmark Parallel	20-40 (inclus ion criteria)	100	overweig ht	Highly controlled, provided	Low GI	High GI	N/A	Glucose Insulin TC LDL-c HDL-c TG	Some concerns
Kahleova 2018[32] USA Parallel	53.2 12.6	89	overweig ht/obese	Food not provided, 3d dietary record	Low fat vegan	US habitual	N/A	HOMA-IR	Low

Reference	Age (year) mean SD	% Female	Health status	Compliance Assessment	Intervention	Control 1	Control 2	Biomarkers	RoB
Kestin 1989[33] Australia Crossover	44.0 10.0	0	Healthy	Partially provided, recipes, measuring tools provided. 4d food records completed	Lacto-ovo vegetarian	Prudent	AU habitual	TC LDL-c HDL-c TG	Low
Kim 2017[34] Australia Crossover	35.1 15.6	71	Healthy	3d WFR daily checklist (The key foods consumed were 241 g of red meat, 56 g of processed meat (19% protein) and 320 g of refined grains (44% carbohydrate) compared with 70 g of nuts, 236 g of wholegrains and 687 g of dairy foods (19% protein, 37% carbohydrate))	Healthy	Unhealthy	N/A	hsCRP IL-6 Glucose Insulin HOMA-IR TC HDL-c TG	Low
Konstantinidou 2010[35] Spain Parallel	44.3 11.1	71	Healthy	Dietitian consultation, Tyrosol and hydroxytyrosol	MD	Habitual (EU)	N/A	CRP Glucose TC LDL-c HDL-c TG	Some concerns

Reference	Age (year) mean SD	% Female	Health status	Compliance Assessment	Intervention	Control 1	Control 2	Biomarkers	RoB
Ling 1992[36] Finland Parallel	43.3 16.7	70	Healthy	Food records	Uncooked vegan	Finland habitual	N/A	TC LDL-c HDL-c TG ApoB ApoA1	Some concerns
Marin 2011[37] Spain Crossover	67.1 4.52	50	Healthy	24-h recall	MD	Low fat (ALA enriched)	Western	TC LDL-c HDL-c TG ApoB ApoA1	Some concerns
Mellberg 2014[38] Sweden Parallel	59.9 5.7	100	overweig ht/obese postmeno pausal	4-day estimated self-reported food record; poor adherence in Paleo group	Paleo	Healthy Nordic	N/A	hsCRP	Low
Meslier 2020[39] Italy Parallel	43 12	52	overweig ht/obese	7d food diary	MD	Habitual (EU)	N/A	hsCRP Glucose Insulin TC LDL-c HDL-c TG	Some concerns
Miller 2005[40] USA Parallel	52 10	56	Healthy	all food was provided	DASH	US	N/A	TC LDL-c HDL-c TG	Some concerns

Reference	Age (year) mean SD	% Female	Health status	Compliance Assessment	Intervention	Control 1	Control 2	Biomarkers	RoB
Miller 2009[41] USA Crossover	30.6 9.6	50	Healthy	Food records Excellent compliance	MD (South Beach)	High (sat) fat low CHO (Atkins)	High CHO low fat (Ornish)	CRP TC LDL-c HDL-c TG ApoB ApoA1	Some concerns
Mishra 2013[42] USA Parallel	45.2 14.5	83	BMI>=25 and/or T2D	24h diet recall (ASA24)	Vegan	US habitual	N/A	TC LDL-c HDL-c TG	Low
Mohammadshahi 2014[43] Iran Parallel	34.15 5.34	100	Obese	7d food record	HEI (Education)	Habitual	N/A	hsCRP	Low
Morgantini 2018[44] Italy Crossover	25 2.3	57	Healthy	Daily food record	Healthy Western (Low cholesterol, low fat)	Unhealthy Western (High cholesterol, high fat)	N/A	CRP Glucose Insulin TC LDL-c HDL-c TG ApoB ApoA1	Some concerns
Newhouser 2012[45] USA Crossover	29.5 8.1	50	50% overweig ht/obese	Food provision, daily checklist completed	Low GL	High GL	N/A	hsCRP IL-6	Some concerns
Nowson 2005[46] Australia Parallel	47.9 9.3	0	Elevated BP, overweig ht/obese	Dietary counselings, 24h dietary record, FFQ	DASH type	Low fat	N/A	TC LDL-c HDL-c TG	Some concerns

Reference	Age (year) mean SD	% Female	Health status	Compliance Assessment	Intervention	Control 1	Control 2	Biomarkers	RoB
Paniagua 2007[47] Spain Crossover	62 9.4	64	Insulin resistant	Fatty acids measured at the end of each dietary period by gas chromatography	MD	Low fat (high CHO)	High SFA	Glucose Insulin HOMA-IR TC LDL-c HDL-c TG ApoB100 ApoA1	Some concerns
Pérez-Jiménez 2001[48] Spain Crossover	23.1 1.8	49	Healthy	Provided and supervised, compliance also evaluated using 7d food diaries	MD	Low fat (high CHO)	High SFA	Glucose Insulin TC LDL-c HDL-c TG	High
Perez-Martinez 2006[49] Spain Crossover	NA NA	0	healthy	Provided and supervised	MD	Low fat, high CHO n-3 enriched	Western	TC LDL-c HDL-c TG ApoB ApoA1	Some concerns
Poulsen 2014[50] Denmark Parallel	42.1 13.1	71	overweig ht/obese	3d WFR (nonconsecutive)	Healthy Nordic	Average Danish	N/A	hsCRP Glucose Insulin HOMA-IR TC LDL-c HDL-c TG	Low

Reference	Age (year) mean SD	% Female	Health status	Compliance Assessment	Intervention	Control 1	Control 2	Biomarkers	RoB
Rallidis 2009[51] Greece Parallel	50.4 7.3	48	abdominal obesity, otherwise healthy	weekly phone calls, 3d food diaries, 24h recall, food check list, food partially provided, empty food packages returned	MD	Control (less counselling)	N/A	Glucose Insulin HOMA-IR TC LDL-c HDL-c TG	Some concerns
Rallidis 2017[52] Greece Parallel	50.4 7.3	45	Abdominal obesity	weekly telephone contacts with dietitian session attendance 3-day food diaries 24-h recalls check list of the daily consumed foods returning empty packages of food provided	MD	Control (less counselling, usual care)	N/A	hsCRP IL-6	Some concerns
Reidlinger 2015[53] UK Parallel	52.5 8	60	Healthy	4d food record 24h urine sodium	DG (UK)	Traditional British	N/A	hsCRP Glucose Insulin TC LDL-c HDL-c TG ApoB ApoA1	Low

Reference	Age (year) mean SD	% Female	Health status	Compliance Assessment	Intervention	Control 1	Control 2	Biomarkers	RoB
Retterstøl 2018[54] Norway Parallel	25.4 4.9	83	Healthy	WFR Showed good compliance	Low CHO high Fat	Habitual (Norway)	N/A	CRP Glucose TC LDL-c HDL-c TG ApoB ApoA1	Some concerns
Roussell 2012[55] USA Crossover	50 1.4 (sem)	58	Hypercholesterolemic	daily and weekly compliance questionnaires	DASH	Healthy US	The other two arms are not relevant	hsCRP Glucose Insulin TC LDL-c HDL-c TG ApoB ApoA1	Some concerns
Sacks 2014[56] USA Crossover	53 11	52	Overweight, normal-hypertensive	Provided and supervised, compliance also evaluated using daily food diaries	Low GI	High GI	N/A	LDL-c HDL-c TG	Low
Santiago-Torres 2016[57] USA Crossover	27 8	100	Healthy	daily checkoff forms;leftover weighted	Traditional Mexican	US	N/A	Glucose	Some concerns

Reference	Age (year) mean SD	% Female	Health status	Compliance Assessment	Intervention	Control 1	Control 2	Biomarkers	RoB
Shikany 2009[58]<br>USA<br>Crossover	34.5<br>8.1	0	Over-weight/ obese	all food provided, questioned about adherence at each meal pick-up	Low GI/GL	High GI/GL	N/A	CRP<br>IL-6<br>Glucose<br>Insulin<br>TC<br>LDL-c<br>HDL-c<br>TG	Low
Singh 2002[59]<br>India<br>Parallel	48.5<br>9.5	10	Increased risk of CAD, 21% T2D	Dietitian consultations, Food diaries	MD	Prudent	N/A	Glucose<br>TC<br>LDL-c<br>HDL-c<br>TG	Some concerns
Stomby 2015[60]<br>Sweden<br>Parallel	59.9<br>5.7	100	Overweight/obese postmenopausal	Dietitian sessions to promote compliance	Paleo	Healthy Nordic	N/A	Glucose<br>Insulin<br>HOMA-IR<br>TC<br>LDL-c<br>HDL-c<br>TG	Low
Tuomainen 2019[61]<br>Nordic (Finland, Sweden, Denmark, Iceland)<br>Parallel	54.7<br>8.3	66	Overweight/obese with impaired glucose metabolism	4d food record	Healthy Nordic	Habitual (Nordic)	N/A	CRP<br>Glucose<br>TC<br>LDL-c<br>HDL-c<br>TG	Some concerns

Reference	Age (year) mean SD	% Female	Health status	Compliance Assessment	Intervention	Control 1	Control 2	Biomarkers	RoB
Uusitupa 2013[62] Nordic (Finland, Sweden, Denmark, Iceland) Parallel	54.4 8.5	67	features of metabolic syndrome	dietary record	Healthy Nordic	Habitual (Nordic)	N/A	hsCRP IL-6 ApoB ApoA1	High
Vincent-Baudry 2005[63] France Parallel	51.2 10.5	59	overweig ht/obese at increased CVD risk	3d food records, 24h dietary recalls	MD	Low fat	N/A	Glucose Insulin HOMA-IR TC LDL-c HDL-c TG ApoB ApoA1	Some concerns
Vitale 2021[64] Italy Parallel	43.5 12.6	52	Healthy	7d food diary	MD	Habitual (Italy)	N/A	Glucose Insulin TC LDL-c HDL-c TG	Low
Wade 2018[65] Australia Crossover	60.2 6.9	68	At increased CVD risk	yoghurt, nuts, evoo, cheese, chickpeas, beans, lentils, canned tuna and salmon provided; 3d WFR 14 item MD score calculated	MD (Dairy)	Low fat	N/A	CRP Glucose Insulin HOMA-IR TC LDL-c HDL-c TG	Low

Reference	Age (year) mean SD	% Female	Health status	Compliance Assessment	Intervention	Control 1	Control 2	Biomarkers	RoB
Wade 2019[66] Australia Crossover	61 7.1	70	At increased CVD risk	EVOO, pork, nuts, chickpeas, beans and lentils, canned tuna and salmon provided; 3d WFR	MD (Pork)	Low fat	N/A	CRP Glucose Insulin TC LDL-c HDL-c TG	Low
Wardle 2000[67] UK Parallel	48.5 14.1	52	overweig ht with elevated serum cholestero l levels	Education sessions, diatary diaries completed	MD	Low fat	Control (waitlist)	TC LDL-c HDL-c TG	Low
Wright 2017[68] New Zealand Parallel	56.0 9.6	60	overweig ht/obese, 14% T2D, 67% CVD	Dietary indiscretions over 3 days recorded	Low fat plant-based	NZ habitual	N/A	TC LDL-c HDL-c TG	Low

Abbreviations: AGHE, Australian Guide to Healthy Eating; ALA, α-Linolenic acid; ApoA1, apolipoprotein A-I; ApoB, apolipoprotein B; ASA24, automated self-administered recall recall system; AU, Australian; BP, blood pressure; CHD, coronary heart disease; CHO, carbohydrate; CRP, C-reactive protein; CVD, cardiovascular disease; DASH, dietary approaches to stop hypertension; DG, dietary guideline; EU, European; EVOO, extra virgin olive oil; FFQ, food frequency questionnaire; GI, glycemic index; GL, glycemic load; HDL-c, high density lipoprotein cholesterol; HOMA-IR, homeostatic model assessment for insulin resistance; hsCRP, high sensitivity C-reactive protein; IL-6, interleukin-6; LDL-c, low density lipoprotein cholesterol; LFD, low fat diet; MD, Mediterranean diet; MUFA, monounsaturated fatty acid; NZ, New Zealand; RoB, risk of bias; T2D, type 2 diabetes; TC, total cholesterol; TG, triglycerides; UK, United Kingdom; US, United States; WFR, weighed food record

Supplementary Table S3. Study-outcomes excluded from quantitative synthesis: characteristics and end-of-intervention biomarker concentrations

Reference	Age (year) mean SD	% Female	Health status	Compliance Assessment	Intervention	Control 1	Control 2	Biomarkers	RoB
Howard 2006[27] USA Parallel	62.3 6.9	100	Healthy postmenopausal	FFQ	Low fat high fruit, vegetables, whole grains	Usual diet	N/A	*geometric mean<br><br>Low fat<br>TG: 1.61 ± 0.76 mmol/L<br><br>Western habitual<br>TG: 1.63 ± 0.72 mmol/L	Some concerns
Juraschek 2020[69] USA Parallel	45.2 0.6	48.2	Healthy	Meals provided and supervised 95% person-days onsite meals 93% offsite adherence	DASH	High fruit and vegetable diet	Typical US	*geometric mean<br><br>DASH<br>hsCRP: 1.7 ± 0.2 mg/L<br><br>High Fruit & Vegetable<br>hsCRP: 1.5 ± 0.2 mg/L<br><br>Western habitual<br>hsCRP: 1.2 ± 0.1 mg/L	Some concerns

Reference	Age (year) mean SD	% Female	Health status	Compliance Assessment	Intervention	Control 1	Control 2	Biomarkers	RoB
Marckmann 1994[70] Denmark Crossover	52.5 5	52	Healthy	Meals provided and supervised	Healthy Nordic	Average Danish	N/A	*median, n<25<br><br>Dietary guidelines-based<br>TG: 1.11 (0.74, 1.23) mmol/L<br>TC: 4.71 (3.82, 5.2) mmol/L<br>LDL-c: 2.77 (2.44, 3.35) mmol/ L<br>HDL-c: 1.08 (0.99, 1.36) mmol/ L<br><br>Western habitual<br>TG: 0.86 (0.76, 1.23)<br>TC: 5.13 (4.31, 5.44) mmol/L<br>LDL-c: 3.04 (2.57, 3.59) mmol/ L<br>HDL-c: 1.24 (1.1, 1.53) mmol/ L	Low
Runchey 2012[71] USA Crossover	29.6 8.2	50	64% overweight / obese	Meals were provided and supervised; unconsumed food returned; daily consumption recorded	Low GL	High GL	N/A	*geometric mean<br><br>Low GI/GL<br>Glucose: 5 (4.94, 5.06) mmol/L<br>Insulin: 8.2 (7.5, 9) mU/L<br>HOMA-IR: 1.82 (1.66, 2.01)<br><br>High GI/GL<br>Glucose: 4.92 (4.86, 4.98) mmol/L<br>Insulin: 8.3 (7.6, 9.1) mU/L<br>HOMA-IR: 1.82 (1.66, 2.01)	Some concerns

Reference	Age (year) mean SD	% Female	Health status	Compliance Assessment	Intervention	Control 1	Control 2	Biomarkers	RoB
Santiago-Torres 2016[57] USA Crossover	27<br>8	100	Healthy	Daily checkoff forms; leftover weighted	Traditional Mexican	US	N/A	*geometric mean<br><br>Traditional Mexican<br>CRP: 0.93 (0.68, 1.25) mg/L<br>IL-6: 1.32 (1.13, 1.54) pg/mL<br>Insulin: 8.03 ± 3.07 mU/L<br>HOMA-IR: 1.75 (1.56, 1.96)<br><br>Western habitual<br>CRP: 0.94 (0.7, 1.28) mg/L<br>IL-6: 1.36 (1.16, 1.58) pg/mL<br>Insulin: 9.29 ± 3.47 mU/L<br>HOMA-IR: 2.05 (1.82, 2.3)	Some concerns
Tinker 2008[72] USA Parallel	62.2<br>6.9	100	Healthy postmenopausal	FFQ	Low fat high fruit, vegetables, whole grains	Usual diet	N/A	*geometric mean<br><br>Low fat<br>Glucose: 5.13 ± 0.6 mmol/L<br>Insulin: 8.9 ± 4.3 uU/mL<br>HOMA-IR: 2 ± 1.1<br><br>Western habitual<br>Glucose: 5.23 ± 0.74 mmol/L<br>Insulin: 9.6 ± 4.9 uU/mL<br>HOMA-IR: 2.2 ± 1.3	Some concerns

Abbreviations: CRP, C-reactive protein; DASH, dietary approaches to stop hypertension; FFQ, food frequency questionnaire; GI, glycemic index; GL, glycemic load; HDL-c, high density lipoprotein cholesterol; HOMA-IR, homeostatic model assessment for insulin resistance; hsCRP, high sensitivity C-reactive protein; IL-6, interleukin-6; LDL-c, low density lipoprotein cholesterol; RoB, risk of bias; TC, total cholesterol; TG, triglycerides; US, United States

Supplementary Table S4. Risk of bias (RoB) for individual studies

Author, Year<br>Country<br>Design	Randomisation process	Deviations from the intended interventions	Missing outcome data	Measurement of the outcome	Selection of the reported result	Overall RoB
Abedi 2010[1]<br>Iran<br>Parallel	Some concerns	Low	Low	Low	Low	Some concerns
Adamsson 2010[2]<br>Sweden<br>Parallel	Some concerns	Low	Low	Low	Low	Some concerns
Ambring 2006[3]<br>Sweden<br>Crossover	Some concerns	Low	Low	Low	Low	Some concerns
Blomquist 2017[4]<br>Sweden<br>Parallel	Some concerns	Low	Low	Low	Low	Some concerns
Bos 2010[5]<br>the Netherlands<br>Parallel	Some concerns	Low	Low	Low	Low	Some concerns
Botero 2009[6]<br>USA<br>Crossover	Low	Low	Low	Low	Low	Low
Bouché 2002[7]<br>France<br>Crossover	Some concerns	Low	Low	Low	Low	Some concerns
Bravo-Herrera 2004[8]<br>Spain<br>Crossover	High	Low	Low	Low	Low	High
Brynes 2003[9]<br>UK<br>Crossover	Some concerns	Low	Low	Low	Low	Some concerns

Author, Year<br>Country<br>Design	Randomisation process	Deviations from the intended interventions	Missing outcome data	Measurement of the outcome	Selection of the reported result	Overall RoB
Casas 2014[10]<br>Spain<br>Parallel	High	Low	Low	Low	Low	High
Casas 2016[11]<br>Spain<br>Parallel	High	Low	Low	Low	Low	High
Casas 2017[12]<br>Spain<br>Parallel	High	Low	Low	Low	Low	High
Chen 2010[13]<br>USA<br>DASH-Trial<br>Parallel	Some concerns	Low	Low	Low	Low	Some concerns
Clapp 2007[14]<br>USA<br>Crossover	Some concerns	Low	Low	Low	Low	Some concerns
Cooper 1982[15]<br>USA<br>Crossover	Some concerns	Low	Low	Low	Low	Some concerns
Dansinger 2005[16]<br>USA<br>Parallel	Low	Low	Low	Low	Low	Low
Davis 2017[17]<br>Australia<br>Parallel	Some concerns	Low	Low	Low	Low	Some concerns
de Rougemont 2007[18]<br>France<br>Parallel	Some concerns	Low	Low	Low	Low	Some concerns
Djuric 2009[19]<br>USA<br>Parallel	Some concerns	Low	Low	Low	Low	Some concerns

Author, Year<br>Country<br>Design	Randomisation process	Deviations from the intended interventions	Missing outcome data	Measurement of the outcome	Selection of the reported result	Overall RoB
Due 2008[20]<br>Denmark<br>Parallel	Low	Low	Low	Low	Low	Low
Fernández-Real 2012[21]<br>Spain<br>Parallel	High	Low	Low	Low	Low	High
Fuentes 2008[22]<br>Spain<br>Crossover	Some concerns	Low	Low	Low	Low	Some concerns
Gardner 2005[23]<br>USA<br>Parallel	Low	Low	Low	Low	Low	Low
Gardner 2007[24]<br>USA<br>Parallel	Low	Low	Low	Low	Low	Low
Genoni 2016[25]<br>Australia<br>Parallel	Low	Low	Low	Low	Low	Low
Hinderliter 2011[26]<br>USA<br>Parallel	Some concerns	Low	Low	Low	Low	Some concerns
Howard 2006[27]<br>USA<br>Parallel	Some concerns	Low	Low	Low	Low	Some concerns
Hunt 1998[28]<br>USA<br>Crossover	Some concerns	Low	Low	Low	Low	Some concerns
Hunt 1999[29]<br>USA<br>Crossover	Some concerns	Low	Low	Low	Low	Some concerns

Author, Year<br>Country<br>Design	Randomisation process	Deviations from the intended interventions	Missing outcome data	Measurement of the outcome	Selection of the reported result	Overall RoB
Jenkins 1987[30]<br>Canada<br>Crossover	Some concerns	Low	Low	Low	Low	Some concerns
Jensen 2008[31]<br>Denmark<br>Parallel	Some concerns	Low	Low	Low	Low	Some concerns
Kahleova 2018[32]<br>USA<br>Parallel	Low	Low	Low	Low	Low	Low
Kestin 1989[33]<br>Australia<br>Crossover	Low	Low	Low	Low	Low	Low
Kim 2017[34]<br>Australia<br>Crossover	Low	Low	Low	Low	Low	Low
Konstantinidou 2010[35]<br>Spain<br>Parallel	Some concerns	Low	Low	Low	Low	Some concerns
Ling 1992[36]<br>Finland<br>Parallel	Some concerns	Low	Low	Low	Low	Some concerns
Marin 2011[37]<br>Spain<br>Crossover	Some concerns	Low	Low	Low	Low	Some concerns
Mellberg 2014[38]<br>Sweden<br>Parallel	Low	Low	Low	Low	Low	Low
Meslier 2020[39]<br>Italy<br>Parallel	Some concerns	Low	Low	Low	Low	Some concerns

Author, Year<br>Country<br>Design	Randomisation process	Deviations from the intended interventions	Missing outcome data	Measurement of the outcome	Selection of the reported result	Overall RoB
Miller 2005[40]<br>USA<br>Parallel	Some concerns	Low	Low	Low	Low	Some concerns
Miller 2009[41]<br>USA<br>Crossover	Some concerns	Low	Low	Low	Low	Some concerns
Mishra 2013[42]<br>USA<br>Parallel	Low	Low	Low	Low	Low	Low
Mohammadshahi 2014[43]<br>Iran<br>Parallel	Low	Low	Low	Low	Low	Low
Morgantini 2018[44]<br>Italy<br>Crossover	Some concerns	Low	Low	Low	Low	Some concerns
Newhouser 2012[45]<br>USA<br>Crossover	Some concerns	Low	Low	Low	Low	Some concerns
Nowson 2005[46]<br>Australia<br>Parallel	Some concerns	Low	Low	Low	Low	Some concerns
Paniagua 2007[47]<br>Spain<br>Crossover	Some concerns	Low	Low	Low	Low	Some concerns
Pérez-Jiménez 2001[48]<br>Spain<br>Crossover	High	Low	Low	Low	Low	High

Author, Year<br>Country<br>Design	Randomisation process	Deviations from the intended interventions	Missing outcome data	Measurement of the outcome	Selection of the reported result	Overall RoB
Perez-Martinez 2006[49]<br>Spain<br>Crossover	Some concerns	Low	Low	Low	Low	Some concerns
Poulsen 2014[50]<br>Denmark<br>Parallel	Low	Low	Low	Low	Low	Low
Rallidis 2009[51]<br>Greece<br>Parallel	Some concerns	Low	Low	Low	Low	Some concerns
Rallidis 2017[52]<br>Greece<br>Parallel	Some concerns	Low	Low	Low	Low	Some concerns
Reidlinger 2015[53]<br>UK<br>Parallel	Low	Low	Low	Low	Low	Low
Retterstøl 2018[54]<br>Norway<br>Parallel	Some concerns	Low	Low	Low	Low	Some concerns
Roussell 2012[55]<br>USA<br>Crossover	Some concerns	Low	Low	Low	Low	Some concerns
Sacks 2014[56]<br>USA<br>Crossover	Low	Low	Low	Low	Low	Low
Santiago-Torres 2016[57]<br>USA<br>Crossover	Some concerns	Low	Low	Low	Low	Some concerns
Shikany 2009[58]<br>USA<br>Crossover	Low	Low	Low	Low	Low	Low

Author, Year<br>Country<br>Design	Randomisation process	Deviations from the intended interventions	Missing outcome data	Measurement of the outcome	Selection of the reported result	Overall RoB
Singh 2002[59]<br>India<br>Parallel	Some concerns	Low	Low	Low	Low	Some concerns
Stomby 2015[60]<br>Sweden<br>Parallel	Low	Low	Low	Low	Low	Low
Tuomainen 2019[61]<br>Nordic (Finland, Sweden, Denmark, Iceland)<br>Parallel	Some concerns	Low	Low	Low	Low	Some concerns
Uusitupa 2013[62]<br>Nordic (Finland, Sweden, Denmark, Iceland)<br>Parallel	Some concerns	Some concerns	Some concerns	Low	Low	High
Vincent-Baudry 2005[63]<br>France<br>Parallel	Some concerns	Low	Low	Low	Low	Some concerns
Vitale 2021[64]<br>Italy<br>Parallel	Low	Low	Low	Low	Low	Low
Wade 2018[65]<br>Australia<br>Crossover	Low	Low	Low	Low	Low	Low
Wade 2019[66]<br>Australia<br>Crossover	Low	Low	Low	Low	Low	Low
Wardle 2000[67]<br>UK<br>Parallel	Low	Low	Low	Low	Low	Low

Author, Year Country Design	Randomisation process	Deviations from the intended interventions	Missing outcome data	Measurement of the outcome	Selection of the reported result	Overall RoB
Wright 2017[68] New Zealand Parallel	Low	Low	Low	Low	Low	Low
Studies excluded from quantitative syntheses						
Jurascheck 2020[69] USA Parallel	Some concerns	Low	Low	Low	Low	Some concerns
Marckmann 1994[70] Denmark Crossover	Low	Low	Low	Low	Low	Low
Runchey 2012[71] USA Crossover	Some concerns	Low	Low	Low	Low	Some concerns
Tinker 2008[72] USA Parallel	Some concerns	Low	Low	Low	Low	Some concerns

Supplementary Table S5. League table for HDL-c showing comparative effect sizes between dietary patterns[1]

Mediterranean									
**0.07 (0.00, 0.14)**	*DASH*								
-0.07 (-0.21, 0.08)	-0.14 (-0.29, 0.01)	*Paleo*							
0.04 (-0.01, 0.09)	-0.03 (-0.10, 0.03)	0.11 (-0.03, 0.24)	*DG-based*						
0.02 (-0.23, 0.27)	-0.05 (-0.30, 0.20)	0.09 (-0.19, 0.37)	-0.02 (-0.26, 0.23)	*Low GI/GL*					
**0.07 (0.02, 0.13)**	0.00 (-0.07, 0.07)	0.14 (-0.01, 0.29)	0.03 (-0.03, 0.10)	0.05 (-0.20, 0.30)	*Plant-based*				
**0.05 (0.00, 0.10)**	-0.02 (-0.09, 0.05)	0.12 (-0.03, 0.27)	0.01 (-0.04, 0.07)	0.03 (-0.22, 0.28)	-0.02 (-0.08, 0.04)	*Low fat*			
**-0.12 (-0.22, -0.02)**	**-0.19 (-0.30, -0.08)**	-0.05 (-0.22, 0.12)	**-0.16 (-0.26, -0.05)**	-0.14 (-0.40, 0.12)	**-0.19 (-0.29, -0.09)**	**-0.17 (-0.28, -0.06)**	*Low CHO high fat*		
-0.00 (-0.25, 0.24)	-0.07 (-0.32, 0.18)	0.07 (-0.21, 0.35)	-0.04 (-0.29, 0.21)	-0.02 (-0.08, 0.03)	-0.07 (-0.32, 0.17)	-0.05 (-0.30, 0.19)	0.12 (-0.15, 0.38)	*High GI/GL*	
-0.03 (-0.07, 0.01)	**-0.10 (-0.15, -0.04)**	0.04 (-0.10, 0.19)	**-0.06 (-0.11, -0.02)**	-0.05 (-0.29, 0.20)	**-0.10 (-0.14, -0.05)**	**-0.08 (-0.12, -0.03)**	0.09 (-0.01, 0.19)	-0.02 (-0.27, 0.22)	*Western habitual*

[1]The values correspond to the mean difference (95% CI) in HDL-c (mmol/L) between the column dietary pattern and the row dietary pattern; column minus row. Values in bold are effect sizes that are statistically significant at level of 0.05. Darker shading corresponds to higher confidence in CINeMA (Confidence in Network Meta-Analysis) rating.

Abbreviations: CHO, carbohydrate; DASH, Dietary Approaches to Stop Hypertension; DG, dietary guidelines; GI/GL, glycemic index/glycemic load

Supplementary Table S6. League table for total cholesterol showing comparative effect sizes between dietary patterns[1]

Mediterranean									
0.06 (-0.24, 0.36)	*DASH*								
-0.25 (-0.80, 0.29)	-0.31 (-0.89, 0.26)	*Paleo*							
-0.06 (-0.28, 0.16)	-0.12 (-0.41, 0.17)	0.20 (-0.30, 0.69)	*DG-based*						
-0.13 (-0.94, 0.67)	-0.19 (-1.02, 0.64)	0.12 (-0.83, 1.07)	-0.07 (-0.88, 0.74)	*Low GI/GL*					
0.06 (-0.19, 0.31)	0.00 (-0.33, 0.33)	0.31 (-0.25, 0.88)	0.12 (-0.14, 0.38)	0.19 (-0.62, 1.01)	*Plant-based*				
0.04 (-0.14, 0.23)	-0.02 (-0.32, 0.29)	0.30 (-0.26, 0.85)	0.10 (-0.14, 0.35)	0.17 (-0.64, 0.99)	-0.02 (-0.27, 0.24)	*Low fat*			
**-0.42 (-0.83, -0.01)**	**-0.48 (-0.94, -0.01)**	-0.16 (-0.82, 0.49)	-0.36 (-0.78, 0.07)	-0.29 (-1.17, 0.59)	**-0.48 (-0.87, -0.08)**	**-0.46 (-0.88, -0.04)**	*Low CHO high fat*		
-0.27 (-1.07, 0.54)	-0.33 (-1.16, 0.51)	-0.01 (-0.96, 0.94)	-0.21 (-1.02, 0.60)	-0.13 (-0.44, 0.17)	-0.33 (-1.14, 0.49)	-0.31 (-1.12, 0.50)	0.15 (-0.73, 1.03)	*High GI/GL*	
**-0.30 (-0.46, -0.14)**	**-0.36 (-0.62, -0.10)**	-0.04 (-0.57, 0.49)	**-0.24 (-0.42, -0.06)**	-0.17 (-0.96, 0.62)	**-0.36 (-0.56, -0.15)**	**-0.34 (-0.53, -0.15)**	0.12 (-0.27, 0.51)	-0.03 (-0.82, 0.76)	*Western habitual*

[1]The values correspond to the mean difference (95% CI) in total cholesterol (mmol/L) between the column dietary pattern and the row dietary pattern; column minus row. Values in bold are effect sizes that are statistically significant at level of 0.05. Darker shading corresponds to higher confidence in CINeMA (Confidence in Network Meta-Analysis) rating.

Abbreviations: CHO, carbohydrate; DASH, Dietary Approaches to Stop Hypertension; DG, dietary guidelines; GI/GL, glycemic index/glycemic load

Supplementary Table S7. League table for triglycerides showing comparative effect sizes between dietary patterns[1]

Mediterranean									
-0.01 (-0.15, 0.13)	*DASH*								
-0.04 (-0.24, 0.15)	-0.03 (-0.25, 0.19)	*Paleo*							
-0.05 (-0.15, 0.04)	-0.04 (-0.18, 0.10)	-0.01 (-0.18, 0.16)	*DG-based*						
-0.21 (-0.70, 0.29)	-0.20 (-0.70, 0.31)	-0.17 (-0.69, 0.36)	-0.16 (-0.65, 0.34)	*Low GI/GL*					
-0.09 (-0.20, 0.01)	-0.08 (-0.23, 0.07)	-0.05 (-0.25, 0.15)	-0.04 (-0.15, 0.07)	0.12 (-0.38, 0.62)	*Plant-based*				
-0.05 (-0.13, 0.04)	-0.03 (-0.18, 0.11)	-0.01 (-0.20, 0.19)	0.00 (-0.10, 0.11)	0.16 (-0.33, 0.66)	0.04 (-0.06, 0.15)	*Low fat*			
0.13 (-0.09, 0.34)	0.14 (-0.10, 0.39)	0.17 (-0.11, 0.45)	0.18 (-0.04, 0.40)	0.34 (-0.19, 0.87)	**0.22 (0.01, 0.44)**	0.18 (-0.05, 0.40)	*Low CHO high fat*		
-0.26 (-0.75, 0.24)	-0.25 (-0.75, 0.26)	-0.22 (-0.74, 0.31)	-0.21 (-0.70, 0.29)	-0.05 (-0.15, 0.06)	-0.17 (-0.66, 0.33)	-0.21 (-0.71, 0.29)	-0.39 (-0.92, 0.15)	*High GI/GL*	
-0.03 (-0.10, 0.04)	-0.02 (-0.15, 0.11)	0.01 (-0.18, 0.20)	0.02 (-0.06, 0.10)	0.18 (-0.31, 0.67)	0.06 (-0.03, 0.15)	0.01 (-0.07, 0.10)	-0.16 (-0.37, 0.05)	0.22 (-0.27, 0.71)	*Western habitual*

[1]The values correspond to the mean difference (95% CI) in triglycerides (mmol/L) between the column dietary pattern and the row dietary pattern; column minus row. Values in bold are effect sizes that are statistically significant at level of 0.05.

Abbreviations: CHO, carbohydrate; DASH, Dietary Approaches to Stop Hypertension; DG, dietary guidelines; GI/GL, glycemic index/glycemic load

Supplementary Table S8. League table for ApoB showing comparative effect sizes between dietary patterns[1]

Mediterranean						
0.02 (-0.08, 0.12)	*DASH*					
0.00 (-0.07, 0.07)	-0.02 (-0.09, 0.05)	*DG-based*				
-0.02 (-0.09, 0.05)	-0.04 (-0.14, 0.06)	-0.02 (-0.10, 0.06)	*Plant-based*			
0.01 (-0.03, 0.05)	-0.01 (-0.11, 0.09)	0.01 (-0.06, 0.08)	0.03 (-0.04, 0.10)	*Low fat*		
**-0.15 (-0.28, -0.02)**	**-0.17 (-0.33, -0.01)**	**-0.15 (-0.29, -0.01)**	-0.13 (-0.27, 0.00)	**-0.16 (-0.30, -0.03)**	*Low CHO High fat*	
**-0.09 (-0.13, -0.04)**	**-0.11 (-0.20, -0.02)**	**-0.09 (-0.14, -0.03)**	**-0.07 (-0.12, -0.01)**	**-0.10 (-0.15, -0.05)**	0.06 (-0.07, 0.20)	*Western habitual*

[1]The values correspond to the mean difference (95% CI) in ApoB (g/L) between the column dietary pattern and the row dietary pattern; column minus row. Values in bold are effect sizes that are statistically significant at level of 0.05.

Abbreviations: CHO, carbohydrate; DASH, Dietary Approaches to Stop Hypertension; DG, dietary guidelines

Supplementary Table S9. League table for ApoA1 showing comparative effect sizes between dietary patterns[1]

Mediterranean						
0.03 (-0.20, 0.26)	*DASH*					
0.01 (-0.13, 0.15)	-0.02 (-0.20, 0.16)	*DG-based*				
0.05 (-0.09, 0.19)	0.02 (-0.21, 0.25)	0.04 (-0.11, 0.19)	*Plant-based*			
-0.01 (-0.10, 0.09)	-0.03 (-0.26, 0.19)	-0.01 (-0.16, 0.13)	-0.05 (-0.20, 0.09)	*Low fat*		
-0.17 (-0.36, 0.03)	-0.20 (-0.47, 0.08)	-0.18 (-0.39, 0.03)	**-0.22 (-0.42, -0.02)**	-0.16 (-0.36, 0.04)	*Low CHO high fat*	
-0.07 (-0.16, 0.03)	-0.09 (-0.30, 0.11)	-0.07 (-0.18, 0.03)	-0.11 (-0.23, 0.00)	-0.06 (-0.16, 0.04)	0.10 (-0.08, 0.29)	*Western habitual*

[1]The values correspond to the mean difference (95% CI) in ApoA1 (g/L) between the column dietary pattern and the row dietary pattern; column minus row. Values in bold are effect sizes that are statistically significant at level of 0.05.

Abbreviations: CHO, carbohydrate; DASH, Dietary Approaches to Stop Hypertension; DG, dietary guidelines

Supplementary Table S10. League table for glucose showing comparative effect sizes between dietary patterns[1]

Mediterranean										
0.03 (-0.10, 0.17)	*DASH*									
-0.05 (-0.35, 0.24)	-0.09 (-0.40, 0.22)	*Paleo*								
-0.07 (-0.16, 0.01)	-0.11 (-0.23, 0.01)	-0.02 (-0.31, 0.27)	*DG-based*							
0.02 (-0.41, 0.44)	-0.02 (-0.45, 0.42)	0.07 (-0.44, 0.59)	0.09 (-0.33, 0.52)	*Low GI/GL*						
0.04 (-0.18, 0.26)	0.00 (-0.24, 0.25)	0.09 (-0.27, 0.45)	0.11 (-0.11, 0.33)	0.02 (-0.45, 0.49)	*Plant-based*					
-0.00 (-0.09, 0.08)	-0.04 (-0.18, 0.11)	0.05 (-0.25, 0.35)	0.07 (-0.02, 0.17)	-0.02 (-0.45, 0.41)	-0.04 (-0.27, 0.19)	*Low fat*				
0.13 (-0.09, 0.35)	0.10 (-0.14, 0.34)	0.18 (-0.18, 0.54)	0.20 (-0.01, 0.42)	0.11 (-0.36, 0.58)	0.09 (-0.15, 0.34)	0.13 (-0.09, 0.36)	*Low CHO high fat*			
0.03 (-0.13, 0.20)	-0.00 (-0.19, 0.19)	0.09 (-0.24, 0.42)	0.11 (-0.06, 0.27)	0.02 (-0.43, 0.46)	-0.00 (-0.26, 0.25)	0.04 (-0.14, 0.21)	-0.10 (-0.35, 0.16)	*Mexican*		
0.09 (-0.34, 0.51)	0.05 (-0.38, 0.49)	0.14 (-0.37, 0.66)	0.16 (-0.26, 0.59)	0.07 (-0.03, 0.18)	0.05 (-0.42, 0.52)	0.09 (-0.34, 0.52)	-0.04 (-0.51, 0.43)	0.06 (-0.39, 0.50)	*High GI/GL*	
-0.05 (-0.11, 0.02)	-0.08 (-0.20, 0.04)	0.01 (-0.29, 0.30)	0.03 (-0.04, 0.09)	-0.06 (-0.49, 0.36)	-0.08 (-0.30, 0.13)	-0.04 (-0.13, 0.04)	-0.18 (-0.38, 0.03)	-0.08 (-0.23, 0.07)	-0.14 (-0.56, 0.29)	*Western habitual*

[1]The values correspond to the mean difference (95% CI) in glucose (mmol/L) between the column dietary pattern and the row dietary pattern; column minus row.

Abbreviations: CHO, carbohydrate; DASH, Dietary Approaches to Stop Hypertension; DG, dietary guidelines; GI/GL, glycemic index/glycemic load

Supplementary Table S11. League table for insulin showing comparative effect sizes between dietary patterns[1]

Mediterranean									
-0.27 (-6.02, 5.49)	*DASH*								
0.96 (-5.23, 7.16)	1.23 (-6.08, 8.54)	*Paleo*							
-0.25 (-3.79, 3.30)	0.02 (-5.23, 5.27)	-1.21 (-6.29, 3.87)	*DG-based*						
-3.44 (-11.72, 4.83)	-3.18 (-12.70, 6.35)	-4.41 (-14.23, 5.41)	-3.20 (-11.60, 5.20)	*Low GI/GL*					
-3.48 (-11.06, 4.10)	-3.21 (-12.14, 5.71)	-4.44 (-13.68, 4.79)	-3.23 (-10.95, 4.48)	-0.03 (-10.74, 10.67)	*Plant-based*				
1.15 (-1.58, 3.89)	1.42 (-4.62, 7.45)	0.19 (-6.22, 6.60)	1.40 (-2.51, 5.30)	4.60 (-3.91, 13.10)	4.63 (-3.20, 12.46)	*Low fat*			
-0.86 (-8.53, 6.82)	-0.59 (-9.60, 8.42)	-1.82 (-11.14, 7.50)	-0.61 (-8.42, 7.20)	2.59 (-8.19, 13.36)	2.62 (-4.16, 9.41)	-2.01 (-9.93, 5.92)	*Low CHO High fat*		
-3.87 (-11.96, 4.21)	-3.61 (-12.97, 5.76)	-4.84 (-14.50, 4.82)	-3.63 (-11.84, 4.59)	-0.43 (-3.57, 2.71)	-0.39 (-10.95, 10.16)	-5.02 (-13.35, 3.30)	-3.02 (-13.65, 7.62)	*High GI/GL*	
-1.94 (-4.32, 0.44)	-1.67 (-6.95, 3.61)	-2.90 (-8.69, 2.89)	-1.69 (-4.47, 1.09)	1.51 (-6.42, 9.43)	1.54 (-5.65, 8.73)	**-3.09 (-6.18, -0.00)**	-1.08 (-8.38, 6.22)	1.94 (-5.79, 9.67)	*Western habitual*

[1]The values correspond to the mean difference (95% CI) in insulin (mU/L) between the column dietary pattern and the row dietary pattern; column minus row. Values in bold are effect sizes that are statistically significant at level of 0.05.

Abbreviations: CHO, carbohydrate; DASH, Dietary Approaches to Stop Hypertension; DG, dietary guidelines; GI/GL, glycemic index/glycemic load

Supplementary Table S12. League table for HOMA-IR showing comparative effect sizes between dietary patterns[1]

Mediterranean							
0.76 (-0.03, 1.56)	*Paleo*						
0.16 (-0.23, 0.56)	-0.60 (-1.30, 0.10)	*DG-based*					
-0.14 (-1.23, 0.94)	-0.91 (-2.16, 0.35)	-0.31 (-1.35, 0.74)	*Low GI/GL*				
**0.72 (0.23, 1.21)**	-0.05 (-0.84, 0.75)	**0.55 (0.16, 0.95)**	0.86 (-0.20, 1.92)	*Plant-based*			
-0.03 (-0.33, 0.27)	**-0.80 (-1.56, -0.03)**	-0.20 (-0.52, 0.13)	0.11 (-0.96, 1.19)	**-0.75 (-1.21, -0.29)**	*Low fat*		
-0.10 (-1.23, 1.03)	-0.87 (-2.15, 0.42)	-0.27 (-1.35, 0.82)	0.04 (-0.48, 0.56)	-0.82 (-1.92, 0.28)	-0.07 (-1.19, 1.05)	*High GI/GL*	
-0.18 (-0.56, 0.20)	**-0.95 (-1.68, -0.21)**	**-0.35 (-0.59, -0.10)**	-0.04 (-1.06, 0.98)	**-0.90 (-1.21, -0.59)**	-0.15 (-0.50, 0.20)	-0.08 (-1.14, 0.98)	*Western habitual*

[1]The values correspond to the mean difference (95% CI) in HOMA-IR between the column dietary pattern and the row dietary pattern; column minus row. Values in bold are effect sizes that are statistically significant at level of 0.05. Darker shading corresponds to higher confidence in CINeMA (Confidence in Network Meta-Analysis) rating.

Abbreviations: DG, dietary guidelines; GI/GL, glycemic index/glycemic load; HOMA-IR, homeostatic model assessment for insulin resistance

Supplementary Table S13. League table for hsCRP showing comparative effect sizes between dietary patterns[1]

Mediterranean							
0.34 (-0.86, 1.53)	*DASH*						
0.49 (-0.72, 1.71)	0.16 (-1.24, 1.56)	*Paleo*					
0.27 (-0.41, 0.95)	-0.07 (-1.05, 0.91)	-0.23 (-1.23, 0.77)	*DG-based*				
-0.11 (-0.77, 0.55)	-0.44 (-1.74, 0.85)	-0.60 (-1.92, 0.71)	-0.37 (-1.22, 0.47)	*Plant-based*			
-0.48 (-1.10, 0.14)	-0.82 (-2.06, 0.43)	-0.98 (-2.24, 0.29)	-0.75 (-1.51, 0.02)	-0.37 (-1.25, 0.51)	*Low fat*		
-0.26 (-0.96, 0.44)	-0.60 (-1.92, 0.73)	-0.76 (-2.10, 0.59)	-0.53 (-1.42, 0.36)	-0.15 (-0.90, 0.59)	0.22 (-0.70, 1.14)	*Low CHO high fat*	
-0.24 (-0.66, 0.18)	-0.58 (-1.72, 0.56)	-0.74 (-1.90, 0.43)	-0.51 (-1.09, 0.08)	-0.13 (-0.76, 0.49)	0.24 (-0.45, 0.93)	0.02 (-0.66, 0.69)	*Western habitual*

[1]The values correspond to the mean difference (95% CI) in hsCRP (mg/L) between the column dietary pattern and the row dietary pattern; column minus row. Values in bold are effect sizes that are statistically significant at level of 0.05. Darker shading corresponds to higher confidence in CINeMA (Confidence in Network Meta-Analysis) rating.

Abbreviations: CHO, carbohydrate; DASH, Dietary Approaches to Stop Hypertension; DG, dietary guidelines; hsCRP, high sensitivity C-reactive protein

Supplementary Table S14. League table for interleukin-6 showing comparative effect sizes between dietary patterns[1]

Mediterranean				
1.45 (-0.51, 3.41)	*Paleo*			
-0.19 (-0.71, 0.34)	-1.64 (-3.53, 0.25)	*DG-based*		
-0.35 (-1.00, 0.30)	-1.80 (-3.87, 0.26)	-0.16 (-1.00, 0.67)	*Low fat*	
-0.13 (-0.59, 0.34)	-1.58 (-3.48, 0.32)	0.06 (-0.18, 0.30)	0.22 (-0.57, 1.02)	*Western habitual*

[1]The values correspond to the mean difference (95% CI) in interleukin-6 (pg/mL) between the column dietary pattern and the row dietary pattern; column minus row. Values in bold are effect sizes that are statistically significant at level of 0.05.

Abbreviations: DG, dietary guidelines

Supplementary Figure S4. Box plot showing the distribution of participants' age across the direct comparisons for LDL-c

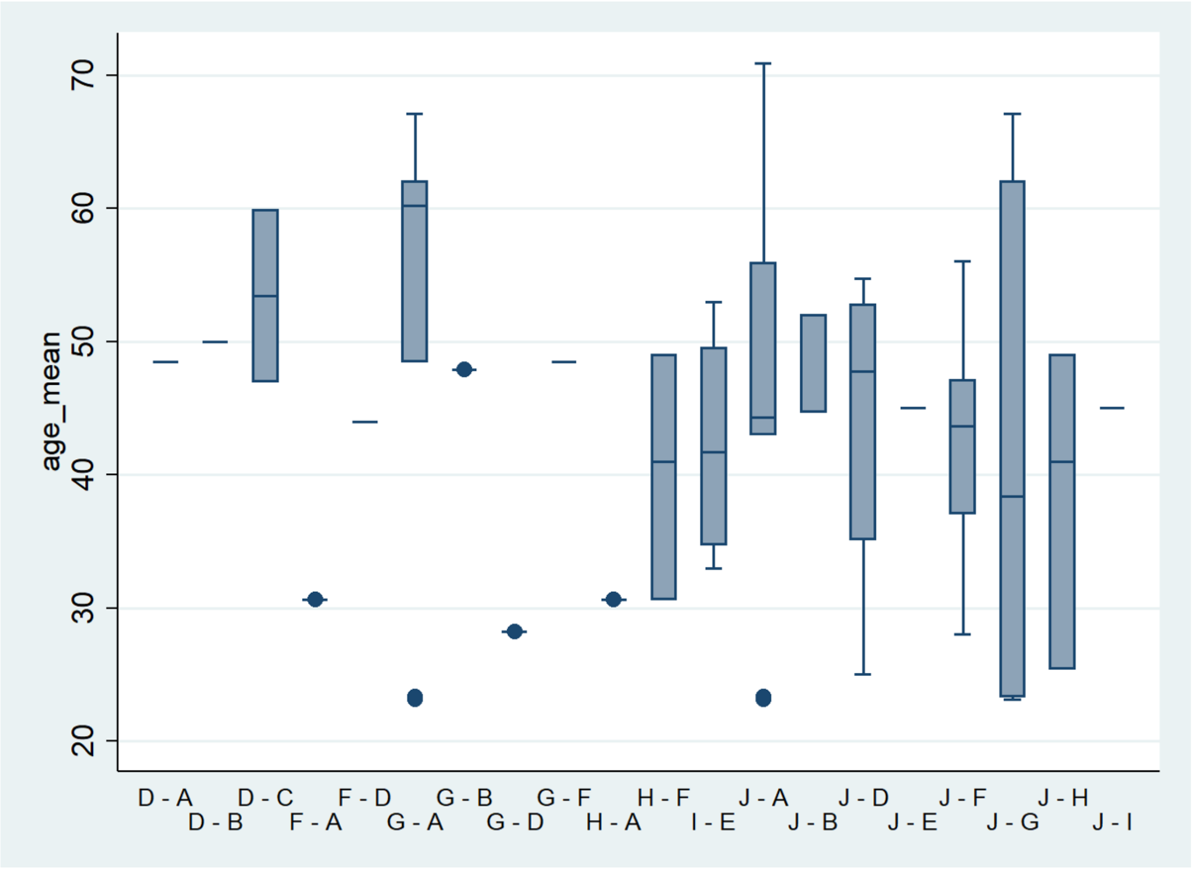

A = Mediterranean diet; **B** = Dietary Approaches to Stop Hypertension; **C** = Paleo diet; **D** = Dietary Guidelines-based diets; **E** = low GI / GL diet; **F** = plant-based diets; **G** = low fat diet; **H** = low carbohydrate high fat diet; **I** = high GI / GL diet; **J** = western habitual diet

The lower and upper edges of the box represent the 25th and 75th percentiles, the middle line in the box marks the 50th percentile (median), the error bars are the 95% confidence intervals, and any dots beyond the error bars are outliers.

Supplementary Figure S5. Box plot showing the distribution of participants' age across the direct comparisons for ApoB

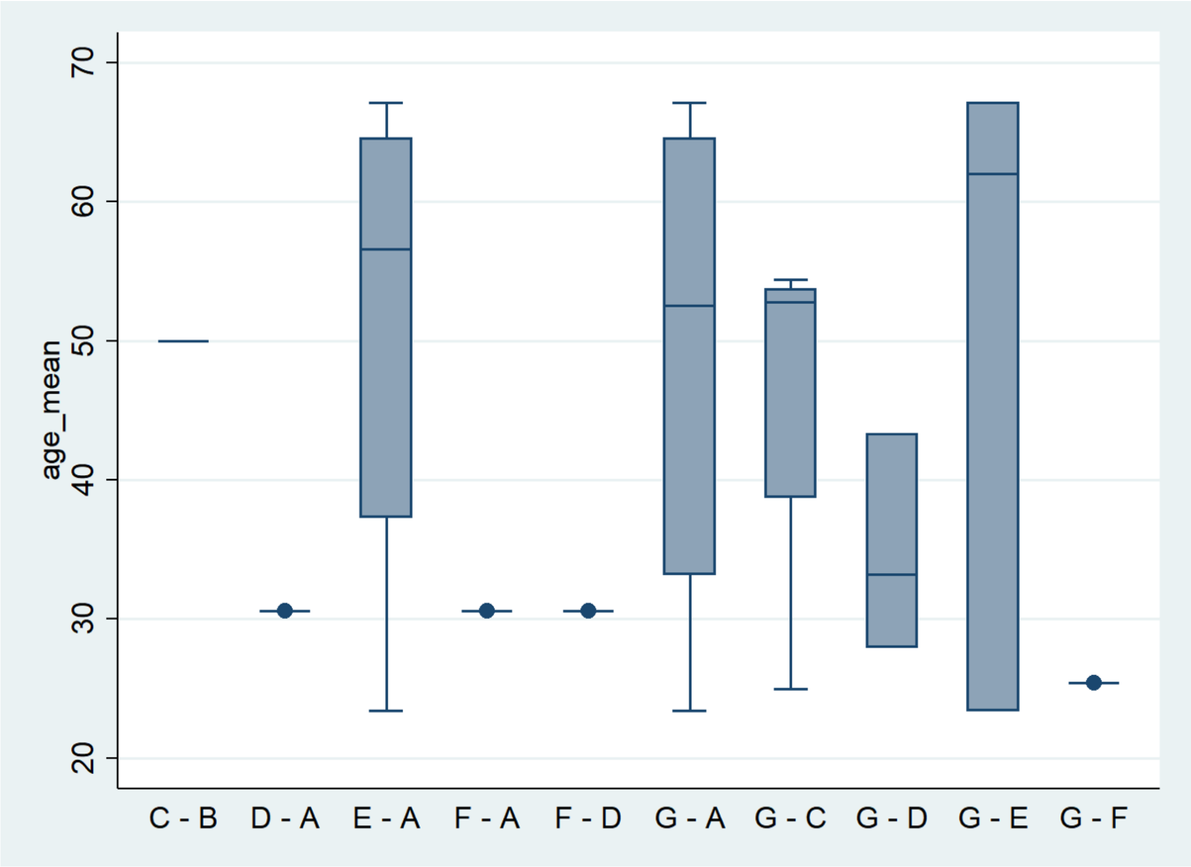

A = Mediterranean diet; **B** = Dietary Approaches to Stop Hypertension; **C** = Dietary Guidelines-based diets; **D** = plant-based diet; **E** = low fat diet; **F**= low carbohydrate high fat diet; **G** = western habitual diet

The lower and upper edges of the box represent the 25th and 75th percentiles, the middle line in the box marks the 50th percentile (median), the error bars are the 95% confidence intervals, and any dots beyond the error bars are outliers.

Supplementary Figure S6. Box plot showing the distribution of participants' age across the direct comparisons for glucose

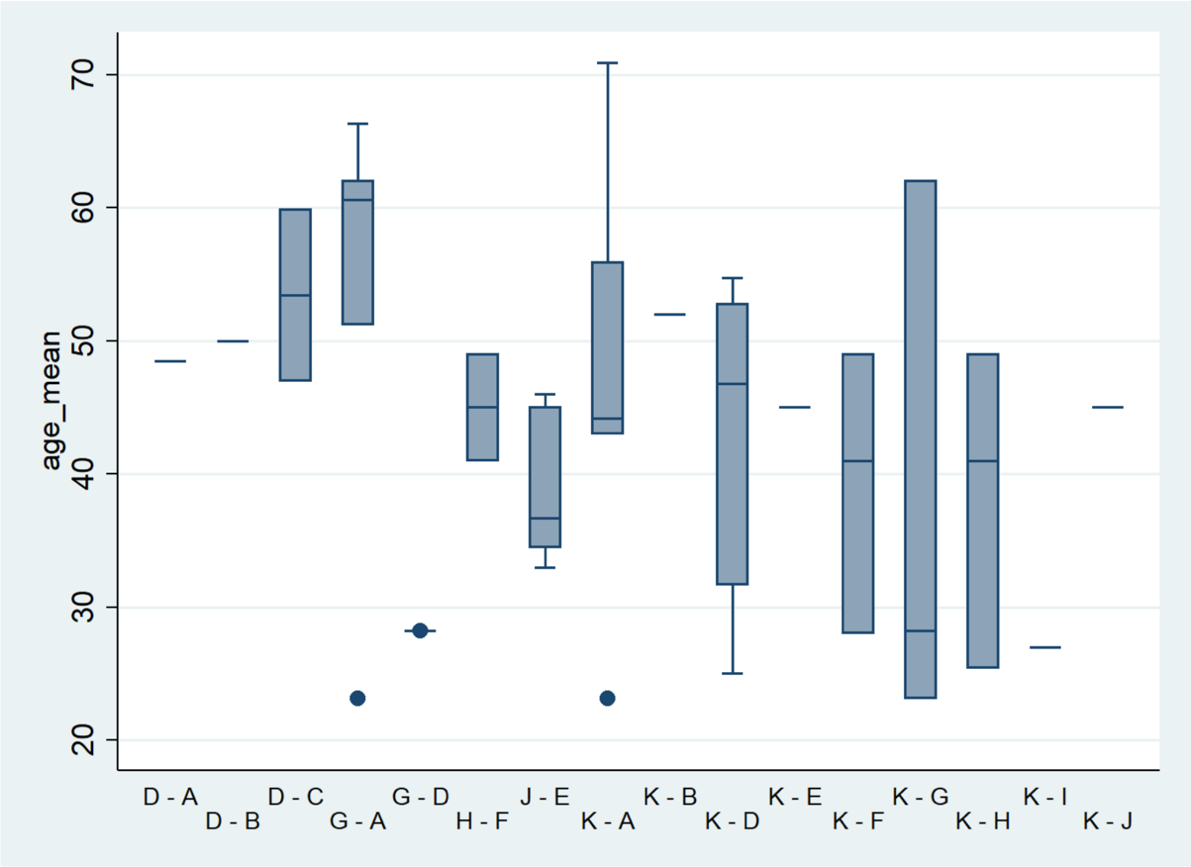

A = Mediterranean diet; **B** = Dietary Approaches to Stop Hypertension; **C** = Paleo diet; **D** = Dietary Guidelines-based diets; **E** = low GI / GL diet; **F** = plant-based diets; **G** = low fat diet; **H** = low carbohydrate high fat diet; **I** = traditional Mexican diet; **J** = high GI / GL diet; **K** = western habitual diet

The lower and upper edges of the box represent the 25th and 75th percentiles, the middle line in the box marks the 50th percentile (median), the error bars are the 95% confidence intervals, and any dots beyond the error bars are outliers.

Supplementary Figure S7. Box plot showing the distribution of participants' age across the direct comparisons for hsCRP

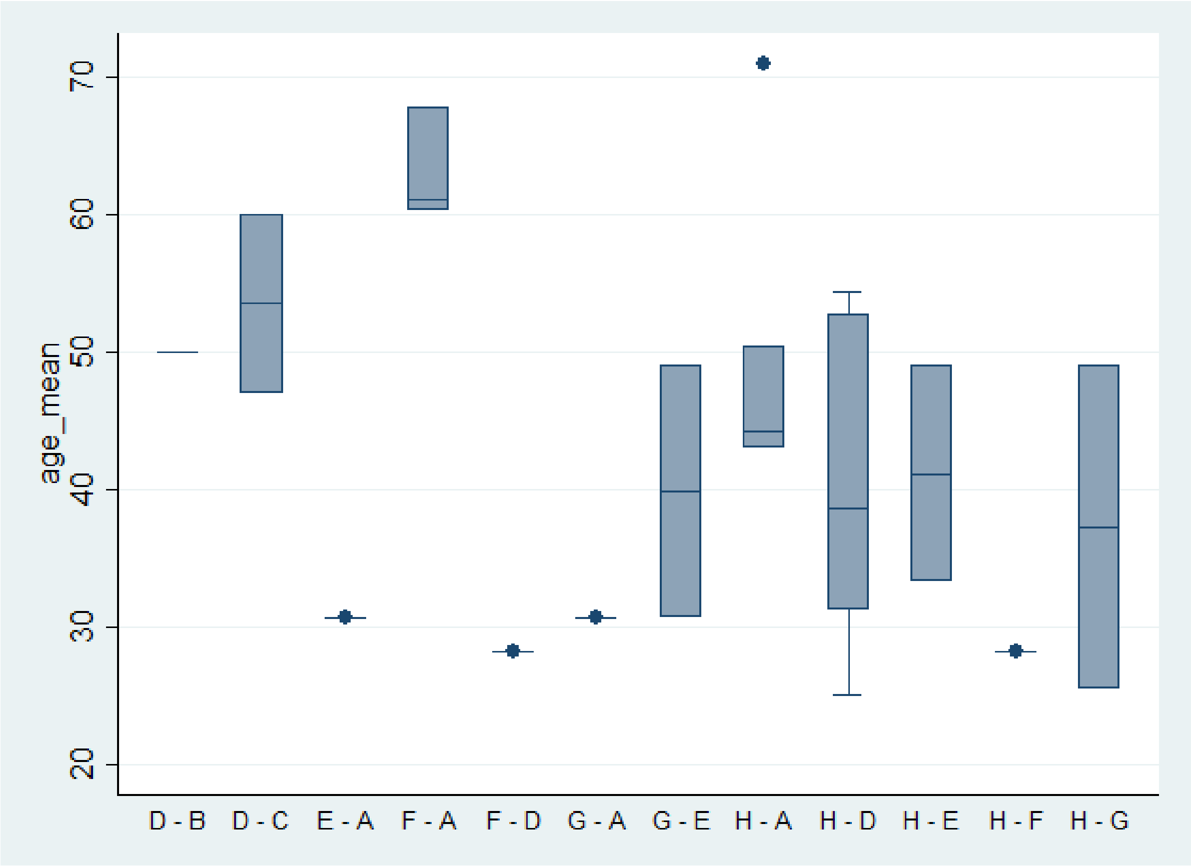

A = Mediterranean diet; **B** = Dietary Approaches to Stop Hypertension; **C** = Paleo diet; **D** = Dietary Guidelines-based diets; **E** = plant-based diets; **F** = low fat diet; **G** = low carbohydrate high fat diet; **H** = western habitual diet

The lower and upper edges of the box represent the 25th and 75th percentiles, the middle line in the box marks the 50th percentile (median), the error bars are the 95% confidence intervals, and any dots beyond the error bars are outliers.

Supplementary Figure S8. Box plot showing the distribution of participants' age across the direct comparisons for interleukin-6

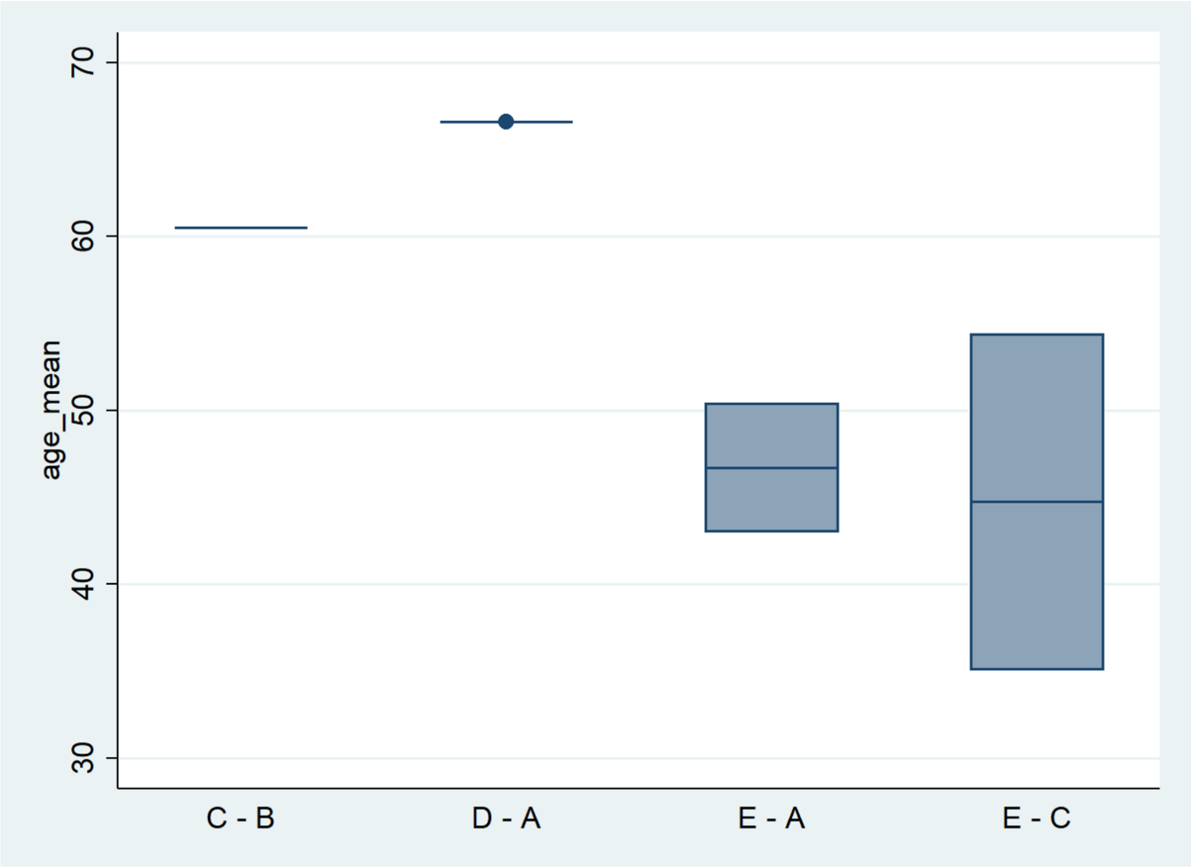

A = Mediterranean diet; **B** = Paleo diet; **C** = Dietary Guidelines-based diets; **D** = low fat diet; **E** = western habitual diet

The lower and upper edges of the box represent the 25th and 75th percentiles, the middle line in the box marks the 50th percentile (median), the error bars are the 95% confidence intervals, and any dots beyond the error bars are outliers.

Supplementary Figure S9. Box plot showing the distribution of percentage of female participants across the direct comparisons for LDL-c

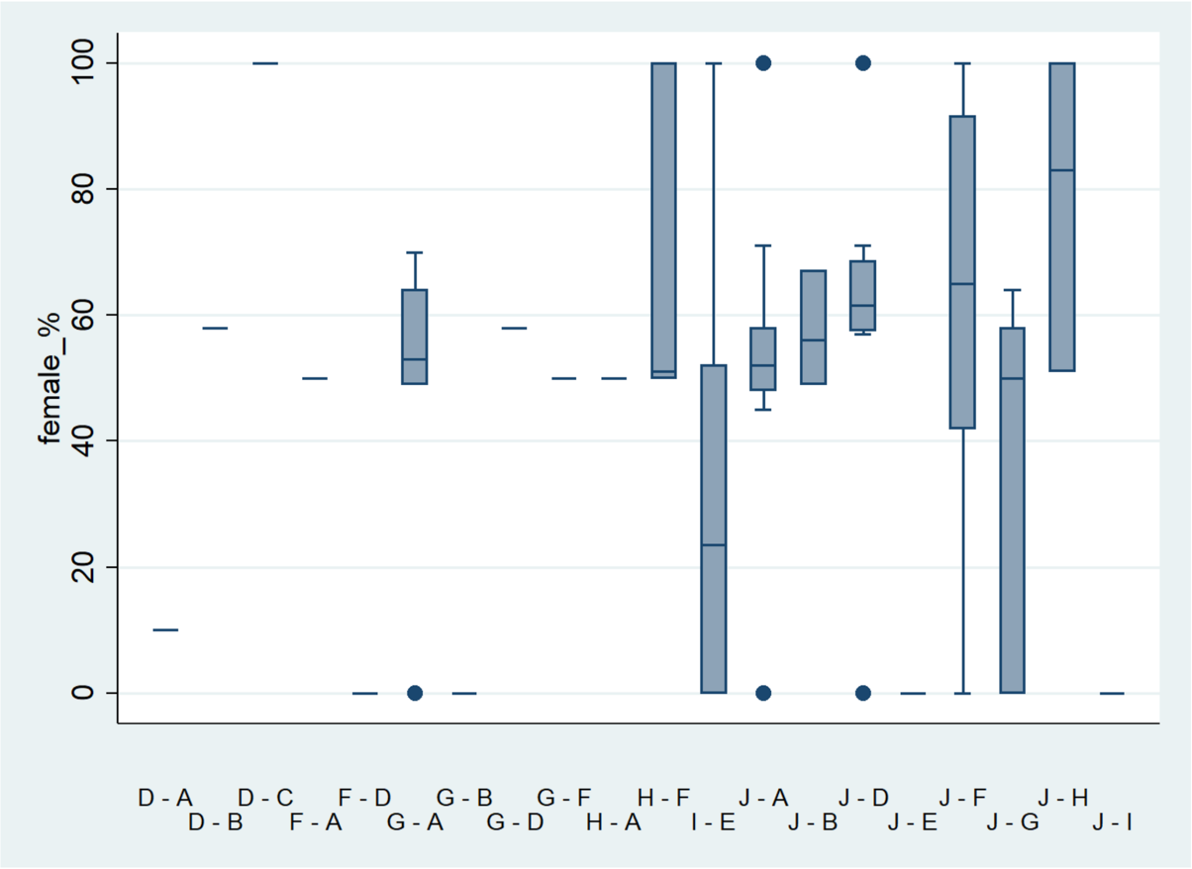

A = Mediterranean diet; **B** = Dietary Approaches to Stop Hypertension; **C** = Paleo diet; **D** = Dietary Guidelines-based diets; **E** = low GI / GL diet; **F** = plant-based diets; **G** = low fat diet; **H** = low carbohydrate high fat diet; **I** = high GI / GL diet; **J** = western habitual diet

The lower and upper edges of the box represent the 25th and 75th percentiles, the middle line in the box marks the 50th percentile (median), the error bars are the 95% confidence intervals, and any dots beyond the error bars are outliers.

Supplementary Figure S10. Box plot showing the distribution of percentage of female participants across the direct comparisons for glucose

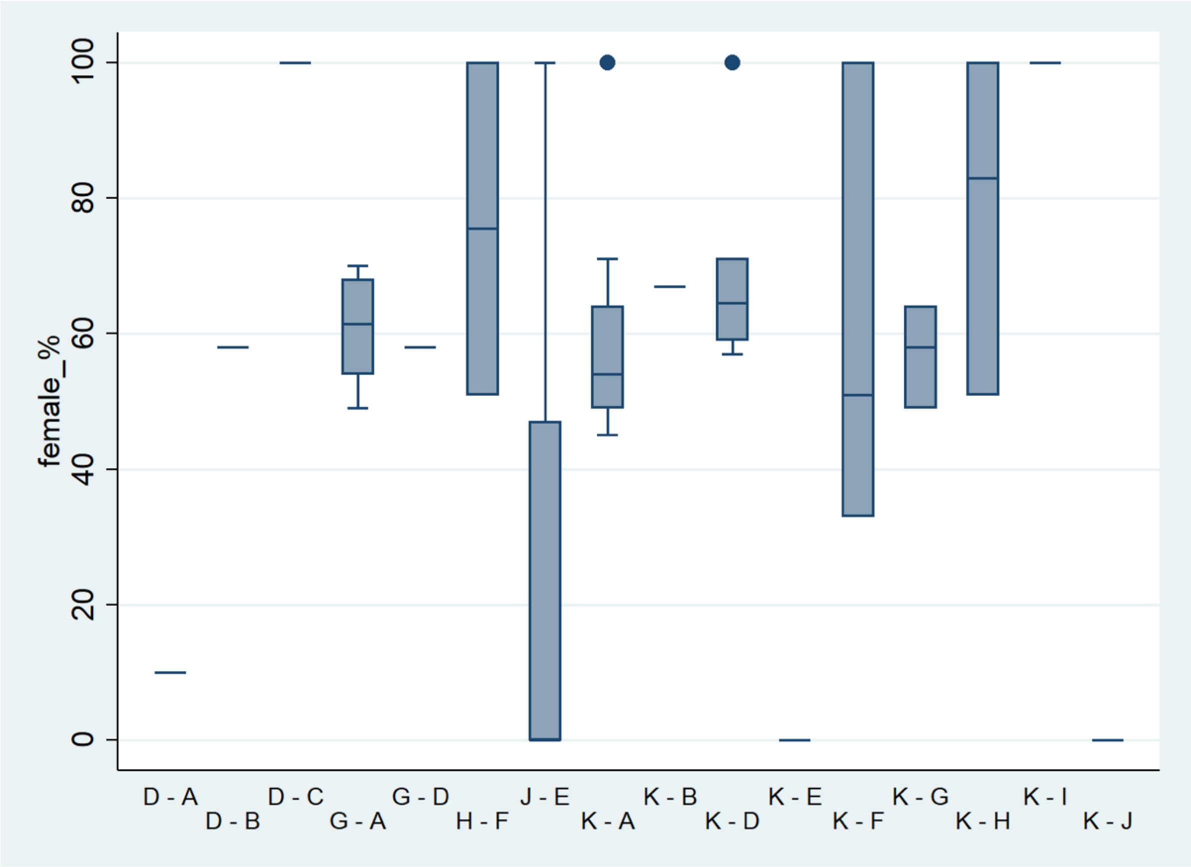

A = Mediterranean diet; **B** = Dietary Approaches to Stop Hypertension; **C** = Paleo diet; **D** = Dietary Guidelines-based diets; **E** = low GI / GL diet; **F** = plant-based diets; **G** = low fat diet; **H** = low carbohydrate high fat diet; **I** = traditional Mexican diet; **J** = high GI / GL diet; **K** = western habitual diet

The lower and upper edges of the box represent the 25th and 75th percentiles, the middle line in the box marks the 50th percentile (median), the error bars are the 95% confidence intervals, and any dots beyond the error bars are outliers.

Supplementary Figure S11. Box plot showing the distribution of percentage of female participants across the direct comparisons for hsCRP

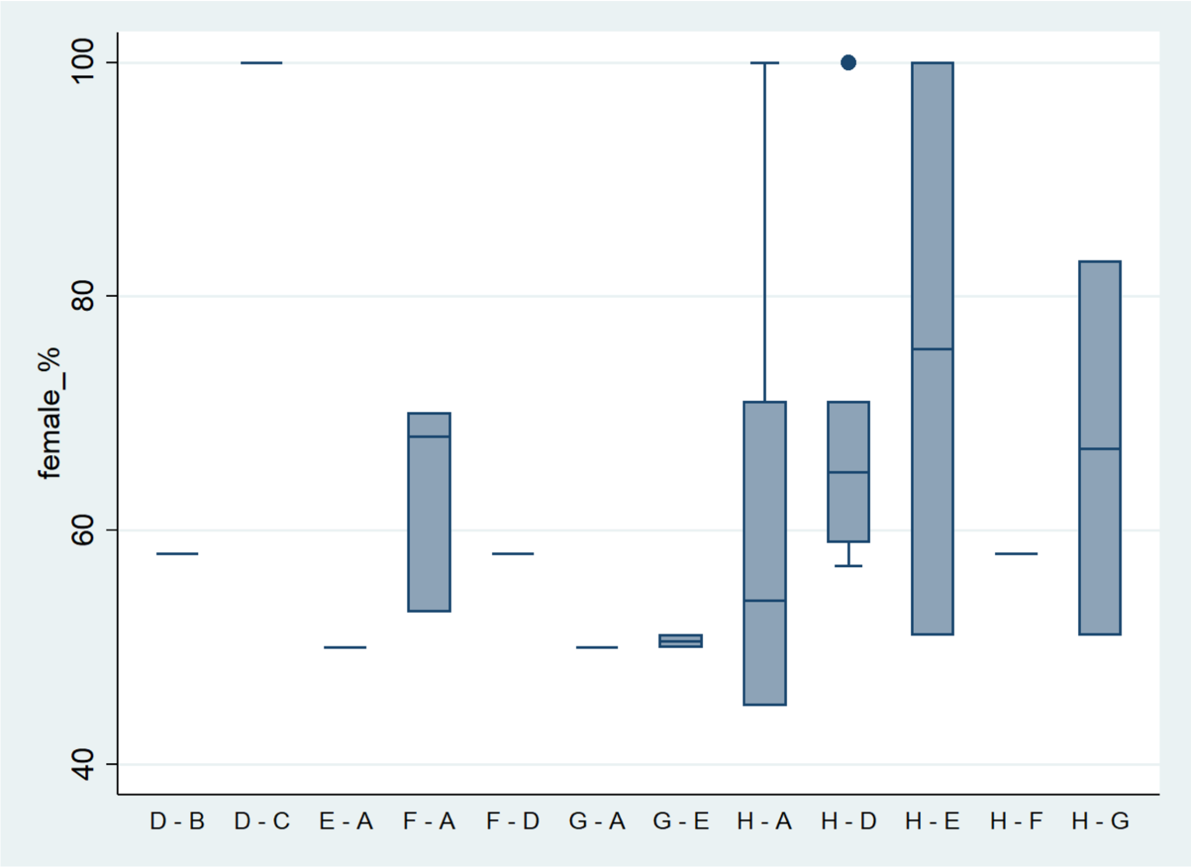

A = Mediterranean diet; **B** = Dietary Approaches to Stop Hypertension; **C** = Paleo diet; **D** = Dietary Guidelines-based diets; **E** = plant-based diets; **F** = low fat diet; **G** = low carbohydrate high fat diet; **H** = western habitual diet

The lower and upper edges of the box represent the 25th and 75th percentiles, the middle line in the box marks the 50th percentile (median), the error bars are the 95% confidence intervals, and any dots beyond the error bars are outliers.

Supplementary Figure S12. Box plot showing the distribution of intervention duration across the direct comparisons for LDL-c

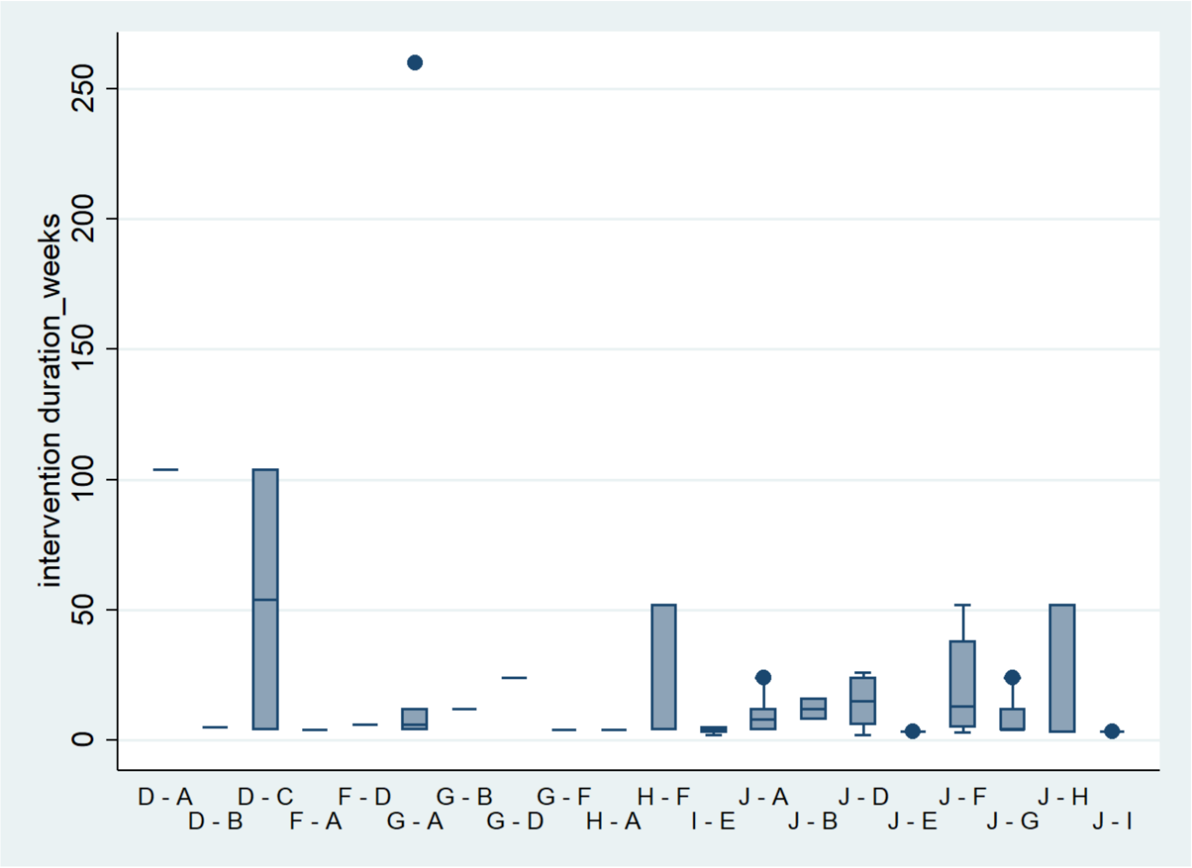

A = Mediterranean diet; **B** = Dietary Approaches to Stop Hypertension; **C** = Paleo diet; **D** = Dietary Guidelines-based diets; **E** = low GI / GL diet; **F** = plant-based diets; **G** = low fat diet; **H** = low carbohydrate high fat diet; **I** = high GI / GL diet; **J** = western habitual diet

The lower and upper edges of the box represent the 25th and 75th percentiles, the middle line in the box marks the 50th percentile (median), the error bars are the 95% confidence intervals, and any dots beyond the error bars are outliers.

Supplementary Figure S13. Box plot showing the distribution of intervention duration across the direct comparisons for glucose

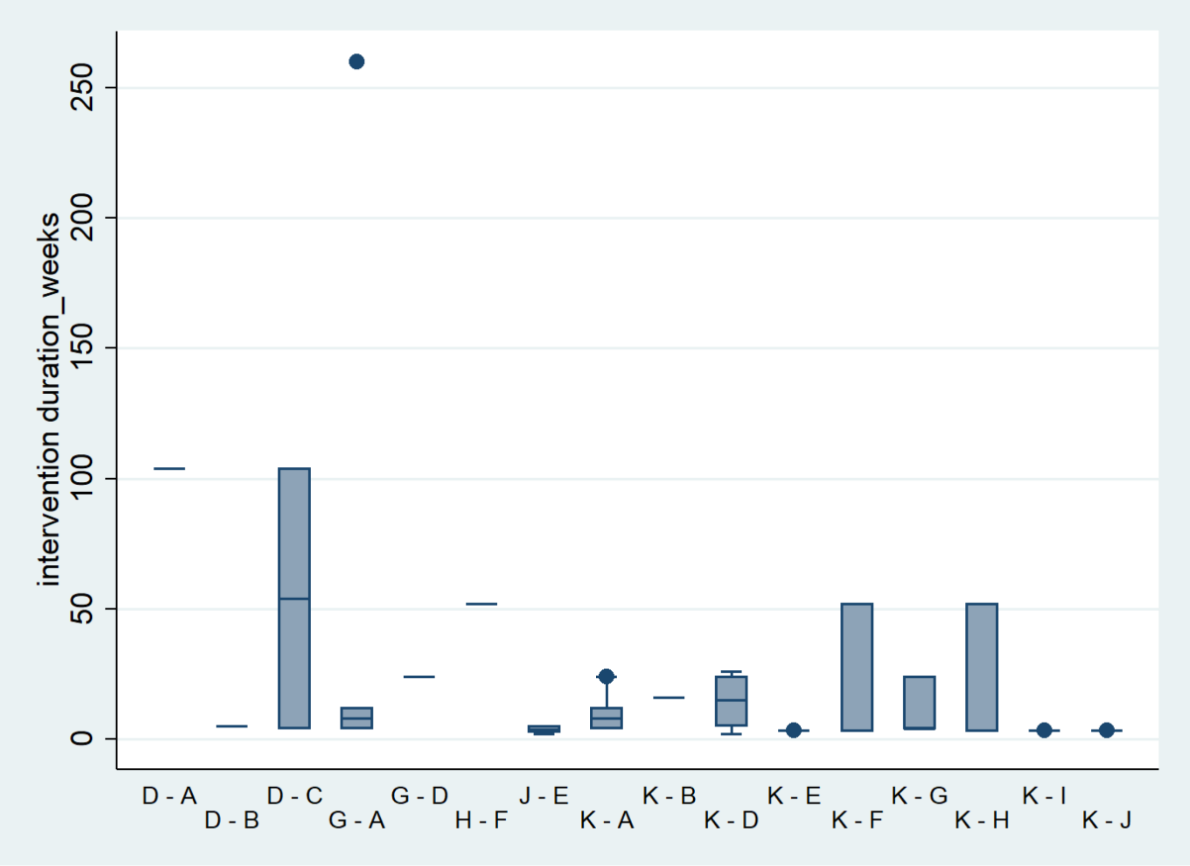

A = Mediterranean diet; **B** = Dietary Approaches to Stop Hypertension; **C** = Paleo diet; **D** = Dietary Guidelines-based diets; **E** = low GI / GL diet; **F** = plant-based diets; **G** = low fat diet; **H** = low carbohydrate high fat diet; **I** = traditional Mexican diet; **J** = high GI / GL diet; **K** = western habitual diet

The lower and upper edges of the box represent the 25th and 75th percentiles, the middle line in the box marks the 50th percentile (median), the error bars are the 95% confidence intervals, and any dots beyond the error bars are outliers.

Supplementary Figure S14. Box plot showing the distribution of intervention duration across the direct comparisons for hsCRP

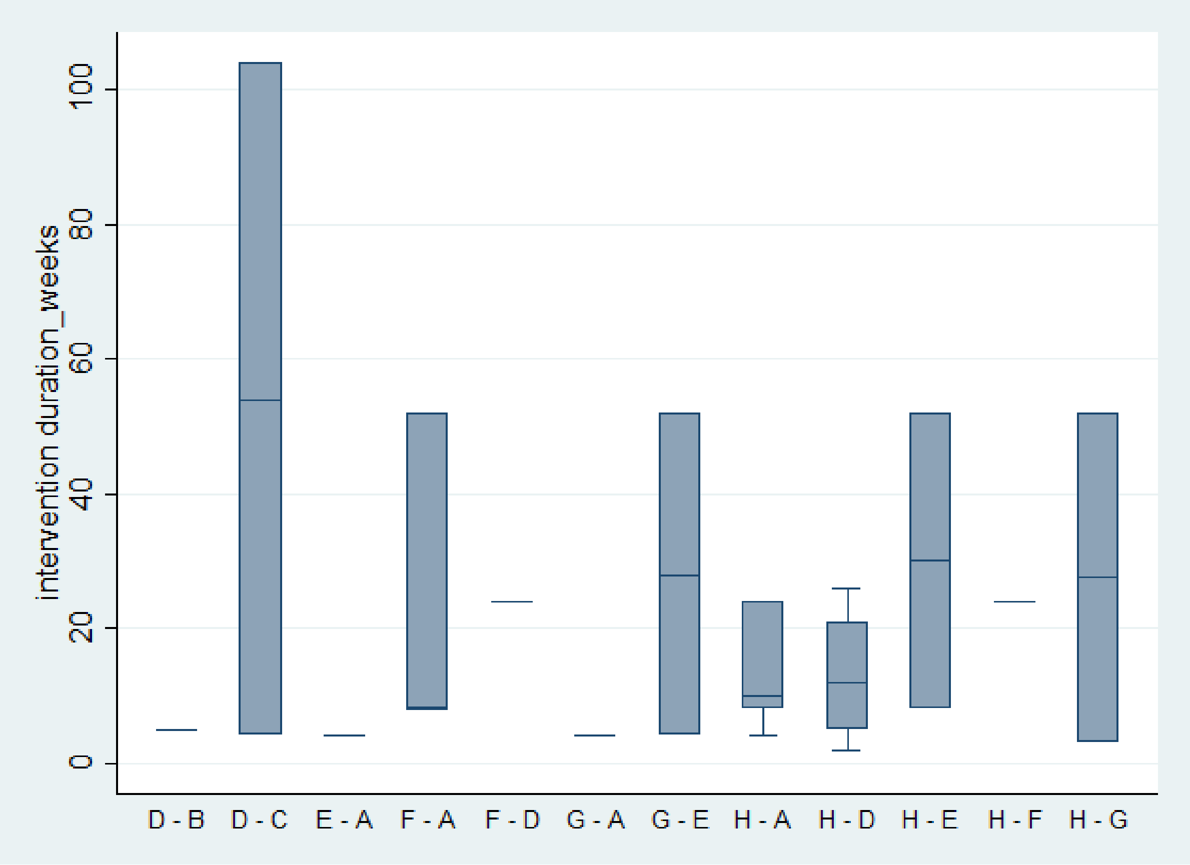

A = Mediterranean diet; **B** = Dietary Approaches to Stop Hypertension; **C** = Paleo diet; **D** = Dietary Guidelines-based diets; **E** = plant-based diets; **F** = low fat diet; **G** = low carbohydrate high fat diet; **H** = western habitual diet

The lower and upper edges of the box represent the 25th and 75th percentiles, the middle line in the box marks the 50th percentile (median), the error bars are the 95% confidence intervals, and any dots beyond the error bars are outliers.

Supplementary Figure S15. Rankograms for LDL-c[1]

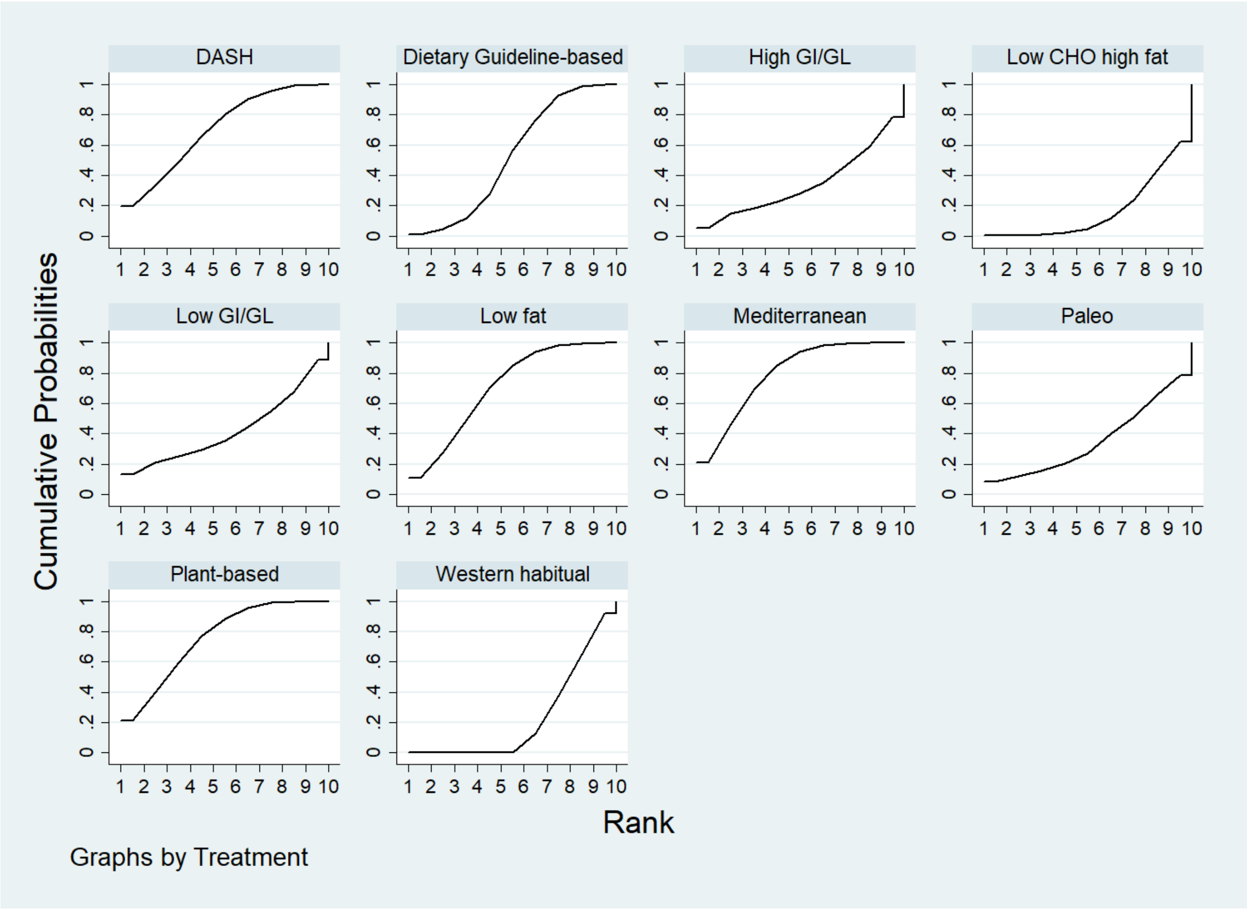

[1]The rankograms in general showed varied distribution of rank probabilities, except for the DASH, low fat, Mediterranean, and plant-based diet indicating uncertainty in their ranking.

Abbreviations: CHO, carbohydrate; DASH, Dietary Approaches to Stop Hypertension; GI/GL, glycemic index/glycemic load

Supplementary Figure S16. Rankograms for HDL-c[1]

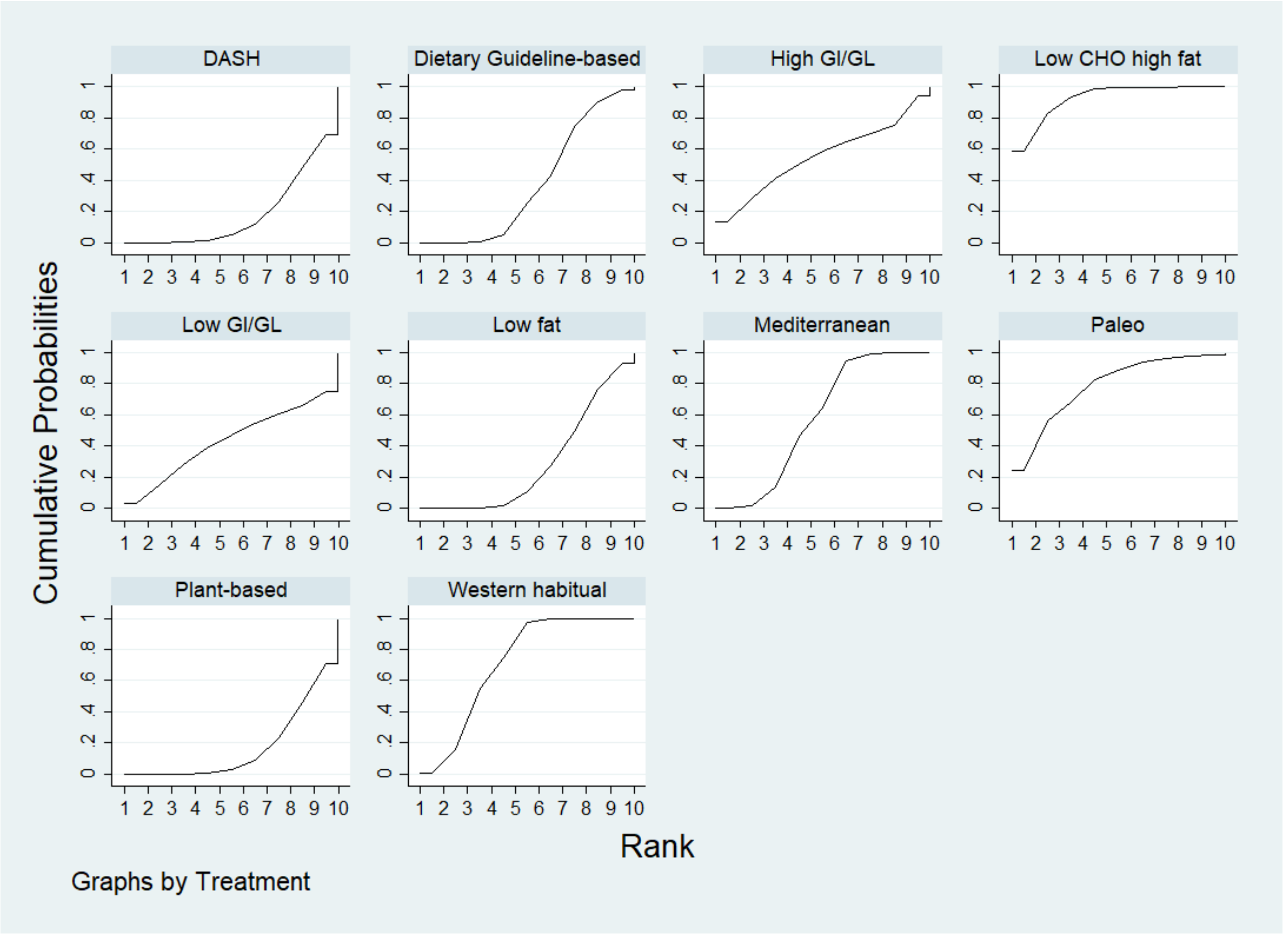

[1]The rankograms in general showed varied distribution of rank probabilities, except for the DASH, dietary guideline-based, low fat, plant-based diet indicating uncertainty in their ranking.

Abbreviations: CHO, carbohydrate; DASH, Dietary Approaches to Stop Hypertension; GI/GL, glycemic index/glycemic load

Supplementary Figure S17. Rankograms for total cholesterol[1]

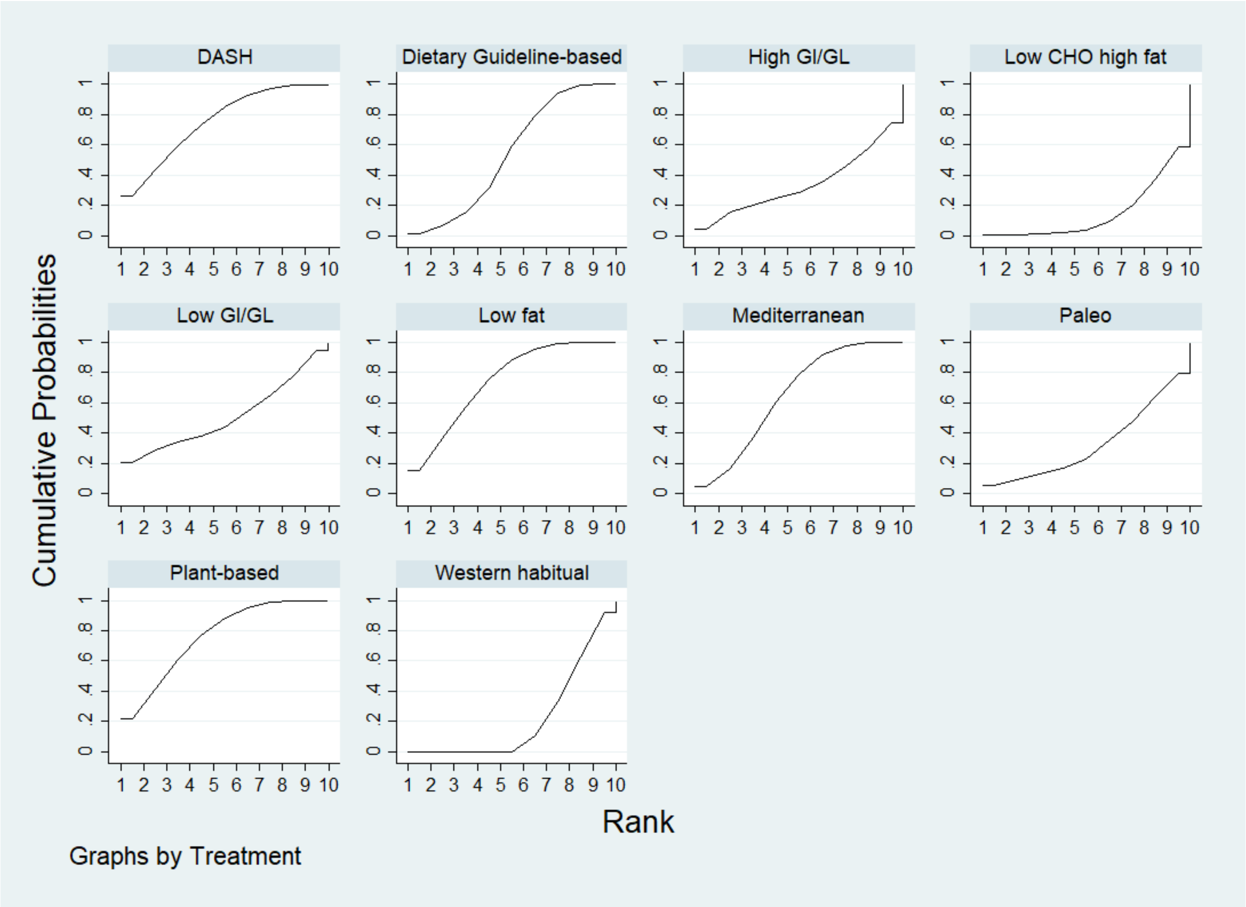

[1]The rankograms in general showed varied distribution of rank probabilities, except for the DASH, dietary guideline-based, low fat, Mediterranean and plant-based diet indicating uncertainty in their ranking.

Abbreviations: CHO, carbohydrate; DASH, Dietary Approaches to Stop Hypertension; GI/GL, glycemic index/glycemic load

Supplementary Figure S18. Rankograms for triglycerides[1]

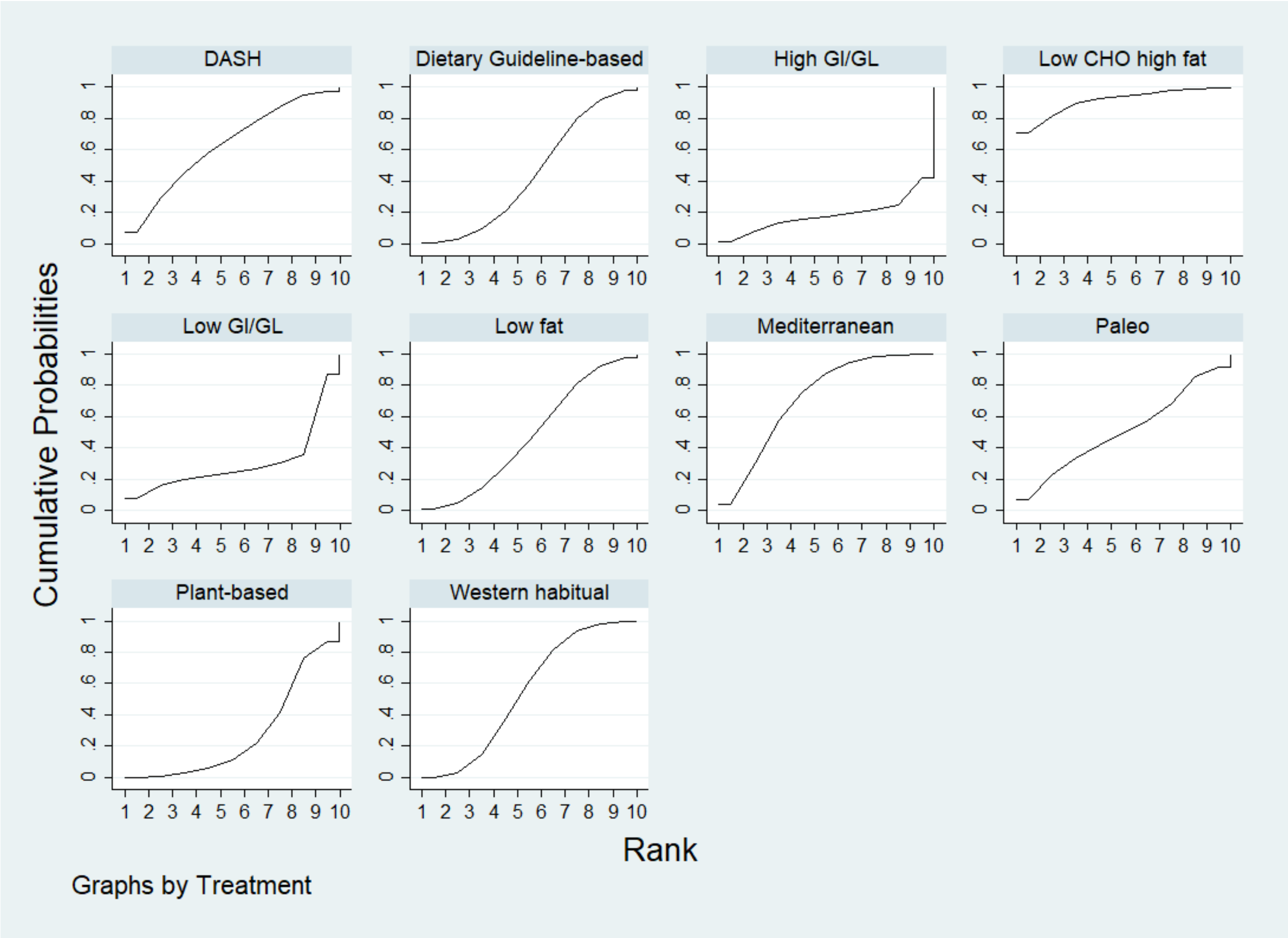

[1]The rankograms in general showed varied distribution of rank probabilities, except for the dietary guideline-based, low fat, and western habitual diet indicating uncertainty in their ranking.

Abbreviations: CHO, carbohydrate; DASH, Dietary Approaches to Stop Hypertension; GI/GL, glycemic index/glycemic load

Supplementary Figure S19. Rankograms for ApoB[1]

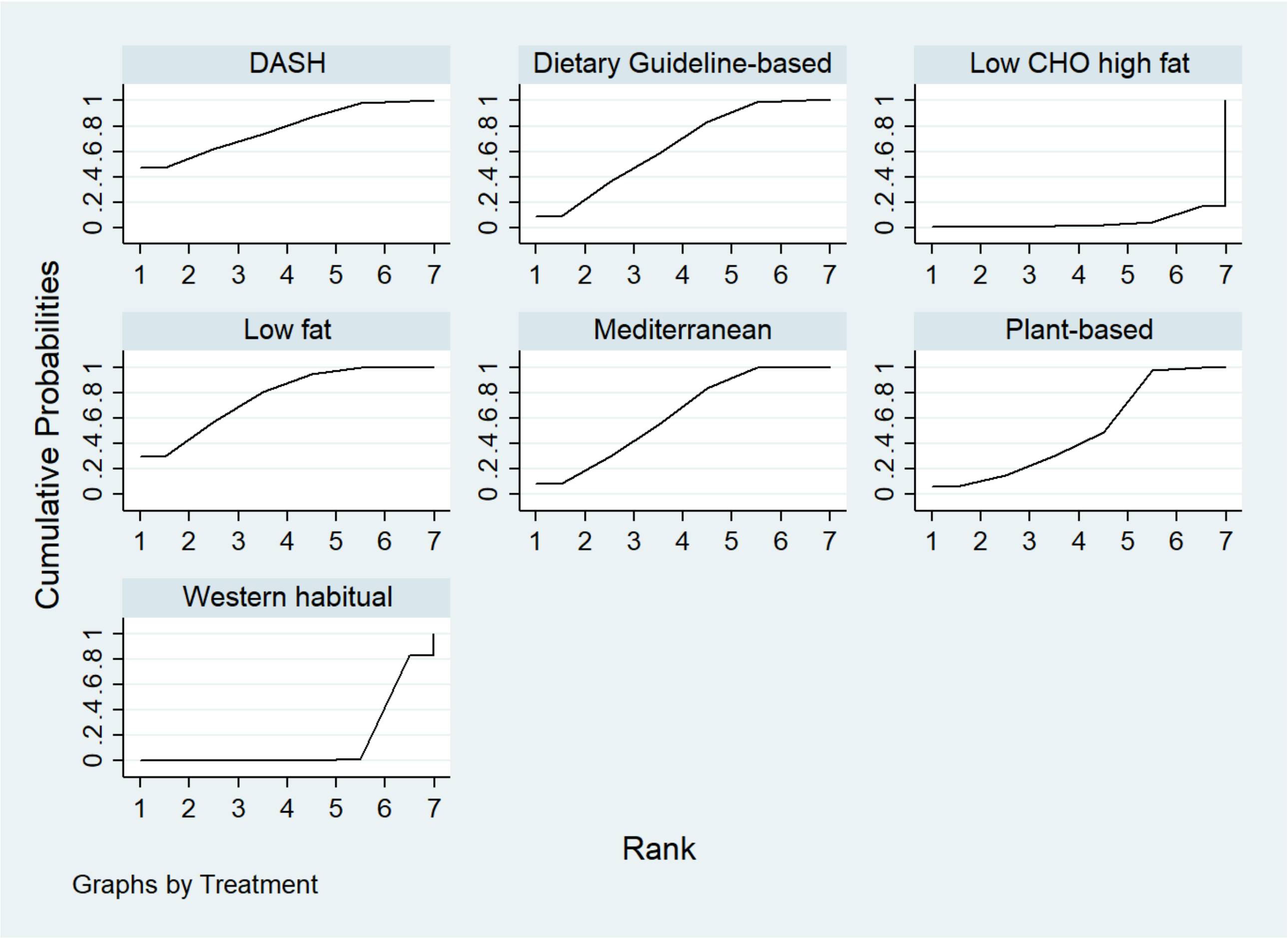

[1]The rankograms in general showed varied distribution of rank probabilities, except for the dietary guideline-based and Mediteranean diet indicating uncertainty in their ranking.

Abbreviations: CHO, carbohydrate; DASH, Dietary Approaches to Stop Hypertension

Supplementary Figure S20. Rankograms for ApoA1[1]

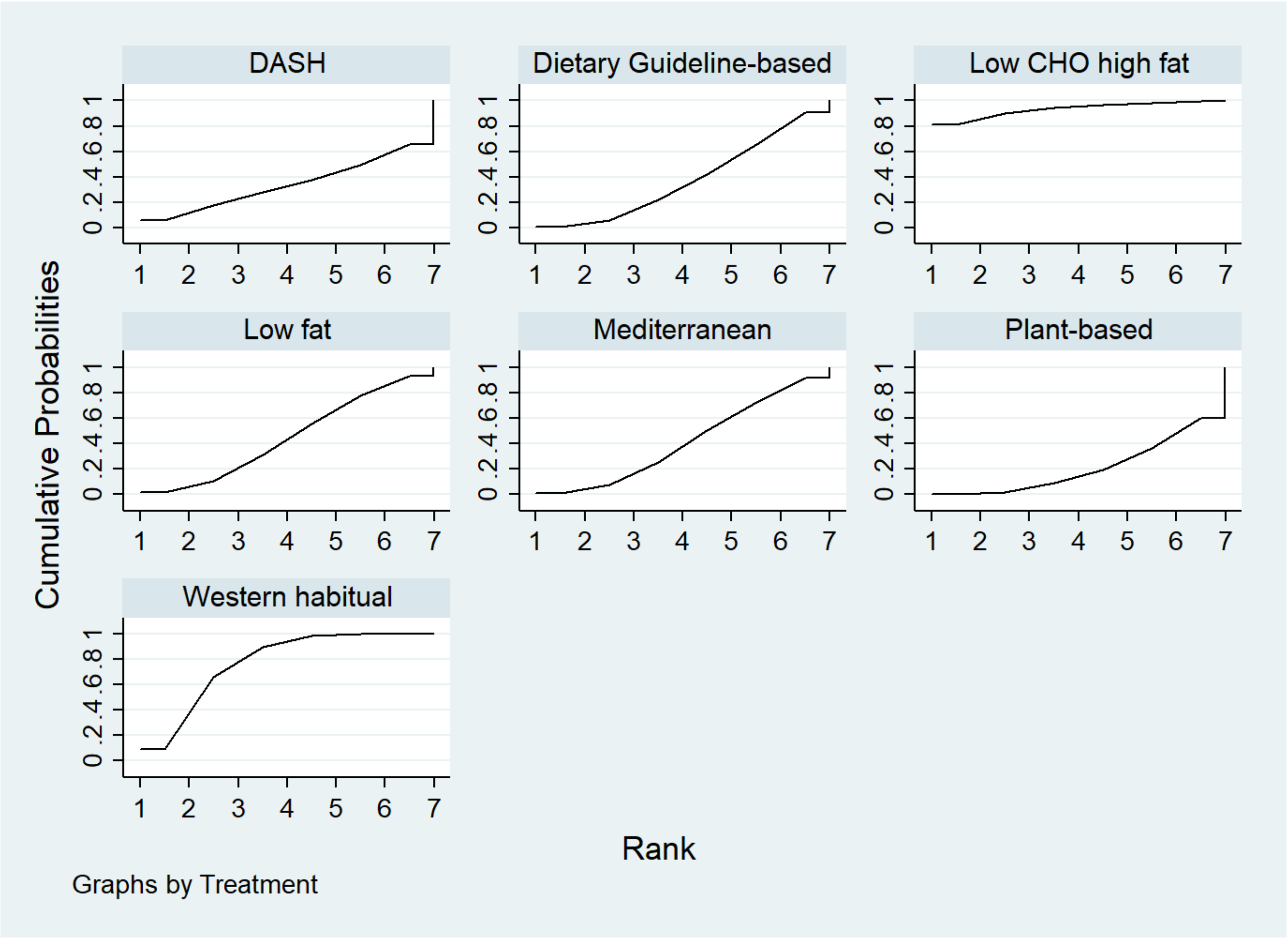

[1]The rankograms in general showed varied distribution of rank probabilities, except for the dietary guideline-based and Mediterranean diet indicating uncertainty in their ranking.

Abbreviations: CHO, carbohydrate; DASH, Dietary Approaches to Stop Hypertension

Supplementary Figure S21. Rankograms for glucose[1]

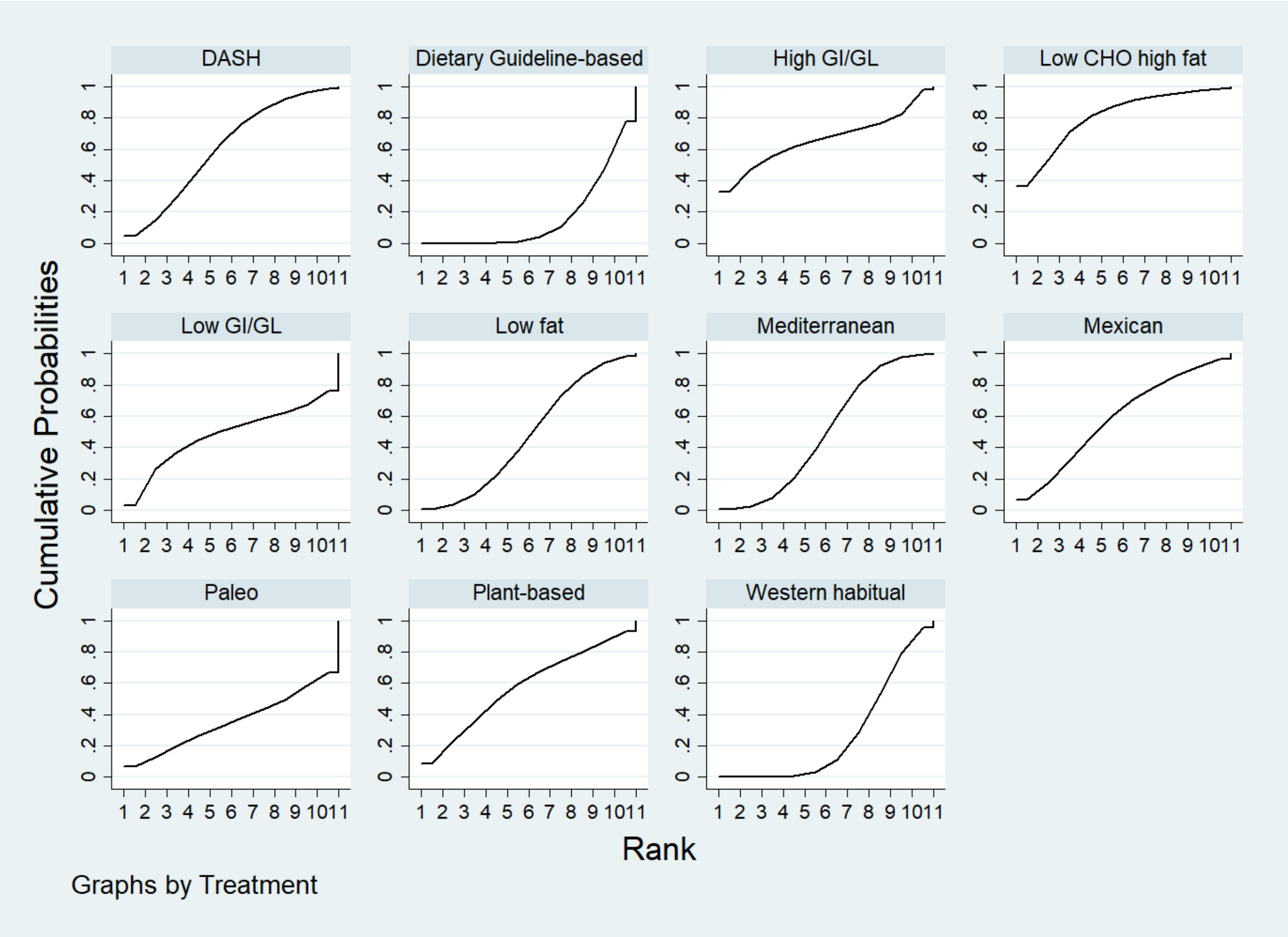

[1]The rankograms in general showed varied distribution of rank probabilities, except for the DASH, low fat, Mediterranean diet indicating uncertainty in their ranking.

Abbreviations: CHO, carbohydrate; DASH, Dietary Approaches to Stop Hypertension; GI/GL, glycemic index/glycemic load

Supplementary Figure S22. Rankograms for insulin[1]

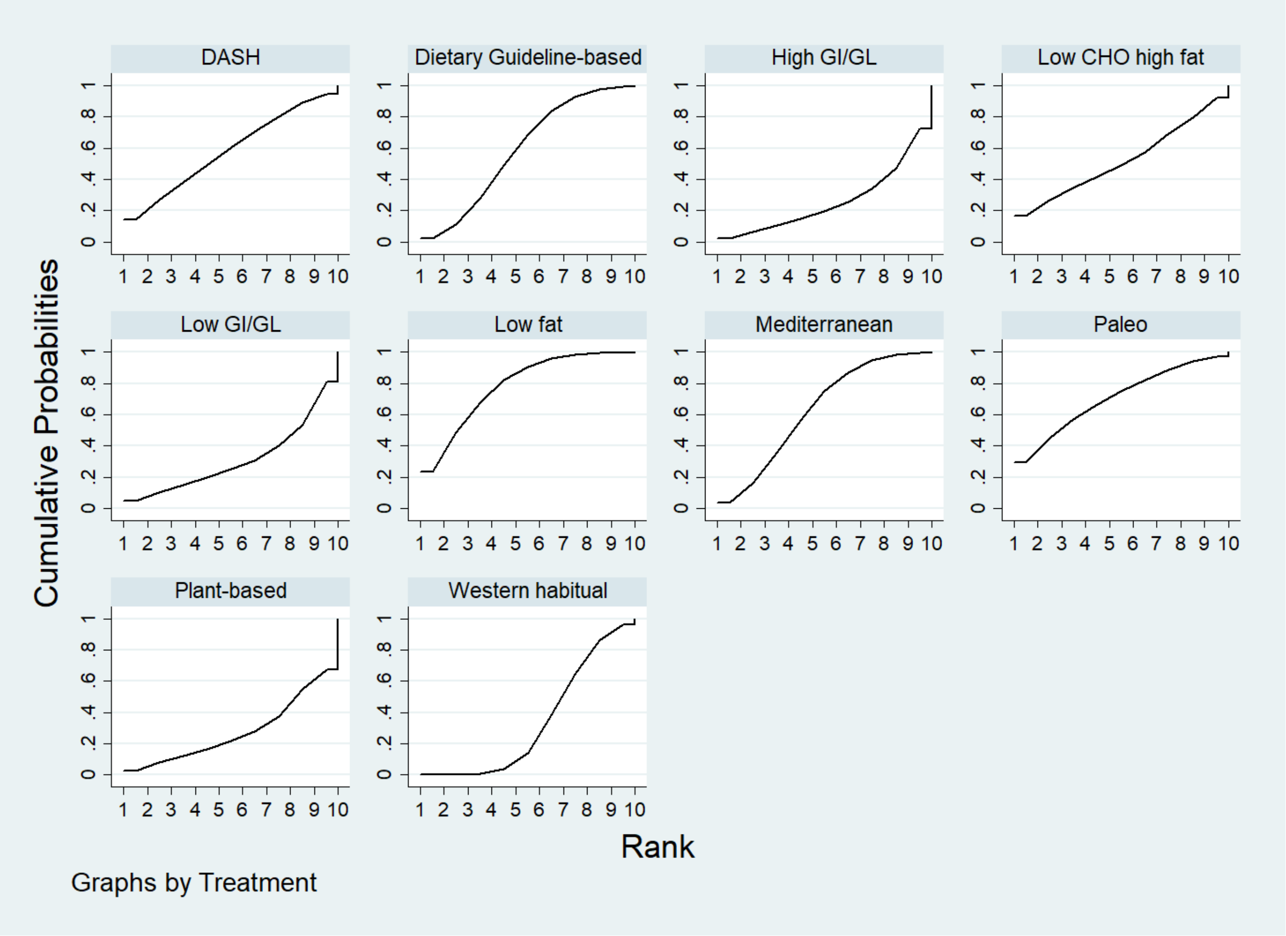

[1]The rankograms in general showed varied distribution of rank probabilities, except for the DASH, dietary guideline-based, high GI/GL, low GI/GL, Mediterranean, paleo, and plant-based diet indicating uncertainty in their ranking.

Abbreviations: CHO, carbohydrate; DASH, Dietary Approaches to Stop Hypertension; GI/GL, glycemic index/glycemic load

Supplementary Figure S23. Rankograms for HOMA-IR[1]

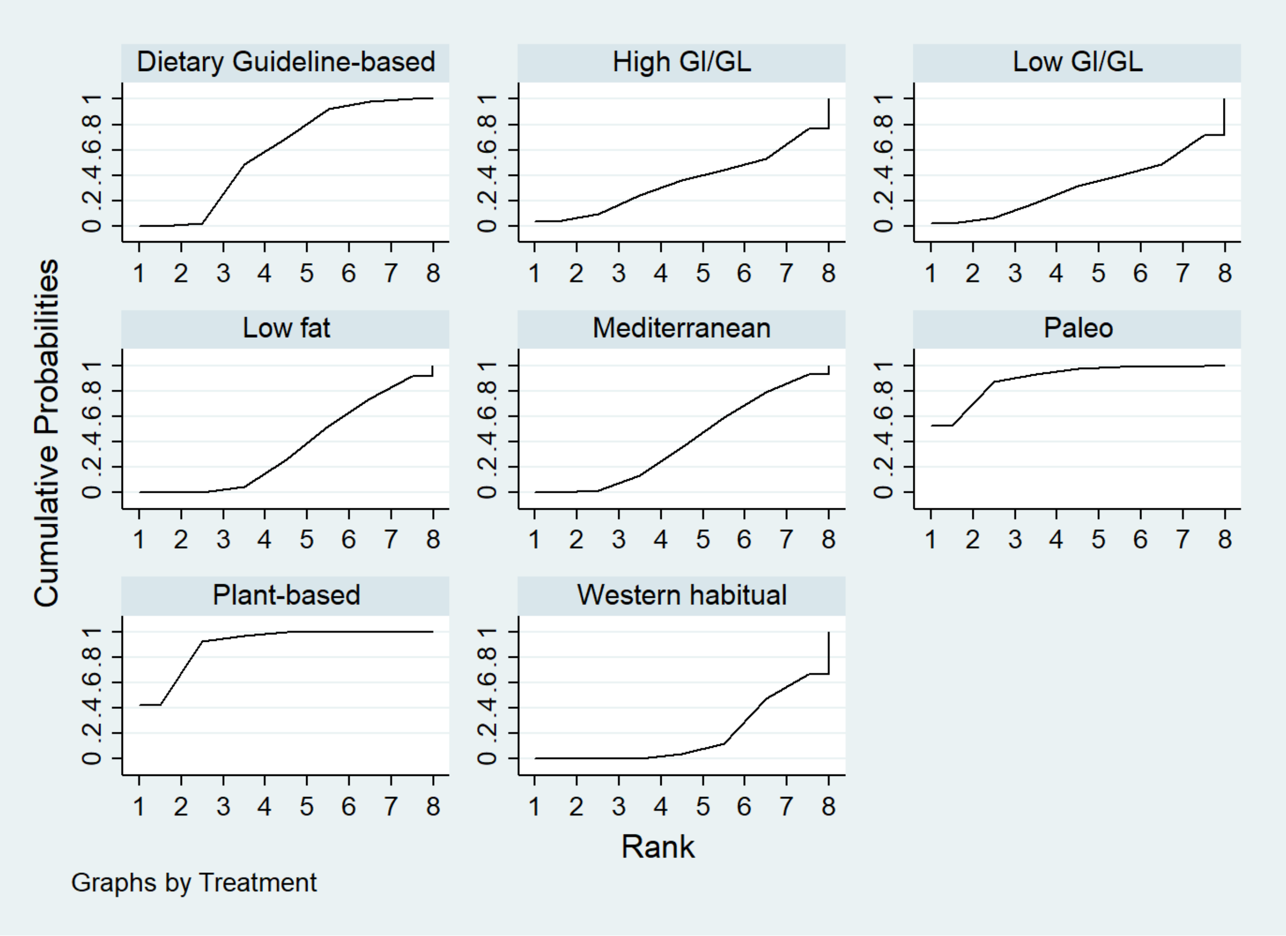

[1]The rankograms in general showed varied distribution of rank probabilities, except for the high GI/GL, low GI/GL, paleo and plant-based diet indicating uncertainty in their ranking.

Abbreviations: GI/GL, glycemic index/glycemic load

Supplementary Figure S24. Rankograms for hsCRP[1]

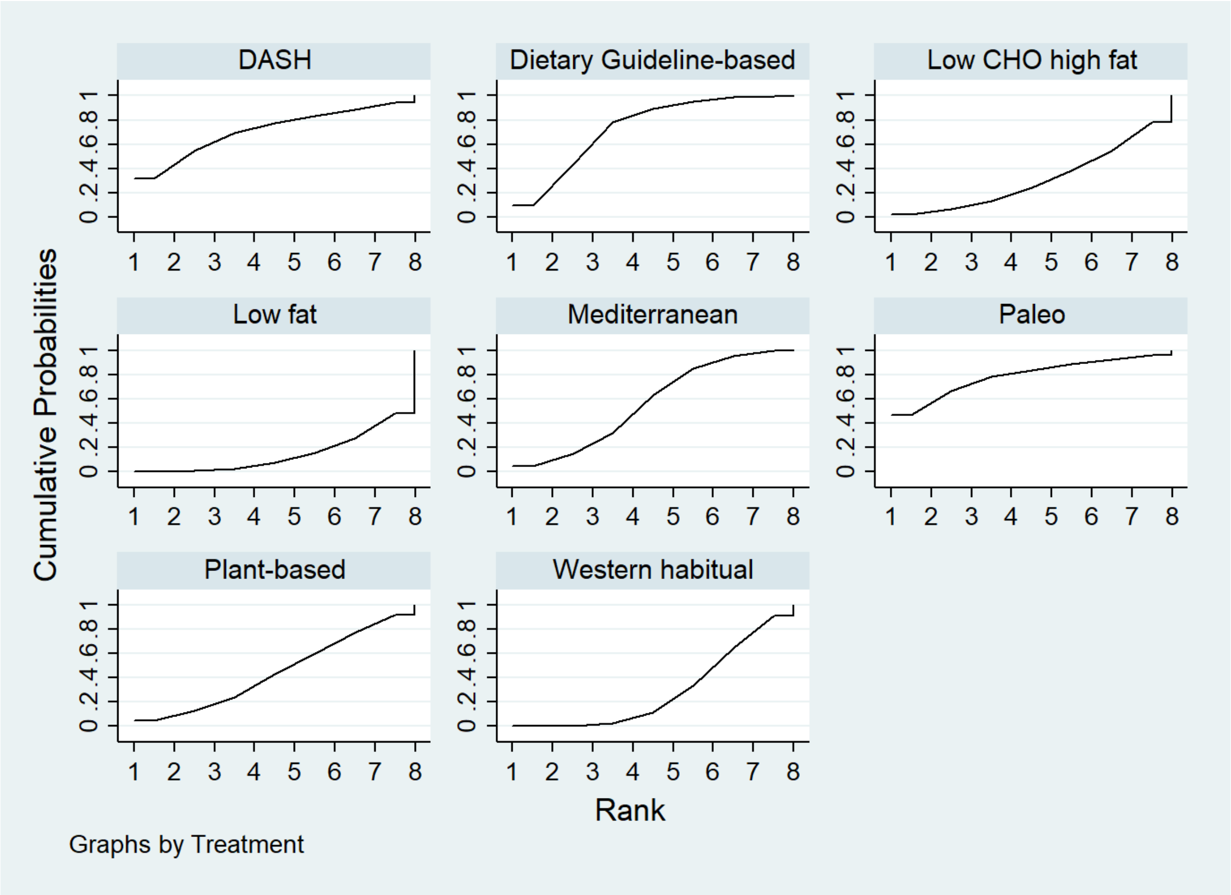

[1]The rankograms in general showed varied distribution of rank probabilities, except for the DASH, low CHO high fat, Mediterranean, and plant-based diet indicating uncertainty in their ranking.

Abbreviations: CHO, carbohydrate; DASH, Dietary Approaches to Stop Hypertension

Supplementary Figure S25. Rankograms for interleukin-6[1]

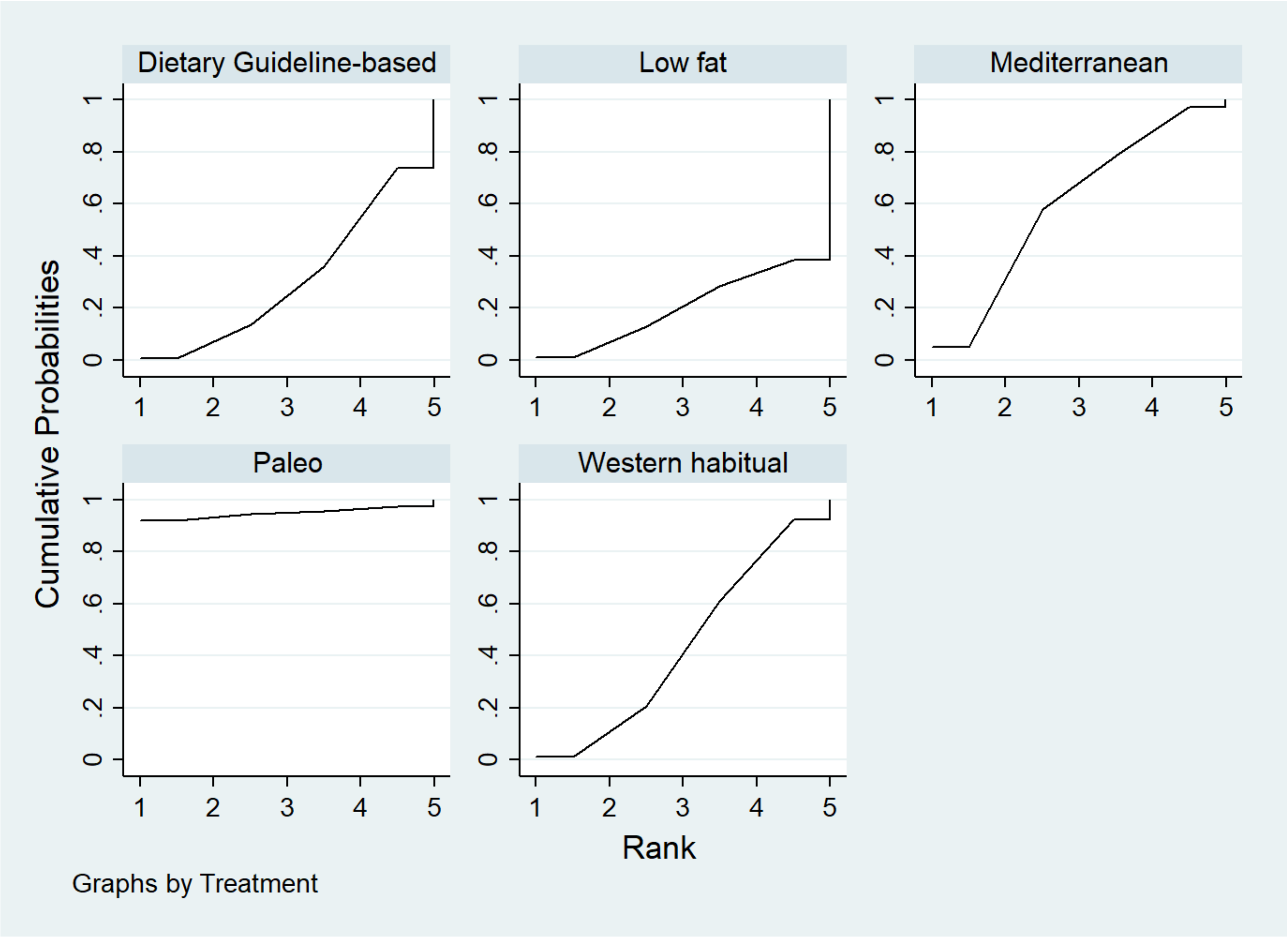

[1]The rankograms in general showed varied distribution of rank probabilities, indicating relatively high certainty in their ranking.

Supplementary Table S15. Loop-specific approach assessing inconsistency for LDL-c

Loop	IF	Standard Error	z value	p value	95% CI	Loop-specific Heterogeneity (T^2)
D-F-G	0.63	0.20	3.08	0.00	(0.23,1.03)	0.000
A-D-G	0.48	0.19	2.55	0.01	(0.11,0.86)	0.000
A-D-J	0.46	0.32	1.45	0.15	(0.00,1.07)	0.064
A-F-H	0.40	0.56	0.72	0.47	(0.00,1.50)	0.000
D-F-J	0.34	0.24	1.44	0.15	(0.00,0.81)	0.021
B-D-G	0.34	0.31	1.11	0.27	(0.00,0.94)	0.000
F-H-J	0.31	0.26	1.20	0.23	(0.00,0.82)	0.023
B-D-J	0.29	0.33	0.89	0.37	(0.00,0.94)	0.041
F-G-J	0.24	0.17	1.43	0.15	(0.00,0.58)	0.016
A-F-J	0.23	0.48	0.48	0.63	(0.00,1.18)	0.043
E-I-J	0.23	0.49	0.48	0.63	(0.00,1.18)	0.015
A-H-J	0.23	0.52	0.44	0.66	(0.00,1.24)	0.071
A-F-G	0.09	0.41	0.21	0.83	(0.00,0.88)	0.000
D-G-J	0.07	0.34	0.22	0.83	(0.00,0.74)	0.044
A-G-J	0.06	0.16	0.40	0.69	(0.00,0.37)	0.030
A-D-F	0.06	0.42	0.14	0.89	(0.00,0.88)	0.000
B-G-J	0.06	0.29	0.19	0.85	(0.00,0.62)	0.016

A = Mediterranean diet; **B** = Dietary Approaches to Stop Hypertension; **D** = Dietary guidelines-based diets; **E** = low GI / GL diet; **F** = plant-based diets; **G** = low fat diet; **H** = low carbohydrate high fat diet; **I** = high GI / GL diet; **J** = western habitual diet

Inconsistency was inferred if IF ≥ 0.7 and the lower limit of CI does not read zero and *p* value ≤ 0.05.

Abbreviations: IF, inconsistency factor

Supplementary Table S16. Loop-specific approach assessing inconsistency for HDL-c

Loop	IF	Standard Error	z value	p value	95% CI	Loop-specific Heterogeneity (T^2)
A-F-G	0.21	0.20	1.08	0.28	(0.00,0.60)	0.000
D-F-J	0.16	0.08	1.89	0.06	(0.00,0.32)	0.001
A-D-F	0.15	0.20	0.74	0.46	(0.00,0.55)	0.000
F-G-J	0.11	0.04	2.83	0.01	(0.03,0.18)	0.000
A-D-G	0.10	0.08	1.35	0.18	(0.00,0.25)	0.000
A-F-J	0.08	0.20	0.43	0.67	(0.00,0.47)	0.000
A-H-J	0.08	0.21	0.39	0.69	(0.00,0.49)	0.000
B-D-J	0.07	0.11	0.59	0.56	(0.00,0.29)	0.004
A-D-J	0.07	0.05	1.37	0.17	(0.00,0.16)	0.001
D-F-G	0.04	0.09	0.45	0.65	(0.00,0.21)	0.000
D-G-J	0.04	0.11	0.37	0.72	(0.00,0.25)	0.004
A-G-J	0.04	0.05	0.80	0.42	(0.00,0.13)	0.000
B-G-J	0.04	0.07	0.50	0.62	(0.00,0.17)	0.000
B-D-G	0.02	0.11	0.18	0.86	(0.00,0.23)	0.000
A-F-H	0.02	0.29	0.07	0.95	(0.00,0.58)	0.000
F-H-J	0.02	0.08	0.22	0.83	(0.00,0.17)	0.000
E-I-J	0.01	0.20	0.05	0.96	(0.00,0.39)	0.000

A = Mediterranean diet; **B** = Dietary Approaches to Stop Hypertension; **D** = Dietary guidelines-based diets; **E** = low GI / GL diet; **F** = plant-based diets; **G** = low fat diet; **H** = low carbohydrate high fat diet; **I** = high GI / GL diet; **J** = western habitual diet

Inconsistency was inferred if IF ≥ 0.7 and the lower limit of CI does not read zero and *p* value ≤ 0.05.

Abbreviations: IF, inconsistency factor

Supplementary Table S17. Loop-specific approach assessing inconsistency for total cholesterol

Loop	IF	Standard Error	z value	p value	95% CI	Loop-specific Heterogeneity (T^2)
A-D-G	0.75	0.22	3.41	0.00	(0.32,1.18)	0.000
D-F-G	0.70	0.22	3.13	0.00	(0.26,1.14)	0.000
A-D-J	0.68	0.38	1.77	0.08	(0.00,1.43)	0.099
A-F-H	0.41	0.81	0.51	0.61	(0.00,2.01)	0.000
E-I-J	0.39	0.53	0.74	0.46	(0.00,1.42)	0.000
F-H-J	0.36	0.29	1.23	0.22	(0.00,0.93)	0.007
B-D-J	0.33	0.40	0.83	0.40	(0.00,1.11)	0.065
A-D-F	0.32	0.61	0.52	0.60	(0.00,1.52)	0.000
B-D-G	0.32	0.37	0.87	0.38	(0.00,1.04)	0.000
F-G-J	0.30	0.15	2.04	0.04	(0.01,0.59)	0.010
A-F-G	0.27	0.60	0.45	0.65	(0.00,1.45)	0.000
D-F-J	0.26	0.31	0.83	0.41	(0.00,0.86)	0.047
B-G-J	0.13	0.36	0.36	0.72	(0.00,0.84)	0.026
A-H-J	0.11	0.68	0.16	0.88	(0.00,1.45)	0.109
A-F-J	0.10	0.69	0.15	0.88	(0.00,1.45)	0.054
D-G-J	0.08	0.42	0.20	0.84	(0.00,0.90)	0.074
A-G-J	0.03	0.19	0.15	0.88	(0.00,0.39)	0.042

A = Mediterranean diet; **B** = Dietary Approaches to Stop Hypertension; **D** = Dietary guidelines-based diets; **E** = low GI / GL diet; **F** = plant-based diets; **G** = low fat diet; **H** = low carbohydrate high fat diet; **I** = high GI / GL diet; **J** = western habitual diet

Inconsistency was inferred if IF ≥ 0.7 and the lower limit of CI does not read zero and *p* value ≤ 0.05.

Abbreviations: IF, inconsistency factor

Supplementary Table S18. Loop-specific approach assessing inconsistency for triglycerides

Loop	IF	Standard Error	z value	p value	95% CI	Loop-specific Heterogeneity (T^2)
B-G-J	0.40	0.23	1.73	0.08	(0.00,0.86)	0.000
A-D-G	0.39	0.08	4.89	0.00	(0.24,0.55)	0.000
B-D-G	0.30	0.25	1.19	0.24	(0.00,0.80)	0.000
A-D-J	0.27	0.10	2.77	0.01	(0.08,0.46)	0.005
D-F-J	0.21	0.11	1.86	0.06	(0.00,0.43)	0.001
A-F-G	0.17	0.22	0.78	0.43	(0.00,0.61)	0.000
D-G-J	0.14	0.15	0.99	0.32	(0.00,0.43)	0.009
F-G-J	0.14	0.06	2.49	0.01	(0.03,0.25)	0.000
A-F-H	0.13	0.35	0.38	0.71	(0.00,0.82)	0.000
F-H-J	0.13	0.19	0.68	0.50	(0.00,0.51)	0.000
A-D-F	0.11	0.24	0.47	0.64	(0.00,0.57)	0.000
D-F-G	0.11	0.12	0.96	0.34	(0.00,0.34)	0.000
A-H-J	0.07	0.24	0.31	0.76	(0.00,0.54)	0.000
E-I-J	0.05	0.39	0.13	0.90	(0.00,0.82)	0.000
A-F-J	0.03	0.22	0.12	0.91	(0.00,0.46)	0.000
A-G-J	0.01	0.06	0.16	0.87	(0.00,0.14)	0.000
B-D-J	0.00	0.19	0.01	0.99	(0.00,0.37)	0.013

A = Mediterranean diet; **B** = Dietary Approaches to Stop Hypertension; **D** = Dietary guidelines-based diets; **E** = low GI / GL diet; **F** = plant-based diets; **G** = low fat diet; **H** = low carbohydrate high fat diet; **I** = high GI / GL diet; **J** = western habitual diet

Inconsistency was inferred if IF ≥ 0.7 and the lower limit of CI does not read zero and *p* value ≤ 0.05.

Abbreviations: IF, inconsistency factor

Supplementary Table S19. Loop-specific approach assessing inconsistency for ApoB

Loop	IF	Standard Error	z value	p value	95% CI	Loop-specific Heterogeneity (T^2)
A-F-G	0.07	0.15	0.49	0.63	(0.00,0.36)	0.001
D-F-G	0.07	0.14	0.47	0.64	(0.00,0.34)	0.000
A-E-G	0.06	0.04	1.37	0.17	(0.00,0.14)	0.000
A-D-G	0.01	0.11	0.05	0.96	(0.00,0.21)	0.000

A = Mediterranean diet; **D** = plant-based diets; **E** = low fat diet; **F** = low carbohydrate high fat diet; **G** = western habitual diet

Inconsistency was inferred if IF ≥ 0.7 and the lower limit of CI does not read zero and *p* value ≤ 0.05.

Abbreviations: IF, inconsistency factor

Supplementary Table S20. Loop-specific approach assessing inconsistency for ApoA1

Loop	IF	Standard Error	z value	p value	95% CI	Loop-specific Heterogeneity (T^2)
A-F-G	0.28	0.17	1.60	0.11	(0.00,0.61)	0.000
D-F-G	0.14	0.18	0.77	0.44	(0.00,0.50)	0.003
A-D-G	0.13	0.15	0.89	0.37	(0.00,0.42)	0.001
A-E-G	0.13	0.06	2.29	0.02	(0.02,0.25)	0.000

A = Mediterranean diet; **D** = plant-based diets; **E** = low fat diet; **F** = low carbohydrate high fat diet; **G** = western habitual diet

Inconsistency was inferred if IF ≥ 0.7 and the lower limit of CI does not read zero and *p* value ≤ 0.05.

Abbreviations: IF, inconsistency factor

Supplementary Table S21. Loop-specific approach assessing inconsistency for glucose

Loop	IF	Standard Error	z value	p value	95% CI	Loop-specific Heterogeneity (T^2)
A-D-G	0.23	0.12	1.89	0.06	(0.00,0.46)	0.000
E-J-K	0.15	0.32	0.48	0.64	(0.00,0.77)	0.000
A-D-K	0.14	0.10	1.45	0.15	(0.00,0.33)	0.000
B-D-K	0.12	0.11	1.02	0.31	(0.00,0.34)	0.000
D-G-K	0.08	0.10	0.74	0.46	(0.00,0.27)	0.001
A-G-K	0.02	0.09	0.26	0.79	(0.00,0.19)	0.001
F-H-K	0.00	0.23	0.00	1.00	(0.00,0.45)	0.000

A = Mediterranean diet; **B** = Dietary Approaches to Stop Hypertension; **D** = Dietary guidelines-based diets; **E** = low GI / GL diet; **F** = plant-based diets; **G** = low fat diet; **H** = low carbohydrate high fat diet; **J** = high GI / GL diet; **K** = western habitual diet

Inconsistency was inferred if IF ≥ 0.7 and the lower limit of CI does not read zero and *p* value ≤ 0.05.

Abbreviations: IF, inconsistency factor

Supplementary Table S22. Loop-specific approach assessing inconsistency for insulin

Loop	IF	Standard Error	z value	p value	95% CI	Loop-specific Heterogeneity (T^2)
A-G-J	6.92	3.48	1.99	0.05	(0.10,13.74)	7.580
D-G-J	6.62	7.48	0.88	0.38	(0.00,21.28)	20.354
B-D-J	1.08	1.90	0.57	0.57	(0.00,4.81)	0.764
E-I-J	1.05	4.38	0.24	0.81	(0.00,9.62)	0.000

A = Mediterranean diet; **B** = Dietary Approaches to Stop Hypertension; **D** = Dietary guidelines-based diets; **E** = low GI / GL diet; **G** = low fat diet; **I** = high GI / GL diet; **J** = western habitual diet

Inconsistency was inferred if IF ≥ 0.7 and the lower limit of CI does not read zero and *p* value ≤ 0.05.

Abbreviations: IF, inconsistency factor

Supplementary Table S23. Loop-specific approach assessing inconsistency for HOMA-IR

Loop	IF	Standard Error	z value	p value	95% CI	Loop-specific Heterogeneity (T^2)
D-G-H	0.40	0.82	0.49	0.63	(0.00,2.01)	0.000
C-F-H	0.28	0.28	1.02	0.31	(0.00,0.83)	0.000
A-F-H	0.02	0.52	0.04	0.97	(0.00,1.04)	0.112

A = Mediterranean diet; **C** = Dietary guidelines-based diets; **D** = low GI / GL diet; **F** = low fat diet; **G** = high GI / GL diet; **H** = western habitual diet

Inconsistency was inferred if IF ≥ 0.7 and the lower limit of CI does not read zero and *p* value ≤ 0.05.

Abbreviations: IF, inconsistency factor

Supplementary Table S24. Loop-specific approach assessing inconsistency for hsCRP

Loop	IF	Standard Error	z value	p value	95% CI	Loop-specific Heterogeneity (T^2)
A-F-H	1.07	0.69	1.56	0.12	(0.00,2.42)	0.089
A-G-H	0.34	0.40	0.86	0.39	(0.00,1.11)	0.000
A-E-H	0.24	0.25	0.98	0.33	(0.00,0.72)	0.000
E-G-H	0.10	0.37	0.26	0.79	(0.00,0.82)	0.000
D-F-H	0.05	2.16	0.02	0.98	(0.00,4.29)	1.299
A-E-G	0.04	1.39	0.03	0.98	(0.00,2.77)	0.000

A = Mediterranean diet; **D** = Dietary guidelines-based diets; **E** = plant-based diets; **F** = low fat diet; **G** = low carbohydrate high fat diet; **H** = western habitual diet

Inconsistency was inferred if IF ≥ 0.7 and the lower limit of CI does not read zero and *p* value ≤ 0.05.

Abbreviations: IF, inconsistency factor

Supplementary Table S25. Side-splitting approach assessing inconsistency for LDL-c

Side	Direct		Indirect		Difference		
	Coef.	Std. Err.	Coef.	Std. Err.	Coef.	Std. Err.	P>z
A J	0.2240	0.0701	0.5303	0.1330	-0.3063	0.1500	**0.0410**
A D	0.3900	0.1882	0.0366	0.1022	0.3534	0.2142	0.0990
A F	0.0867	0.4448	0.0042	0.1021	0.0824	0.4561	0.8570
A G	0.0353	0.0839	0.0181	0.1590	0.0171	0.1796	0.9240
A H	0.5964	0.4012	0.3171	0.1805	0.2793	0.4449	0.5300
B J	0.2035	0.1328	0.3559	0.1873	-0.1524	0.2296	0.5070
B D	0.2200	0.2206	0.0189	0.1463	0.2011	0.2647	0.4470
B G	0.0000	0.2956	-0.0001	0.1390	0.0001	0.3266	1.0000
C D *	-0.1331	0.2193	-0.3288	360.3073	0.1957	360.3074	1.0000
D J	0.2833	0.0856	-0.1016	0.1321	0.3849	0.1587	**0.0150**
D F	-0.3402	0.2127	-0.0288	0.1208	-0.3113	0.2446	0.2030
D G	0.1161	0.2591	-0.1163	0.1093	0.2323	0.2812	0.4090
E J *	-0.1000	0.3989	0.4252	0.6565	-0.5252	0.8272	0.5250
F J	0.3385	0.0934	0.0821	0.1683	0.2564	0.1927	0.1830
F G	-0.1700	0.1892	0.0974	0.1186	-0.2674	0.2233	0.2310
F H	0.1891	0.1865	0.7333	0.2815	-0.5441	0.3385	0.1080
G J	0.2371	0.1046	0.2757	0.1157	-0.0387	0.1562	0.8050
H J	-0.1241	0.1921	0.0044	0.2671	-0.1285	0.3318	0.6990
I J *	0.1000	0.3347	-0.4252	0.7564	0.5252	0.8272	0.5250

A = Mediterranean diet; **B** = Dietary Approaches to Stop Hypertension; **C** = Paleo diet; **D** = Dietary guidelines-based diets; **E** = low GI / GL diet; **F** = plant-based diets; **G** = low fat diet; **H** = low carbohydrate high fat diet; **I** = high GI / GL diet; **J** = western habitual diet

*All the evidence about these contrasts comes from the trials which directly compare them

Supplementary Table S26. Side-splitting approach assessing inconsistency for HDL-c

Side	Direct		Indirect		Difference		
	Coef.	Std. Err.	Coef.	Std. Err.	Coef.	Std. Err.	P>z
A J	0.0251	0.0243	0.0285	0.0382	-0.0034	0.0455	0.9410
A D	-0.0800	0.0338	-0.0019	0.0325	-0.0781	0.0469	0.0960
A F	-0.1890	0.1982	-0.0687	0.0280	-0.1203	0.2002	0.5480
A G	-0.0169	0.0256	-0.1442	0.0394	0.1273	0.0453	**0.0050**
A H	0.0300	0.2032	0.1258	0.0550	-0.0958	0.2105	0.6490
B J	0.0756	0.0331	0.1462	0.0522	-0.0706	0.0619	0.2540
B D	0.1000	0.0695	0.0135	0.0381	0.0865	0.0793	0.2750
B G	0.0400	0.0691	0.0141	0.0399	0.0259	0.0798	0.7450
C D *	-0.1056	0.0693	-0.1147	135.1146	0.0091	135.1146	1.0000
D J	0.0416	0.0284	0.0974	0.0359	-0.0558	0.0461	0.2260
D F	0.0404	0.0659	-0.0560	0.0359	0.0964	0.0750	0.1990
D G	-0.0394	0.0787	-0.0095	0.0312	-0.0298	0.0846	0.7240
E J *	0.0500	0.1419	0.0357	0.2531	0.0143	0.2901	0.9610
F J	0.1342	0.0223	0.0002	0.0280	0.1340	0.0358	**0.0000**
F G	-0.0400	0.0173	0.0824	0.0316	-0.1224	0.0360	**0.0010**
F H	0.2064	0.0573	0.1443	0.0950	0.0621	0.1089	0.5680
G J	0.0638	0.0347	0.0863	0.0327	-0.0225	0.0478	0.6370
H J	-0.0815	0.0592	-0.1235	0.0971	0.0420	0.1143	0.7130
I J *	0.0200	0.1419	0.0343	0.2531	-0.0143	0.2901	0.9610

A = Mediterranean diet; **B** = Dietary Approaches to Stop Hypertension; **C** = Paleo diet; **D** = Dietary guidelines-based diets; **E** = low GI / GL diet; **F** = plant-based diets; **G** = low fat diet; **H** = low carbohydrate high fat diet; **I** = high GI / GL diet; **J** = western habitual diet

*All the evidence about these contrasts comes from the trials which directly compare them

Supplementary Table S27. Side-splitting approach assessing inconsistency for total cholesterol

Side	Direct		Indirect		Difference		
	Coef.	Std. Err.	Coef.	Std. Err.	Coef.	Std. Err.	P>z
A J	0.2332	0.0861	0.5915	0.1770	-0.3584	0.1963	0.0680
A D	0.5500	0.2360	-0.0523	0.1195	0.6023	0.2646	**0.0230**
A F	-0.1000	0.6496	-0.0587	0.1290	-0.0413	0.6622	0.9500
A G	-0.0588	0.1108	0.0075	0.1997	-0.0664	0.2282	0.7710
A H	0.5368	0.5605	0.3964	0.2292	0.1404	0.6097	0.8180
B J	0.2887	0.1641	0.5004	0.2356	-0.2117	0.2871	0.4610
B D	0.2700	0.2779	0.0547	0.1792	0.2153	0.3306	0.5150
B G	0.1000	0.3781	-0.0004	0.1730	0.1004	0.4158	0.8090
C D *	-0.1955	0.2539	-0.4597	385.8246	0.2642	385.8247	0.9990
D J	0.3537	0.0990	-0.1180	0.1698	0.4717	0.1975	**0.0170**
D F	-0.3301	0.2687	-0.0518	0.1516	-0.2783	0.3085	0.3670
D G	0.1328	0.3170	-0.1441	0.1350	0.2769	0.3443	0.4210
E J *	0.0000	0.4377	0.8047	0.7660	-0.8047	0.8226	0.3280
F J	0.4272	0.1190	0.1287	0.2180	0.2985	0.2489	0.2300
F G	-0.2200	0.2469	0.1047	0.1507	-0.3247	0.2893	0.2620
F H	0.2805	0.2491	0.8571	0.3489	-0.5766	0.4330	0.1830
G J	0.2901	0.1267	0.4123	0.1500	-0.1222	0.1964	0.5340
H J	-0.1779	0.2478	0.0044	0.3686	-0.1823	0.4578	0.6900
I J *	0.2000	0.4377	-0.6047	0.7660	0.8047	0.8226	0.3280

A = Mediterranean diet; **B** = Dietary Approaches to Stop Hypertension; **C** = Paleo diet; **D** = Dietary guidelines-based diets; **E** = low GI / GL diet; **F** = plant-based diets; **G** = low fat diet; **H** = low carbohydrate high fat diet; **I** = high GI / GL diet; **J** = western habitual diet

*All the evidence about these contrasts comes from the trials which directly compare them

Supplementary Table S28. Side-splitting approach assessing inconsistency for triglycerides

Side	Direct		Indirect		Difference		
	Coef.	Std. Err.	Coef.	Std. Err.	Coef.	Std. Err.	P>z
A J	0.0109	0.0390	0.1126	0.0729	-0.1017	0.0828	0.2200
A D	0.2600	0.0447	-0.0456	0.0457	0.3056	0.0640	**0.0000**
A F	0.1291	0.2326	0.0890	0.0548	0.0401	0.2390	0.8670
A G	-0.0115	0.0484	0.1842	0.0728	-0.1957	0.0880	**0.0260**
A H	-0.1200	0.2171	-0.1326	0.1280	0.0126	0.2520	0.9600
B J	0.0054	0.0794	0.0601	0.1194	-0.0547	0.1436	0.7030
B D	-0.0200	0.1266	0.0669	0.0859	-0.0869	0.1530	0.5700
B G	0.4000	0.2387	-0.0047	0.0785	0.4047	0.2513	0.1070
C D *	0.0106	0.0867	0.0401	130.5248	-0.0295	130.5248	1.0000
D J	0.0267	0.0474	-0.1207	0.0701	0.1474	0.0859	0.0860
D F	-0.0162	0.1228	0.0570	0.0656	-0.0733	0.1393	0.5990
D G	0.1198	0.1065	-0.0436	0.0590	0.1634	0.1218	0.1800
E J *	-0.2000	0.2870	-0.0997	0.5091	-0.1003	0.5844	0.8640
F J	-0.1029	0.0514	0.0592	0.0826	-0.1621	0.0975	0.0960
F G	0.0300	0.0869	-0.0883	0.0664	0.1183	0.1094	0.2790
F H	-0.1682	0.1532	-0.2758	0.1576	0.1076	0.2195	0.6240
G J	0.0232	0.0567	-0.0611	0.0658	0.0843	0.0882	0.3390
H J	0.2093	0.1370	0.0878	0.1733	0.1215	0.2218	0.5840
I J *	-0.2000	0.2870	-0.3003	0.5091	0.1003	0.5844	0.8640

A = Mediterranean diet; **B** = Dietary Approaches to Stop Hypertension; **C** = Paleo diet; **D** = Dietary guidelines-based diets; **E** = low GI / GL diet; **F** = plant-based diets; **G** = low fat diet; **H** = low carbohydrate high fat diet; **I** = high GI / GL diet; **J** = western habitual diet

*All the evidence about these contrasts comes from the trials which directly compare them

Supplementary Table S29. Side-splitting approach assessing inconsistency for ApoB

Side	Direct		Indirect		Difference		
	Coef.	Std. Err.	Coef.	Std. Err.	Coef.	Std. Err.	P>z
A G	0.0926	0.0212	0.0089	0.0700	0.0837	0.0725	0.2480
A D	0.0200	0.1045	0.0184	0.0373	0.0016	0.1110	0.9880
A E *	-0.0197	0.0156	0.1741	0.0756	-0.1939	0.0774	**0.0120**
A F	0.1200	0.1044	0.1727	0.0911	-0.0527	0.1385	0.7040
B C *	0.0200	0.0359	-0.1745	56.5251	0.1945	56.5251	0.9970
C G *	0.0873	0.0281	-0.0101	28.7800	0.0974	28.7800	0.9970
D G	0.0689	0.0296	0.0477	0.0987	0.0212	0.1031	0.8370
D F	0.1000	0.1045	0.1565	0.0937	-0.0565	0.1404	0.6870
E G	0.0758	0.0197	0.2294	0.0495	-0.1536	0.0534	**0.0040**
F G	-0.1000	0.0978	-0.0323	0.0922	-0.0677	0.1344	0.6150

A = Mediterranean diet; **B** = Dietary Approaches to Stop Hypertension; **C** = Dietary guidelines-based diets; **D** = plant-based diets; **E** = low fat diet; **F** = low carbohydrate high fat diet; **G** = western habitual diet

*All the evidence about these contrasts comes from the trials which directly compare them

Supplementary Table S30. Side-splitting approach assessing inconsistency for ApoA1

Side	Direct		Indirect		Difference		
	Coef.	Std. Err.	Coef.	Std. Err.	Coef.	Std. Err.	P>z
A G	0.0723	0.0565	0.0224	0.1293	0.0500	0.1412	0.7230
A D	-0.1800	0.1581	-0.0124	0.0822	-0.1676	0.1782	0.3470
A E *	0.0153	0.0462	-0.4116	0.3129	0.4269	0.3162	0.1770
A F	-0.0099	0.1544	0.2915	0.1273	-0.3013	0.2004	0.1330
B C *	0.0200	0.0895	-0.1480	81.0544	0.1680	81.0545	0.9980
C G *	0.0740	0.0524	-0.0099	41.2595	0.0839	41.2596	0.9980
D G	0.1038	0.0644	0.1628	0.1508	-0.0590	0.1640	0.7190
D F	0.1681	0.1576	0.2518	0.1376	-0.0837	0.2095	0.6900
E G	0.0881	0.0502	-0.2079	0.1535	0.2960	0.1617	0.0670
F G	-0.2000	0.1246	0.0193	0.1402	-0.2193	0.1876	0.2420

A = Mediterranean diet; **B** = Dietary Approaches to Stop Hypertension; **C** = Dietary guidelines-based diets; **D** = plant-based diets; **E** = low fat diet; **F** = low carbohydrate high fat diet; **G** = western habitual diet

*All the evidence about these contrasts comes from the trials which directly compare them

Supplementary Table S31. Side-splitting approach assessing inconsistency for glucose

Side	Direct		Indirect		Difference		
	Coef.	Std. Err.	Coef.	Std. Err.	Coef.	Std. Err.	P>z
A K	0.041479	0.036829	0.068578	0.077788	-0.0271	0.086329	0.754
A D	0.22	0.08915	0.036603	0.047266	0.183397	0.100904	0.069
A G	-0.0115	0.053182	0.035421	0.081773	-0.04692	0.096758	0.628
B K	0.14	0.087099	0.02303	0.086344	0.11697	0.122644	0.34
B D	0.06	0.078585	0.17697	0.09416	-0.11697	0.122645	0.34
C D *	0.02003	0.146061	0.018839	293.354	0.001191	293.3541	1
D K	-0.01888	0.041128	-0.04813	0.073962	0.029248	0.086287	0.735
D G	9.22E-05	0.076668	-0.11448	0.060746	0.114575	0.097841	0.242
E K *	-4.19E-06	0.224268	0.302486	0.322528	-0.30249	0.306661	0.324
F K *	0.072748	0.116610	0.194866	0.408797	-0.122118	0.437995	0.780
F H	-0.107579	0.158313	-0.064929	0.209202	-0.042651	0.264101	0.872
G K	0.038240	0.060898	0.053239	0.073397	-0.014999	0.097454	0.878
H K *	0.160569	0.110343	0.396425	0.417943	-0.235856	0.434437	0.587
J K *	0.200002	0.224268	-0.102494	0.322528	0.302496	0.306661	0.324

A = Mediterranean diet; **B** = Dietary Approaches to Stop Hypertension; **C** = Paleo diet; **D** = Dietary guidelines-based diets; **E** = low GI / GL diet; **F** = plant-based diets; **G** = low fat diet; **H** = low carbohydrate high fat diet; **J** = high GI / GL diet; **K** = western habitual diet

*All the evidence about these contrasts comes from the trials which directly compare them

Supplementary Table S32. Side-splitting approach assessing inconsistency for insulin

Side	Direct		Indirect		Difference		
	Coef.	Std. Err.	Coef.	Std. Err.	Coef.	Std. Err.	P>z
A J	1.911093	1.320989	2.152888	3.621182	-0.2418	3.857038	0.95
A G	-0.24218	1.526745	-5.22363	3.235967	4.981458	3.584061	0.165
B J	1	3.823079	2.395222	3.978639	-1.39522	5.517745	0.8
B D	0.599999	3.681548	-0.79529	4.109965	1.39529	5.517755	0.8
C D *	1.209583	2.592567	-3.47835	1200.877	4.687932	1200.88	0.997
D J	1.576608	1.544956	2.568696	4.318496	-0.99209	4.597195	0.829
D G	1.220286	3.617674	-2.55051	2.402839	3.770791	4.342683	0.385
E J *	-2.33999	5.130763	0.503361	8.540391	-2.84335	10.60975	0.789
G J	6.7953	2.057686	-0.27954	1.959337	7.074842	2.851511	**0.013**
I J *	-1.49996	4.307423	-4.34025	9.809142	2.84029	10.60868	0.789

A = Mediterranean diet; **B** = Dietary Approaches to Stop Hypertension; **C** = Paleo diet; **D** = Dietary guidelines-based diets; **E** = low GI / GL diet; **G** = low fat diet; **I** = high GI / GL diet; **J** = western habitual diet

*All the evidence about these contrasts comes from the trials which directly compare them

Supplementary Table S33. Side-splitting approach assessing inconsistency for HOMA-IR

Side	Direct		Indirect		Difference		
	Coef.	Std. Err.	Coef.	Std. Err.	Coef.	Std. Err.	P>z
A H	0.2373	0.2488	0.2226	0.3231	0.0147	0.3964	0.9700
A F	0.0583	0.1959	0.0176	0.3365	0.0407	0.3886	0.9170
B C *	0.6000	0.3549	-0.7487	678.5767	1.3487	678.5769	0.9980
C H *	0.3551	0.1456	0.3342	0.7034	0.0209	0.7305	0.9770
C F	0.2349	0.2383	0.0477	0.3299	0.1872	0.4049	0.6440
D H *	0.1300	0.5363	-0.6700	1.1169	0.8000	1.1152	0.4730
F H	0.2875	0.2127	-0.0310	0.3071	0.3185	0.3842	0.4070
G H *	-0.0900	0.5898	0.7100	1.0327	-0.8000	1.1152	0.4730

A = Mediterranean diet; **B** = Paleo diet; **C** = Dietary guidelines-based diets; **D** = low GI / GL diet; **F** = low fat diet; **G** = high GI / GL diet; **H** = western habitual diet

*All the evidence about these contrasts comes from the trials which directly compare them

Supplementary Table S34. Side-splitting approach assessing inconsistency for hsCRP

Side	Direct		Indirect		Difference		
	Coef.	Std. Err.	Coef.	Std. Err.	Coef.	Std. Err.	P>z
A H	0.224738	0.260401	0.309875	0.440396	-0.08514	0.510968	0.868
A E	-0.12	0.51015	0.334391	0.499175	-0.45439	0.713743	0.524
A F	0.696086	0.345232	-0.15765	0.599999	0.853736	0.68219	0.211
A G	0.02	0.500921	0.558915	0.545613	-0.53892	0.740686	0.467
B D *	0.07	0.500561	-1.01858	641.2795	1.088575	641.2797	0.999
C D *	0.229324	0.512194	-1.04088	837.1506	1.270206	837.1508	0.999
D H *	0.580334	0.297528	-1.12711	1.331612	1.707447	1.364362	0.211
D F	0.402453	0.606844	1.002122	0.526682	-0.59967	0.801387	0.454
E H	-0.00249	0.452394	0.312877	0.519266	-0.31536	0.688293	0.647
E G	0.150922	0.489895	0.170335	0.748945	-0.01941	0.895227	0.983
F H	0.504001	0.641603	-0.53794	0.391419	1.041936	0.759914	0.17
G H	-0.23024	0.521269	0.170105	0.501619	-0.40035	0.723555	0.58

A = Mediterranean diet; **B** = Dietary Approaches to Stop Hypertension; **C** = Paleo diet; **D** = Dietary guidelines-based diets; **E** = plant-based diets; **F** = low fat diet; **G** = low carbohydrate high fat diet; **H** = western habitual diet

*All the evidence about these contrasts comes from the trials which directly compare them

Supplementary Table S35. Side-splitting approach assessing inconsistency for interleukin-6

Side	Direct		Indirect		Difference		
	Coef.	Std. Err.	Coef.	Std. Err.	Coef.	Std. Err.	P>z
A E *	0.125326	0.23787	0.12018	390.0671	0.005146	390.0672	1
A D *	0.35	0.330977	0.377315	744.759	-0.02732	744.759	1
B C *	1.64	0.96285	-0.01215	2039.884	1.652146	2039.885	0.999
C E *	-0.06188	0.121346	-0.92217	924.4848	0.860285	924.4848	0.999

A = Mediterranean diet; **B** = Paleo diet; **C** = Dietary guidelines-based diets; **D** = low fat diet; **E** = western habitual diet

*All the evidence about these contrasts comes from the trials which directly compare them

Supplementary Table S36. League table of sensitivity analysis excluding high risk of bias studies for LDL-c[1]

Mediterranean									
-0.01 (-0.26, 0.24)	*DASH*								
-0.22 (-0.70, 0.25)	-0.21 (-0.71, 0.28)	*Paleo*							
-0.09 (-0.28, 0.10)	-0.08 (-0.32, 0.16)	0.13 (-0.30, 0.57)	*DG-based*						
-0.20 (-0.83, 0.43)	-0.19 (-0.84, 0.46)	0.02 (-0.74, 0.79)	-0.11 (-0.74, 0.52)	*Low GI/GL*					
0.01 (-0.19, 0.21)	0.02 (-0.24, 0.29)	0.24 (-0.24, 0.72)	0.10 (-0.11, 0.32)	0.22 (-0.42, 0.85)	*Plant-based*				
-0.03 (-0.19, 0.13)	-0.02 (-0.27, 0.24)	0.20 (-0.28, 0.68)	0.07 (-0.14, 0.27)	0.18 (-0.46, 0.81)	-0.04 (-0.25, 0.17)	*Low fat*			
**-0.35 (-0.67, -0.02)**	-0.34 (-0.71, 0.03)	-0.12 (-0.67, 0.43)	-0.25 (-0.59, 0.08)	-0.14 (-0.83, 0.54)	**-0.36 (-0.67, -0.05)**	-0.32 (-0.66, 0.02)	*Low CHO high fat*		
-0.25 (-0.87, 0.37)	-0.24 (-0.88, 0.40)	-0.02 (-0.78, 0.74)	-0.16 (-0.78, 0.47)	-0.05 (-0.25, 0.16)	-0.26 (-0.89, 0.36)	-0.22 (-0.85, 0.40)	0.10 (-0.58, 0.77)	*High GI/GL*	
**-0.26 (-0.40, -0.13)**	**-0.25 (-0.47, -0.04)**	-0.04 (-0.50, 0.42)	**-0.17 (-0.32, -0.01)**	-0.06 (-0.67, 0.55)	**-0.27 (-0.44, -0.11)**	**-0.24 (-0.40, -0.07)**	0.09 (-0.22, 0.39)	-0.01 (-0.62, 0.59)	*Western habitual*

[1]The values correspond to the mean difference (95% CI) in LDL-c (mmol/L) between the column dietary pattern and the row dietary pattern; column minus row. Values in bold are effect sizes that are statistically significant at level of 0.05.

Abbreviations: CHO, carbohydrate; DASH, Dietary Approaches to Stop Hypertension; DG, dietary guidelines; GI/GL, glycemic index/glycemic load

Supplementary Table S37. League table of sensitivity analysis excluding high risk of bias studies for HDL-c[1]

Mediterranean									
**0.08 (0.01, 0.15)**	*DASH*								
-0.06 (-0.21, 0.08)	-0.14 (-0.29, 0.01)	*Paleo*							
0.04 (-0.01, 0.09)	-0.04 (-0.10, 0.03)	0.11 (-0.03, 0.24)	*DG-based*						
0.03 (-0.22, 0.28)	-0.05 (-0.30, 0.20)	0.09 (-0.19, 0.38)	-0.01 (-0.26, 0.24)	*Low GI/GL*					
**0.08 (0.02, 0.14)**	0.00 (-0.07, 0.07)	0.14 (-0.01, 0.29)	0.04 (-0.02, 0.10)	0.05 (-0.20, 0.30)	*Plant-based*				
0.05 (-0.01, 0.11)	-0.03 (-0.10, 0.05)	0.12 (-0.03, 0.27)	0.01 (-0.05, 0.07)	0.02 (-0.23, 0.27)	-0.03 (-0.09, 0.03)	*Low fat*			
**-0.11 (-0.22, -0.01)**	**-0.19 (-0.30, -0.08)**	-0.05 (-0.22, 0.13)	**-0.15 (-0.26, -0.04)**	-0.14 (-0.40, 0.12)	**-0.19 (-0.29, -0.09)**	**-0.16 (-0.27, -0.05)**	*Low CHO high fat*		
0.01 (-0.24, 0.25)	-0.07 (-0.32, 0.18)	0.07 (-0.21, 0.35)	-0.04 (-0.28, 0.21)	-0.02 (-0.08, 0.04)	-0.07 (-0.32, 0.18)	-0.05 (-0.29, 0.20)	0.12 (-0.15, 0.38)	*High GI/GL*	
-0.02 (-0.06, 0.03)	**-0.09 (-0.15, -0.04)**	0.05 (-0.10, 0.19)	**-0.06 (-0.10, -0.01)**	-0.05 (-0.29, 0.20)	**-0.10 (-0.14, -0.05)**	**-0.07 (-0.12, -0.02)**	0.09 (-0.01, 0.19)	-0.02 (-0.27, 0.22)	*Western habitual*

[1]The values correspond to the mean difference (95% CI) in HDL-c (mmol/L) between the column dietary pattern and the row dietary pattern; column minus row. Values in bold are effect sizes that are statistically significant at level of 0.05.

Abbreviations: CHO, carbohydrate; DASH, Dietary Approaches to Stop Hypertension; DG, dietary guidelines; GI/GL, glycemic index/glycemic load

Supplementary Table S38. League table of sensitivity analysis excluding high risk of bias studies for total cholesterol[1]

Mediterranean									
0.08<br>(-0.23, 0.39)	*DASH*								
-0.23<br>(-0.79, 0.33)	-0.31<br>(-0.90, 0.28)	*Paleo*							
-0.04<br>(-0.27, 0.20)	-0.12<br>(-0.42, 0.18)	0.20<br>(-0.31, 0.70)	*DG-based*						
-0.10<br>(-0.92, 0.71)	-0.19<br>(-1.03, 0.66)	0.13<br>(-0.84, 1.09)	-0.07<br>(-0.89, 0.75)	*Low GI/GL*					
0.08<br>(-0.18, 0.34)	-0.00<br>(-0.34, 0.34)	0.31<br>(-0.26, 0.88)	0.12<br>(-0.15, 0.38)	0.19<br>(-0.64, 1.01)	*Plant-based*				
0.04<br>(-0.17, 0.25)	-0.04<br>(-0.36, 0.28)	0.27<br>(-0.30, 0.84)	0.08<br>(-0.18, 0.34)	0.14<br>(-0.68, 0.97)	-0.04<br>(-0.31, 0.23)	*Low fat*			
-0.40<br>(-0.82, 0.02)	**-0.48<br>(-0.95, -0.00)**	-0.16<br>(-0.83, 0.50)	-0.36<br>(-0.79, 0.07)	-0.29<br>(-1.18, 0.60)	**-0.48<br>(-0.88, -0.08)**	-0.44<br>(-0.87, 0.00)	*Low CHO high fat*		
-0.24<br>(-1.06, 0.58)	-0.32<br>(-1.16, 0.52)	-0.01<br>(-0.97, 0.95)	-0.20<br>(-1.02, 0.62)	-0.14<br>(-0.44, 0.17)	-0.32<br>(-1.15, 0.50)	-0.28<br>(-1.10, 0.55)	0.16<br>(-0.73, 1.05)	*High GI/GL*	
**-0.27<br>(-0.44, -0.10)**	**-0.35<br>(-0.62, -0.08)**	-0.04<br>(-0.58, 0.50)	**-0.24<br>(-0.42, -0.05)**	-0.17<br>(-0.97, 0.63)	**-0.35<br>(-0.56, -0.14)**	**-0.31<br>(-0.52, -0.10)**	0.12<br>(-0.27, 0.52)	-0.03<br>(-0.83, 0.77)	*Western habitual*

[1]The values correspond to the mean difference (95% CI) in total cholesterol (mmol/L) between the column dietary pattern and the row dietary pattern; column minus row. Values in bold are effect sizes that are statistically significant at level of 0.05.

Abbreviations: CHO, carbohydrate; DASH, Dietary Approaches to Stop Hypertension; DG, dietary guidelines; GI/GL, glycemic index/glycemic load

Supplementary Table S39. League table of sensitivity analysis excluding high risk of bias studies for triglycerides[1]

Mediterranean									
-0.02 (-0.16, 0.13)	*DASH*								
-0.04 (-0.24, 0.16)	-0.03 (-0.25, 0.20)	*Paleo*							
-0.05 (-0.15, 0.04)	-0.04 (-0.18, 0.10)	-0.01 (-0.18, 0.16)	*DG-based*						
-0.22 (-0.71, 0.28)	-0.20 (-0.71, 0.31)	-0.17 (-0.70, 0.36)	-0.16 (-0.66, 0.34)	*Low GI/GL*					
-0.10 (-0.21, 0.01)	-0.08 (-0.24, 0.07)	-0.05 (-0.26, 0.15)	-0.04 (-0.16, 0.07)	0.12 (-0.38, 0.62)	*Plant-based*				
-0.06 (-0.16, 0.04)	-0.04 (-0.19, 0.11)	-0.01 (-0.22, 0.19)	-0.00 (-0.11, 0.11)	0.16 (-0.34, 0.66)	0.04 (-0.07, 0.15)	*Low fat*			
0.12 (-0.09, 0.34)	0.14 (-0.11, 0.39)	0.17 (-0.12, 0.45)	0.18 (-0.05, 0.40)	0.34 (-0.20, 0.87)	**0.22 (0.01, 0.44)**	0.18 (-0.05, 0.41)	*Low CHO high fat*		
-0.26 (-0.76, 0.23)	-0.25 (-0.76, 0.26)	-0.22 (-0.75, 0.31)	-0.21 (-0.71, 0.29)	-0.05 (-0.16, 0.06)	-0.17 (-0.67, 0.33)	-0.21 (-0.71, 0.29)	-0.39 (-0.92, 0.15)	*High GI/GL*	
-0.04 (-0.12, 0.04)	-0.02 (-0.15, 0.11)	0.00 (-0.19, 0.20)	0.02 (-0.07, 0.10)	0.18 (-0.32, 0.67)	0.06 (-0.03, 0.15)	0.02 (-0.08, 0.11)	-0.16 (-0.37, 0.05)	0.22 (-0.27, 0.72)	*Western habitual*

[1]The values correspond to the mean difference (95% CI) in triglycerides (mmol/L) between the column dietary pattern and the row dietary pattern; column minus row. Values in bold are effect sizes that are statistically significant at level of 0.05.

Abbreviations: CHO, carbohydrate; DASH, Dietary Approaches to Stop Hypertension; DG, dietary guidelines; GI/GL, glycemic index/glycemic load

Supplementary Table S40. League table of sensitivity analysis excluding high risk of bias studies for ApoB[1]

Mediterranean						
0.04<br>(-0.07, 0.15)	*DASH*					
0.02<br>(-0.07, 0.11)	-0.02<br>(-0.09, 0.05)	*DG-based*				
-0.03<br>(-0.10, 0.05)	-0.07<br>(-0.18, 0.04)	-0.05<br>(-0.14, 0.04)	*Plant-based*			
0.02<br>(-0.03, 0.07)	-0.02<br>(-0.14, 0.09)	-0.00<br>(-0.09, 0.09)	0.05<br>(-0.03, 0.13)	*Low fat*		
**-0.16<br>(-0.29, -0.02)**	**-0.20<br>(-0.36, -0.03)**	**-0.18<br>(-0.33, -0.03)**	-0.13<br>(-0.27, 0.01)	**-0.18<br>(-0.32, -0.03)**	*Low CHO high fat*	
**-0.10<br>(-0.15, -0.05)**	**-0.14<br>(-0.24, -0.04)**	**-0.12<br>(-0.19, -0.05)**	**-0.07<br>(-0.12, -0.01)**	**-0.12<br>(-0.17, -0.06)**	0.06<br>(-0.07, 0.19)	*Western habitual*

[1]The values correspond to the mean difference (95% CI) in ApoB (g/L) between the column dietary pattern and the row dietary pattern; column minus row. Values in bold are effect sizes that are statistically significant at level of 0.05.

Abbreviations: CHO, carbohydrate; DASH, Dietary Approaches to Stop Hypertension; DG, dietary guidelines

Supplementary Table S41. League table of sensitivity analysis excluding high risk of bias studies for ApoA1[1]

Mediterranean						
0.09 (-0.13, 0.31)	*DASH*					
0.07 (-0.08, 0.22)	-0.02 (-0.17, 0.13)	*DG-based*				
0.05 (-0.09, 0.19)	-0.04 (-0.25, 0.17)	-0.02 (-0.17, 0.13)	*Plant-based*			
-0.03 (-0.13, 0.06)	-0.12 (-0.34, 0.09)	-0.10 (-0.26, 0.05)	-0.08 (-0.23, 0.06)	*Low fat*		
-0.17 (-0.36, 0.02)	**-0.26 (-0.51, -0.01)**	**-0.24 (-0.45, -0.04)**	**-0.22 (-0.41, -0.03)**	-0.14 (-0.33, 0.06)	*Low CHO high fat*	
-0.07 (-0.17, 0.04)	-0.16 (-0.34, 0.03)	**-0.14 (-0.25, -0.02)**	**-0.11 (-0.21, -0.01)**	-0.03 (-0.14, 0.08)	0.11 (-0.07, 0.28)	*Western habitual*

[1]The values correspond to the mean difference (95% CI) in ApoA1 (g/L) between the column dietary pattern and the row dietary pattern; column minus row. Values in bold are effect sizes that are statistically significant at level of 0.05.

Abbreviations: CHO, carbohydrate; DASH, Dietary Approaches to Stop Hypertension; DG, dietary guidelines

Supplementary Table S42. League table of sensitivity analysis excluding high risk of bias studies for glucose[1]

Mediterranean										
0.05 (-0.10, 0.19)	*DASH*									
-0.04 (-0.34, 0.26)	-0.09 (-0.40, 0.23)	*Paleo*								
-0.06 (-0.15, 0.03)	-0.11 (-0.23, 0.02)	-0.02 (-0.31, 0.27)	*DG-based*							
0.03 (-0.40, 0.46)	-0.02 (-0.46, 0.42)	0.07 (-0.45, 0.58)	0.09 (-0.34, 0.52)	*Low GI/GL*						
0.05 (-0.18, 0.28)	0.00 (-0.25, 0.25)	0.09 (-0.28, 0.45)	0.11 (-0.12, 0.33)	0.02 (-0.45, 0.49)	*Plant-based*					
0.03 (-0.08, 0.14)	-0.01 (-0.18, 0.15)	0.07 (-0.24, 0.38)	0.09 (-0.02, 0.21)	0.00 (-0.43, 0.44)	-0.02 (-0.26, 0.23)	*Low fat*				
0.14 (-0.08, 0.36)	0.09 (-0.15, 0.34)	0.18 (-0.19, 0.54)	0.20 (-0.02, 0.42)	0.11 (-0.36, 0.58)	0.09 (-0.16, 0.34)	0.11 (-0.13, 0.35)	*Low CHO high fat*			
0.04 (-0.13, 0.22)	-0.00 (-0.21, 0.20)	0.08 (-0.26, 0.42)	0.10 (-0.07, 0.28)	0.02 (-0.44, 0.47)	-0.00 (-0.27, 0.26)	0.01 (-0.19, 0.21)	-0.10 (-0.36, 0.17)	*Mexican*		
0.10 (-0.33, 0.53)	0.05 (-0.39, 0.49)	0.14 (-0.38, 0.66)	0.16 (-0.27, 0.59)	0.07 (-0.04, 0.18)	0.05 (-0.42, 0.52)	0.07 (-0.37, 0.50)	-0.04 (-0.51, 0.43)	0.06 (-0.40, 0.51)	*High GI/GL*	
-0.04 (-0.11, 0.04)	-0.08 (-0.21, 0.04)	0.00 (-0.29, 0.30)	0.02 (-0.05, 0.09)	-0.06 (-0.49, 0.36)	-0.08 (-0.30, 0.13)	-0.07 (-0.18, 0.05)	-0.18 (-0.39, 0.03)	-0.08 (-0.24, 0.08)	-0.14 (-0.56, 0.29)	*Western habitual*

[1]The values correspond to the mean difference (95% CI) in glucose (mmol/L) between the column dietary pattern and the row dietary pattern; column minus row.

Abbreviations: CHO, carbohydrate; DASH, Dietary Approaches to Stop Hypertension; DG, dietary guidelines; GI/GL, glycemic index/glycemic load

Supplementary Table S43. League table of sensitivity analysis excluding high risk of bias studies for insulin[1]

Mediterranean									
**1.76 (0.40, 3.12)**	*DASH*								
**2.26 (0.77, 3.74)**	0.50 (-1.13, 2.13)	*Paleo*							
**1.33 (0.43, 2.23)**	-0.43 (-1.52, 0.67)	-0.92 (-2.11, 0.26)	*DG-based*						
-1.39 (-5.97, 3.19)	-3.15 (-7.83, 1.52)	-3.65 (-8.38, 1.08)	-2.72 (-7.30, 1.85)	*Low GI/GL*					
-1.24 (-4.64, 2.15)	-3.00 (-6.53, 0.52)	-3.50 (-7.09, 0.09)	-2.58 (-5.96, 0.81)	0.15 (-5.45, 5.75)	*Plant-based*				
0.19 (-0.43, 0.81)	**-1.57 (-2.95, -0.19)**	**-2.07 (-3.56, -0.58)**	**-1.14 (-2.05, -0.24)**	1.58 (-3.02, 6.18)	1.43 (-1.99, 4.85)	*Low fat*			
0.65 (-3.06, 4.36)	-1.11 (-4.94, 2.73)	-1.60 (-5.50, 2.29)	-0.68 (-4.39, 3.02)	2.04 (-3.76, 7.84)	1.90 (-1.26, 5.05)	0.46 (-3.27, 4.20)	*Low CHO high fat*		
-1.59 (-6.12, 2.95)	-3.34 (-7.98, 1.29)	-3.84 (-8.53, 0.85)	-2.92 (-7.45, 1.62)	-0.19 (-0.96, 0.57)	-0.34 (-5.91, 5.23)	-1.77 (-6.33, 2.78)	-2.24 (-8.00, 3.53)	*High GI/GL*	
0.10 (-0.63, 0.84)	**-1.65 (-2.87, -0.44)**	**-2.15 (-3.54, -0.76)**	**-1.23 (-1.94, -0.52)**	1.50 (-3.02, 6.01)	1.35 (-1.96, 4.66)	-0.08 (-0.93, 0.76)	-0.55 (-4.18, 3.09)	1.69 (-2.79, 6.17)	*Western habitual*

[1]The values correspond to the mean difference (95% CI) in insulin (mU/L) between the column dietary pattern and the row dietary pattern; column minus row. Values in bold are effect sizes that are statistically significant at level of 0.05.

Abbreviations: CHO, carbohydrate; DASH, Dietary Approaches to Stop Hypertension; DG, dietary guidelines; GI/GL, glycemic index/glycemic load

Supplementary Table S44. League table of sensitivity analysis excluding high risk of bias studies for hsCRP[1]

Mediterranean							
**0.81 (0.10, 1.52)**	*DASH*						
**0.96 (0.00, 1.91)**	0.14 (-0.78, 1.07)	*Paleo*					
**0.74 (0.21, 1.27)**	-0.07 (-0.54, 0.40)	-0.21 (-1.01, 0.58)	*DG-based*				
-0.05 (-0.27, 0.17)	**-0.86 (-1.55, -0.17)**	**-1.01 (-1.95, -0.07)**	**-0.79 (-1.29, -0.29)**	*Plant-based*			
0.17 (-0.39, 0.73)	-0.64 (-1.42, 0.14)	-0.79 (-1.80, 0.22)	-0.57 (-1.19, 0.05)	0.22 (-0.35, 0.79)	*Low fat*		
-0.15 (-0.47, 0.18)	**-0.96 (-1.71, -0.21)**	**-1.10 (-2.09, -0.12)**	**-0.89 (-1.47, -0.31)**	-0.10 (-0.41, 0.21)	-0.32 (-0.94, 0.31)	*Low CHO high fat*	
-0.10 (-0.32, 0.11)	**-0.92 (-1.60, -0.23)**	**-1.06 (-2.00, -0.12)**	**-0.85 (-1.34, -0.35)**	-0.05 (-0.11, 0.00)	-0.27 (-0.84, 0.29)	0.04 (-0.26, 0.35)	*Western habitual*

[1]The values correspond to the mean difference (95% CI) in hsCRP (mg/L) between the column dietary pattern and the row dietary pattern; column minus row. Values in bold are effect sizes that are statistically significant at level of 0.05.

Abbreviations: CHO, carbohydrate; DASH, Dietary Approaches to Stop Hypertension; DG, dietary guidelines; hsCRP, high sensitivity C-reactive protein

Supplementary Table S45. League table of sensitivity analysis excluding high risk of bias studies for interleukin-6[1]

Mediterranean			
1.51<br>(-0.47, 3.50)	*Paleo*		
-0.13<br>(-0.73, 0.48)	-1.64<br>(-3.53, 0.25)	*DG-based*	
-0.13<br>(-0.59, 0.34)	-1.64<br>(-3.57, 0.29)	0.00<br>(-0.39, 0.39)	*Western habitual*

[1]The values correspond to the mean difference (95% CI) in interleukin-6 (pg/mL) between the column dietary pattern and the row dietary pattern; column minus row. Values in bold are effect sizes that are statistically significant at level of 0.05.

Abbreviations: DG, dietary guidelines

Supplementary Table S46. League table of sensitivity analysis excluding studies with intervention duration longer than 52 weeks for LDL-c[1]

Mediterranean									
0.02 (-0.22, 0.26)	*DASH*								
-0.20 (-0.85, 0.45)	-0.21 (-0.88, 0.45)	*Paleo*							
-0.03 (-0.23, 0.17)	-0.04 (-0.28, 0.19)	0.17 (-0.45, 0.79)	*DG-based*						
-0.19 (-0.81, 0.43)	-0.21 (-0.85, 0.43)	0.01 (-0.87, 0.89)	-0.16 (-0.79, 0.46)	*Low GI/GL*					
0.03 (-0.16, 0.23)	0.01 (-0.24, 0.27)	0.23 (-0.43, 0.88)	0.06 (-0.15, 0.27)	0.22 (-0.40, 0.85)	*Plant-based*				
0.01 (-0.14, 0.15)	-0.01 (-0.25, 0.23)	0.20 (-0.45, 0.86)	0.03 (-0.17, 0.24)	0.20 (-0.42, 0.82)	-0.03 (-0.22, 0.17)	*Low fat*			
**-0.33 (-0.65, -0.01)**	-0.35 (-0.71, 0.02)	-0.13 (-0.84, 0.57)	-0.30 (-0.64, 0.03)	-0.14 (-0.81, 0.54)	**-0.36 (-0.66, -0.06)**	**-0.34 (-0.66, -0.01)**	*Low CHO high fat*		
-0.23 (-0.84, 0.37)	-0.25 (-0.88, 0.38)	-0.04 (-0.91, 0.84)	-0.21 (-0.82, 0.41)	-0.04 (-0.25, 0.16)	-0.27 (-0.88, 0.35)	-0.24 (-0.85, 0.37)	0.09 (-0.57, 0.76)	*High GI/GL*	
**-0.25 (-0.38, -0.12)**	**-0.27 (-0.47, -0.06)**	-0.05 (-0.69, 0.59)	**-0.22 (-0.38, -0.06)**	-0.06 (-0.66, 0.55)	**-0.28 (-0.44, -0.12)**	**-0.26 (-0.41, -0.11)**	0.08 (-0.22, 0.38)	-0.02 (-0.61, 0.58)	*Western habitual*

[1]The values correspond to the mean difference (95% CI) in LDL-c (mmol/L) between the column dietary pattern and the row dietary pattern; column minus row. Values in bold are effect sizes that are statistically significant at level of 0.05.

Abbreviations: CHO, carbohydrate; DASH, Dietary Approaches to Stop Hypertension; DG, dietary guidelines; GI/GL, glycemic index/glycemic load

Supplementary Table S47. League table of sensitivity analysis excluding studies with intervention duration longer than 52 weeks for HDL-c[1]

Mediterranean									
0.05<br>(-0.01, 0.12)	*DASH*								
0.01<br>(-0.20, 0.22)	-0.04<br>(-0.25, 0.17)	*Paleo*							
0.00<br>(-0.06, 0.07)	-0.05<br>(-0.12, 0.02)	-0.01<br>(-0.21, 0.19)	*DG-based*						
0.01<br>(-0.24, 0.25)	-0.05<br>(-0.29, 0.20)	-0.01<br>(-0.32, 0.31)	0.00<br>(-0.24, 0.25)	*Low GI/GL*					
0.05<br>(-0.00, 0.11)	0.00<br>(-0.06, 0.07)	0.04<br>(-0.17, 0.25)	0.05<br>(-0.01, 0.11)	0.05<br>(-0.20, 0.29)	*Plant-based*				
0.04<br>(-0.00, 0.09)	-0.01<br>(-0.08, 0.06)	0.03<br>(-0.18, 0.24)	0.04<br>(-0.02, 0.11)	0.04<br>(-0.21, 0.28)	-0.01<br>(-0.07, 0.05)	*Low fat*			
**-0.14**<br>**(-0.24, -0.03)**	**-0.19**<br>**(-0.30, -0.08)**	-0.15<br>(-0.37, 0.08)	**-0.14**<br>**(-0.24, -0.03)**	-0.14<br>(-0.40, 0.12)	**-0.19**<br>**(-0.28, -0.09)**	**-0.18**<br>**(-0.28, -0.07)**	*Low CHO high fat*		
-0.02<br>(-0.26, 0.23)	-0.07<br>(-0.32, 0.18)	-0.03<br>(-0.35, 0.29)	-0.02<br>(-0.26, 0.23)	-0.02<br>(-0.08, 0.03)	-0.07<br>(-0.31, 0.17)	-0.06<br>(-0.31, 0.18)	0.12<br>(-0.14, 0.38)	*High GI/GL*	
-0.04<br>(-0.08, 0.00)	**-0.09**<br>**(-0.15, -0.04)**	-0.05<br>(-0.26, 0.15)	-0.04<br>(-0.09, 0.01)	-0.05<br>(-0.29, 0.19)	**-0.09**<br>**(-0.14, -0.05)**	**-0.09**<br>**(-0.13, -0.04)**	0.09<br>(-0.00, 0.19)	-0.02<br>(-0.26, 0.22)	*Western habitual*

[1]The values correspond to the mean difference (95% CI) in HDL-c (mmol/L) between the column dietary pattern and the row dietary pattern; column minus row. Values in bold are effect sizes that are statistically significant at level of 0.05.

Abbreviations: CHO, carbohydrate; DASH, Dietary Approaches to Stop Hypertension; DG, dietary guidelines; GI/GL, glycemic index/glycemic load

Supplementary Table S48. League table of sensitivity analysis excluding studies with intervention duration longer than 52 weeks for total cholesterol[1]

Mediterranean									
0.12 (-0.17, 0.42)	*DASH*								
-0.13 (-0.90, 0.63)	-0.26 (-1.04, 0.52)	*Paleo*							
0.06 (-0.18, 0.30)	-0.07 (-0.35, 0.22)	0.19 (-0.54, 0.92)	*DG-based*						
-0.08 (-0.87, 0.71)	-0.21 (-1.02, 0.61)	0.05 (-1.03, 1.13)	-0.14 (-0.94, 0.66)	*Low GI/GL*					
0.11 (-0.13, 0.36)	-0.01 (-0.33, 0.30)	0.25 (-0.53, 1.02)	0.06 (-0.20, 0.31)	0.19 (-0.61, 1.00)	*Plant-based*				
0.08 (-0.11, 0.27)	-0.04 (-0.34, 0.26)	0.22 (-0.55, 0.98)	0.03 (-0.22, 0.27)	0.16 (-0.63, 0.96)	-0.03 (-0.28, 0.21)	*Low fat*			
-0.37 (-0.77, 0.04)	**-0.49 (-0.95, -0.04)**	-0.24 (-1.08, 0.60)	**-0.43 (-0.84, -0.01)**	-0.29 (-1.15, 0.58)	**-0.48 (-0.87, -0.10)**	**-0.45 (-0.87, -0.04)**	*Low CHO high fat*		
-0.21 (-1.01, 0.58)	-0.34 (-1.15, 0.48)	-0.08 (-1.16, 1.00)	-0.27 (-1.07, 0.53)	-0.13 (-0.43, 0.16)	-0.33 (-1.13, 0.47)	-0.30 (-1.10, 0.50)	0.15 (-0.71, 1.02)	*High GI/GL*	
**-0.25 (-0.41, -0.09)**	**-0.37 (-0.62, -0.12)**	-0.12 (-0.87, 0.64)	**-0.31 (-0.49, -0.12)**	-0.17 (-0.94, 0.61)	**-0.36 (-0.56, -0.16)**	**-0.33 (-0.52, -0.14)**	0.12 (-0.26, 0.50)	-0.03 (-0.81, 0.74)	*Western habitual*

[1]The values correspond to the mean difference (95% CI) in total cholesterol (mmol/L) between the column dietary pattern and the row dietary pattern; column minus row. Values in bold are effect sizes that are statistically significant at level of 0.05.

Abbreviations: CHO, carbohydrate; DASH, Dietary Approaches to Stop Hypertension; DG, dietary guidelines; GI/GL, glycemic index/glycemic load

Supplementary Table S49. League table of sensitivity analysis excluding studies with intervention duration longer than 52 weeks for triglycerides[1]

Mediterranean									
0.04 (-0.08, 0.16)	*DASH*								
-0.01 (-0.21, 0.18)	-0.05 (-0.26, 0.16)	*Paleo*							
0.05 (-0.04, 0.14)	0.01 (-0.11, 0.12)	0.06 (-0.12, 0.24)	*DG-based*						
-0.17 (-0.64, 0.31)	-0.20 (-0.69, 0.28)	-0.15 (-0.66, 0.36)	-0.21 (-0.69, 0.27)	*Low GI/GL*					
-0.04 (-0.12, 0.04)	-0.08 (-0.20, 0.04)	-0.03 (-0.22, 0.17)	-0.09 (-0.17, 0.00)	0.13 (-0.35, 0.60)	*Plant-based*				
-0.02 (-0.09, 0.05)	-0.06 (-0.18, 0.06)	-0.01 (-0.20, 0.19)	-0.07 (-0.15, 0.02)	0.14 (-0.33, 0.62)	0.02 (-0.06, 0.09)	*Low fat*			
0.17 (-0.03, 0.37)	0.13 (-0.09, 0.35)	0.18 (-0.09, 0.45)	0.12 (-0.09, 0.32)	0.33 (-0.18, 0.84)	**0.21 (0.01, 0.40)**	0.19 (-0.01, 0.39)	*Low CHO high fat*		
-0.22 (-0.69, 0.26)	-0.25 (-0.74, 0.23)	-0.20 (-0.71, 0.31)	-0.26 (-0.74, 0.22)	-0.05 (-0.14, 0.03)	-0.18 (-0.65, 0.30)	-0.19 (-0.67, 0.28)	-0.38 (-0.89, 0.13)	*High GI/GL*	
0.01 (-0.05, 0.07)	-0.03 (-0.13, 0.07)	0.02 (-0.17, 0.21)	-0.04 (-0.11, 0.03)	0.17 (-0.30, 0.65)	0.05 (-0.02, 0.11)	0.03 (-0.03, 0.09)	-0.16 (-0.35, 0.04)	0.23 (-0.25, 0.70)	*Western habitual*

[1]The values correspond to the mean difference (95% CI) in triglycerides (mmol/L) between the column dietary pattern and the row dietary pattern; column minus row. Values in bold are effect sizes that are statistically significant at level of 0.05.

Abbreviations: CHO, carbohydrate; DASH, Dietary Approaches to Stop Hypertension; DG, dietary guidelines; GI/GL, glycemic index/glycemic load

Supplementary Table S50. League table of sensitivity analysis excluding studies with intervention duration longer than 52 weeks for glucose[1]

Mediterranean										
0.06 (-0.07, 0.20)	*DASH*									
-0.25 (-0.64, 0.15)	-0.31 (-0.71, 0.09)	*Paleo*								
-0.04 (-0.13, 0.06)	-0.10 (-0.22, 0.02)	0.21 (-0.17, 0.59)	*DG-based*							
0.04 (-0.39, 0.46)	-0.03 (-0.46, 0.41)	0.28 (-0.29, 0.86)	0.07 (-0.35, 0.50)	*Low GI/GL*						
0.06 (-0.16, 0.28)	-0.01 (-0.25, 0.23)	0.30 (-0.14, 0.75)	0.09 (-0.13, 0.31)	0.02 (-0.45, 0.49)	*Plant-based*					
0.01 (-0.07, 0.10)	-0.05 (-0.19, 0.09)	0.26 (-0.14, 0.65)	0.05 (-0.05, 0.14)	-0.03 (-0.45, 0.40)	-0.05 (-0.27, 0.18)	*Low fat*				
0.15 (-0.06, 0.37)	0.09 (-0.15, 0.32)	0.40 (-0.05, 0.84)	0.19 (-0.03, 0.40)	0.11 (-0.35, 0.58)	0.09 (-0.15, 0.34)	0.14 (-0.08, 0.36)	*Low CHO high fat*			
0.05 (-0.11, 0.21)	-0.01 (-0.20, 0.18)	0.30 (-0.12, 0.72)	0.09 (-0.07, 0.25)	0.02 (-0.43, 0.46)	-0.01 (-0.26, 0.25)	0.04 (-0.13, 0.21)	-0.10 (-0.35, 0.15)	*Mexican*		
0.11 (-0.32, 0.53)	0.04 (-0.39, 0.48)	0.35 (-0.22, 0.93)	0.14 (-0.28, 0.57)	0.07 (-0.03, 0.17)	0.05 (-0.42, 0.52)	0.10 (-0.33, 0.52)	-0.04 (-0.51, 0.42)	0.06 (-0.39, 0.50)	*High GI/GL*	
-0.03 (-0.09, 0.04)	-0.09 (-0.21, 0.03)	0.22 (-0.17, 0.61)	0.01 (-0.06, 0.08)	-0.06 (-0.48, 0.35)	-0.09 (-0.29, 0.12)	-0.04 (-0.13, 0.05)	-0.18 (-0.38, 0.03)	-0.08 (-0.23, 0.07)	-0.14 (-0.55, 0.28)	*Western habitual*

[1]The values correspond to the mean difference (95% CI) in glucose (mmol/L) between the column dietary pattern and the row dietary pattern; column minus row.

Abbreviations: CHO, carbohydrate; DASH, Dietary Approaches to Stop Hypertension; DG, dietary guidelines; GI/GL, glycemic index/glycemic load

Supplementary Table S51. League table of sensitivity analysis excluding studies with intervention duration longer than 52 weeks for insulin[1]

Mediterranean									
-0.24 (-6.28, 5.80)	*DASH*								
0.34 (-7.97, 8.66)	0.59 (-8.66, 9.83)	*Paleo*							
-0.22 (-3.95, 3.52)	0.03 (-5.48, 5.53)	-0.56 (-7.99, 6.87)	*DG-based*						
-3.42 (-11.98, 5.14)	-3.18 (-13.06, 6.70)	-3.77 (-15.20, 7.67)	-3.21 (-11.90, 5.48)	*Low GI/GL*					
-3.46 (-11.33, 4.40)	-3.22 (-12.51, 6.07)	-3.81 (-14.73, 7.11)	-3.25 (-11.26, 4.76)	-0.04 (-11.12, 11.03)	*Plant-based*				
1.26 (-1.84, 4.36)	1.50 (-4.88, 7.87)	0.91 (-7.61, 9.43)	1.47 (-2.70, 5.64)	4.68 (-4.17, 13.52)	4.72 (-3.46, 12.90)	*Low fat*			
-0.80 (-8.76, 7.16)	-0.55 (-9.92, 8.82)	-1.14 (-12.13, 9.85)	-0.58 (-8.68, 7.52)	2.63 (-8.52, 13.77)	2.67 (-4.36, 9.69)	-2.05 (-10.32, 6.22)	*Low CHO high fat*		
-3.87 (-12.24, 4.51)	-3.62 (-13.34, 6.10)	-4.21 (-15.50, 7.08)	-3.65 (-12.15, 4.85)	-0.44 (-3.72, 2.83)	-0.40 (-11.33, 10.53)	-5.12 (-13.79, 3.54)	-3.07 (-14.07, 7.93)	*High GI/GL*	
-1.91 (-4.41, 0.59)	-1.67 (-7.21, 3.86)	-2.26 (-10.24, 5.72)	-1.70 (-4.61, 1.22)	1.51 (-6.68, 9.69)	1.55 (-5.91, 9.01)	-3.17 (-6.52, 0.18)	-1.12 (-8.68, 6.44)	1.95 (-6.04, 9.94)	*Western habitual*

[1]The values correspond to the mean difference (95% CI) in insulin (mU/L) between the column dietary pattern and the row dietary pattern; column minus row.

Abbreviations: CHO, carbohydrate; DASH, Dietary Approaches to Stop Hypertension; DG, dietary guidelines; GI/GL, glycemic index/glycemic load

Supplementary Table S52. League table of sensitivity analysis excluding studies with intervention duration longer than 52 weeks for HOMA-IR[1]

Mediterranean						
0.16<br>(-0.23, 0.56)	*DG-based*					
-0.14<br>(-1.23, 0.94)	-0.31<br>(-1.35, 0.74)	*Low GI/GL*				
**0.72**<br>**(0.23, 1.21)**	**0.55**<br>**(0.16, 0.95)**	0.86<br>(-0.20, 1.92)	*Plant-based*			
-0.03<br>(-0.33, 0.27)	-0.20<br>(-0.52, 0.13)	0.11<br>(-0.96, 1.19)	**-0.75**<br>**(-1.21, -0.29)**	*Low fat*		
-0.10<br>(-1.23, 1.03)	-0.27<br>(-1.35, 0.82)	0.04<br>(-0.48, 0.56)	-0.82<br>(-1.92, 0.28)	-0.07<br>(-1.19, 1.05)	*High GI/GL*	
-0.18<br>(-0.56, 0.20)	**-0.35**<br>**(-0.59, -0.10)**	-0.04<br>(-1.06, 0.98)	**-0.90**<br>**(-1.21, -0.59)**	-0.15<br>(-0.50, 0.20)	-0.08<br>(-1.14, 0.98)	*Western habitual*

[1]The values correspond to the mean difference (95% CI) in HOMA-IR between the column dietary pattern and the row dietary pattern; column minus row. Values in bold are effect sizes that are statistically significant at level of 0.05.

Abbreviations: DG, dietary guidelines; GI/GL, glycemic index/glycemic load; HOMA-IR, homeostatic model assessment for insulin resistance

Supplementary Table S53. League table of sensitivity analysis excluding studies with intervention duration longer than 52 weeks for hsCRP[1]

Mediterranean							
0.34 (-0.87, 1.55)	*DASH*						
0.12 (-1.43, 1.67)	-0.22 (-1.93, 1.49)	*Paleo*					
0.27 (-0.42, 0.96)	-0.07 (-1.06, 0.92)	0.15 (-1.24, 1.54)	*DG-based*				
-0.11 (-0.78, 0.56)	-0.45 (-1.76, 0.86)	-0.23 (-1.86, 1.40)	-0.38 (-1.24, 0.48)	*Plant-based*			
-0.48 (-1.10, 0.15)	-0.82 (-2.08, 0.45)	-0.60 (-2.18, 0.99)	-0.75 (-1.52, 0.03)	-0.37 (-1.26, 0.52)	*Low fat*		
-0.26 (-0.97, 0.45)	-0.60 (-1.95, 0.74)	-0.38 (-2.04, 1.27)	-0.53 (-1.43, 0.37)	-0.15 (-0.91, 0.60)	0.21 (-0.72, 1.14)	*Low CHO high fat*	
-0.24 (-0.67, 0.18)	-0.58 (-1.74, 0.57)	-0.36 (-1.87, 1.14)	-0.51 (-1.10, 0.08)	-0.13 (-0.77, 0.50)	0.23 (-0.47, 0.93)	0.02 (-0.66, 0.70)	*Western habitual*

[1]The values correspond to the mean difference (95% CI) in hsCRP (mg/L) between the column dietary pattern and the row dietary pattern; column minus row. Values in bold are effect sizes that are statistically significant at level of 0.05.

Abbreviations: CHO, carbohydrate; DASH, Dietary Approaches to Stop Hypertension; DG, dietary guidelines; hsCRP, high sensitivity C-reactive protein

Supplementary Table S54. League table of sensitivity analysis excluding studies with intervention duration longer than 52 weeks for interleukin-6[1]

Mediterranean		
-0.19<br>(-0.71, 0.34)	*DG-based*	
-0.13<br>(-0.59, 0.34)	0.06<br>(-0.18, 0.30)	*Western habitual*

[1]The values correspond to the mean difference (95% CI) in interleukin-6 (pg/mL) between the column dietary pattern and the row dietary pattern; column minus row. Values in bold are effect sizes that are statistically significant at level of 0.05.

Abbreviations: DG, dietary guidelines

Supplementary Table S55. League table of sensitivity analysis excluding studies with participants' mean age greater than 70 years for LDL-c[1]

Mediterranean									
-0.04 (-0.28, 0.20)	*DASH*								
-0.25 (-0.72, 0.22)	-0.21 (-0.71, 0.28)	*Paleo*							
-0.12 (-0.31, 0.07)	-0.08 (-0.32, 0.16)	0.13 (-0.30, 0.56)	*DG-based*						
-0.24 (-0.86, 0.39)	-0.20 (-0.84, 0.45)	0.02 (-0.75, 0.78)	-0.12 (-0.75, 0.51)	*Low GI/GL*					
-0.02 (-0.22, 0.18)	0.02 (-0.24, 0.29)	0.24 (-0.24, 0.72)	0.10 (-0.11, 0.31)	0.22 (-0.41, 0.85)	*Plant-based*				
-0.04 (-0.18, 0.11)	0.00 (-0.24, 0.25)	0.22 (-0.26, 0.69)	0.08 (-0.12, 0.28)	0.20 (-0.43, 0.83)	-0.02 (-0.22, 0.18)	*Low fat*			
**-0.38 (-0.70, -0.05)**	-0.33 (-0.70, 0.03)	-0.12 (-0.67, 0.43)	-0.25 (-0.59, 0.08)	-0.14 (-0.82, 0.55)	**-0.36 (-0.66, -0.05)**	**-0.34 (-0.67, -0.01)**	*Low CHO high fat*		
-0.28 (-0.90, 0.33)	-0.24 (-0.88, 0.39)	-0.03 (-0.79, 0.73)	-0.16 (-0.78, 0.46)	-0.04 (-0.25, 0.16)	-0.27 (-0.89, 0.36)	-0.25 (-0.87, 0.37)	0.09 (-0.58, 0.76)	*High GI/GL*	
**-0.30 (-0.43, -0.16)**	**-0.26 (-0.47, -0.04)**	-0.04 (-0.50, 0.41)	**-0.18 (-0.33, -0.02)**	-0.06 (-0.67, 0.55)	**-0.28 (-0.44, -0.12)**	**-0.26 (-0.41, -0.11)**	0.08 (-0.22, 0.38)	-0.01 (-0.61, 0.59)	*Western habitual*

[1]The values correspond to the mean difference (95% CI) in LDL-c (mmol/L) between the column dietary pattern and the row dietary pattern; column minus row. Values in bold are effect sizes that are statistically significant at level of 0.05.

Abbreviations: CHO, carbohydrate; DASH, Dietary Approaches to Stop Hypertension; DG, dietary guidelines; GI/GL, glycemic index/glycemic load

Supplementary Table S56. League table of sensitivity analysis excluding studies with participants' mean age greater than 70 years for HDL-c[1]

Mediterranean									
**0.07 (0.00, 0.13)**	*DASH*								
-0.07 (-0.22, 0.07)	-0.14 (-0.29, 0.01)	*Paleo*							
0.03 (-0.02, 0.09)	-0.03 (-0.10, 0.03)	0.11 (-0.03, 0.24)	*DG-based*						
0.02 (-0.23, 0.26)	-0.05 (-0.30, 0.20)	0.09 (-0.19, 0.37)	-0.02 (-0.26, 0.23)	*Low GI/GL*					
**0.07 (0.01, 0.12)**	0.00 (-0.07, 0.07)	0.14 (-0.01, 0.29)	0.03 (-0.03, 0.10)	0.05 (-0.20, 0.30)	*Plant-based*				
0.05 (-0.00, 0.10)	-0.02 (-0.09, 0.05)	0.12 (-0.03, 0.27)	0.01 (-0.04, 0.07)	0.03 (-0.22, 0.28)	-0.02 (-0.08, 0.04)	*Low fat*			
**-0.12 (-0.23, -0.02)**	**-0.19 (-0.30, -0.08)**	-0.05 (-0.22, 0.12)	**-0.16 (-0.26, -0.05)**	-0.14 (-0.40, 0.12)	**-0.19 (-0.29, -0.09)**	**-0.17 (-0.28, -0.06)**	*Low CHO high fat*		
-0.01 (-0.25, 0.24)	-0.07 (-0.32, 0.18)	0.07 (-0.22, 0.35)	-0.04 (-0.29, 0.21)	-0.02 (-0.08, 0.04)	-0.07 (-0.32, 0.17)	-0.05 (-0.30, 0.19)	0.12 (-0.15, 0.38)	*High GI/GL*	
-0.03 (-0.07, 0.01)	**-0.10 (-0.15, -0.04)**	0.04 (-0.10, 0.19)	**-0.06 (-0.11, -0.02)**	-0.05 (-0.29, 0.20)	**-0.10 (-0.14, -0.05)**	**-0.08 (-0.12, -0.03)**	0.09 (-0.01, 0.19)	-0.02 (-0.27, 0.22)	*Western habitual*

[1]The values correspond to the mean difference (95% CI) in HDL-c (mmol/L) between the column dietary pattern and the row dietary pattern; column minus row. Values in bold are effect sizes that are statistically significant at level of 0.05.

Abbreviations: CHO, carbohydrate; DASH, Dietary Approaches to Stop Hypertension; DG, dietary guidelines; GI/GL, glycemic index/glycemic load

Supplementary Table S57. League table of sensitivity analysis excluding studies with participants' mean age greater than 70 years for total cholesterol[1]

Mediterranean									
0.05 (-0.26, 0.35)	*DASH*								
-0.27 (-0.81, 0.28)	-0.31 (-0.89, 0.27)	*Paleo*							
-0.07 (-0.30, 0.16)	-0.12 (-0.41, 0.18)	0.20 (-0.30, 0.70)	*DG-based*						
-0.14 (-0.95, 0.66)	-0.19 (-1.03, 0.64)	0.12 (-0.83, 1.07)	-0.07 (-0.89, 0.74)	*Low GI/GL*					
0.05 (-0.20, 0.30)	0.00 (-0.33, 0.33)	0.31 (-0.25, 0.88)	0.12 (-0.14, 0.38)	0.19 (-0.63, 1.01)	*Plant-based*				
0.04 (-0.15, 0.23)	-0.01 (-0.32, 0.30)	0.30 (-0.26, 0.86)	0.11 (-0.14, 0.35)	0.18 (-0.63, 1.00)	-0.01 (-0.27, 0.24)	*Low fat*			
**-0.43 (-0.84, -0.01)**	**-0.48 (-0.95, -0.01)**	-0.16 (-0.82, 0.49)	-0.36 (-0.79, 0.07)	-0.28 (-1.17, 0.60)	**-0.48 (-0.87, -0.08)**	**-0.47 (-0.89, -0.04)**	*Low CHO high fat*		
-0.28 (-1.09, 0.53)	-0.33 (-1.16, 0.51)	-0.01 (-0.97, 0.94)	-0.21 (-1.02, 0.60)	-0.13 (-0.44, 0.17)	-0.33 (-1.15, 0.49)	-0.32 (-1.13, 0.50)	0.15 (-0.73, 1.03)	*High GI/GL*	
**-0.31 (-0.48, -0.15)**	**-0.36 (-0.62, -0.10)**	-0.05 (-0.58, 0.49)	**-0.24 (-0.42, -0.06)**	-0.17 (-0.96, 0.62)	**-0.36 (-0.57, -0.15)**	**-0.35 (-0.54, -0.16)**	0.12 (-0.27, 0.51)	-0.03 (-0.82, 0.76)	*Western habitual*

[1]The values correspond to the mean difference (95% CI) in total cholesterol (mmol/L) between the column dietary pattern and the row dietary pattern; column minus row. Values in bold are effect sizes that are statistically significant at level of 0.05.

Abbreviations: CHO, carbohydrate; DASH, Dietary Approaches to Stop Hypertension; DG, dietary guidelines; GI/GL, glycemic index/glycemic load

Supplementary Table S58. League table of sensitivity analysis excluding studies with participants' mean age greater than 70 years for triglycerides[1]

Mediterranean									
-0.00<br>(-0.15, 0.14)	*DASH*								
-0.03<br>(-0.23, 0.16)	-0.03<br>(-0.25, 0.19)	*Paleo*							
-0.04<br>(-0.14, 0.05)	-0.04<br>(-0.18, 0.10)	-0.01<br>(-0.18, 0.16)	*DG-based*						
-0.20<br>(-0.70, 0.29)	-0.20<br>(-0.70, 0.31)	-0.17<br>(-0.69, 0.36)	-0.16<br>(-0.65, 0.34)	*Low GI/GL*					
-0.08<br>(-0.19, 0.02)	-0.08<br>(-0.24, 0.07)	-0.05<br>(-0.26, 0.15)	-0.04<br>(-0.15, 0.07)	0.12<br>(-0.38, 0.62)	*Plant-based*				
-0.04<br>(-0.13, 0.04)	-0.04<br>(-0.19, 0.11)	-0.01<br>(-0.21, 0.19)	0.00<br>(-0.10, 0.11)	0.16<br>(-0.34, 0.66)	0.04<br>(-0.06, 0.15)	*Low fat*			
0.14<br>(-0.08, 0.35)	0.14<br>(-0.11, 0.39)	0.17<br>(-0.11, 0.45)	0.18<br>(-0.04, 0.40)	0.34<br>(-0.20, 0.87)	**0.22**<br>**(0.00, 0.44)**	0.18<br>(-0.04, 0.40)	*Low CHO high fat*		
-0.25<br>(-0.75, 0.25)	-0.25<br>(-0.75, 0.26)	-0.22<br>(-0.74, 0.31)	-0.20<br>(-0.70, 0.29)	-0.05<br>(-0.15, 0.06)	-0.16<br>(-0.66, 0.33)	-0.21<br>(-0.71, 0.29)	-0.38<br>(-0.92, 0.15)	*High GI/GL*	
-0.03<br>(-0.10, 0.05)	-0.02<br>(-0.15, 0.11)	0.01<br>(-0.18, 0.20)	0.02<br>(-0.06, 0.10)	0.18<br>(-0.31, 0.67)	0.06<br>(-0.03, 0.15)	0.02<br>(-0.07, 0.10)	-0.16<br>(-0.37, 0.05)	0.22<br>(-0.27, 0.71)	*Western habitual*

[1]The values correspond to the mean difference (95% CI) in triglycerides (mmol/L) between the column dietary pattern and the row dietary pattern; column minus row. Values in bold are effect sizes that are statistically significant at level of 0.05.

Abbreviations: CHO, carbohydrate; DASH, Dietary Approaches to Stop Hypertension; DG, dietary guidelines; GI/GL, glycemic index/glycemic load

Supplementary Table S59. League table of sensitivity analysis excluding studies with participants' mean age greater than 70 years for glucose[1]

Mediterranean										
0.03 (-0.11, 0.16)	*DASH*									
-0.06 (-0.36, 0.24)	-0.09 (-0.40, 0.22)	*Paleo*								
-0.08 (-0.17, 0.01)	-0.11 (-0.23, 0.01)	-0.02 (-0.31, 0.27)	*DG-based*							
0.01 (-0.42, 0.44)	-0.02 (-0.46, 0.42)	0.07 (-0.44, 0.58)	0.09 (-0.34, 0.52)	*Low GI/GL*						
0.03 (-0.19, 0.25)	0.00 (-0.24, 0.25)	0.09 (-0.27, 0.45)	0.11 (-0.11, 0.33)	0.02 (-0.45, 0.49)	*Plant-based*					
-0.01 (-0.10, 0.08)	-0.03 (-0.18, 0.11)	0.05 (-0.25, 0.36)	0.07 (-0.02, 0.17)	-0.02 (-0.45, 0.41)	-0.04 (-0.27, 0.19)	*Low fat*				
0.12 (-0.10, 0.34)	0.09 (-0.15, 0.34)	0.18 (-0.18, 0.54)	0.20 (-0.02, 0.42)	0.11 (-0.36, 0.58)	0.09 (-0.15, 0.34)	0.13 (-0.10, 0.36)	*Low CHO high fat*			
0.02 (-0.14, 0.19)	-0.00 (-0.20, 0.19)	0.08 (-0.25, 0.42)	0.10 (-0.06, 0.27)	0.02 (-0.43, 0.46)	-0.00 (-0.27, 0.26)	0.03 (-0.15, 0.21)	-0.10 (-0.36, 0.16)	*Mexican*		
0.08 (-0.35, 0.51)	0.05 (-0.39, 0.49)	0.14 (-0.37, 0.65)	0.16 (-0.27, 0.59)	0.07 (-0.04, 0.18)	0.05 (-0.42, 0.52)	0.09 (-0.34, 0.52)	-0.04 (-0.51, 0.43)	0.06 (-0.39, 0.50)	*High GI/GL*	
-0.06 (-0.12, 0.01)	-0.08 (-0.21, 0.04)	0.00 (-0.29, 0.30)	0.02 (-0.04, 0.09)	-0.06 (-0.49, 0.36)	-0.08 (-0.30, 0.13)	-0.05 (-0.14, 0.04)	-0.18 (-0.39, 0.03)	-0.08 (-0.23, 0.07)	-0.14 (-0.56, 0.29)	*Western habitual*

[1]The values correspond to the mean difference (95% CI) in glucose (mmol/L) between the column dietary pattern and the row dietary pattern; column minus row.

Abbreviations: CHO, carbohydrate; DASH, Dietary Approaches to Stop Hypertension; DG, dietary guidelines; GI/GL, glycemic index/glycemic load

Supplementary Table S60. League table of sensitivity analysis excluding studies with participants' mean age greater than 70 years for insulin[1]

Mediterranean									
-0.60 (-6.47, 5.26)	*DASH*								
0.64 (-5.66, 6.93)	1.24 (-6.13, 8.61)	*Paleo*							
-0.57 (-4.23, 3.09)	0.03 (-5.27, 5.33)	-1.21 (-6.33, 3.91)	*DG-based*						
-3.80 (-12.16, 4.57)	-3.19 (-12.78, 6.40)	-4.43 (-14.31, 5.45)	-3.22 (-11.67, 5.23)	*Low GI/GL*					
-3.83 (-11.50, 3.84)	-3.23 (-12.21, 5.76)	-4.47 (-13.77, 4.83)	-3.26 (-11.02, 4.50)	-0.04 (-10.80, 10.73)	*Plant-based*				
1.04 (-1.73, 3.81)	1.64 (-4.46, 7.75)	0.40 (-6.08, 6.88)	1.61 (-2.36, 5.58)	4.83 (-3.74, 13.41)	4.87 (-3.03, 12.77)	*Low fat*			
-1.20 (-8.97, 6.57)	-0.60 (-9.67, 8.48)	-1.84 (-11.22, 7.54)	-0.63 (-8.49, 7.23)	2.59 (-8.24, 13.43)	2.63 (-4.20, 9.46)	-2.24 (-10.24, 5.76)	*Low CHO high fat*		
-4.23 (-12.41, 3.95)	-3.62 (-13.05, 5.80)	-4.87 (-14.59, 4.86)	-3.66 (-11.92, 4.61)	-0.43 (-3.60, 2.73)	-0.40 (-11.02, 10.23)	-5.27 (-13.66, 3.13)	-3.03 (-13.72, 7.67)	*High GI/GL*	
-2.29 (-4.83, 0.25)	-1.68 (-7.01, 3.64)	-2.93 (-8.77, 2.91)	-1.72 (-4.52, 1.09)	1.51 (-6.46, 9.48)	1.54 (-5.70, 8.78)	**-3.33 (-6.49, -0.16)**	-1.09 (-8.43, 6.26)	1.94 (-5.84, 9.71)	*Western habitual*

[1]The values correspond to the mean difference (95% CI) in insulin (mU/L) between the column dietary pattern and the row dietary pattern; column minus row. Values in bold are effect sizes that are statistically significant at level of 0.05.

Abbreviations: CHO, carbohydrate; DASH, Dietary Approaches to Stop Hypertension; DG, dietary guidelines; GI/GL, glycemic index/glycemic load

Supplementary Table S61. League table of sensitivity analysis excluding studies with participants' mean age greater than 70 years for hsCRP[1]

Mediterranean							
0.36 (-0.89, 1.61)	*DASH*						
0.52 (-0.73, 1.78)	0.16 (-1.29, 1.61)	*Paleo*					
0.29 (-0.42, 1.01)	-0.07 (-1.10, 0.96)	-0.23 (-1.26, 0.80)	*DG-based*				
-0.10 (-0.80, 0.60)	-0.47 (-1.82, 0.89)	-0.63 (-1.98, 0.73)	-0.40 (-1.28, 0.49)	*Plant-based*			
-0.46 (-1.10, 0.18)	-0.82 (-2.12, 0.48)	-0.98 (-2.28, 0.32)	-0.75 (-1.55, 0.05)	-0.36 (-1.27, 0.56)	*Low fat*		
-0.26 (-1.00, 0.48)	-0.62 (-2.00, 0.76)	-0.78 (-2.17, 0.60)	-0.55 (-1.48, 0.37)	-0.16 (-0.94, 0.63)	0.20 (-0.75, 1.15)	*Low CHO high fat*	
-0.23 (-0.69, 0.23)	-0.59 (-1.78, 0.59)	-0.76 (-1.95, 0.44)	-0.52 (-1.12, 0.08)	-0.13 (-0.79, 0.53)	0.23 (-0.49, 0.95)	0.03 (-0.68, 0.73)	*Western habitual*

[1]The values correspond to the mean difference (95% CI) in hsCRP (mg/L) between the column dietary pattern and the row dietary pattern; column minus row. Values in bold are effect sizes that are statistically significant at level of 0.05.

Abbreviations: CHO, carbohydrate; DASH, Dietary Approaches to Stop Hypertension; DG, dietary guidelines; hsCRP, high sensitivity C-reactive protein

Supplementary Table S62. League table of sensitivity analysis excluding studies reported hsCRP levels greater than 10 mg/L[1]

Mediterranean							
0.24<br>(-0.90, 1.37)	*DASH*						
0.39<br>(-0.79, 1.57)	0.16<br>(-1.20, 1.51)	*Paleo*					
0.17<br>(-0.49, 0.82)	-0.07<br>(-1.00, 0.86)	-0.23<br>(-1.21, 0.75)	*DG-based*				
-0.10<br>(-0.73, 0.52)	-0.34<br>(-1.57, 0.89)	-0.49<br>(-1.76, 0.77)	-0.27<br>(-1.07, 0.54)	*Plant-based*			
-0.51<br>(-1.11, 0.08)	-0.75<br>(-1.94, 0.45)	-0.91<br>(-2.14, 0.32)	-0.68<br>(-1.43, 0.07)	-0.41<br>(-1.26, 0.43)	*Low fat*		
-0.25<br>(-0.92, 0.42)	-0.49<br>(-1.75, 0.77)	-0.65<br>(-1.95, 0.65)	-0.42<br>(-1.27, 0.43)	-0.15<br>(-0.86, 0.56)	0.26<br>(-0.62, 1.14)	*Low CHO high fat*	
-0.23<br>(-0.64, 0.17)	-0.47<br>(-1.56, 0.62)	-0.63<br>(-1.76, 0.50)	-0.40<br>(-0.96, 0.16)	-0.13<br>(-0.72, 0.45)	0.28<br>(-0.39, 0.95)	0.02<br>(-0.63, 0.66)	*Western habitual*

[1]The values correspond to the mean difference (95% CI) in hsCRP (mg/L) between the column dietary pattern and the row dietary pattern; column minus row. Values in bold are effect sizes that are statistically significant at level of 0.05.

Abbreviations: CHO, carbohydrate; DASH, Dietary Approaches to Stop Hypertension; DG, dietary guidelines; hsCRP, high sensitivity C-reactive protein

Supplementary Figure S26. Comparison-adjusted funnel plot for LDL-c

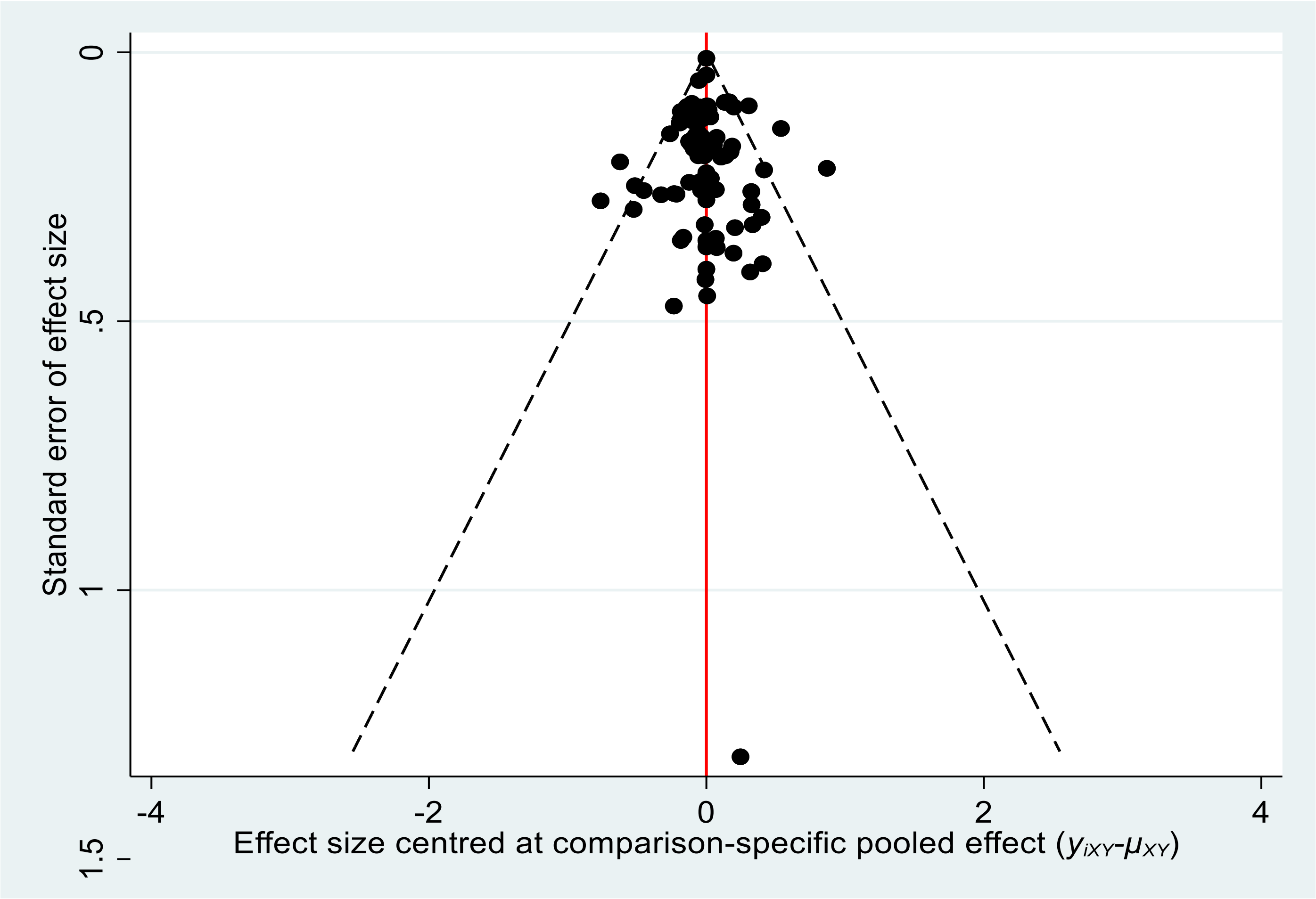

Comparison-adjusted funnel plot showed no asymmetry indicates no evidence of publication bias.

Supplementary Figure S27. Comparison-adjusted funnel plot for HDL-c

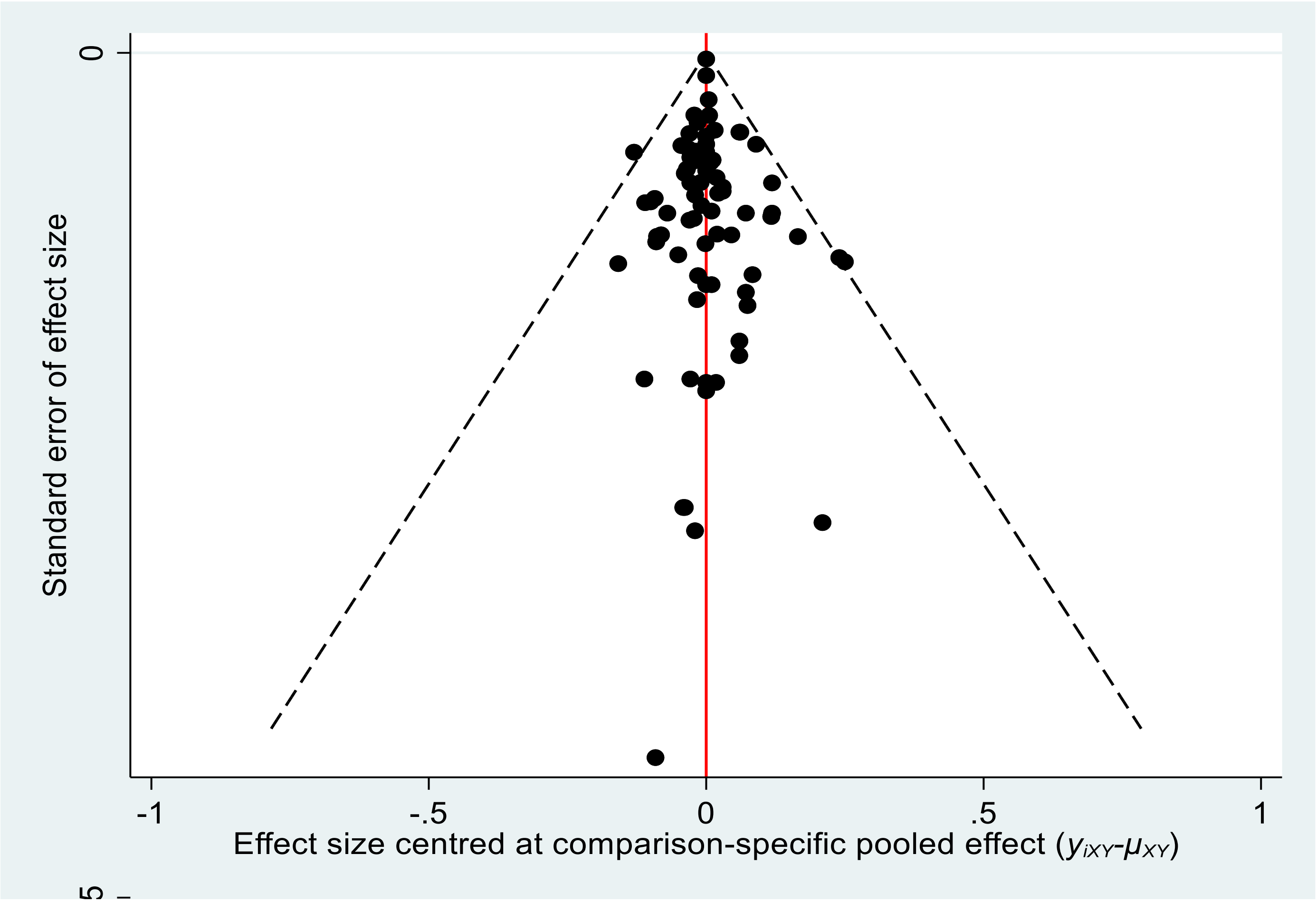

Comparison-adjusted funnel plot showed no asymmetry indicates no evidence of publication bias.

Supplementary Figure S28. Comparison-adjusted funnel plot for total cholesterol

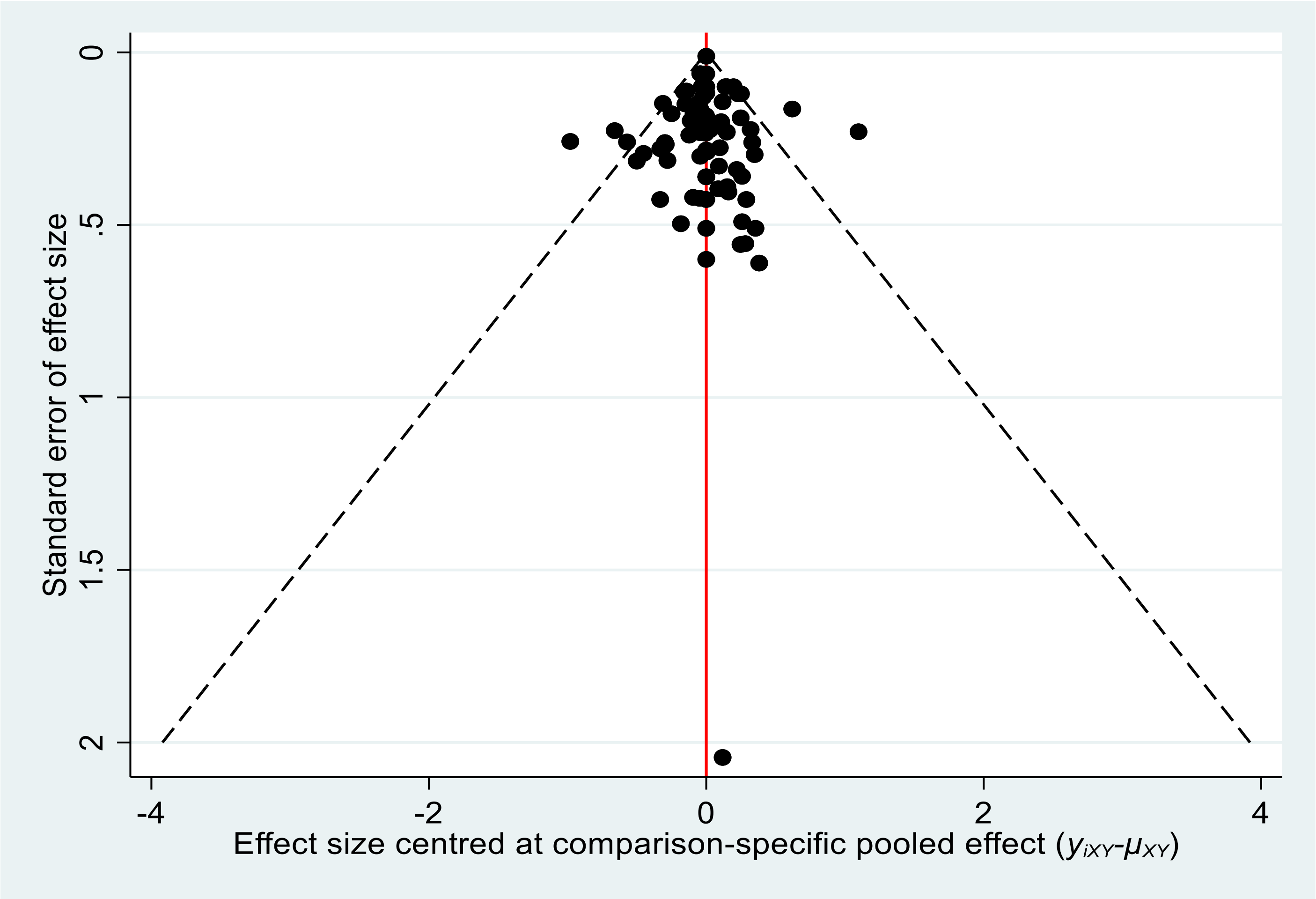

Comparison-adjusted funnel plot showed no asymmetry indicates no evidence of publication bias.

Supplementary Figure S29. Comparison-adjusted funnel plot for triglycerides

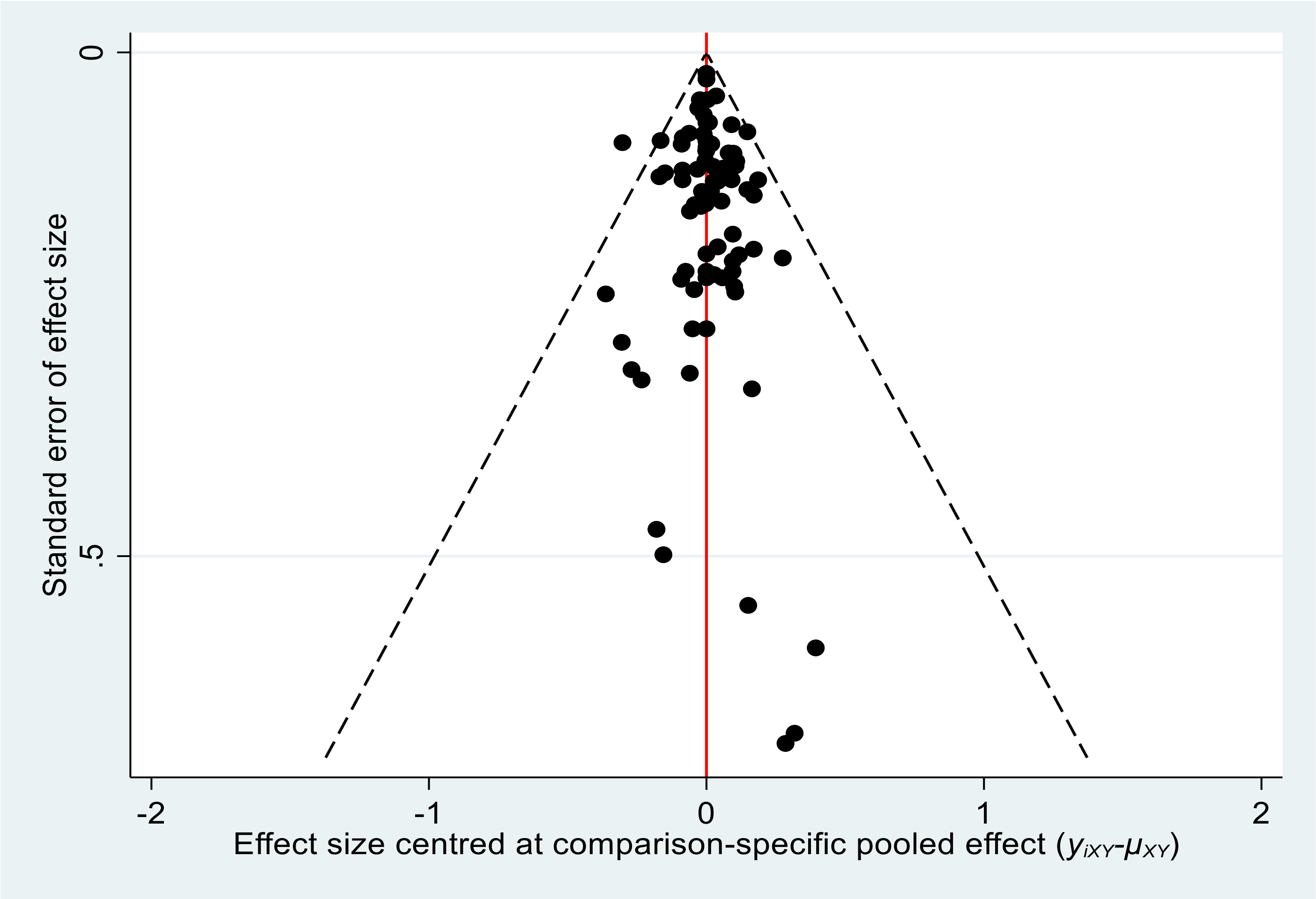

Comparison-adjusted funnel plot showed no asymmetry indicates no evidence of publication bias.

Supplementary Figure S30. Comparison-adjusted funnel plot for ApoB

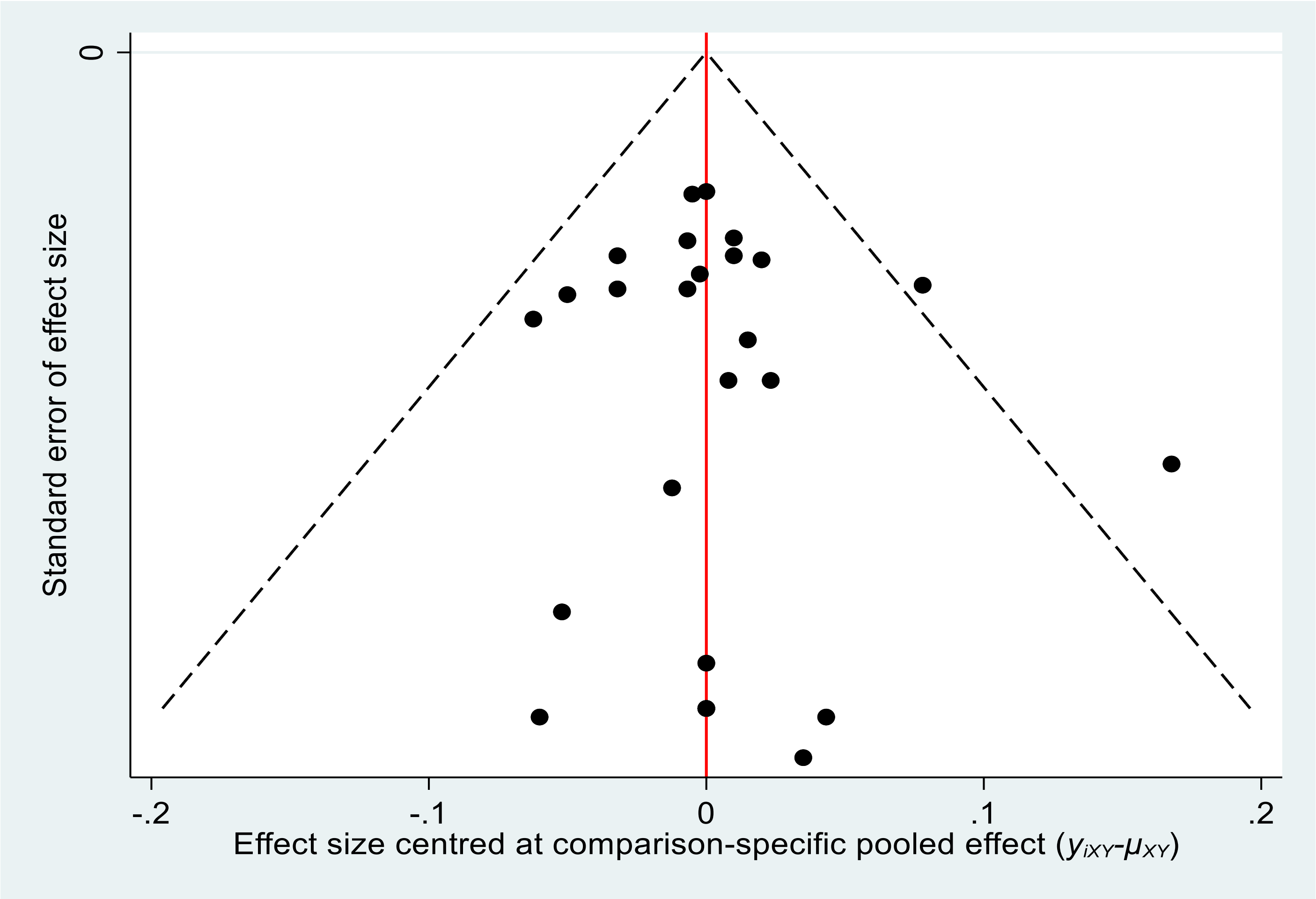

Comparison-adjusted funnel plot showed no asymmetry indicates no evidence of publication bias.

Supplementary Figure S31. Comparison-adjusted funnel plot for ApoA1

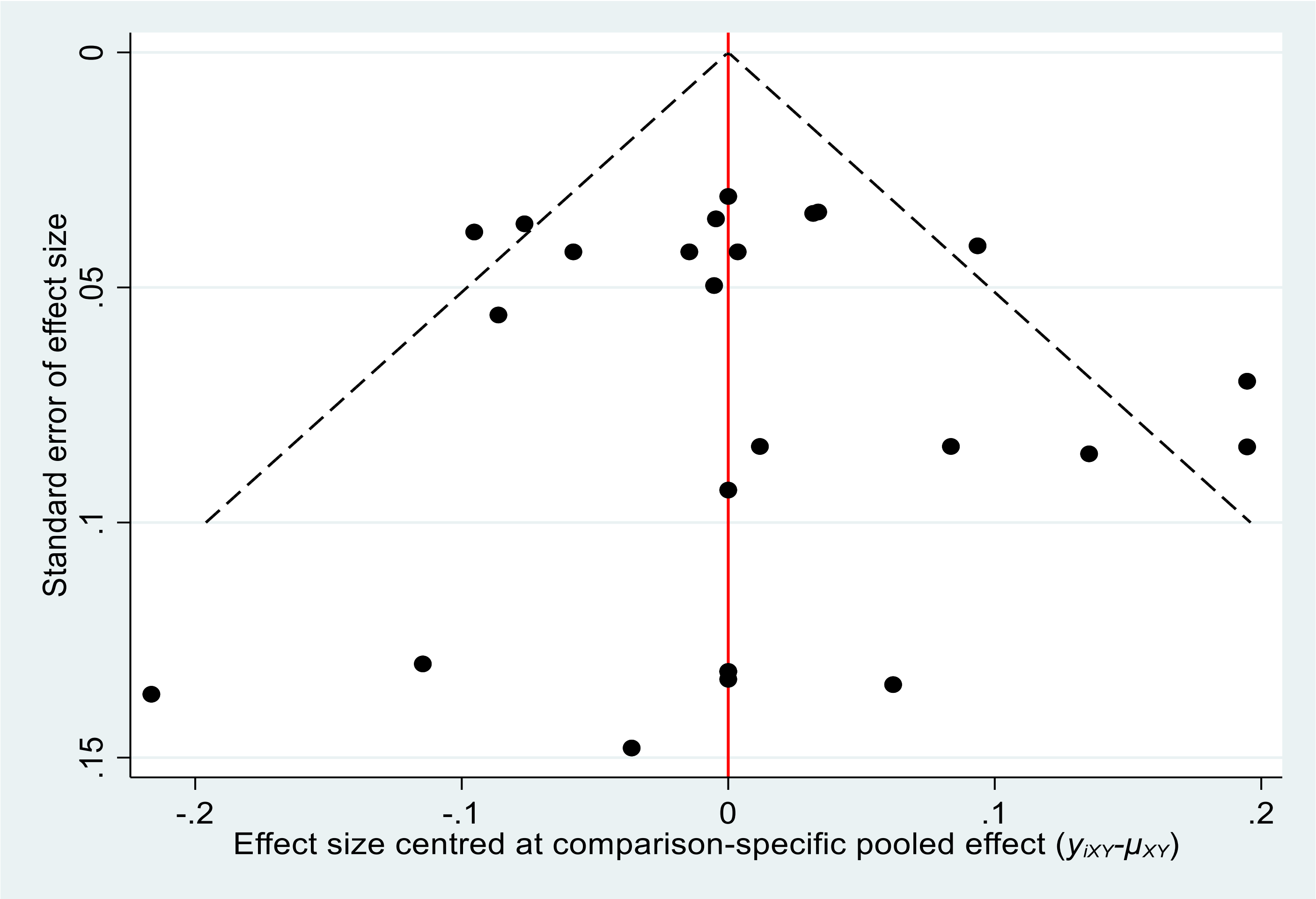

Comparison-adjusted funnel plot showed no asymmetry indicates no evidence of publication bias.

Supplementary Figure S32. Comparison-adjusted funnel plot for glucose

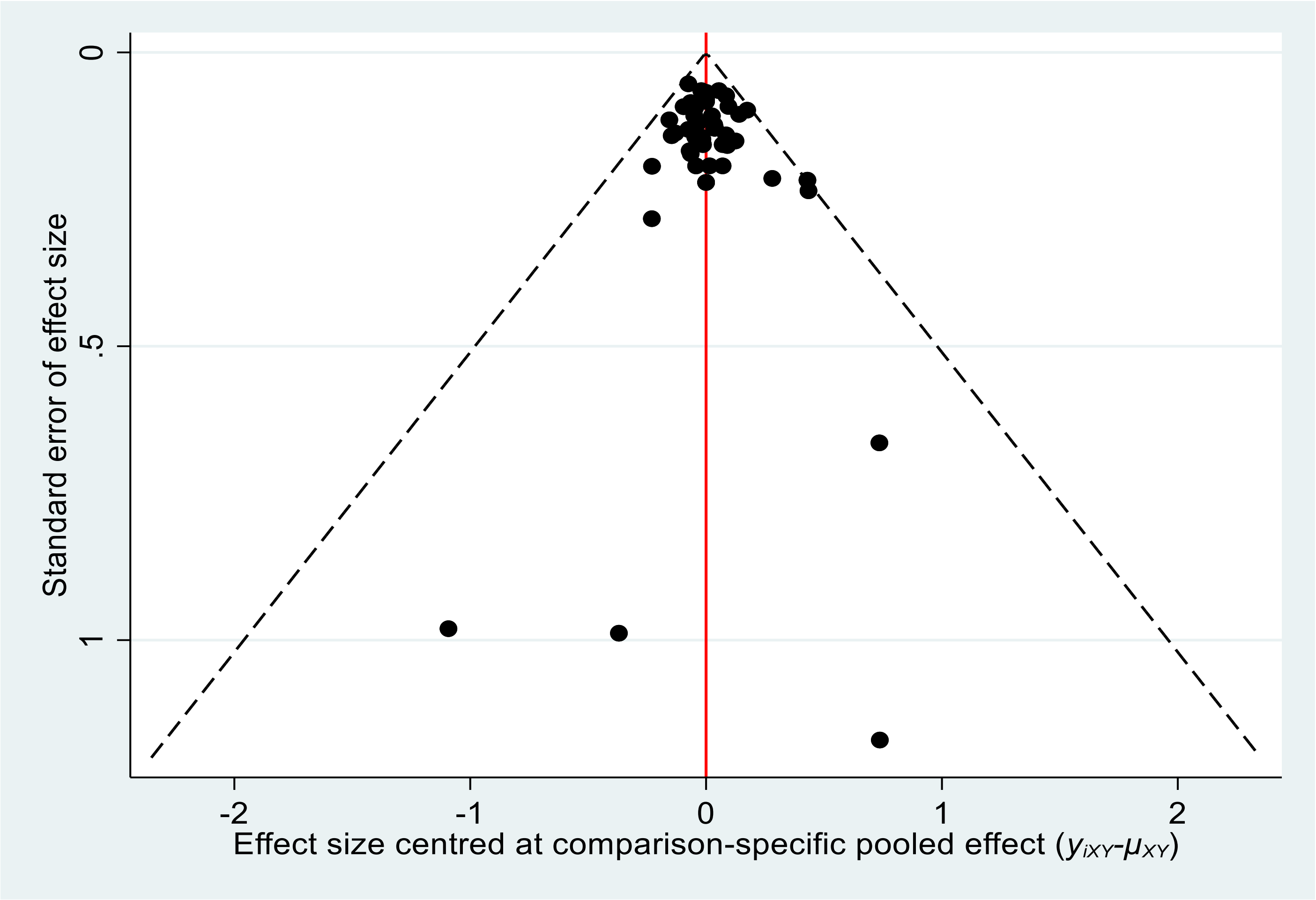

Comparison-adjusted funnel plot showed no asymmetry indicates no evidence of publication bias.

Supplementary Figure S33. Comparison-adjusted funnel plot for insulin

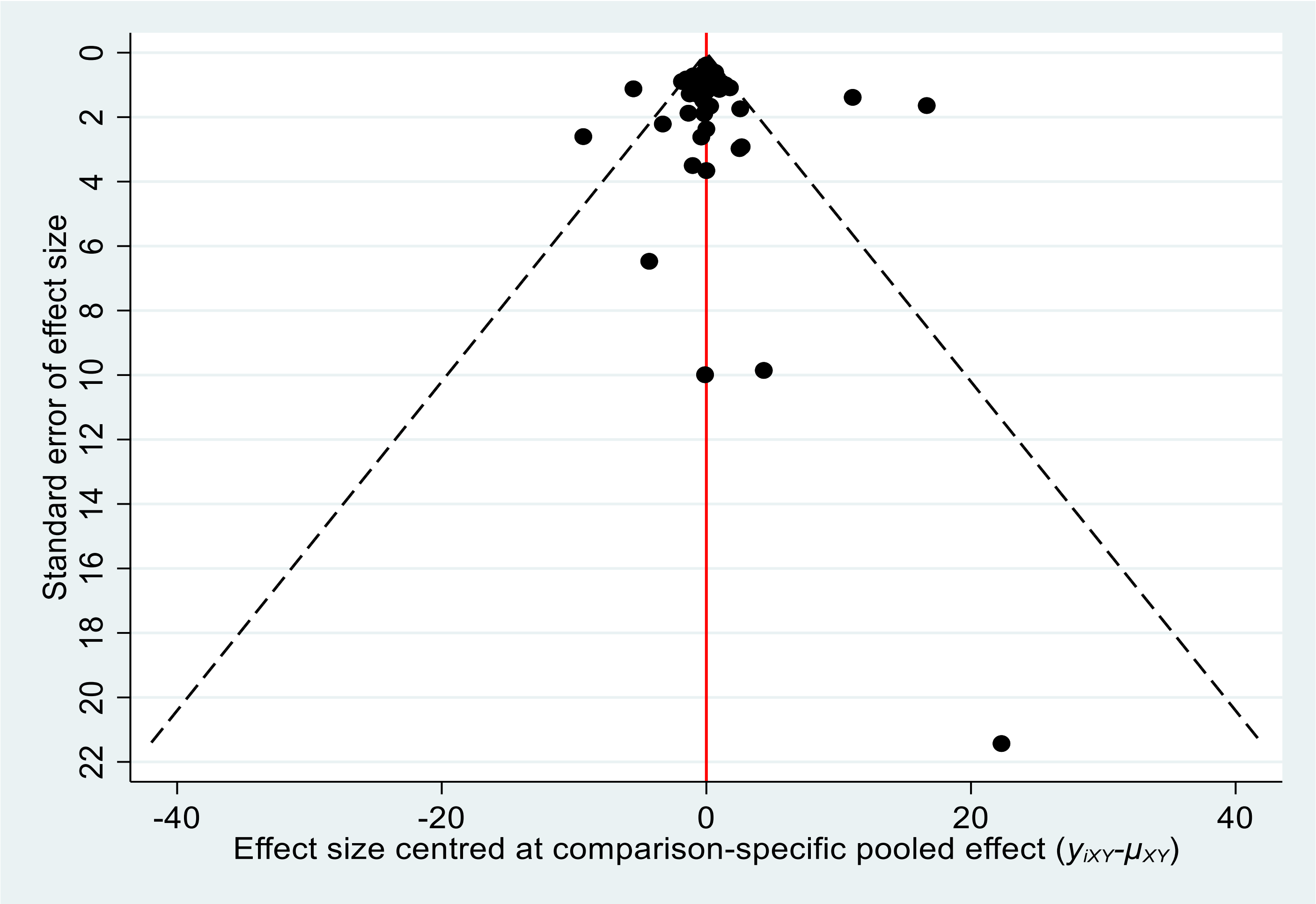

Comparison-adjusted funnel plot showed no asymmetry indicates no evidence of publication bias.

Supplementary Figure S34. Comparison-adjusted funnel plot for HOMA-IR

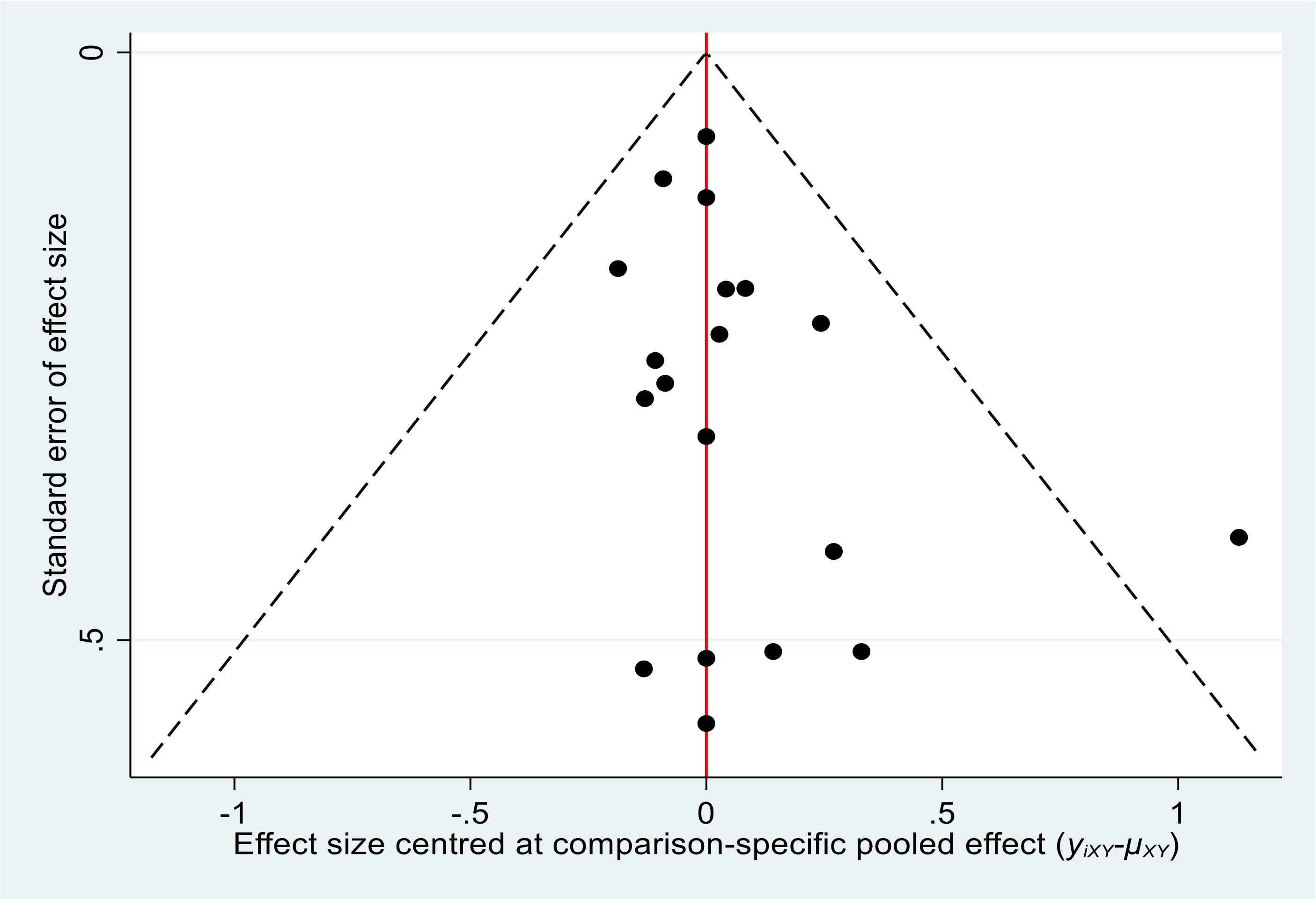

Comparison-adjusted funnel plot showed no asymmetry indicates no evidence of publication bias.

Supplementary Figure S35. Comparison-adjusted funnel plot for hsCRP

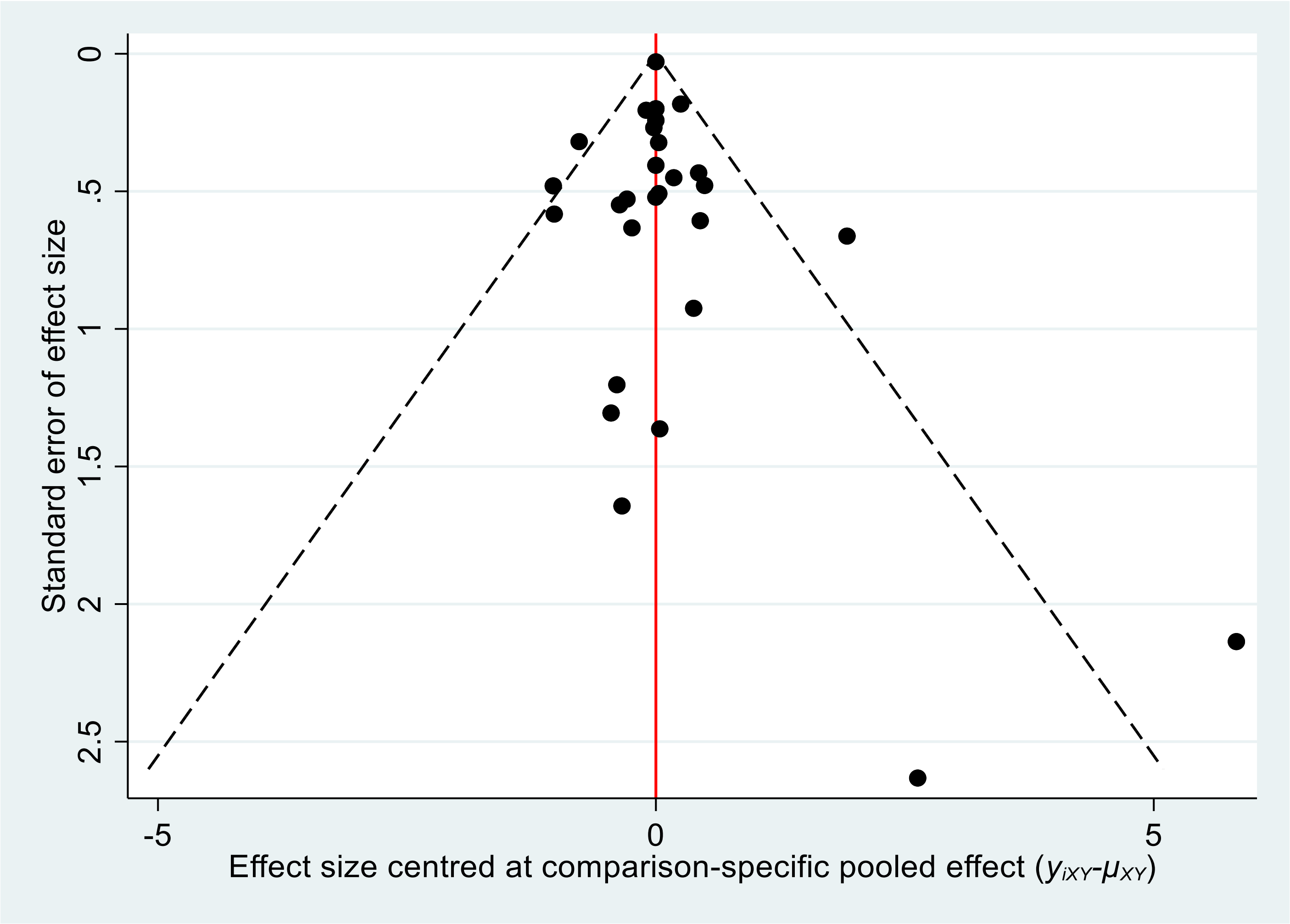

Comparison-adjusted funnel plot showed no asymmetry indicates no evidence of publication bias.

Supplementary Figure S36. Comparison-adjusted funnel plot for interleukin-6

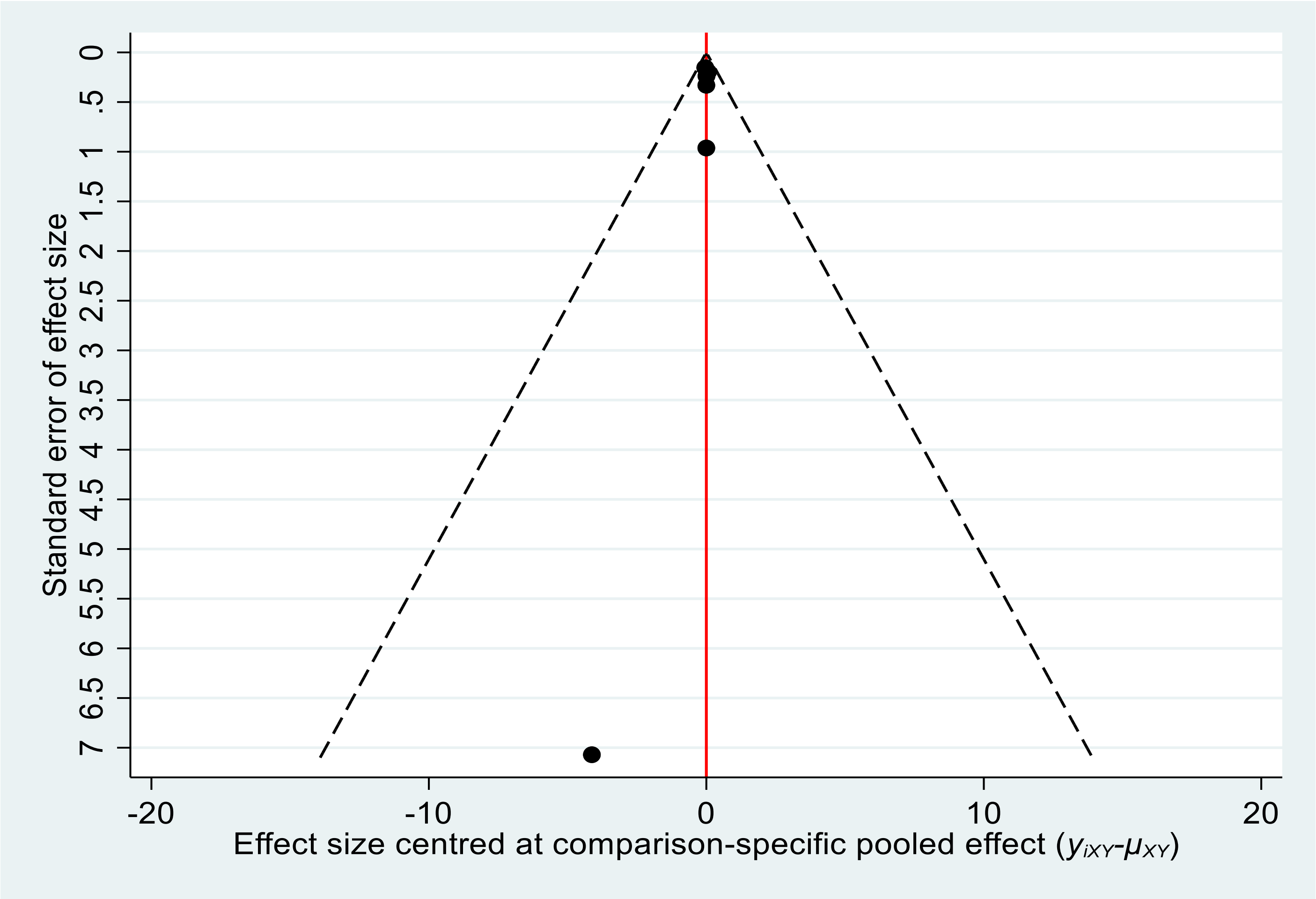

Comparison-adjusted funnel plot showed no asymmetry indicates no evidence of publication bias.

Supplementary Table S63. The Confidence in Network Meta-Analysis (CINeMA) framework ratings for credibility for LDL-c

Comparison	Number of studies	Within-study bias	Reporting bias	Indirectness	Imprecision	Heterogeneity	Incoherence	Confidence rating	Reason(s) for downgrading
A:D	1	Some concerns	Low risk	No concerns	Some concerns	Some concerns	Some concerns	Moderate	["Imprecision"]
A:F	1	Some concerns	Low risk	No concerns	Major concerns	No concerns	No concerns	Low	["Imprecision"]
A:G	10	Some concerns	Low risk	No concerns	Major concerns	No concerns	No concerns	Low	["Imprecision"]
A:H	1	Some concerns	Low risk	No concerns	No concerns	Major concerns	No concerns	Low	["Heterogeneity"]
A:K	14	Some concerns	Low risk	No concerns	No concerns	Some concerns	Some concerns	Moderate	["Heterogeneity"]
B:D	1	Some concerns	Low risk	Some concerns	Major concerns	No concerns	No concerns	Very low	["Indirectness","Imprecision"]
B:G	1	Some concerns	Low risk	No concerns	Major concerns	No concerns	No concerns	Low	["Imprecision"]
B:K	3	Some concerns	Low risk	No concerns	No concerns	Major concerns	No concerns	Low	["Heterogeneity"]
C:D	2	No concerns	Low risk	No concerns	Major concerns	No concerns	No concerns	Low	["Imprecision"]
D:F	1	No concerns	Low risk	No concerns	Some concerns	Some concerns	No concerns	Moderate	["Imprecision"]
D:G	1	Some concerns	Low risk	No concerns	Some concerns	Some concerns	No concerns	Moderate	["Imprecision"]
D:K	8	Some concerns	Low risk	No concerns	No concerns	Major concerns	Major concerns	Low	["Heterogeneity"]
E:J	9	No concerns	Low risk	Some concerns	Major concerns	No concerns	No concerns	Very low	["Indirectness","Imprecision"]
E:K	1	Some concerns	Low risk	Some concerns	Major concerns	No concerns	No concerns	Very low	["Indirectness","Imprecision"]
F:G	1	Some concerns	Low risk	No concerns	Major concerns	No concerns	No concerns	Low	["Imprecision"]
F:H	3	No concerns	Low risk	No concerns	No concerns	Major concerns	Major concerns	Low	["Heterogeneity"]
F:K	8	No concerns	Low risk	No concerns	No concerns	Major concerns	No concerns	Low	["Heterogeneity"]

G:K	7	Some concerns	Low risk	No concerns	No concerns	Major concerns	No concerns	Low	["Heterogeneity"]
H:K	3	No concerns	Low risk	No concerns	Major concerns	No concerns	No concerns	Low	["Imprecision"]
J:K	1	Some concerns	Low risk	Some concerns	Major concerns	No concerns	No concerns	Very low	["Indirectness","Imprecision"]
A:B	0	Some concerns	Low risk	No concerns	Major concerns	No concerns	No concerns	Low	["Imprecision"]
A:C	0	No concerns	Low risk	No concerns	Major concerns	No concerns	No concerns	Low	["Imprecision"]
A:E	0	Some concerns	Low risk	Some concerns	Major concerns	No concerns	No concerns	Very low	["Indirectness","Imprecision"]
A:J	0	Some concerns	Low risk	Some concerns	Major concerns	No concerns	No concerns	Very low	["Indirectness","Imprecision"]
B:C	0	No concerns	Low risk	No concerns	Major concerns	No concerns	No concerns	Low	["Imprecision"]
B:E	0	Some concerns	Low risk	Some concerns	Major concerns	No concerns	No concerns	Very low	["Indirectness","Imprecision"]
B:F	0	Some concerns	Low risk	Some concerns	Major concerns	No concerns	No concerns	Very low	["Indirectness","Imprecision"]
B:H	0	Some concerns	Low risk	No concerns	Some concerns	Some concerns	No concerns	Moderate	["Imprecision"]
B:J	0	Some concerns	Low risk	Some concerns	Major concerns	No concerns	No concerns	Very low	["Indirectness","Imprecision"]
C:E	0	Some concerns	Low risk	No concerns	Major concerns	No concerns	No concerns	Low	["Imprecision"]
C:F	0	No concerns	Low risk	No concerns	Major concerns	No concerns	No concerns	Low	["Imprecision"]
C:G	0	No concerns	Low risk	No concerns	Major concerns	No concerns	No concerns	Low	["Imprecision"]
C:H	0	No concerns	Low risk	No concerns	Major concerns	No concerns	No concerns	Low	["Imprecision"]
C:J	0	Some concerns	Low risk	No concerns	Major concerns	No concerns	No concerns	Low	["Imprecision"]
C:K	0	No concerns	Low risk	No concerns	Major concerns	No concerns	No concerns	Low	["Imprecision"]
D:E	0	Some concerns	Low risk	Some concerns	Major concerns	No concerns	No concerns	Very low	["Indirectness","Imprecision"]

**D:H**	0	No concerns	Low risk	No concerns	Some concerns	Some concerns	No concerns	Moderate	["Imprecision"]
**D:J**	0	Some concerns	Low risk	Some concerns	Major concerns	No concerns	No concerns	Very low	["Indirectness","Imprecision"]
**E:F**	0	Some concerns	Low risk	Some concerns	Major concerns	No concerns	No concerns	Very low	["Indirectness","Imprecision"]
**E:G**	0	Some concerns	Low risk	Some concerns	Major concerns	No concerns	No concerns	Very low	["Indirectness","Imprecision"]
**E:H**	0	Some concerns	Low risk	No concerns	Major concerns	No concerns	No concerns	Low	["Imprecision"]
**F:J**	0	Some concerns	Low risk	Some concerns	Major concerns	No concerns	No concerns	Very low	["Indirectness","Imprecision"]
**G:H**	0	Some concerns	Low risk	No concerns	No concerns	Major concerns	No concerns	Low	["Heterogeneity"]
**G:J**	0	Some concerns	Low risk	Some concerns	Major concerns	No concerns	No concerns	Very low	["Indirectness","Imprecision"]
**H:J**	0	Some concerns	Low risk	No concerns	Major concerns	No concerns	No concerns	Low	["Imprecision"]

A = Mediterranean diet; **B** = Dietary Approaches to Stop Hypertension; **C** = Paleo diet; **D** = Dietary guidelines-based diets; **E** = low GI / GL diet; **F** = plant-based diets; **G** = low fat diet; **H** = low carbohydrate high fat diet; **I** = traditional Mexican diet; **J** = high GI / GL diet; **K** = western habitual diet

Confidence ratings were moderate (13%), low (53%) and very low (33%).

Supplementary Table S64. The Confidence in Network Meta-Analysis (CINeMA) framework ratings for HDL-c

Comparison	Number of studies	Within-study bias	Reporting bias	Indirectness	Imprecision	Heterogeneity	Incoherence	Confidence rating	Reason(s) for downgrading
A:D	1	Some concerns	Low risk	Some concerns	No concerns	Some concerns	Some concerns	Moderate	["Indirectness"]
A:F	1	Some concerns	Low risk	No concerns	No concerns	Some concerns	No concerns	Moderate	["Within-study bias"]
A:G	10	Some concerns	Low risk	No concerns	No concerns	No concerns	Some concerns	Moderate	["Within-study bias"]
A:H	1	Some concerns	Low risk	No concerns	No concerns	No concerns	No concerns	Moderate	["Within-study bias"]
A:K	14	Some concerns	Low risk	No concerns	No concerns	No concerns	No concerns	Moderate	["Within-study bias"]
B:D	1	Some concerns	Low risk	Some concerns	No concerns	No concerns	No concerns	Moderate	["Indirectness"]
B:G	1	Some concerns	Low risk	Some concerns	No concerns	No concerns	No concerns	Moderate	["Indirectness"]
B:K	3	Some concerns	Low risk	No concerns	No concerns	No concerns	No concerns	Moderate	["Within-study bias"]
C:D	2	No concerns	Low risk	No concerns	Some concerns	No concerns	Major concerns	Low	["Incoherence"]
D:F	1	No concerns	Low risk	No concerns	No concerns	No concerns	No concerns	High	[]
D:G	1	Some concerns	Low risk	Some concerns	No concerns	No concerns	No concerns	Low	["Within-study bias","Indirectness"]
D:K	9	Some concerns	Low risk	No concerns	No concerns	No concerns	Some concerns	Moderate	["Within-study bias"]
E:J	10	No concerns	Low risk	Some concerns	No concerns	No concerns	Major concerns	Very low	["Indirectness","Incoherence"]
E:K	1	Some concerns	Low risk	Some concerns	Major concerns	No concerns	No concerns	Very low	["Indirectness","Imprecision"]
F:G	1	No concerns	Low risk	Some concerns	No concerns	No concerns	Some concerns	Low	["Indirectness","Incoherence"]
F:H	3	No concerns	Low risk	No concerns	No concerns	No concerns	No concerns	High	[]
F:K	8	No concerns	Low risk	No concerns	No concerns	No concerns	Some concerns	Moderate	["Incoherence"]

G:K	7	Some concerns	Low risk	No concerns	No concerns	No concerns	No concerns	Moderate	["Within-study bias"]
H:K	3	No concerns	Low risk	No concerns	No concerns	Some concerns	No concerns	Moderate	["Heterogeneity"]
J:K	1	Some concerns	Low risk	Some concerns	Major concerns	No concerns	No concerns	Very low	["Indirectness","Imprecision"]
A:B	0	Some concerns	Low risk	No concerns	No concerns	No concerns	Major concerns	Low	["Incoherence"]
A:C	0	No concerns	Low risk	No concerns	Some concerns	No concerns	Major concerns	Low	["Incoherence"]
A:E	0	Some concerns	Low risk	Some concerns	Major concerns	No concerns	Major concerns	Very low	["Indirectness","Imprecision"]
A:J	0	Some concerns	Low risk	Some concerns	Major concerns	No concerns	Major concerns	Very low	["Indirectness","Imprecision"]
B:C	0	Some concerns	Low risk	No concerns	Some concerns	No concerns	Major concerns	Low	["Incoherence"]
B:E	0	Some concerns	Low risk	Some concerns	Major concerns	No concerns	Major concerns	Very low	["Indirectness","Imprecision"]
B:F	0	Some concerns	Low risk	Some concerns	No concerns	No concerns	Major concerns	Very low	["Indirectness","Incoherence"]
B:H	0	Some concerns	Low risk	No concerns	No concerns	No concerns	Major concerns	Low	["Incoherence"]
B:J	0	Some concerns	Low risk	Some concerns	Major concerns	No concerns	Major concerns	Very low	["Indirectness","Imprecision"]
C:E	0	Some concerns	Low risk	No concerns	Major concerns	No concerns	Major concerns	Low	["Imprecision"]
C:F	0	No concerns	Low risk	No concerns	Some concerns	No concerns	Major concerns	Low	["Incoherence"]
C:G	0	No concerns	Low risk	No concerns	Some concerns	No concerns	Major concerns	Low	["Incoherence"]
C:H	0	No concerns	Low risk	No concerns	Some concerns	Some concerns	Major concerns	Low	["Incoherence"]
C:J	0	Some concerns	Low risk	No concerns	Major concerns	No concerns	Major concerns	Low	["Imprecision"]
C:K	0	No concerns	Low risk	No concerns	Major concerns	No concerns	Major concerns	Low	["Imprecision"]
D:E	0	Some concerns	Low risk	Some concerns	Major concerns	No concerns	Major concerns	Very low	["Indirectness","Imprecision"]

**D:H**	0	No concerns	Low risk	No concerns	No concerns	No concerns	Major concerns	Low	["Incoherence"]
**D:J**	0	Some concerns	Low risk	Some concerns	Major concerns	No concerns	Major concerns	Very low	["Indirectness","Imprecision"]
**E:F**	0	Some concerns	Low risk	Some concerns	Major concerns	No concerns	Major concerns	Very low	["Indirectness","Imprecision"]
**E:G**	0	Some concerns	Low risk	Some concerns	Major concerns	No concerns	Major concerns	Very low	["Indirectness","Imprecision"]
**E:H**	0	Some concerns	Low risk	Some concerns	Major concerns	No concerns	Major concerns	Very low	["Indirectness","Imprecision"]
**F:J**	0	Some concerns	Low risk	Some concerns	Major concerns	No concerns	Major concerns	Very low	["Indirectness","Imprecision"]
**G:H**	0	No concerns	Low risk	No concerns	No concerns	No concerns	Major concerns	Low	["Incoherence"]
**G:J**	0	Some concerns	Low risk	Some concerns	Major concerns	No concerns	Major concerns	Very low	["Indirectness","Imprecision"]
**H:J**	0	Some concerns	Low risk	Some concerns	Major concerns	No concerns	Major concerns	Very low	["Indirectness","Imprecision"]

A = Mediterranean diet; **B** = Dietary Approaches to Stop Hypertension; **C** = Paleo diet; **D** = Dietary guidelines-based diets; **E** = low GI / GL diet; **F** = plant-based diets; **G** = low fat diet; **H** = low carbohydrate high fat diet; **I** = traditional Mexican diet; **J** = high GI / GL diet; **K** = western habitual diet

Confidence ratings were high (4%), moderate (27%), low (33%) and very low (36%).

Supplementary Table S65. The Confidence in Network Meta-Analysis (CINeMA) framework ratings for total cholesterol

Comparison	Number of studies	Within-study bias	Reporting bias	Indirectness	Imprecision	Heterogeneity	Incoherence	Confidence rating	Reason(s) for downgrading
A:D	1	Some concerns	Low risk	No concerns	Major concerns	No concerns	Some concerns	Low	["Imprecision"]
A:F	1	Some concerns	Low risk	No concerns	Major concerns	No concerns	No concerns	Low	["Imprecision"]
A:G	10	Some concerns	Low risk	No concerns	Major concerns	No concerns	No concerns	Low	["Imprecision"]
A:H	1	Some concerns	Low risk	No concerns	No concerns	Major concerns	No concerns	Low	["Heterogeneity"]
A:K	14	Some concerns	Low risk	No concerns	No concerns	Major concerns	Some concerns	Low	["Heterogeneity"]
B:D	1	Some concerns	Low risk	Some concerns	Major concerns	No concerns	No concerns	Very low	["Indirectness","Imprecision"]
B:G	1	Some concerns	Low risk	No concerns	Major concerns	No concerns	No concerns	Low	["Imprecision"]
B:K	3	Some concerns	Low risk	No concerns	No concerns	Major concerns	No concerns	Low	["Heterogeneity"]
C:D	2	No concerns	Low risk	No concerns	Major concerns	No concerns	No concerns	Low	["Imprecision"]
D:F	1	No concerns	Low risk	No concerns	Major concerns	No concerns	No concerns	Low	["Imprecision"]
D:G	1	Some concerns	Low risk	No concerns	Major concerns	No concerns	No concerns	Low	["Imprecision"]
D:K	9	No concerns	Low risk	No concerns	No concerns	Major concerns	Major concerns	Low	["Heterogeneity"]
E:J	8	Some concerns	Low risk	No concerns	Major concerns	No concerns	No concerns	Low	["Imprecision"]
E:K	1	Some concerns	Low risk	Some concerns	Major concerns	No concerns	No concerns	Very low	["Indirectness","Imprecision"]
F:G	1	Some concerns	Low risk	No concerns	Major concerns	No concerns	No concerns	Low	["Imprecision"]
F:H	3	No concerns	Low risk	No concerns	No concerns	Major concerns	No concerns	Low	["Heterogeneity"]
F:K	8	No concerns	Low risk	No concerns	No concerns	Major concerns	No concerns	Low	["Heterogeneity"]

G:K	7	Some concerns	Low risk	No concerns	No concerns	Major concerns	No concerns	Low	["Heterogeneity"]
H:K	3	No concerns	Low risk	No concerns	Major concerns	No concerns	No concerns	Low	["Imprecision"]
J:K	1	Some concerns	Low risk	Some concerns	Major concerns	No concerns	No concerns	Very low	["Indirectness","Imprecision"]
A:B	0	Some concerns	Low risk	No concerns	Major concerns	No concerns	No concerns	Low	["Imprecision"]
A:C	0	No concerns	Low risk	No concerns	Major concerns	No concerns	No concerns	Low	["Imprecision"]
A:E	0	Some concerns	Low risk	Some concerns	Major concerns	No concerns	No concerns	Very low	["Indirectness","Imprecision"]
A:J	0	Some concerns	Low risk	Some concerns	Major concerns	No concerns	No concerns	Very low	["Indirectness","Imprecision"]
B:C	0	No concerns	Low risk	No concerns	Major concerns	No concerns	No concerns	Low	["Imprecision"]
B:E	0	Some concerns	Low risk	Some concerns	Major concerns	No concerns	No concerns	Very low	["Indirectness","Imprecision"]
B:F	0	Some concerns	Low risk	Some concerns	Major concerns	No concerns	No concerns	Very low	["Indirectness","Imprecision"]
B:H	0	Some concerns	Low risk	No concerns	No concerns	Major concerns	No concerns	Low	["Heterogeneity"]
B:J	0	Some concerns	Low risk	Some concerns	Major concerns	No concerns	No concerns	Very low	["Indirectness","Imprecision"]
C:E	0	Some concerns	Low risk	No concerns	Major concerns	No concerns	No concerns	Low	["Imprecision"]
C:F	0	No concerns	Low risk	No concerns	Major concerns	No concerns	No concerns	Low	["Imprecision"]
C:G	0	No concerns	Low risk	No concerns	Major concerns	No concerns	No concerns	Low	["Imprecision"]
C:H	0	No concerns	Low risk	No concerns	Major concerns	No concerns	No concerns	Low	["Imprecision"]
C:J	0	Some concerns	Low risk	No concerns	Major concerns	No concerns	No concerns	Low	["Imprecision"]
C:K	0	No concerns	Low risk	No concerns	Major concerns	No concerns	No concerns	Low	["Imprecision"]
D:E	0	Some concerns	Low risk	Some concerns	Major concerns	No concerns	No concerns	Very low	["Indirectness","Imprecision"]

D:H	0	No concerns	Low risk	No concerns	Some concerns	Some concerns	No concerns	Moderate	["Imprecision"]
D:J	0	Some concerns	Low risk	Some concerns	Major concerns	No concerns	No concerns	Very low	["Indirectness","Imprecision"]
E:F	0	Some concerns	Low risk	Some concerns	Major concerns	No concerns	No concerns	Very low	["Indirectness","Imprecision"]
E:G	0	Some concerns	Low risk	Some concerns	Major concerns	No concerns	No concerns	Very low	["Indirectness","Imprecision"]
E:H	0	Some concerns	Low risk	No concerns	Major concerns	No concerns	No concerns	Low	["Imprecision"]
F:J	0	Some concerns	Low risk	Some concerns	Major concerns	No concerns	No concerns	Very low	["Indirectness","Imprecision"]
G:H	0	Some concerns	Low risk	No concerns	No concerns	Major concerns	No concerns	Low	["Heterogeneity"]
G:J	0	Some concerns	Low risk	Some concerns	Major concerns	No concerns	No concerns	Very low	["Indirectness","Imprecision"]
H:J	0	Some concerns	Low risk	No concerns	Major concerns	No concerns	No concerns	Low	["Imprecision"]

A = Mediterranean diet; **B** = Dietary Approaches to Stop Hypertension; **C** = Paleo diet; **D** = Dietary guidelines-based diets; **E** = low GI / GL diet; **F** = plant-based diets; **G** = low fat diet; **H** = low carbohydrate high fat diet; **I** = traditional Mexican diet; **J** = high GI / GL diet; **K** = western habitual diet

Confidence ratings were moderate (2%), low (67%) and very low (31%).

Supplementary Table S66. The Confidence in Network Meta-Analysis (CINeMA) framework ratings for HOMA-IR

Comparison	Number of studies	Within-study bias	Reporting bias	Indirectness	Imprecision	Heterogeneity	Incoherence	Confidence rating	Reason(s) for downgrading
A:G	3	Some concerns	Some concerns	Some concerns	Major concerns	No concerns	No concerns	Very low	["Indirectness","Imprecision"]
A:K	3	Some concerns	Some concerns	No concerns	Major concerns	No concerns	No concerns	Low	["Imprecision"]
C:D	1	No concerns	Some concerns	No concerns	Major concerns	No concerns	No concerns	Low	["Imprecision"]
D:G	1	No concerns	Some concerns	No concerns	Major concerns	No concerns	No concerns	Low	["Imprecision"]
D:K	4	No concerns	Some concerns	No concerns	No concerns	Major concerns	No concerns	Low	["Heterogeneity"]
E:J	2	Some concerns	Some concerns	No concerns	Major concerns	No concerns	No concerns	Low	["Imprecision"]
E:K	1	Some concerns	Some concerns	Some concerns	Major concerns	No concerns	No concerns	Very low	["Indirectness","Imprecision"]
F:K	1	No concerns	Some concerns	No concerns	No concerns	No concerns	No concerns	Moderate	["Reporting bias"]
G:K	2	No concerns	Some concerns	No concerns	Major concerns	No concerns	No concerns	Low	["Imprecision"]
J:K	1	Some concerns	Some concerns	Some concerns	Major concerns	No concerns	No concerns	Very low	["Indirectness","Imprecision"]
A:C	0	No concerns	Some concerns	No concerns	Major concerns	No concerns	No concerns	Low	["Imprecision"]
A:D	0	Some concerns	Some concerns	No concerns	Major concerns	No concerns	No concerns	Low	["Imprecision"]
A:E	0	Some concerns	Some concerns	Some concerns	Major concerns	No concerns	No concerns	Very low	["Indirectness","Imprecision"]
A:F	0	No concerns	Some concerns	No concerns	No concerns	No concerns	No concerns	Moderate	[]
A:J	0	Some concerns	Some concerns	Some concerns	Major concerns	No concerns	No concerns	Very low	["Indirectness","Imprecision"]
C:E	0	No concerns	Some concerns	No concerns	Major concerns	No concerns	No concerns	Low	["Imprecision"]
C:F	0	No concerns	Some concerns	No concerns	Major concerns	No concerns	No concerns	Low	["Imprecision"]

C:G	0	No concerns	Some concerns	No concerns	No concerns	Major concerns	No concerns	Low	["Heterogeneity"]
C:J	0	No concerns	Some concerns	No concerns	Major concerns	No concerns	No concerns	Low	["Imprecision"]
C:K	0	No concerns	Some concerns	No concerns	No concerns	No concerns	No concerns	Moderate	["Reporting bias"]
D:E	0	Some concerns	Some concerns	No concerns	Major concerns	No concerns	No concerns	Low	["Imprecision"]
D:F	0	No concerns	Some concerns	No concerns	No concerns	Major concerns	No concerns	Low	["Heterogeneity"]
D:J	0	Some concerns	Some concerns	No concerns	Major concerns	No concerns	No concerns	Low	["Imprecision"]
E:F	0	Some concerns	Some concerns	No concerns	Major concerns	No concerns	No concerns	Low	["Imprecision"]
E:G	0	Some concerns	Some concerns	Some concerns	Major concerns	No concerns	No concerns	Very low	["Indirectness","Imprecision"]
F:G	0	No concerns	Some concerns	No concerns	No concerns	No concerns	No concerns	Moderate	["Reporting bias"]
F:J	0	Some concerns	Some concerns	No concerns	Major concerns	No concerns	No concerns	Low	["Imprecision"]
G:J	0	Some concerns	Some concerns	Some concerns	Major concerns	No concerns	No concerns	Very low	["Indirectness","Imprecision"]

A = Mediterranean diet; **B** = Dietary Approaches to Stop Hypertension; **C** = Paleo diet; **D** = Dietary guidelines-based diets; **E** = low GI / GL diet; **F** = plant-based diets; **G** = low fat diet; **H** = low carbohydrate high fat diet; **I** = traditional Mexican diet; **J** = high GI / GL diet; **K** = western habitual diet

Confidence ratings were moderate (14%), low (61%) and very low (25%).

Supplementary Table S67. The Confidence in Network Meta-Analysis (CINeMA) framework ratings for hsCRP

Comparison	Number of studies	Within-study bias	Reporting bias	Indirectness	Imprecision	Heterogeneity	Incoherence	Confidence rating	Reason(s) for downgrading
A:F	1	Some concerns	Some concerns	No concerns	Major concerns	No concerns	No concerns	Low	["Imprecision"]
A:G	3	Some concerns	Some concerns	Some concerns	Some concerns	Some concerns	No concerns	Low	["Indirectness","Imprecision"]
A:H	1	Some concerns	Some concerns	No concerns	Some concerns	Some concerns	No concerns	Moderate	["Imprecision"]
A:K	6	Some concerns	Some concerns	No concerns	Some concerns	Some concerns	No concerns	Moderate	["Imprecision"]
B:D	1	Some concerns	Some concerns	Some concerns	Major concerns	No concerns	No concerns	Very low	["Indirectness","Imprecision"]
C:D	2	No concerns	Some concerns	No concerns	Major concerns	No concerns	No concerns	Low	["Imprecision"]
D:G	1	No concerns	Some concerns	No concerns	No concerns	Some concerns	No concerns	Moderate	["Heterogeneity"]
D:K	8	No concerns	Some concerns	No concerns	Some concerns	Some concerns	No concerns	Moderate	["Imprecision"]
F:H	2	Some concerns	Some concerns	No concerns	Major concerns	No concerns	No concerns	Low	["Imprecision"]
F:K	2	Some concerns	Some concerns	No concerns	Some concerns	Some concerns	No concerns	Moderate	["Imprecision"]
G:K	1	Some concerns	Some concerns	No concerns	Some concerns	Some concerns	No concerns	Moderate	["Imprecision"]
H:K	2	Some concerns	Some concerns	No concerns	Major concerns	No concerns	No concerns	Low	["Imprecision"]
A:B	0	Some concerns	Some concerns	Some concerns	Major concerns	No concerns	No concerns	Very low	["Indirectness","Imprecision"]
A:C	0	No concerns	Some concerns	No concerns	Major concerns	No concerns	No concerns	Low	["Imprecision"]
A:D	0	Some concerns	Some concerns	No concerns	Some concerns	Some concerns	No concerns	Moderate	["Imprecision"]
B:C	0	Some concerns	Some concerns	Some concerns	Major concerns	No concerns	No concerns	Very low	["Indirectness","Imprecision"]
B:F	0	Some concerns	Some concerns	No concerns	Major concerns	No concerns	No concerns	Low	["Imprecision"]

B:G	0	Some concerns	Some concerns	Some concerns	Some concerns	Some concerns	No concerns	Low	["Indirectness","Imprecision"]
B:H	0	Some concerns	Some concerns	No concerns	Major concerns	No concerns	No concerns	Low	["Imprecision"]
B:K	0	Some concerns	Some concerns	Some concerns	Major concerns	No concerns	No concerns	Very low	["Indirectness","Imprecision"]
C:F	0	Some concerns	Some concerns	No concerns	Major concerns	No concerns	No concerns	Low	["Imprecision"]
C:G	0	No concerns	Some concerns	No concerns	Some concerns	Some concerns	No concerns	Moderate	["Imprecision"]
C:H	0	Some concerns	Some concerns	No concerns	Major concerns	No concerns	No concerns	Low	["Imprecision"]
C:K	0	No concerns	Some concerns	No concerns	Some concerns	Some concerns	No concerns	Moderate	["Imprecision"]
D:F	0	Some concerns	Some concerns	No concerns	Some concerns	Some concerns	No concerns	Moderate	["Imprecision"]
D:H	0	Some concerns	Some concerns	No concerns	Some concerns	Some concerns	No concerns	Moderate	["Imprecision"]
F:G	0	Some concerns	Some concerns	No concerns	Some concerns	Some concerns	No concerns	Moderate	["Imprecision"]
G:H	0	Some concerns	Some concerns	No concerns	Major concerns	No concerns	No concerns	Low	["Imprecision"]

A = Mediterranean diet; **B** = Dietary Approaches to Stop Hypertension; **C** = Paleo diet; **D** = Dietary guidelines-based diets; **E** = low GI / GL diet; **F** = plant-based diets; **G** = low fat diet; **H** = low carbohydrate high fat diet; **I** = traditional Mexican diet; **J** = high GI / GL diet; **K** = western habitual diet

Confidence ratings were moderate (43%), low (43%) and very low (14%).

Supplementary Figure S37. Right-angle Mixture Triangles (RMTs) for total cholesterol[1]

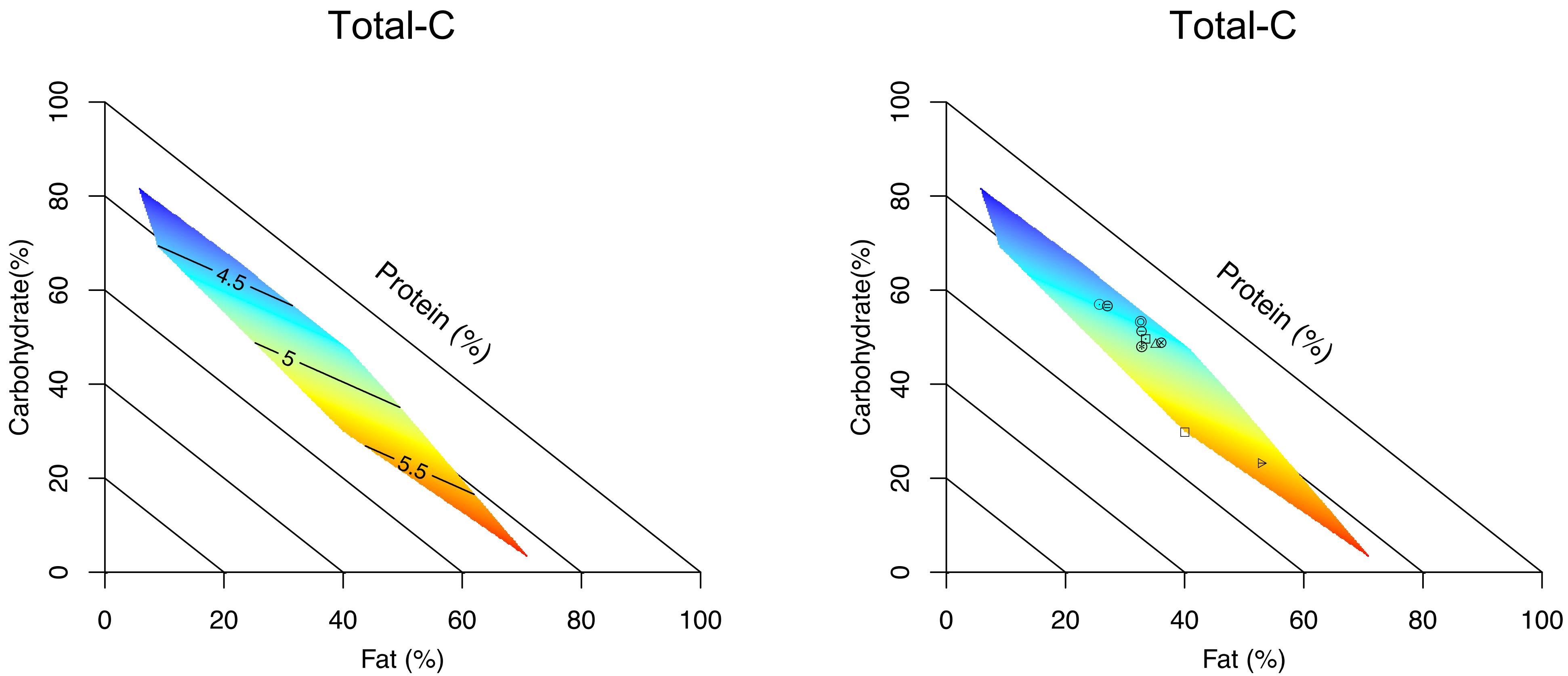

△ = Mediterranean diet; ⊙ = Dietary Approaches to Stop Hypertension; □ = Paleo diet; ⊡ = Dietary guidelines-based diets; ⊖ = low GI / GL diet; ◎ = plant-based diets; ⊛ = low fat diet; ⊳ = low carbohydrate high fat diet; ⊜ = high GI / GL diet; ⊗ = western habitual diet

The symbols on the response surfaces correspond to the average macronutrient composition of each dietary pattern.

[1]Response surfaces with contour lines show the predicted effects superimposed onto a composition triangle for total cholesterol (mmol/L). Each space on these RMTs represents 100% of dietary energy, being the sum of the x-axis (fat), the y-axis (carbohydrate), and an inferred z-axis (protein). A diagonal line closer to the origin corresponds to higher protein (%) intake. The distribution of the surface limited to the intakes observed in the included dietary studies.

Supplementary Figure S38. Right-angle Mixture Triangles (RMTs) for HDL-c[1]

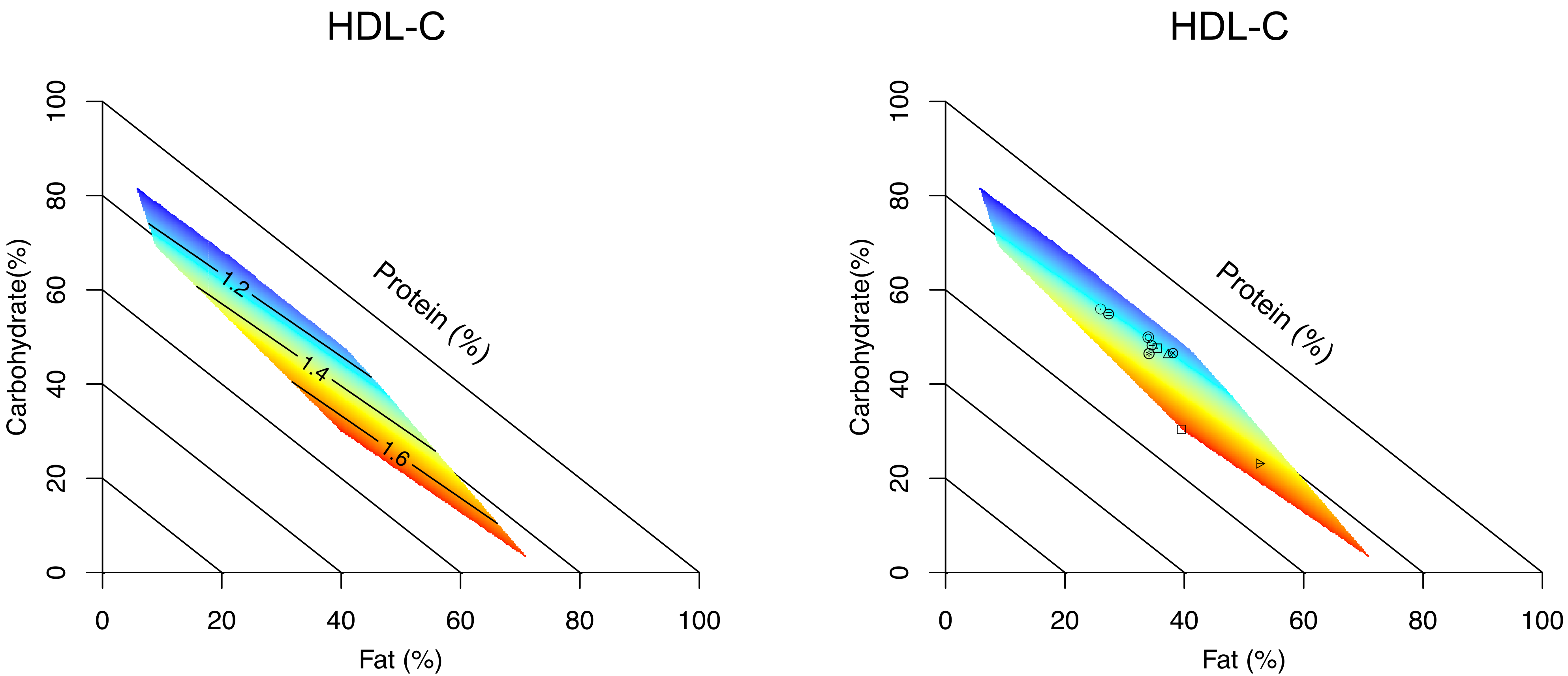

△ = Mediterranean diet; ⊙ = Dietary Approaches to Stop Hypertension; □ = Paleo diet; ⊡ = Dietary guidelines-based diets; ⊖ = low GI / GL diet; ◎ = plant-based diets; ⊛ = low fat diet; ⊳ = low carbohydrate high fat diet; ⊜ = high GI / GL diet; ⊗ = western habitual diet

The symbols on the response surfaces correspond to the average macronutrient composition of each dietary pattern.

[1]Response surfaces with contour lines show the predicted effects superimposed onto a composition triangle for HDL-c (mmol/L). Each space on these RMTs represents 100% of dietary energy, being the sum of the x-axis (fat), the y-axis (carbohydrate), and an inferred z-axis (protein). A diagonal line closer to the origin corresponds to higher intake. The distribution of the surface limited to the intakes observed in the included dietary studies.

Supplementary Figure S39. Right-angle Mixture Triangles (RMTs) for ApoA1[1]

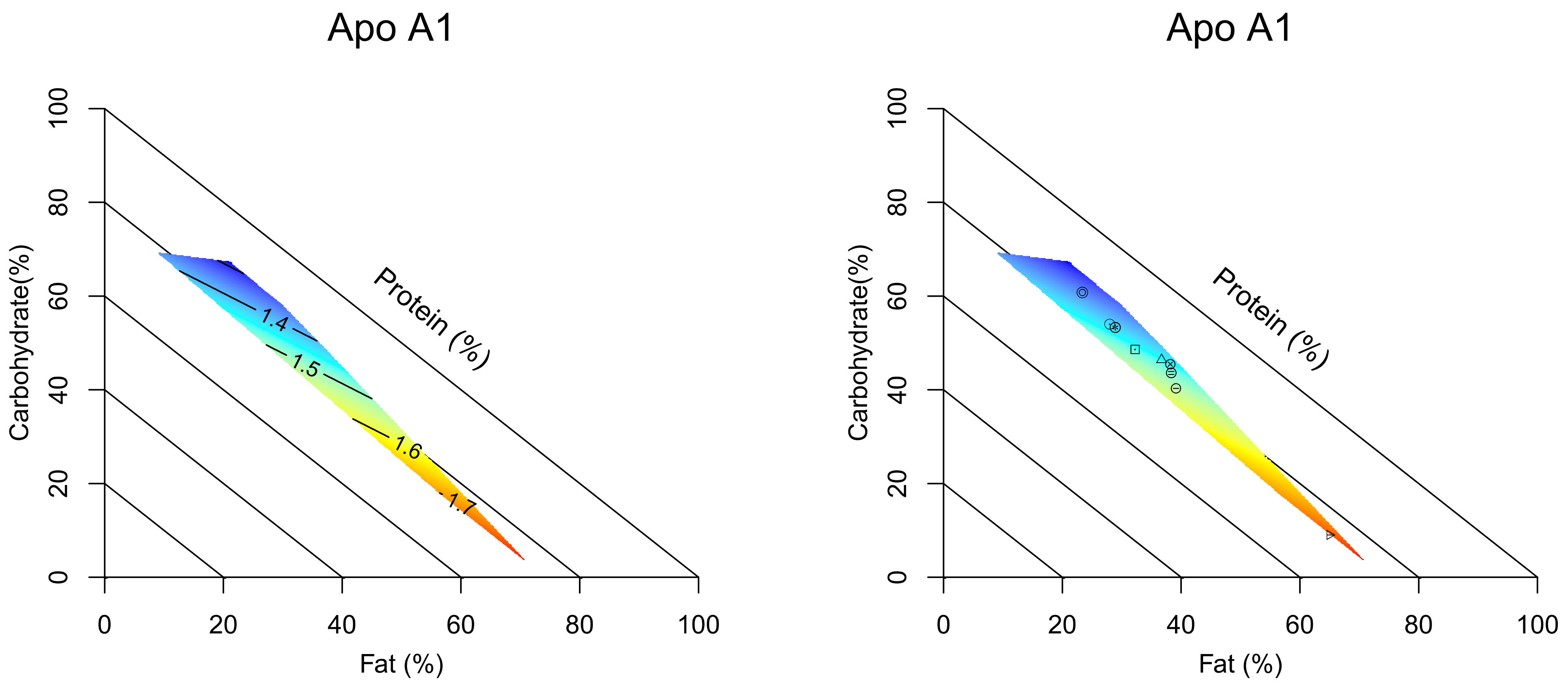

△ = Mediterranean diet; ⊙ = Dietary Approaches to Stop Hypertension; ⊡ = Dietary guidelines-based diets; ⊖ = low GI / GL diet; ◎ = plant-based diets; ⊛ = low fat diet; ⊳ = low carbohydrate high fat diet; ⊜ = high GI / GL diet; ⊗ = western habitual diet

The symbols on the response surfaces correspond to the average macronutrient composition of each dietary pattern.

[1]Response surfaces with contour lines show the predicted effects superimposed onto a composition triangle for ApoA1 (g/L). Each space on these RMTs represents 100% of dietary energy, being the sum of the x-axis (fat), the y-axis (carbohydrate), and an inferred z-axis (protein). A diagonal line closer to the origin corresponds to higher intake. The distribution of the surface limited to the intakes observed in the included dietary studies.

Supplementary Table S68. Coefficients for macronutrient and total cholesterol, HDL-c and ApoA1 Right-angle Mixture Triangles[1]

Total cholesterol: Model 2	Estimate	Standard Error	t-value	p-value
% Protein	7.7451	2.2622	3.4236	0.0009
% Carbohydrate	3.3866	0.4866	6.9603	<0.0001
% Fat	5.2888	0.9021	5.8625	<0.0001
HDL-c: Model 2	Estimate	Standard Error	t-value	p-value
% Protein	3.7677	0.6088	6.1884	<0.0001
% Carbohydrate	0.5904	0.131	4.5069	<0.0001
% Fat	0.9922	0.2459	4.0344	0.0001
ApoA1: Model 2	Estimate	Standard Error	t-value	p-value
% Protein	2.5522	0.6504	3.9241	0.0004
% Carbohydrate	0.9847	0.1278	7.7061	<0.0001
% Fat	1.5414	0.2421	6.3668	<0.0001

[1]Model 2 represents linear associations between macronutrient composition and biomarker outcomes.

eReferences

1. Abedi P, Lee MHS, Kandiah M, et al. (2010) Diet intervention to improve cardiovascular risk factors among Iranian postmenopausal women. Nutrition research and practice 4(6):522−7. doi:10.4162/nrp.2010.4.6.522
2. Adamsson V, Reumark A, Fredriksson IB, et al. (2010) Effects of a healthy Nordic diet on cardiovascular risk factors in hypercholesterolaemic subjects: a randomized controlled trial (NORDIET). Journal of internal medicine 269(2):150−9. doi:10.1111/j.1365−2796.2010.02290.x
3. Ambring A, Johansson M, Axelsen M, Gan L, Strandvik B, Friberg P (2006) Mediterranean−inspired diet lowers the ratio of serum phospholipid *n*−6 to *n*−3 fatty acids, the number of leukocytes and platelets, and vascular endothelial growth factor in healthy subjects. American Journal of Clinical Nutrition 83(3):575−81.
4. Blomquist C, Alvehus M, Buren J, et al. (2017) Attenuated Low−Grade Inflammation Following Long−Term Dietary Intervention in Postmenopausal Women with Obesity. Obesity (Silver Spring) 25(5):892−900. doi:10.1002/oby.21815
5. Bos MB, de Vries JHM, Feskens EJM, et al. (2010) Effect of a high monounsaturated fatty acids diet and a Mediterranean diet on serum lipids and insulin sensitivity in adults with mild abdominal obesity. Nutrition, metabolism, and cardiovascular diseases 20(8):591−8. doi:10.1016/j.numecd.2009.05.008
6. Botero D, Ebbeling CB, Blumberg JB, et al. (2009) Acute effects of dietary glycemic index on antioxidant capacity in a nutrient−controlled feeding study. Obesity (Silver Spring) 17(9):1664−70. doi:https://dx.doi.org/10.1038/oby.2009.203
7. Bouché C, Rizkalla SW, Luo J, et al. (2002) Five−Week, Low−Glycemic Index Diet Decreases Total Fat Mass and Improves Plasma Lipid Profile in Moderately Overweight Nondiabetic Men. Diabetes Care 25(5):822−8. doi:10.2337/diacare.25.5.822
8. Bravo−Herrera MD, López−Miranda J, Marín C, et al. (2004) Tissue factor expression is decreased in monocytes obtained from blood during Mediterranean or high carbohydrate diets. Nutrition, metabolism, and cardiovascular diseases 14(3):128−32. doi:10.1016/S0939−4753(04)80032−2
9. Brynes AE, Edwards CM, Ghatei MA, et al. (2003) A randomised four−intervention crossover study investigating the effect of carbohydrates on daytime profiles of insulin, glucose, non−esterified fatty acids and triacylglycerols in middle−aged men. British Journal of Nutrition 89(2):207−18. doi:http://dx.doi.org/10.1079/BJN2002769
10. Casas R, Sacanella E, Urpi−Sarda M, et al. (2014) The effects of the Mediterranean diet on biomarkers of vascular wall inflammation and plaque vulnerability in subjects with high risk for cardiovascular disease. A randomized trial. Plos one 9(6).
11. Casas R, Sacanella E, Urpi−Sarda M, et al. (2016) Long−term immunomodulatory effects of a mediterranean diet in adults at high risk of cardiovascular disease in the prevencion con dieta mediterranea (predimed) randomized controlled trial. Journal of Nutrition 146(9):1684−93. doi:http://dx.doi.org/10.3945/jn.115.229476
12. Casas R, Urpi−Sarda M, Sacanella E, et al. (2017) Anti−Inflammatory Effects of the Mediterranean Diet in the Early and Late Stages of Atheroma Plaque Development. Mediators of Inflammation 2017(3674390). doi:http://dx.doi.org/10.1155/2017/3674390
13. Chen ST, Maruthur NM, Appel LJ (2010) The effect of dietary patterns on estimated coronary heart disease risk: results from the Dietary Approaches to Stop Hypertension (DASH) trial. Circ Cardiovasc Qual Outcomes 3(5):484−9. doi:10.1161/CIRCOUTCOMES.109.930685
14. Clapp JF, Lopez B (2007) Low−versus high−glycemic index diets in women: effects on caloric requirement, substrate utilization and insulin sensitivity. Metabolic syndrome and related disorders 5(3):231−42. doi:10.1089/met.2006.0040
15. Cooper RS, Goldberg RB, Trevisan M, et al. (1982) The selective lipid−lowering effect of vegetarianism on low density lipoproteins in a cross−over experiment. Atherosclerosis 44(3):293−305. doi:10.1016/0021−9150(82)90004−1
16. Dansinger ML, Gleason JA, Griffith JL, Selker HP, Schaefer EJ (2005) Comparison of the Atkins, Ornish, Weight Watchers, and Zone Diets for Weight Loss and Heart Disease Risk Reduction: A Randomized Trial. JAMA : the journal of the American Medical Association 293(1):43−53. doi:10.1001/jama.293.1.43
17. Davis CR, Bryan J, Hodgson JM, Woodman R, Murphy KJ (2017) A Mediterranean diet reduces F2−Isoprostanes and triglycerides among older Australian men and women after 6 months. Journal of Nutrition 147(7):1348−55. doi:http://dx.doi.org/10.3945/jn.117.248419
18. de Rougemont A, Normand S, Nazare J−A, et al. (2007) Beneficial effects of a 5−week low−glycaemic index regimen on weight control and cardiovascular risk factors in overweight non−diabetic subjects. British journal of nutrition 98(6):1288−98. doi:10.1017/S0007114507778674
19. Djuric Z, Ren J, Blythe J, VanLoon G, Sen A (2009) A Mediterranean dietary intervention in healthy American women changes plasma carotenoids and fatty acids in distinct clusters. Nutrition research (New York, *n*.Y.) 29(3):156−63. doi:10.1016/j.nutres.2009.03.001

20. Due A, Larsen TM, Huiling MU, Hermansen K, Stender S, Astrup A (2008) Comparison of 3 ad libitum diets for weight−loss maintenance, risk of cardiovascular disease, and diabetes : a 6−mo randomized, controlled trial. The American journal of clinical nutrition 88(5):1232−41.
21. Fernández−Real JM, Bulló M, Moreno−Navarrete JM, et al. (2012) A mediterranean diet enriched with olive oil is associated with higher serum total osteocalcin levels in elderly men at high cardiovascular risk. Journal of Clinical Endocrinology & Metabolism 97(10):3792−8. doi:10.1210/jc.2012−2221
22. Fuentes F, Lopez−Miranda J, Perez−Martinez P, et al. (2008) Chronic effects of a high−fat diet enriched with virgin olive oil and a low−fat diet enriched with alpha−linolenic acid on postprandial endothelial function in healthy men. British Journal of Nutrition 100(1):159−65. doi:http://dx.doi.org/10.1017/S0007114508888708
23. Gardner CD, Coulston A, Chatterjee L, Rigby A, Spiller G, Farquhar JW (2005) The effect of a plant−based diet on plasma lipids in hypercholesterolemic adults : A randomized trial. Ann Intern Med 142(9):725−33. doi:10.7326/0003−4819−142−9−200505030−00007
24. Gardner CD, Kiazand A, Alhassan S, et al. (2007) Comparison of the Atkins, Zone, Ornish, and LEARN Diets for Change in Weight and Related Risk Factors Among Overweight Premenopausal Women: The A TO Z Weight Loss Study: A Randomized Trial. JAMA : the journal of the American Medical Association 297(9):969−77. doi:10.1001/jama.297.9.969
25. Genoni A, Lyons−Wall P, Lo J, Devine A (2016) Cardiovascular, Metabolic Effects and Dietary Composition of Ad−Libitum Paleolithic vs. Australian Guide to Healthy Eating Diets: A 4−Week Randomised Trial. Nutrients 8(5):23. doi:https://dx.doi.org/10.3390/nu8050314
26. Hinderliter AL, Hinderliter AL, Babyak MA, et al. (2011) The DASH Diet and Insulin Sensitivity. Current hypertension reports 13(1):67−73. doi:10.1007/s11906−010−0168−5
27. Howard BV, Van Horn L, Hsia J, et al. (2006) Low−Fat Dietary Pattern and Risk of Cardiovascular Disease: The Women's Health Initiative Randomized Controlled Dietary Modification Trial. JAMA : the journal of the American Medical Association 295(6):655−66. doi:10.1001/jama.295.6.655
28. Hunt JR, Matthys LA, Johnson LK (1998) Zinc absorption, mineral balance, and blood lipids in women consuming controlled lactoovovegetarian and
omnivorous diets for 8 wk. American Journal of Clinical Nutrition 67:421−30.
29. Hunt JR, Roughead ZK (1999) Nonheme−iron absorption, fecal ferritin excretion, and blood indexes of iron status in women consuming controlled lactoovovegetarian diets for 8 wk. The American journal of clinical nutrition 69(5):944−52.
30. Jenkins DJA, Wolever TMS, Collier GR, et al. (1987) Metabolic effects of a low−glycemic−index diet. The American journal of clinical nutrition 46(6):968−75. doi:10.1093/ajcn/46.6.968
31. Jensen L, Sloth B, Krog−Mikkelsen I, et al. (2008) A low−glycemic−index diet reduces plasma plasminogen activator inhibitor−1 activity, but not tissue inhibitor of proteinases−1 or plasminogen activator inhibitor−1 protein, in overweight women. The American journal of clinical nutrition 87(1):97−105. doi:10.1093/ajcn/87.1.97
32. Kahleova H, Dort S, Holubkov R, Barnard ND (2018) A Plant−Based High−Carbohydrate, Low−Fat Diet in Overweight Individuals in a 16−Week Randomized Clinical Trial: The Role of Carbohydrates. Nutrients 10(9):1302. doi:10.3390/nu10091302
33. Kestin M, Rouse IL, Correll RA, Nestel PJ (1989) Cardiovascular disease risk factors in free−living men: Comparison of two prudent diets, one based on lactoovovegetarianism and the other allowing lean meat. The American journal of clinical nutrition 50(2):280−7. doi:10.1093/ajcn/50.2.280
34. Kim Y, Keogh JB, Clifton PM (2017) Effects of Two Different Dietary Patterns on Inflammatory Markers, Advanced Glycation End Products and Lipids in Subjects without Type 2 Diabetes: A Randomised Crossover Study. Nutrients 9(4):29. doi:https://dx.doi.org/10.3390/nu9040336
35. Konstantinidou V, Covas MI, Munoz−Aguayo D, et al. (2010) In vivo nutrigenomic effects of virgin olive oil polyphenols within the frame of the Mediterranean diet: a randomized controlled trial. FASEB J 24(7):2546−57. doi:10.1096/fj.09−148452
36. Ling WH, Laitinen M, Hänninen O (1992) Shifting from conventional diet to an uncooked vegan diet reversibly alters serum lipid and apolipoprotein levels. Nutrition research (New York, *n*.Y.) 12(12):1431−40. doi:10.1016/S0271−5317(05)80185−X
37. Marin C, Ramirez R, Delgado−Lista J, et al. (2011) Mediterranean diet reduces endothelial damage and improves the regenerative capacity of endothelium. American Journal of Clinical Nutrition 93(2):267−74. doi:10.3945/ajcn.110.006866
38. Mellberg C, Sandberg S, Ryberg M, et al. (2014) Long−term effects of a Palaeolithic−type diet in obese postmenopausal women: a 2−year randomized trial. Eur J Clin Nutr 68(3):350−7. doi:10.1038/ejcn.2013.290
39. Meslier V, Laiola M, Roager HM, et al. (2020) Mediterranean diet intervention in overweight and obese subjects lowers plasma cholesterol and causes changes in the gut microbiome and metabolome independently of energy intake. Gut 69(7):1258−68. doi:http://dx.doi.org/10.1136/gutjnl−2019−320438

40. Miller ER, 3rd, Erlinger TP, Sacks FM, et al. (2005) A dietary pattern that lowers oxidative stress increases antibodies to oxidized LDL: results from a randomized controlled feeding study. Atherosclerosis 183(1):175−82.
41. Miller M, Beach V, Sorkin JD, et al. (2009) Comparative effects of three popular diets on lipids, endothelial function, and C−reactive protein during weight maintenance. J Am Diet Assoc 109(4):713−7. doi:10.1016/j.jada.2008.12.023
42. Mishra S, Xu J, Agarwal U, Gonzales J, Levin S, Barnard ND (2013) A multicenter randomized controlled trial of a plant−based nutrition program to reduce body weight and cardiovascular risk in the corporate setting: the GEICO study. European journal of clinical nutrition 67(7):718−24. doi:10.1038/ejcn.2013.92
43. Mohammadshahi M, Haidari F, Karandish M, Ebrahimi S, Haghighizadeh MH (2014) A randomized clinical trial of nutrition education for improvement of diet quality and inflammation in Iranian obese women. J Nutr Metab 2014:605782. doi:https://dx.doi.org/10.1155/2014/605782
44. Morgantini C, Trifiro S, Trico D, et al. (2018) A short−term increase in dietary cholesterol and fat intake affects high−density lipoprotein composition in healthy subjects. Nutrition, metabolism, and cardiovascular diseases : NMCD 28(6):575−81. doi:https://dx.doi.org/10.1016/j.numecd.2018.03.005
45. Neuhouser ML, Schwarz Y, Wang C, et al. (2012) A low−glycemic load diet reduces serum C−reactive protein and modestly increases adiponectin in overweight and obese adults. Journal of Nutrition 142(2):369−74. doi:10.3945/jn.111.149807
46. Nowson CA, Worsley A, Margerison C, Jorna MK, Godfrey SJ, Booth A (2005) Blood pressure change with weight loss is affected by diet type in men. The American journal of clinical nutrition 81(9):983−9.
47. Paniagua JA, Sacristana AGdl, Sanchez E, et al. (2007) A Mufa−Rich Diet Improves Posprandial GLucose, Lipid and Glp−1 Responses in Insulin−Resistant Subjects. Journal of the American College of Nutrition 26(5):434−44.
48. Pérez−Jiménez F, López−Miranda J, Pinillos MD, et al. (2001) A Mediterranean and a high−carbohydrate diet improve glucose metabolism in healthy young persons. Diabetologia 44(11):2038−43. doi:10.1007/s001250100009
49. Perez−Martinez P, Lopez−Miranda J, Blanco−Colio L, et al. (2006) The chronic intake of a Mediterranean diet enriched in virgin olive oil, decreases nuclear transcription factor κB activation in peripheral blood mononuclear cells from healthy men. Atherosclerosis 194(2):e141−e6. doi:10.1016/j.atherosclerosis.2006.11.033
50. Poulsen SK, Due A, Jordy AB, et al. (2014) Health effect of the New Nordic Diet in adults with increased waist circumference: a 6−mo randomized controlled trial. The American journal of clinical nutrition 99(1):35−45. doi:10.3945/ajcn.113.069393
51. Rallidis LS, Lekakis J, Kolomvotsou A, et al. (2009) Close adherence to a Mediterranean diet improves endothelial function in subjects with abdominal obesity. The American journal of clinical nutrition 90(2):263−8. doi:10.3945/ajcn.2008.27290
52. Rallidis LS, Kolomvotsou A, Lekakis J, et al. (2017) Short−term effects of Mediterranean−type diet intervention on soluble cellular adhesion molecules in subjects with abdominal obesity. Clin Nutr ESPEN 17:38−43. doi:https://dx.doi.org/10.1016/j.clnesp.2016.11.002
53. Reidlinger DP, Darzi J, Hall WL, et al. (2015) How effective are current dietary guidelines for cardiovascular disease prevention in healthy middle−aged and older men and women? A randomized controlled trial. American Journal of Clinical Nutrition 101(5):922−30. doi:http://dx.doi.org/10.3945/ajcn.114.097352
54. Retterstol K, Svendsen M, Narverud I, Holven KB (2018) Effect of low carbohydrate high fat diet on LDL cholesterol and gene expression in normal−weight, young adults: A randomized controlled study. Atherosclerosis 279:52−61. doi:https://dx.doi.org/10.1016/j.atherosclerosis.2018.10.013
55. Roussell MA, Hill AM, Gaugler TL, et al. (2012) Beef in an Optimal Lean Diet study: effects on lipids, lipoproteins, and apolipoproteins. The American journal of clinical nutrition 95(1):9−16. doi:10.3945/ajcn.111.016261
56. Sacks FM, Carey VJ, Anderson CAM, et al. (2014) Effects of High vs Low Glycemic Index of Dietary Carbohydrate on Cardiovascular Disease Risk Factors and Insulin Sensitivity: The OmniCarb Randomized Clinical Trial. JAMA : the journal of the American Medical Association 312(23):2531−41. doi:10.1001/jama.2014.16658
57. Santiago−Torres M, Kratz M, Lampe JW, et al. (2016) Metabolic responses to a traditional Mexican diet compared with a commonly consumed US diet in women of Mexican descent: A randomized crossover feeding trial. American Journal of Clinical Nutrition 103(2):366−74. doi:http://dx.doi.org/10.3945/ajcn.115.119016
58. Shikany JM, Phadke RP, Redden DT, Gower BA (2009) Effects of low− and high−glycemic index/glycemic load diets on coronary heart disease risk factors in overweight/obese men. Metabolism, clinical and experimental 58(12):1793−801. doi:10.1016/j.metabol.2009.06.006

59. Singh RB, Dubnov G, Niaz MA, et al. (2002) Effect of an Indo−Mediterranean diet on progression of coronary artery disease in high risk patients (Indo−Mediterranean Diet Heart Study): a randomised single−blind trial. The Lancet 360(9344):1455−61. doi:10.1016/s0140−6736(02)11472−3
60. Stomby A, Simonyte K, Mellberg C, et al. (2015) Diet−induced weight loss has chronic tissue−specific effects on glucocorticoid metabolism in overweight postmenopausal women. Int J Obes (Lond) 39(5):814−9. doi:10.1038/ijo.2014.188
61. Tuomainen M, Karkkainen O, Leppanen J, et al. (2019) Quantitative assessment of betainized compounds and associations with dietary and metabolic biomarkers in the randomized study of the healthy Nordic diet (SYSDIET). The American journal of clinical nutrition 110(5):1108−18. doi:https://dx.doi.org/10.1093/ajcn/nqz179
62. Uusitupa M, Hermansen K, Savolainen MJ, et al. (2013) Effects of an isocaloric healthy Nordic diet on insulin sensitivity, lipid profile and inflammation markers in metabolic syndrome – a randomized study (SYSDIET). Journal of internal medicine 274(1):52−66. doi:10.1111/joim.12044
63. Vincent−Baudry S, Defoort C, Vague P, et al. (2005) The Medi−RIVAGE study : reduction of cardiovascular disease risk factors after a 3−mo intervention with a Mediterranean−type diet or a low−fat diet. The American journal of clinical nutrition 82(5):964−71. doi:10.1093/ajcn/82.5.964
64. Vitale M, Giacco R, Laiola M, et al. (2021) Acute and chronic improvement in postprandial glucose metabolism by a diet resembling the traditional Mediterranean dietary pattern: can SCFAs play a role? Clinical nutrition (Edinburgh, Scotland) 40(2):428−37.
65. Wade AT, Davis CR, Dyer KA, Hodgson JM, Woodman RJ, Murphy KJ (2018) A Mediterranean diet supplemented with dairy foods improves markers of cardiovascular risk: results from the MedDairy randomized controlled trial. The American journal of clinical nutrition 108(6):1166−82. doi:https://dx.doi.org/10.1093/ajcn/nqy207
66. Wade AT, Davis CR, Dyer KA, Hodgson JM, Woodman RJ, Murphy KJ (2019) Effects of Mediterranean diet supplemented with lean pork on blood pressure and markers of cardiovascular risk: findings from the MedPork trial. The British journal of nutrition 122(8):873−83. doi:https://dx.doi.org/10.1017/S0007114519001168
67. Wardle J, Rogers P, Judd P, et al. (2000) Randomized Trial of the Effects of Cholesterol− lowering Dietary Treatment on Psychological Function. The American journal of medicine 108:547−53.
68. Wright *n*, Wilson L, Smith M, Duncan B, McHugh P (2017) The BROAD study: A randomised controlled trial using a whole food plant−based diet in the community for obesity, ischaemic heart disease or diabetes. Nutr Diabetes 7(3):e256. doi:10.1038/nutd.2017.3
69. Juraschek SP, Kovell LC, Appel LJ, et al. (2020) Associations Between Dietary Patterns and Subclinical Cardiac Injury: An Observational Analysis From the DASH Trial. Ann Intern Med 172(12):786−94. doi:http://dx.doi.org/10.7326/M20−0336
70. Marckmann P, Sandstrom B, Jespersen J (1994) Low−fat, high−fiber diet favorably affects several independent risk markers of ischemic heart disease: observations on blood lipids, coagulation, and fibrinolysis from a trial of middle−aged. American Journal of Clinical Nutrition 59(9):935−9.
71. Runchey SS, Pollak MN, Valsta LM, et al. (2012) Glycemic load effect on fasting and post−prandial serum glucose, insulin, IGF−1 and IGFBP−3 in a randomized, controlled feeding study. European Journal of Clinical Nutrition 66(10):1146−52. doi:10.1038/ejcn.2012.107
72. Tinker LF, Bonds DE, Margolis KL, et al. (2008) Low−Fat Dietary Pattern and Risk of Treated Diabetes Mellitus in Postmenopausal Women: The Women's Health Initiative Randomized Controlled Dietary Modification Trial. Arch Intern Med 168(14):1500−11. doi:10.1001/archinte.168.14.1500.
